# Limb Mesoderm and Head Ectomesenchyme Both Express a Core Transcriptional Program During Chondrocyte Differentiation

**DOI:** 10.3389/fcell.2022.876825

**Published:** 2022-06-17

**Authors:** Patsy Gomez-Picos, Katie Ovens, B. Frank Eames

**Affiliations:** ^1^ Department of Anatomy, Physiology, and Pharmacology, University of Saskatchewan, Saskatoon, SK, Canada; ^2^ Department of Computer Science, University of Calgary, Calgary, AB, Canada

**Keywords:** chondrocytes, GRN evolution, limb cartilage, head cartilage, GRN co-option

## Abstract

To explain how cartilage appeared in different parts of the vertebrate body at discrete times during evolution, we hypothesize that different embryonic populations co-opted expression of a core gene regulatory network (GRN) driving chondrocyte differentiation. To test this hypothesis, laser-capture microdissection coupled with RNA-seq was used to reveal chondrocyte transcriptomes in the developing chick humerus and ceratobranchial, which are mesoderm- and neural crest-derived, respectively. During endochondral ossification, two general types of chondrocytes differentiate. Immature chondrocytes (IMM) represent the early stages of cartilage differentiation, while mature chondrocytes (MAT) undergo additional stages of differentiation, including hypertrophy and stimulating matrix mineralization and degradation. Venn diagram analyses generally revealed a high degree of conservation between chondrocyte transcriptomes of the limb and head, including *SOX9*, *COL2A1*, and *ACAN* expression. Typical maturation genes, such as *COL10A1*, *IBSP*, and *SPP1*, were upregulated in MAT compared to IMM in both limb and head chondrocytes. Gene co-expression network (GCN) analyses of limb and head chondrocyte transcriptomes estimated the core GRN governing cartilage differentiation. Two discrete portions of the GCN contained genes that were differentially expressed in limb or head chondrocytes, but these genes were enriched for biological processes related to limb/forelimb morphogenesis or neural crest-dependent processes, respectively, perhaps simply reflecting the embryonic origin of the cells. A core GRN driving cartilage differentiation in limb and head was revealed that included typical chondrocyte differentiation and maturation markers, as well as putative novel “chondrocyte” genes. Conservation of a core transcriptional program during chondrocyte differentiation in both the limb and head suggest that the same core GRN was co-opted when cartilage appeared in different regions of the skeleton during vertebrate evolution.

## Introduction

The formation of cartilage is a trait with an interesting evolutionary history. While initially considered a vertebrate novelty, the presence of cartilage in vertebrate outgroups, such as hemichordates and cephalochordates, indicates that cartilage was very likely present in the ancestor to vertebrates (Rychel et al., 2006; Rychel and Swalla 2007). During vertebrate evolution, however, cartilage appeared in different parts of the body at different times. For example, the cranial skeleton appeared before the appendicular skeleton (Janvier 1996). A tantalizing hypothesis to explain this phenomenon is that a core gene regulatory network (GRN) driving chondrocyte differentiation in the head was later co-opted in the paired appendages. If true, then both limb and head chondrocytes might express a core transcriptional program underlying chondrocyte differentiation. As proof of principle, shared chondrocyte gene expression in amphioxus and vertebrates suggested that neural crest-derived cartilage evolved by co-opting a chondrocyte GRN from mesoderm or endoderm (Meulemans and Bronner-Fraser 2007; Hall and Gillis 2013; Jandzik et al., 2015). An understanding of chondrocyte differentiation, including the chondrocyte GRN, and embryonic origins of limb and head mesenchyme is required to test this hypothesis.

Endochondral ossification generally involves the differentiation of two types of chondrocyte: immature (IMM) and mature (MAT; Eames et al., 2003; Eames et al., 2004; Tamamura et al., 2005; Gentili and Cancedda 2009). Examples of IMM are proliferative and resting chondrocytes that deposit Col2 fibers and proteoglycans in the extracellular matrix, whereas examples of MAT include pre-hypertrophic and hypertrophic chondrocytes that modify immature cartilage extracellular matrix, such as by depositing Col10 fibers and mineralizing the matrix (Leboy et al., 1988; Farquharson et al., 1994; Takeda et al., 2001).

The GRN driving chondrocyte differentiation has been refined over the years (Cole and Hall 2009; Kerkhofs et al., 2012; Oh et al., 2014; Liu and Lefebvre 2015; Ohba et al., 2015; He et al., 2016; Tan et al., 2018; Hojo and Ohba 2019). Initially, a chondrocyte GRN inferred from published mammalian literature reflected the regulatory importance of SOX9 and RUNX2 on their downstream targets (Cole 2011; Kerkhofs et al., 2012). During IMM differentiation, for example, SOX9 binds to its cofactors SOX5 and SOX6 to activate expression of important cartilage differentiation markers, such as *Col2a1*, *Col9a1*, and *Acan* (Lefebvre et al., 2001; Akiyama et al., 2002; Liu and Lefebvre 2015). During MAT differentiation, SOX9 levels decrease, and RUNX2 levels increase to activate the expression of such genes as *Col10a1*, *Mef2c, Mmp13*, *Spp1*, and *Ibsp* (Ducy et al., 1997; Komori et al., 1997; Inada et al., 1999; Lee et al., 2000; Arnold et al., 2007; Li et al., 2011; Lu et al., 2014; Niu et al., 2017; Komori 2018)*.* Since cartilage maturation-like changes, such as hypertrophy and matrix degradation, play a role at different stages during osteoarthritis (OA), several of these MAT genes have also been linked to this skeletal pathology (Lamas et al., 2010; van der Kraan and van den Berg 2012; Wang et al., 2013; Lv et al., 2015; Chen et al., 2020). Later microarray analyses coupled to ChIP-seq from developing cartilage refined the GRN further. For example, analyses of newborn mouse tibia revealed interactions of SOX9 with GLI1, GLI3, and FOXA2 (Tan et al., 2018). Many studies provide valuable insight into the chondrocyte GRN, but they mostly focus on limb cartilage, so whether expression of this GRN is conserved throughout the body remains unclear.

Cartilages in the limb and head of vertebrates can have two distinct embryonic origins: mesoderm and neural crest. The appendicular skeleton within fins or limbs derives from lateral plate mesoderm, whereas cranial neural crest-derived ectomesenchyme gives rise to a large portion of the cranial skeleton, including the jaws, anterior calvarium, palate, and hyoid bone (Couly et al., 1993; Knight and Schilling 2006; Fonseca et al., 2017). IMM and MAT are present in both the limb and head, so mesenchyme derived from both mesoderm and neural crest can produce both types of chondrocyte.

Previous studies of a targetted subset of molecular markers suggested that the same GRN driving chondrocyte differentiation is expressed regardless of its embryonic origin or location in the body. For example, IMM from both limb and head express *Sox9, Sox5, Sox6, Acan*, and *Col2a1* (Lefebvre and de Crombrugghe 1998; Smits et al., 2001; Akiyama et al., 2002; Eames et al., 2004; Smits et al., 2004; Dale and Topczewski 2011; Lefebvre and Dvir-Ginzberg 2017; Xiong et al., 2018), while MAT from both limb and head express *Runx2*, *Col10a1,* and *Ihh* (Eames et al., 2004; Yoshida et al., 2004; Young et al., 2006). Moreover, if the function of any of these genes is perturbed, then chondrocytes are affected throughout the body, suggesting that the same core GRN driving chondrocyte differentiation might be expressed in both limb and head (Komori et al., 1997; Bi et al., 1999; Smits et al., 2001; Smits et al., 2004; Yoshida et al., 2004). Unbiased studies comparing gene expression globally between limb and head are needed to verify if the GRN underlying chondrocyte differentiation is the same throughout the body.

Comparative transcriptomics has revealed differences between mesenchymal precursors of limb mesoderm and cranial neural crest, but a differentiated cell type can express a core set of genes in both limb and head. Gene expression profiles from mesenchyme derived from neural crest and mesoderm that were isolated from the first pharyngeal arch using laser-capture microdissection (LCM) revealed 140 differentially expressed genes (Bhattacherjee et al., 2007). Very few studies have used transcriptomics to reveal how gene expression in skeletal cells from head and limb might vary. In perhaps the most relevant study, osteoblasts were obtained from mouse calvaria and hindlimb cortical bones, and scRNA-seq revealed that the transcriptomes of head and limb osteoblasts were highly similar (Ayturk et al., 2020). Typical osteoblast differentiation markers, including *Col1a1, Col1a2, Bglap, Ifitm5*, and *Dmp1*, were conserved in head and limb, suggesting that regardless of embryonic origin and location, osteoblasts employ a core GRN during differentiation (Ayturk et al., 2020).

**FIGURE 1:**
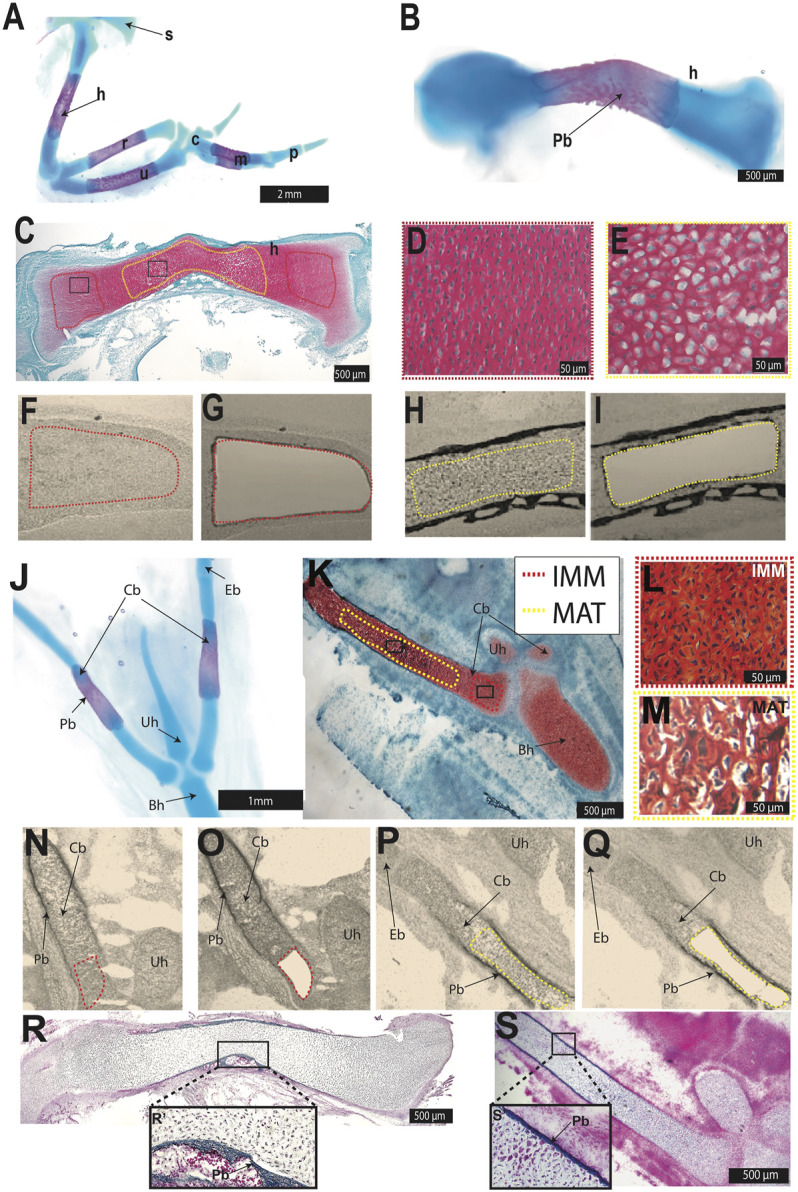
Laser capture microdissection was used to isolate chondrocytes from the chick HH36 humerus and ceratobranchial. **(A,B,J)** Whole-mount Alcian blue and Alizarin red staining identified cartilage and perichondral bone in chondral bones of the chick forelimb **(A,B)** or hyoid **(J)**. **(C,K)** Safranin O-stained section of HH36 humerus or ceratobranchial highlighted immature (IMM, red dotted outline) and mature cartilage (MAT, yellow dotted outline). **(D,E,L,M)** High-magnification images of IMM **(D,L)** and MAT **(E,M)** from black boxes in **(C)** or **(K) (F–I,N–Q)** Unstained sections of HH36 chick humerus or ceratobranchial before **(F,N)** and after **(G,O)** laser capture of IMM, and before **(H,P)** and after **(I,Q)** laser capture of MAT. **(R,S)** Trichrome-stained section of HH36 humerus or ceratobranchial showed Aniline blue staining of bone matrix in perichondral bone. Abbreviations: Bh = basihyal; c = carpal; Cb = ceratobranchial; Eb = epibranchial; h = humerus; IMM = immature chondrocytes; m = metacarpals; MAT = mature chondrocytes; p = phalanges; Pb = perichondral bone; r = radius; s = scapula; u = ulna; Uh = urohyal.

To test the hypothesis that a core GRN is expressed during chondrocyte differentiation in the limb and head, LCM coupled with RNA-seq was used to generate transcriptomes of IMM and MAT from a limb cartilage (i.e., humerus) and a head cartilage (i.e., ceratobranchial) in the chick embryo (Figure 1). Analyses of the resulting data highlight a core transcriptional program (e.g., *SOX9*, *SOX5*, *SOX6*, *COL2A1*, *ACAN,* and *COL10A1*) that drives chondrocyte differentiation throughout the body. GRN estimates from gene co-expression network (GCN) analyses showed that enriched IMM and MAT genes were negatively correlated in both the limb and head. We discuss that many genes commonly described as cartilage genes that were enriched in limb or head chondrocytes (e.g., *ID1, PAX7, ZIC1, HOXA, HOXD,* and *SHOX2*) might actually only serve that purpose in specific regions of the body, and should not be considered part of the core chondrocyte GRN. Together these data support the hypothesis that cartilage appeared in different regions of the vertebrate body during evolution by co-opting the same core GRN driving chondrocyte differentiation.

## Results

### Identification and Isolation of Limb and Head Immature and Mature Chondrocyte Transcriptomes

IMM and MAT were obtained from two chondral bones, the humerus and ceratobranchial (Figure 1), which are derived from lateral plate mesoderm and cranial neural crest, respectively (Noden 1982; Couly et al., 1993; Le Douarin et al., 1999; Burke and Nowicki 2001; Tani et al., 2020). In chick, the epiphyseal growth plate of such long bones as the humerus and ceratobranchial should contain IMM and MAT at HH36 (E10; Milz et al., 2002; Conen et al., 2009; Lui et al., 2014). Alcian blue identified cartilage, whereas Alizarin Red identified perichondral bone in whole-mount stains of both skeletal elements at HH36 (Figures 1A,B,J). Perichondral bone is often associated with underlying MAT, and Safranin O staining of histological sections demonstrated that MAT, underneath perichondral bone, have undergone hypertrophy in the mid-diaphyseal region (i.e., shaft of a long bone) of the humerus and ceratobranchial (Figures 1C–E,K–M). LCM was used to isolate IMM and MAT from the humerus and ceratobranchial (Figures 1F–I,N–Q). At HH36, vascular invasion had not yet occurred, and bone matrix was only detected in perichondral bone in both the humerus and ceratobranchial, as shown by Trichrome staining (Figures 1R,S), suggesting that no transdifferentiation of chondrocytes to osteoblasts had yet occurred (Conen et al., 2009; Zhou et al., 2014; Qin et al., 2020). RNA-seq was then carried out on RNA isolated from IMM and MAT.

### Comparative Transcriptomics Revealed Expression of a Core Set of Genes Underlying Chondrocyte Differentiation in Limb and Head

**FIGURE 2:**
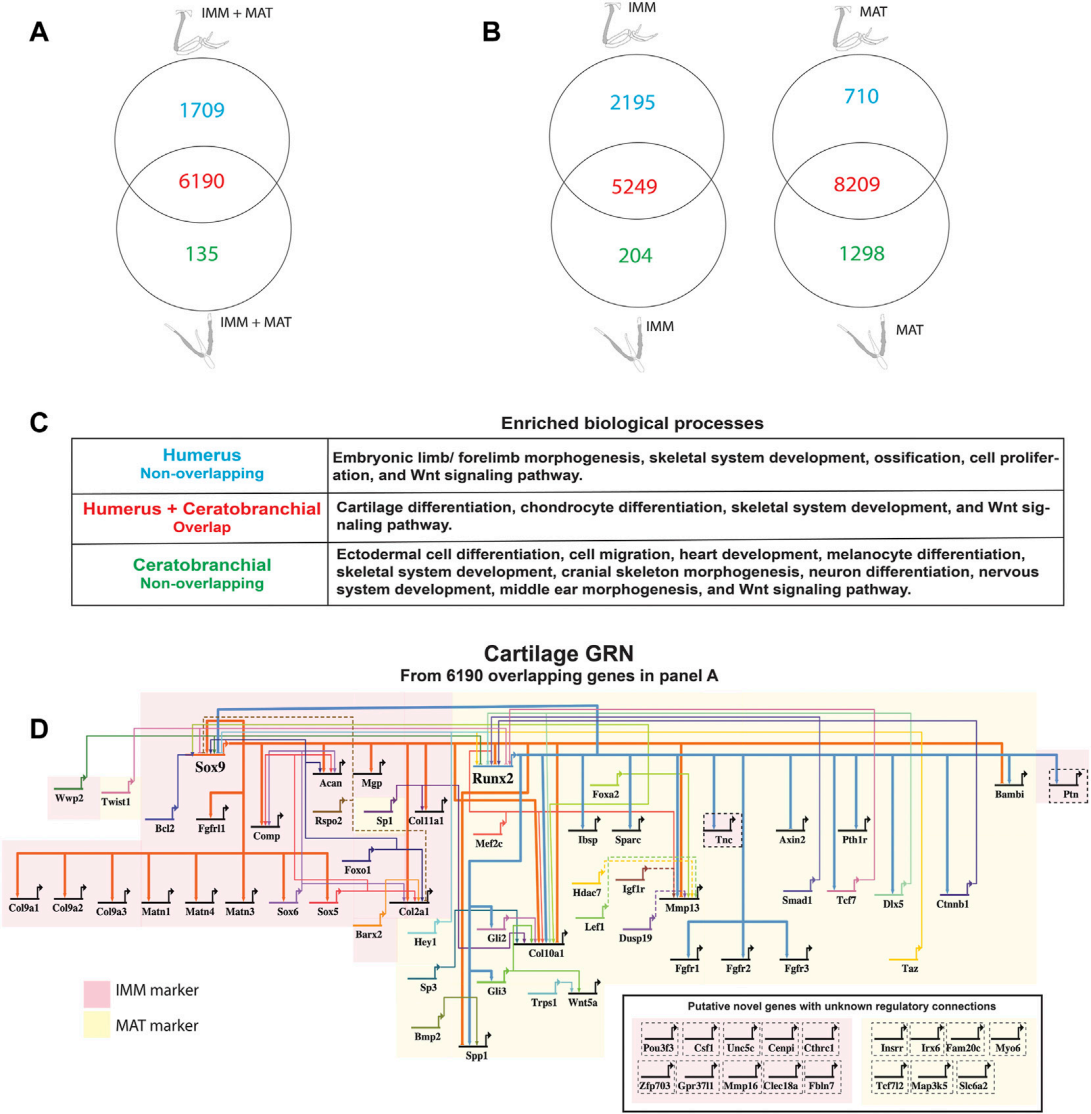
A core GRN drives chondrocyte differentiation in the limb and head. **(A)** Venn diagram demonstrated that limb and head chondrocytes shared almost 80% of the genes expressed above threshold (limb and head data combined before normalization and cutoff calculations). **(B)** Venn diagrams of IMM or MAT (separately normalized) showed that limb and head chondrocytes again shared expression of most genes. **(C)** Enriched biological processes in the overlap of panel A included cartilage differentiation processes, whereas limb morphogenesis and neural crest-related processes were enriched in the non-overlapping humerus and ceratobranchial portions, respectively. **(D)** A core cartilage gene regulatory network (GRN), compiled from the overlapping portion of panel A and published literature, included typical IMM and MAT markers highlighted in pink and yellow, respectively. Arrowheads represent positive interaction, whereas negative interactions are depicted as –|. Interactions included in this network could be direct (solid lines) or indirect (dashed lines). Novel putative cartilage genes with unknown regulatory interactions were included as single-nodes surrounded by dashed lines to the bottom right of the network, except for *PTN* and *TNC* whose regulatory interactions have been confirmed.

To test the hypothesis that a core GRN is expressed during chondrocyte differentiation in the limb and head, chondrocyte transcriptomes from the HH36 chick humerus and ceratobranchial were compared (Figure 2; Supplementary Figure S1). To identify similarities and differences among limb and head chondrocyte transcriptomes, a principal component analysis (PCA) was performed (Supplementary Figure S1). The variation in the samples was captured with two components (39% variance explained by PC1 and PC2; Supplementary Figure S1). The limb IMM and MAT transcriptomes were separated from other samples in PC1/PC2 with 95% confidence, while head IMM and MAT transcriptomes overlapped in PC1/PC2 (Supplementary Figure S1).

When IMM and MAT datasets from limb and head were combined before normalization (i.e., IMM and MAT were considered as the same cell type) to reflect generally the chick “chondrocyte”, limb and head chondrocytes shared 77% of genes expressed above threshold (6190/8034 genes; Figure 2A; see normalization techniques in Methods). Limb and head chondrocytes expressed typical IMM genes, such as *SOX9, SOX5, SOX6, ACAN,* and *COL2A1*, and typical MAT genes, including *RUNX2, COL10A1, MEF2C, MMP13*, *IBSP*, and *SPP1* at high levels (Tables 1, 2; Vortkamp et al., 1996; Zhao et al., 1997; Bridgewater et al., 1998; Watanabe et al., 1998; Bi et al., 1999; Smits et al., 2001; Smits et al., 2004; Arnold et al., 2007; Nicolae et al., 2007; Dy et al., 2012; Lu et al., 2014; Nakatani and Partridge 2017).

**TABLE 1:** Normalized gene expression counts of relevant cartilage genes in chick limb.

Gene ID	^a^Rank (outof 8647 genes above threshold)	Average counts IMM	Average counts MAT	Average counts IMM + MAT	%Total counts	Fold change over average gene counts
*COL9A1*	3	141,605	127,189	133,586	1.554	134.4
*MATN1*	9	44,056	74,224	60,816	0.707	61.2
*COL9A2*	10	78,652	39,838	57,089	0.664	57.4
*COL9A3*	12	62,622	44,541	52,577	0.611	52.9
*SPP1*	17	45	66,839	37,153	0.432	37.4
*COL11A1*	19	53,577	15,067	32,182	0.374	32.4
*COL2A1*	54	23,233	12,351	17,188	0.200	17.3
*MMP13*	69	12	24,590	13,667	0.159	13.7
*ACAN*	90	12,258	9,403	10,672	0.124	10.7
*SPARC*	113	2,489	13,801	8,773	0.102	8.8
*SOX5*	471	3,427	1,370	2,284	0.027	2.3
*MEF2C*	621	43	3,416	1,917	0.022	1.9
*RUNX2*	1,139	894	1,436	1,195	0.014	1.2
*COL10A1*	1,318	1	1,896	1,054	0.012	1.1
*PTH1R*	1,459	607	1,268	974	0.011	1.0
*SOX9*	1,597	1,203	663	903	0.011	0.9
*SOX6*	3,750	591	187	366	0.004	0.4
*IBSP*	7,157	10	141	83	0.001	0.1

a 9/30 highest genes were mitochondrial genes.

**TABLE 2:** Normalized gene expression counts of relevant cartilage genes in chick head.

Gene ID	^a^Rank (outof 8647 genes above threshold)	Average counts IMM	Average counts MAT	Average counts IMM + MAT	%Total counts	Fold change over average gene counts
*COL9A1*	13	80,955	32,828	56,892	0.511	50.2
*COL9A3*	39	32,205	15,072	23,638	0.212	20.9
*MATN1*	41	12,180	33,875	23,028	0.207	20.3
*COL9A2*	47	25,777	16,637	21,207	0.191	18.7
*COL11A1*	79	18,755	8,303	13,529	0.122	11.9
*COL2A1*	147	12,802	2,321	7,562	0.068	6.7
*SPARC*	175	8,291	3,650	5,970	0.054	5.3
*ACAN*	211	5,117	4,714	4,916	0.044	4.3
*MEF2C*	214	1,029	8,727	4,878	0.044	4.3
*SOX5*	329	1,295	5,038	3,166	0.028	2.8
*RUNX2*	1,162	179	2,025	1,102	0.010	1.0
*PTH1R*	1,217	219	1,888	1,054	0.009	0.9
*SOX9*	1,979	656	668	662	0.006	0.6
*SPP1*	2,508	25	1,014	519	0.005	0.5
*COL10A1*	2,863	37	861	449	0.004	0.4
*SOX6*	3,461	306	395	351	0.003	0.3
*MMP13*	4,820	54	355	205	0.002	0.2
*IBSP*	5,637	1	286	143	0.001	0.1

a 10/30 hightest genes were mitochondrial genes.

When IMM and MAT were each compared separately between the limb and head before normalization (i.e., IMM and MAT were considered as different cell types), each cell type still shared the vast majority of genes expressed above threshold in both the limb and head (for IMM: 5249/7648 = 69%; for MAT: 8209/10,217 = 80%; Figure 2B). Overlapping genes in the limb and head again included many typical cartilage genes, such as *SOX9*, *COL2A1*, *ACAN*, and *COL9A1* for IMM, and *RUNX2*, *COL10A1*, *SPP1*, *MMP13*, and *IBSP* for MAT (Tables 1, 2). Gene ontogeny analyses on IMM and MAT from both limb and head demonstrated that cartilage-specific processes were enriched and conserved between the limb and head, even though genes associated exclusively with these processes only comprise approximately 2% of the GO term-associated genes (Figure 2C). The most enriched biological processes (>60% genes expressed above threshold) were related to basic cellular processes, such as cell proliferation, cell differentiation, transcription, and translation. Collectively, these data suggest that a core GRN driving chondrocyte differentiation is expressed in different regions of the skeleton.

Using published work on mouse, chick, frog, and fish, regulatory interactions among important genes from chick chondrocyte transcriptomes were summarized into a GRN (Figure 2D; Longabaugh et al., 2005). As master regulators of IMM and MAT, respectively, *SOX9* and *RUNX2* were placed at the top of the GRN hierarchy (Figure 2D; Komori et al., 1997; Bi et al., 1999; Lian and Stein 2003; Eames et al., 2004). In the IMM portion of the GRN (highlighted in pink in Figure 2D), SOX9 binds to SOX5 and SOX6 during early stages of chondrocyte differentiation and activates the expression of typical IMM markers, such as *COL2A1*, *COL9A1*, and *ACAN* (Lefebvre et al., 2001; Akiyama et al., 2002; Liu and Lefebvre 2015). In the MAT portion of the GRN (highlighted in yellow in Figure 2D), RUNX2 activates the expression of typical MAT markers, including *COL10A1*, *MMP13*, *SPP1*, and *IBSP* (Ducy et al., 1997; Komori et al., 1997; Inada et al., 1999; Inada et al., 1999; Lee et al., 2000; Wang et al., 2009; Li et al., 2011; Peacock et al., 2011; Lu et al., 2014). Since *SOX9* and *RUNX2* generally exhibit an antagonistic relationship, SOX9 inhibits the expression of MAT markers, such as *RUNX2*, *SPP1*, and *IBSP,* while RUNX2 inhibits the expression of *SOX9* and thus likely other IMM markers (Figure 2D; Zhou et al., 2006; Cheng and Genever 2010; Peacock et al., 2011; Lui et al., 2019).

Genes expressed above threshold only in limb or head might reflect the embryonic origin of the cells. For genes located in the humerus portion of the Venn diagrams, such as *TBX5, DLX6, SALL4, HOXA10,* and *HOXD10*, embryonic limb/forelimb morphogenesis was an enriched process, and these genes are all known regulators of limb development (Figure 2B; Wahba et al., 2001; Robledo et al., 2002; Zakany and Duboule 2007; Vieux-Rochas et al., 2013; Neufeld et al., 2014; Akiyama et al., 2015). More general skeletal processes, such as skeletal system development and ossification, were also enriched in limb chondrocytes. Many neural crest-dependent biological processes were enriched specifically in ceratobranchial IMM and MAT transcriptomes, such as cranial skeleton morphogenesis, cell migration, neuron differentiation, middle ear morphogenesis, heart development, and melanocyte differentiation (Figure 2B). Many orthologs of a proposed GRN driving neural crest-derived cartilage, such as *PAX7*, *SIX1*, and *ID1*, were also identified (Figure 2B; Meulemans and Bronner-Fraser 2005; Meulemans and Bronner-Fraser 2007; Betancur et al., 2010; Murdoch et al., 2012; Wu et al., 2019).

### Differentially Expressed Genes in Immature Chondrocytes Were Negatively Correlated With Those of Mature Chondrocytes During Differentiation in Both the Limb and Head

**FIGURE 3:**
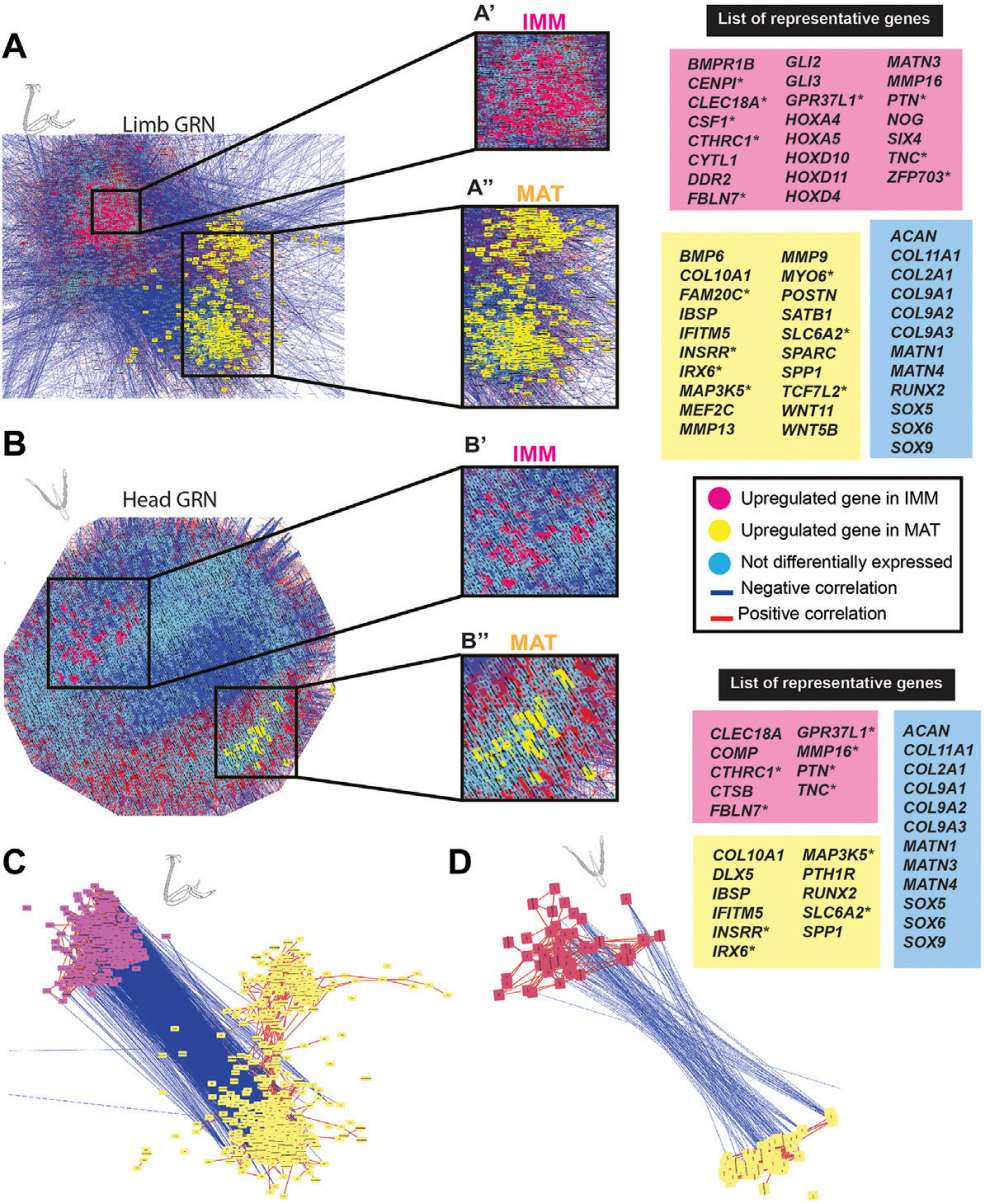
GCN of either limb or head chondrocyte data was organized into groups of enriched IMM or MAT genes that were negatively correlated with each other. **(A,B)** A GRN underlying chondrocyte differentiation was estimated from a gene co-expression network (GCN) from limb **(A)** or head **(B)** chondrocytes (separately normalized). In both limb and head GRNs, one portion contained genes upregulated in IMM (pink nodes), while another portion contained genes upregulated in MAT (yellow nodes). Typical chondrocyte differentiation genes included in the network (light blue nodes) were not differentially expressed between IMM and MAT. Representative lists of genes in each of these three categories are shown to the side of the GRNs. These candidate genes were selected because they are known to have a role during cartilage differentiation in different vertebrates, and they are listed in alphabetical order. Putative novel genes are indicated by an asterisk next to the gene name. **(C,D)** Isolated portions of IMM- and MAT-enriched genes from the limb **(C)** or head **(D)** GRNs. Most upregulated genes in IMM or MAT were connected by positive interactions (red lines), whereas IMM and MAT genes were mostly connected by negative interactions (blue lines).

GRNs rely upon functional data to verify regulatory interactions, but such studies are limited (Figure 2D; Su et al., 2009; Peter and Davidson 2011). To infer regulatory interactions underlying chondrocyte differentiation, and to compare GRN organization in the limb and head, GRNs were estimated using Cytoscape to graph gene co-expression networks (GCNs) of chondrocyte transcriptomes of the HH36 humerus or ceratobranchial (McCall 2013; Khosravi et al., 2015). All genes expressed above threshold were used to construct these GCNs (>8,000 genes, Figures 2A,B). GRNs of both limb and head data were organized into two large groups of genes expressed during chondrocyte differentiation (Figures 3A,B). Expression within each group was positively correlated (red lines in Figures 3A,B), but expression between the two groups was negatively correlated (blue lines in Figures 3A,B). One group was enriched for genes that were differentially expressed in IMM, and the other group was enriched for genes that were differentially expressed in MAT (for ease of view, selected IMM and MAT differentially expressed genes of limb and head chondrocytes are depicted in Figures 3C,D, respectively).

In the limb, these two portions of the GRN included 859 genes [absolute log2 fold change greater than 2 (*p* < 0.01)] that were differentially expressed between IMM and MAT of the HH36 chick humerus (see Supplementary Tables S1, S2 for a full list of genes). A total of 263 genes were upregulated in IMM, whereas 596 genes were upregulated in MAT (Figure 3A; Supplementary Tables S1, S2). Upregulated genes in IMM included *MATN3, GLI2, GLI3*, *DDR2,* and *NOG*, and also some *HOX* genes that have a role during chondrocyte differentiation in the limb (Figure 3A, labelled pink in GRN; Koziel et al., 2005; Kruger and Kappen 2010; Tan et al., 2018; Yamamoto et al., 2019). Typical maturation genes were upregulated in MAT, including *COL10A1, MMP13, SPP1, MEF2C, IBSP*, and *SPARC* (Figure 3A, labelled yellow in GRN; Bianco et al., 1991; D'Angelo et al., 2000; Arnold et al., 2007; Peacock et al., 2011; Lu et al., 2014; Rosset and Bradshaw 2016). These differences in gene expression patterns were also demonstrated by unsupervised model-based clustering analysis (Supplementary Table S2). Some clusters showed enriched expression of genes in IMM including hallmark cartilage genes, while others showed enhanced expression in MAT including several important maturation markers (Supplementary Table S2). Typical chondrocyte differentiation genes, such as *SOX9*, *SOX5*, *SOX6*, *COL2A1*, and *ACAN* showed high expression levels in both IMM and MAT of the HH36 chick humerus (Figure 3A, labelled blue in GRN; Tables 1, 2).

In the head, the two portions of the GRN included 118 genes that were differentially expressed between IMM and MAT of the HH36 chick ceratobranchial (see Supplementary Tables S3, S4 for a full list of genes). A total of 70 genes were upregulated in IMM, whereas 48 genes were upregulated in MAT (Figure 3B; Supplementary Tables S3, S4). Upregulated genes in IMM included *COMP* and *MMP16* (Figure 3B, labelled pink in GRN; Hecht et al., 2005). Typical maturation genes were upregulated in MAT, including *RUNX2*, *COL10A1, IBSP,* and *SPP1* (Figure 3B, labelled yellow in GRN; Bianco et al., 1991; Komori and Kishimoto 1998; Arnold et al., 2007; Peacock et al., 2011; Lu et al., 2014).

**FIGURE 4:**
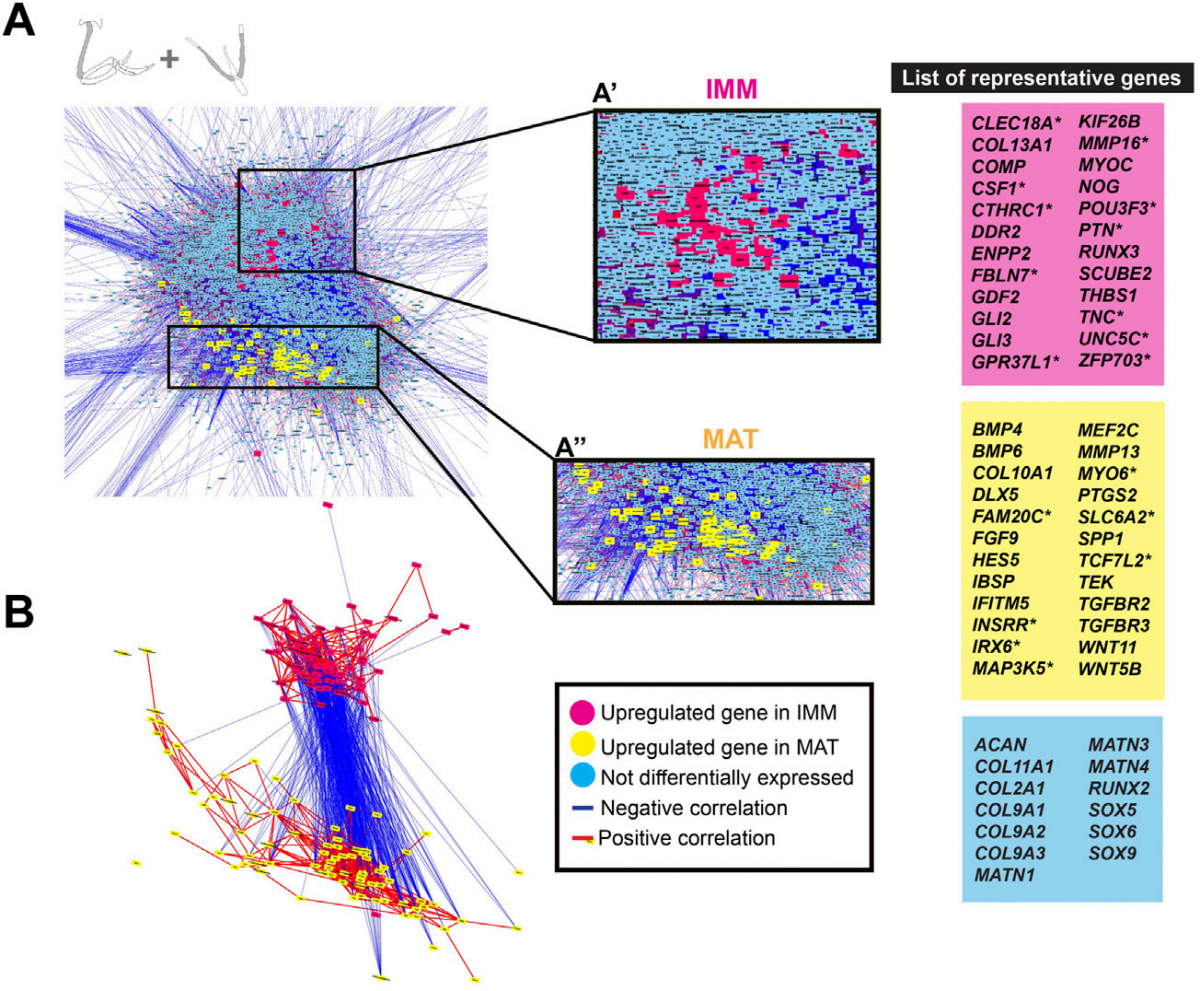
GCN of combined limb and head chondrocyte data was organized into groups of enriched IMM or MAT genes that were negatively correlated with each other. **(A)** A GRN underlying chondrocyte differentiation was estimated from a GCN from limb and head chondrocytes (normalized together). One portion of the GRN contained genes upregulated in IMM (pink nodes), while another portion contained genes upregulated in MAT (yellow nodes). Typical chondrocyte differentiation genes included in the network (light blue nodes) were not differentially expressed between IMM and MAT. Representative lists of genes in each of these three categories are shown to the side of the GRNs. These candidate genes were selected because they are known to have a role during cartilage differentiation in different vertebrates, and they are listed in alphabetical order. Putative novel genes are indicated by an asterisk next to the gene name. **(B)** Isolated portions of IMM- and MAT-enriched genes in the GRN. Most upregulated genes in IMM or MAT were connected by positive interactions (red lines), whereas IMM and MAT genes were mostly connected by negative interactions (blue lines).

To directly visualize an estimate of the general chick chondrocyte GRN, limb and head data were combined before normalization and graphed as a GCN. The overall organization of this GRN was similar to that for just limb or head chondrocyte data. Two portions of positively correlated genes that were differentially expressed in each type of chondrocyte (i.e., IMM or MAT) were negatively correlated with each other (Figures 4A,B). A total of 458 genes were differentially expressed between IMM and MAT (see Supplementary Tables S5, S6 for a full list of genes). Of these, 195 genes were upregulated in IMM, and 263 genes were upregulated in MAT (Supplementary Tables S5, S6). Upregulated genes in IMM and MAT included many of the same genes that were upregulated in either limb or head data alone (compare genes labelled pink in Figures 4A,B to Figures 3A,B). Other upregulated IMM genes were not previously implicated in chondrocyte differentiation, such as those encoding the transcription factors POU3F3 and ZFP703, growth factors CSF1 and PTN, and the Netrin receptor UNC5C (Figures 4A,B, labelled pink; Cecchini et al., 1997; Tare et al., 2002; Srivatsa et al., 2014; Kumar A. et al., 2016; Kumar S. et al., 2016). Upregulated genes in MAT included *IFITM5*, whose role had only been studied previously in osteoblast differentiation, and many other signalling pathway genes previously implicated in cartilage maturation, including *BMP4, FGF9, HES5, TEK, TGFBR2, TGFBR3, WNT5B,* and *WNT11* (Figures 4A,B, labelled yellow; Hoffmann and Gross 2001; Yang et al., 2003; Karlsson et al., 2010; Shen et al., 2013; Usami et al., 2016; Zhang et al., 2021). Additional genes upregulated in MAT had never been associated previously with chondrocyte maturation, including those encoding the kinase FAM20C and transcription factor TCF7L2 (Hirose et al., 2020; Li et al., 2021).

Although typical chondrocyte differentiation genes, such as *SOX9* and *COL2A1*, were not differentially expressed between limb and head (Figures 3, 4), differences in gene expression were obvious when the normalized counts were compared (Tables 1, 2). In the limb, for instance, the SOX trio (*SOX9, SOX5*, and *SOX6*) showed higher expression levels in IMM, and expression levels decreased in MAT, as reported by others (Ikegami et al., 2011; Lui et al., 2019). In the head, however, expression levels of *SOX9* and *SOX6* remained at comparable levels in IMM and MAT, and MAT even showed higher levels of *SOX5* expression compared to IMM. A similar situation is observed when *RUNX2* levels are compared between limb and head. In the head, *RUNX2* expression strikingly increases during IMM to MAT transition (Tables 1, 2; Figure 3B), while in the limb this increase in *RUNX2* expression is not as as dramatic (Tables 1, 2). These differences in the expression might be the result of variation in the timing of cartilage maturation among skeletal elements, as well as differences related to growth and shape of endochondral bones in distinct locations of the body (Chiba et al., 1995; Patton and Kaufman 1995; Mitgutsch et al., 2011). Together, these data demonstrated that the GRN driving chondrocyte differentiation is organized similarly throughout the body and provided novel genes that might regulate IMM and MAT differentiation.

### Patterning Genes Expressed During Chondrocyte Differentiation Were Enriched in the Limb or Head

**FIGURE 5:**
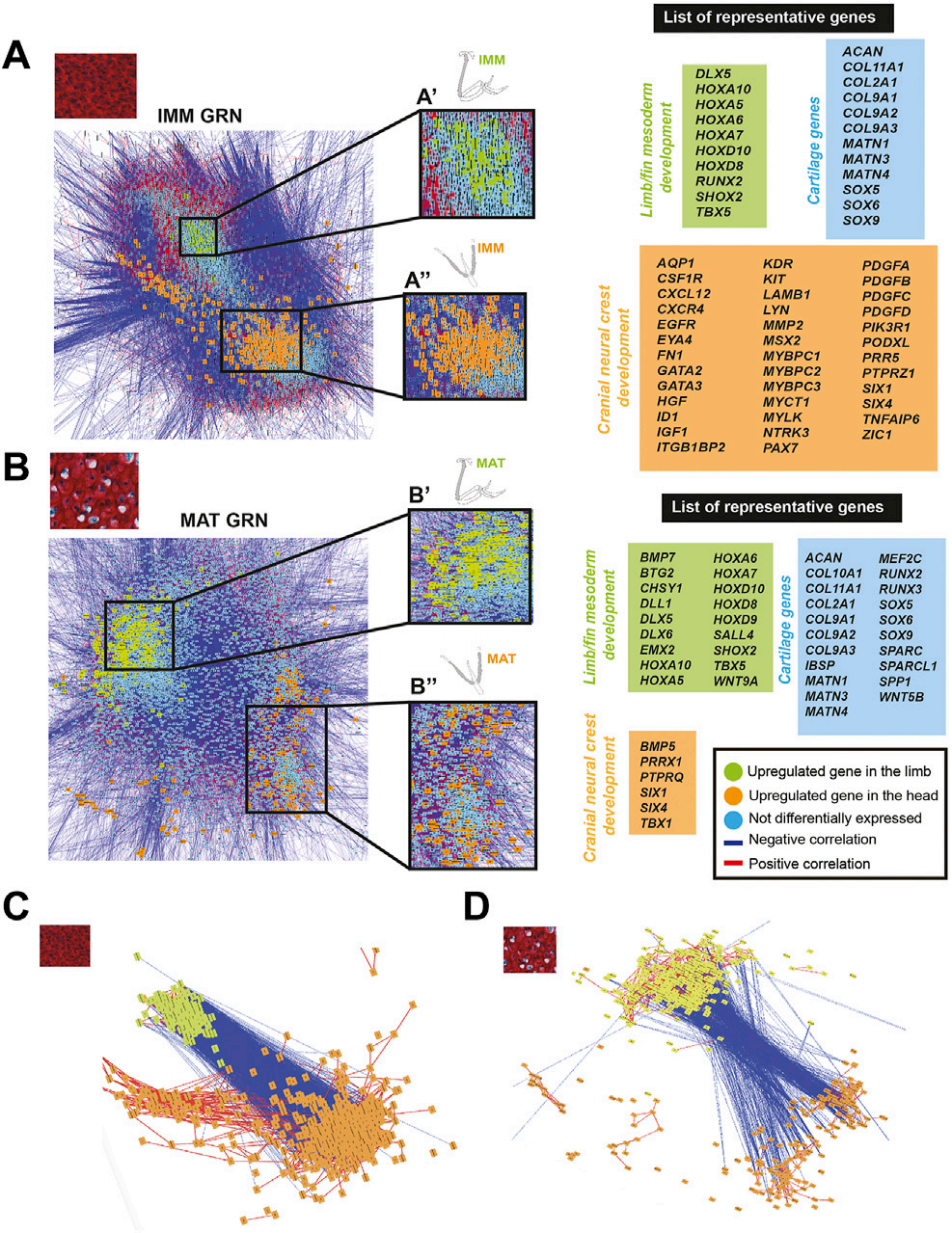
GCN of either IMM or MAT chondrocyte data was organized into groups of enriched limb or head genes that were negatively correlated with each other. **(A,B)** A GRN underlying IMM **(A)** or MAT **(B)** differentiation was estimated from a GCN from limb and head chondrocytes (IMM and MAT data separately normalized). In both IMM and MAT GRNs, one portion contained genes upregulated in the limb (green nodes), while another portion contained genes upregulated in the head (orange nodes). Typical chondrocyte differentiation genes included in the network (light blue nodes) were not differentially expressed between limb and head. Representative lists of genes in each of these three categories are shown to the side of the GRNs. **(C,D)** Isolated portions of limb- and head-enriched genes in the IMM **(C)** and MAT **(D)** GRNs. Most upregulated genes in limb or head were connected by positive interactions (red lines), whereas limb and head genes were mostly connected by negative interactions (blue lines).

To identify genes that might influence which type of chondrocyte differentiates in specific embryonic regions, IMM or MAT data from the limb and head was used to estimate a GRN underlying IMM or MAT differentiation by graphing a GCN. Two portions of each GRN were identified, this time enriched for genes that were differentially expressed in chondrocytes of the limb or the head (Figures 5A,B). Many of these region-specific genes were positively correlated to each other, while negatively correlated to genes enriched in chondrocytes from the other embryonic region (Figures 5A–D). Many of these genes were patterning genes of the limb or head.

Genes upregulated in limb chondrocytes were very similar for the IMM and MAT data, while genes upregulated in head chondrocytes varied among IMM and MAT data. The two portions of the IMM GRN included 1223 genes that were differentially expressed between limb and head chondrocytes (Figure 5A; see Supplementary Tables S7, S8 for a full list of genes). A total of 216 genes were upregulated in limb IMM, while 1,007 genes were upregulated in head IMM (Supplementary Tables S7, S8). The two portions of the MAT GRN included 1,215 genes that were differentially expressed between limb and head chondrocytes (Figure 5B; Supplementary Tables S9, S10). A total of 664 genes were upregulated in limb MAT, whereas 551 genes were upregulated in head MAT (Supplementary Tables S9, S10). Upregulated genes in limb IMM and MAT included several *HOX* genes, *DLX5, TBX5,* and *SHOX2,* all of which are known to have a role during limb morphogenesis (Figures 5A,B, labelled green in GRN; Agarwal et al., 2003; Koziel et al., 2005; Yu et al., 2007; Kruger and Kappen 2010; Vieux-Rochas et al., 2013; Tan et al., 2018; Yamamoto et al., 2019). The other portion of the IMM GRN included genes upregulated in the head that are involved in neural crest related processes, such as *ZIC1*, *SIX1, SIX4, PAX7*, *ID1*, *GATA2*, *GATA3*, and *MSX2*, as identified by gene ontology analyses and previous studies (Figure 5A, labelled orange in the GRN; Han et al., 2007; Murdoch et al., 2012; Simões-Costa and Bronner 2013; Garcez et al., 2014; Plouhinec et al., 2014; Simões-Costa and Bronner 2015; Wu et al., 2019; Seal and Monsoro-Burq 2020). Similar to the IMM GRN, *SIX1,* and *SIX4* were also upregulated in the head in one portion of the MAT GRN. Also upregulated in head MAT were *TBX1* and *PRRX1*, which actually can regulate both limb and cranial neural crest (Figure 5B, labelled orange in GRN; Moraes et al., 2005; Balic et al., 2009; Simões-Costa and Bronner 2015). Similar results were obtained when IMM and MAT data from the limb and head were considered as four separate datasets before normalization, and included in the same GRN (Supplementary Figure S3). Unsupervised model-based clustering analyses also identified specific categories of gene expression when comparing limb and head chondrocyte data (Supplementary Figure S2). Some clusters showed enriched expression of genes in the limb, including several classic limb patterning genes, while other clusters showed increased expression in the head, including many genes involved in cranial neural crest differentiation (Supplementary Figure S2). Again, comparisons between limb and head transcriptomes revealed that typical IMM and MAT genes, including *SOX9*, *COL2A1*, *RUNX2*, *COL10A1*, and *SPP1*, were not differentially expressed between head and limb, supporting the hypothesis that a core transcriptional program driving chondrocyte differentiation is expressed wherever cartilage forms in the body.

## Discussion

Perhaps the most interesting chapter in the story of cartilage evolution is that cartilage appeared in different parts of the body at different times during vertebrate evolution. How could this have happened? Once the ability to differentiate cartilage was encoded in an ancestor’s genome as a core GRN, then adding cartilage to another location in the body might only require that a different population of cells co-opt expression of this GRN (Eames et al., 2020). This hypothesis is realistic, because *SOX9* sits at the top of the chondrocyte GRN hierarchy, so perhaps during evolution only a few regulatory elements were added to *SOX9* for cartilage to form in another embryonic region.

**FIGURE 6:**
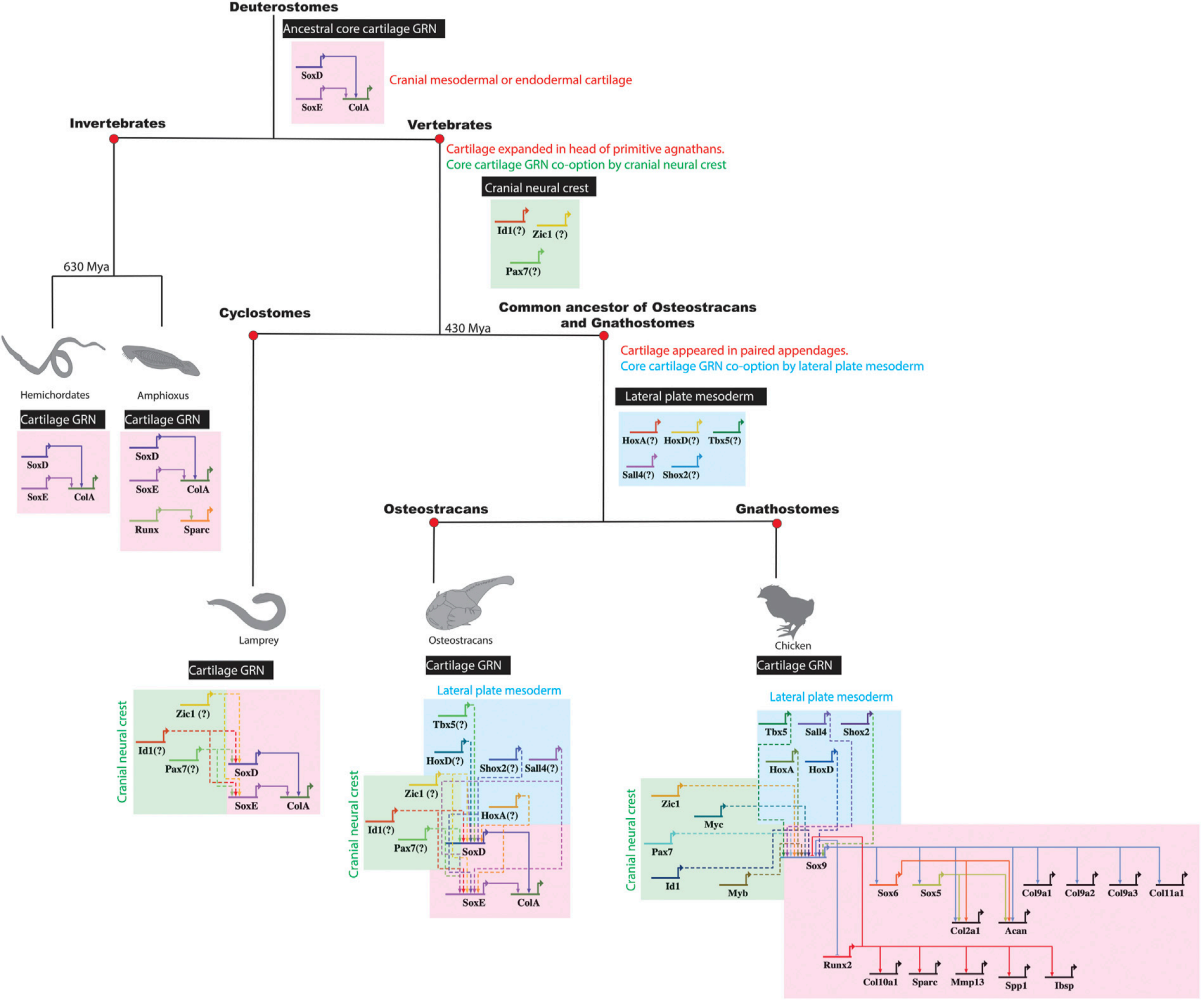
Co-option of a core cartilage GRN by different embryonic populations during vertebrate evolution. The ancestral core cartilage GRN, shared in the common ancestor of vertebrates and their sister invertebrates, likely included such genes as *SoxD*, *SoxE*, and *ColA* expressed in cranial mesoderm or endoderm. In primitive agnathan vertebrates, cranial cartilage expanded when cranial neural crest cells co-opted a core cartilage GRN, likely building new regulatory connections between cranial neural crest genes and *SoxE* orthologs. Later, when paired appendages with cartilage evolved in the common ancestor of osteostracans and gnathostomes, a core cartilage GRN was additionally co-opted by lateral plate mesoderm, likely by establishing new regulatory connections between lateral plate mesoderm genes and *SoxE* orthologs.

As proof of principle, data argues that cartilage appeared in a different embryonic population at the origin of vertebrates by GRN co-option. The GRN driving chondrocyte differentiation of invertebrates in cranial mesoderm or endoderm contains *Sox9*-like (*SoxE*), *Sox5/6*-like (*SoxD*), and *Col2a1*-like (*ColA*) genes (Rychel et al., 2006; Cattell et al., 2011; Jandzik et al., 2015; Tarazona et al., 2016). Regulatory connections in the chondrocyte GRN seem conserved among invertebrates and vertebrates (Figure 6). For example, SoxE from a hard-shell invertebrate activated expression of the human *COL2A1* gene (Tarazona et al., 2016). In the ancestral vertebrate, *SoxE* regulatory elements, and thus the core cartilage GRN, might have been co-opted from cranial mesoderm or endoderm by cranial neural crest to expand cartilage in the ancestral vertebrate head (Meulemans and Bronner-Fraser 2007; Hall and Gillis 2013; Jandzik et al., 2015).

After the origin of vertebrates, did lateral plate mesoderm similarly co-opt a core chondrocyte GRN? Cartilage was in the head of primitive agnathans, such as ancestors of cyclostomes, before the evolution of paired appendages, such as pelvic or pectoral fins (Figure 6; Janvier 1996; Berendsen and Olsen 2015). While extant cyclostomes lack paired appendages, the fossil record reveals that some extinct agnathans (e.g., osteostracans) had evolved paired appendages with cartilage (Janvier et al., 2004; Adachi et al., 2016). If cartilage in paired appendages of osteostracans was derived from lateral plate mesenchyme, and if the common ancestor of osteostracans and gnathostomes shared this feature, then all subsequent lineages of vertebrates might retain features of lateral plate mesoderm co-opting expression of a core GRN underlying chondrocyte differentiation (Figure 6). In this case, chondrocytes in the limb and head of all living gnathostomes might express the same core transcriptional program making cartilage.

Before discussing our data testing similarities between limb and head chondrocyte transcriptomes, what is meant by “core GRN”? Ultimately, GRNs derive from an organism’s genome, which is the same in most cells of the body. Here, we propose that the core GRN of a chondrocyte is the set of genes and their regulatory connections that are required for the organism to produce this cell type (Figure 6). In creating cartilage, the chondrocyte largely functions to secrete ECM, so genes encoding ECM molecules and the transcription factors that regulate them are likely key components of the chondrocyte core GRN (Gray and Williams, 1989; Gentili and Cancedda 2009; Hojo and Ohba 2019; Neefjes et al., 2020). In support of this definition, ECM genes were among the most highly expressed genes in all chondrocyte transcriptome data presented here. Regarding the idea that genes in the core GRN are required for an organism to produce a chondrocyte, we also discuss below examples of genes that are expressed in a region-specific manner. While debatable, we have argued that such genes are not part of the core chondrocyte GRN, because their loss-of-function produces only a region-specific loss of cartilage (Eames et al., 2020). Such genes, which often include transcription factors and growth factors that might act as region-specific cartilage competency or morphogenesis factors, would not be required for an organism to produce a chondrocyte, since chondrocytes are still produced in other parts of the organism’s body. Admittedly, according to another definition of core GRN, conclusions from our data might differ, but we hope this discussion helps to focus efforts on understanding the evolution of cell types (Arendt 2008; Achim and Arendt 2014; Sachkova and Burkhardt 2019; Callier 2020).

Here, comparative transcriptomics supported the hypothesis that a core GRN driving chondrocyte differentiation is expressed in cartilage of the limb and head. Venn diagram analyses revealed a large overlap (∼75%) in gene expression between the transcriptomes of chick chondrocytes isolated from the humerus and the ceratobranchial. Gene ontology analyses revealed that biological processes related to cartilage differentiation were enriched in genes shared between limb and head chondrocytes. The master chondrocyte differentiation gene, *SOX9*, as well as many other genes that are regulated by this transcription factor, such as *SOX5*, *SOX6, COL2A1, COL9A1*, *COL10A1*, and *ACAN*, were not differentially expressed between limb and head chondrocytes, as previously suggested by others, however differences in gene expression were identified when comparing normalized gene counts in limb and head (Tables 1, 2; Lefebvre et al., 1997; Zhao et al., 1997; Bi et al., 1999; Sekiya et al., 2000; Smits et al., 2001; Akiyama et al., 2002; Zhang et al., 2003; Eames and Helms 2004; Dy et al., 2012; Gu et al., 2012). Since the timing of cartilage maturation and ossification can vary among skeletal elements, future studies should assess these differences in gene expression by providing a detailed timeline of maturation in limb versus head, given the limitations of the current data. Importantly, the core GRN presented here also shows deep conservation when comparing the present data with mouse limb RNA-seq and scRNA-seq datasets. Many of the typical cartilage genes included in the core GRN, such as *Sox9, Sox5, Sox6, Acan*, *Col2a1*, *Col10a1*, and *Runx2*, were conserved between mouse and chick (Ayturk et al., 2020; Duan et al., 2020; Sebastian et al., 2021). These data clearly support the hypothesis that a core GRN driving chondrocyte differentiation is expressed throughout the body, and perhaps it has not been modified dramatically during evolution.

Interestingly, some other genes that have little or no known role in chondrocyte differentiation showed high expression levels in both the limb and head. For example, novel putative cartilage genes in IMM include those encoding the transcription factors POU3F3 and ZFP703 and the growth factor signalling members CSF1, PTN, and UNC5C, and other genes, such as *CENPI, CLEC18A, CTHRC1, FBLN7, GPR37L1, MMP16*, and *TNC.* Putative mature cartilage genes upregulated in MAT included *INSRR, IRX6, MAP3K5, MYO6*, *SLC6A2*, *FAM20C*, and the transcription factor *TCF7L2* (Figure 2D)*.* Importantly, several of these putative novel genes, including *Ptn, Tnc, Unc5c*, and *Fbln7*, were also found to be expressed above threshold in mouse limb RNA-seq and scRNA-seq datasets (Ayturk et al., 2020; Duan et al., 2020; Sebastian et al., 2021). In addition, orthologs for most of these novel candidate genes are present in the genome of other vertebrates, including bony fishes, reptiles, amphibians, and cartilaginous fishes, and even some invertebrates, but only a few have been shown to be expressed in chondral bones of non-mammalian vertebrates, such as frog and fish (Nalbant et al., 2005; Geurtzen et al., 2014; Russell et al., 2014; Square et al., 2015; Barske et al., 2020; Kraus et al., 2022). Also, many of these novel putative core cartilage GRN genes have been found to be expressed in OA cartilage, and even to have a role during the progression of this skeletal disease, further supporting the importance of these genes during cartilage development (Mentlein 2007; Johnson et al., 2015; Sun et al., 2018; Zhang et al., 2018; Bhattaram and Jones 2019; Rice et al., 2019; Chakraborty et al., 2020; Hasegawa et al., 2020; Shi et al., 2022). Future loss of function experiments in these and other vertebrates will be required to assess whether these genes indeed qualify as cartilage core genes.

The GRN driving chondrocyte differentiation, derived from both traditional methods and from GCN analyses, also had very similar organization using transcriptomic data from limb or head chondrocytes. Traditionally, GRNs are derived from functional experiments that identify positive or negative regulatory relationships between genes involved in a given biological process, such as mesoderm formation (Davidson and Erwin 2006; Peter and Davidson 2011; Erkenbrack 2016). Using published data on regulatory interactions among genes that were shared from our limb and head chondrocyte transcriptomes, we expanded upon the first published traditional chondrocyte GRN (Figure 2C; Cole 2011). Seven transcription factors (*ATF3, DLX6, FOXA3, FOXK2, FOXN2, FOXO1, RUNX3,* and *SOX8*) with no known regulatory connections and many other genes were incorporated into the chondrocyte GRN. This GRN also featured inhibition of MAT genes by *Sox9*, an IMM gene (Zhou et al., 2006; Peacock et al., 2011; Lui et al., 2019).

GCNs also can reveal regulatory relationships among genes (Stuart et al., 2003; McCall 2013; Khosravi et al., 2015), and independent estimation of the chondrocyte GRN from GCNs of our transcriptomic data confirmed and expanded the traditional approach. For both limb and head data, positive correlations between cell-type enriched genes were observed within the same cell type (IMM or MAT), likely driven by Sox9 and Runx2 activity for IMM and MAT, respectively (Eames et al., 2004; Cole 2011; Oh et al., 2014; Wu et al., 2014; Gomez-Picos and Eames 2015; Ohba et al., 2015; Tan et al., 2018). On the other hand, negative correlations were predominant between different cell types (IMM vs. MAT). While Sox9 inhibition of MAT genes can explain some of the cross-inhibition between IMM and MAT genes, additional molecular mechanisms, such as epigenetic switches, should be investigated.

In the chondrogenic program presented here, *SOX9* and *RUNX2* were placed at the top of the hierarchy of the GRN (Figure 2D). Molecular genetic experiments have shown that both SOX9 and RUNX2 drive expression of mature chondrocyte genes in limb and head, while only RUNX2 is known to drive osteoblast genes (Eames et al., 2004; Ding et al., 2012; Dy et al., 2012). Several MAT genes included here as part of the core cartilage GRN are also likely part of an osteogenic core GRN. However, a few of these genes (i.e., *COL10A1*) are only expressed in mature chondrocytes, not osteoblasts, of chick and other tetrapods (Bendall et al., 2003; Conen et al., 2009; Leung et al., 2011; Gu et al., 2014). Also, at this early timepoint of collection, only chondrocytes were identified in both the humerus and ceratobranchial, while osteoblasts were restricted to perichondral bone, not within cartilage itself, suggesting no transdifferentiation from chondrocytes to osteoblasts had yet occurred (Figure 1; Zhou et al., 2014; Qin et al., 2020).

Many genes currently associated with cartilage differentiation, and thus the core cartilage GRN, might only be region-specific cartilage competency or morphogenesis factors. Enriched GO terms from humerus or ceratobranchial chondrocytes reflected the embryonic origin of the cells, including limb/forelimb morphogenesis or neural crest-derived processes, respectively. Given region-specific expression, some commonly described cartilage genes, such as ID genes*,* HOX genes*, Tbx5, Sall4*, and *Shox2*, might only serve that purpose in specific regions of the body (Thornemo et al., 1996; Asp et al., 1998; Jung and Tsonis 1998; Yu et al., 2007; Gross et al., 2012; Yamamoto et al., 2019). In principle, establishing regulatory interactions between these transcription factors and the core cartilage GRN might have been crucial to stabilizing cartilage formation in new areas of the vertebrate body during evolution (Figure 6). Interestingly, genes enriched in different embryonic regions were negatively correlated to each other, suggesting cross-inhibition of region-specific transcriptional programs.

In conjunction with region-specific transcription factors, region-specific signalling pathways might activate or stabilize expression of a core cartilage GRN in different regions of the body (Eames and Helms 2004). Both limb and head chondrocytes showed enriched expression of genes involved in BMP, FGF, interleukin (IL), and TGF-B signalling, but the specific upregulated genes were different in each embryonic region. Signalling genes upregulated in the limb included *BMP7, FGF2, FGFRL1, IL13RA2*, and *TGFBR2*, while those upregulated in the head included *BMP5, FGF13, FGF18, FGF23, IL18RAP, IL1RAPL1*, and *TGFBI*. Insulin growth factors (i.e., *IGF2BP2*) were only upregulated in limb chondrocytes, whereas genes involved in EGF signalling (*EGF, EGFL6,* and *EGFR*), PDGF signalling (i.e., *PDGFA, PDGFB, PDGFC*, and *PDGFD*), and VEGF (*VEGFC*) signalling pathways were only upregulated in head chondrocytes.

With respect to clinical applications, the data presented here supports the idea that the origin of cells does not influence the type of cartilage formed. Therefore, if chondrocytes from one location were to be transplanted into a new location in the body, then genes required for these chondrocytes to properly differentiate in that new environment will ultimately be expressed, and cartilage will grow and differentiate in this new location in the body. Indeed, previous work has shown that when nasal chondrocytes are transplanted into an osteoarthritic knee, they can efficiently adapt to this new environment, and successfully contribute to cartilage repair (Pelttari et al., 2020; Acevedo Rua et al., 2021).

In summary, these comparative transcriptomic results demonstrate that a core transcriptional program is expressed during chondrocyte differentiation of the limb and head. Therefore, when cartilage was added to different regions of the vertebrate skeleton, a core GRN might have been co-opted to drive chondrocyte differentiation (Figure 6). While identifying conserved chondrocyte genes is crucial for developing new therapies for cartilage injuries and disorders, expanding transcriptomic comparisons across more clades will provide valuable insights into the evolutionary development of cartilage.

## Materials and Methods

### Embryo Collection and Tissue Processing

All animal procedures were performed according to guidelines approved by the University of Saskatchewan Animal Care and Use Committee. White leghorn chicken eggs were incubated in a humified incubator at a constant temperature of 37°C. Embryos were harvested at Hamburger-Hamilton stage 36 (∼E10; Hamburger and Hamilton 1951). Each embryo was decapitated, and the forelimbs and lower jaws were dissected and either fixed in 4% paraformaldehyde overnight or immediately placed in 1X PBS/DEPC, followed by embedding in OCT (Tissue Tek, Torrance, CA, United States), and immediately flash-frozen using liquid N2 and 2-Methylbutane (isopentane).

### Histology

Chick HH36 embryos were stained with Alcian blue and Alizarin red using an acid-free solution that included MgCl_2_ to differentiate staining, and then cleared in glycerol/KOH as described elsewhere (Eames et al., 2011). Importantly, in our hands, various sources of Alcian blue do not work with the acid-free protocol, but one that does is from Acros Organics (Alcian Blue 8GX). Safranin O/Fast Green staining was performed on 10 μm thick frozen sections of the HH36 chick humeri and ceratobranchial, as described previously (Ferguson et al., 1998). Trichrome staining was performed on 10 μm thick frozen sections of the HH36 chick mandible, as described elsewhere (Ashique et al., 2022).

### Laser Capture Microdissection

LCM was performed on a Laser Microdissection—Molecular Machines & Industries (MMI) CellCut apparatus. Immature and mature chondrocytes were captured from developing chick HH36 humeri (IMM, *n* = 4; MAT, *n* = 5) and ceratobranchial (IMM, *n* = 3; MAT, *n* = 3). At this early stage of development, perichondral bone was evident in both the humerus and ceratobranchial, but no osteoblasts or other cell types were present in the cartilage template, and invasion by the vasculature had not yet occurred. Tissue slices were processed and sequenced individually (not pooled at any stage), and the captured cells were collected onto the inner lid of 0.5 ml MMI IsolationCaps (either Diffuser caps (Prod#50202) or Transparent caps (Prod#50204; MMI Molecular Machines & Industries).

### RNA Isolation and Amplification

RNA was isolated from laser-captured tissue using the ARCTURUS PicoPure RNA Isolation Kit (ThermoFisher Scientific; Cat# KIT0204), according to the manufacturer’s instructions, and DNase treatment was done using RNase-Free DNase (Qiagen; Cat#79254). RNA was then amplified with one round using MessageAmp II aRNA Kit (ThermoFisher Scientific; Cat# AM1751). RNA integrity was evaluated on the observation of a signature eletropherogram pattern (Bioanalyzer).

### Library Preparation and Deep RNA Sequencing

RNA-seq libraries were prepared by the National Research Council (NRC, Saskatoon) using the Illumina TruSeq RNA Sample Prep Kit v2 with the following modification: the protocol was started at the Elute, Prime, Fragment step using 5 µl amplified mRNA [minimum amount was 5–10 ng mRNA as determined using Quant-iT RiboGreen RNA Assay Kit (Invitrogen)]. The quality of each cDNA library was checked on a DNA 1,000 chip using the 2,100 Bioanalyzer (Agilent Technologies Inc.). In average, the sequencing depth was 21-million reads per sample (min. 14-million reads per sample; max. 31- million reads per sample).

### Reads Preprocessing, Mapping, Quantitation, and Primary Analysis of RNA-Seq Data

The paired-end Illumina reads were trimmed using a Java -based tool, Trimmomatic v0.30 (Bolger et al., 2014), and the reads were then mapped to the chicken genome on Ensembl using STAR v 2.5.2 (Dobin et al., 2012). The location of each read was matched to genome annotation using HTSeq-count (Anders et al., 2015). The distribution of average log_2_ expression across three replicates of each tissue produced three bimodal distributions, which were used to set the count thresholds to 142 and 23 for IMM and MAT isolated from the ceratobranchial, and 37 and 58 for IMM and MAT isolated from the humerus. When head and limb data were combined before normalization, thresholds were set to 64 and 35 for IMM and MAT, respectively. Including different sets of samples before normalization affects the number of genes expressed above threshold, because the gene counts will change depending on the exact samples they are normalized against. Differential expression analysis was performed using EdgeR after excluding genes with zero or very low counts (less than three counts for all cell types) across the cell type. Pairwise comparisons between tissues were made with Fisher’s exact test, and a gene was considered differentially expressed if it had an absolute log_2_ fold change greater than 2 (*p* < 0.01). Venn diagrams were constructed using gplots v3.0.1 for isoforms and RNA-seq expression data.

### Principal Component Analysis

To evaluate similarities and differences among the IMM and MAT transcriptomes obtained from limb and head chondrocytes, a principal component analysis (PCA) was performed. PCA was performed on the data using prcomp from the stats library in R to determine if the biological replicates of each cell type separated into distinct groups based on gene expression variance. The 95% confidence ellipses were included using R package factoextra version 1.0.7. The variation in the samples was captured with two components (39% variance explained by PC1 and PC2; Supplementary Figure S1).

### Cluster Analysis

The algorithms from MBCluster.Seq 1.0 package in R were used to cluster the genes from our RNA-seq data (Si et al., 2014). Genes were assigned to 10 clusters based on expression profiles across all IMM and MAT isolated from limb and head (Supplementary Figure S2).

### Validated Chondrocyte GRN

The skeletal cell GRN was constructed using BioTapestry version 7.1.2 (www.BioTapestry.org/) following developer’s protocol (Longabaugh et al., 2005). Regulatory interactions were validated based on published studies including genetic molecular experiments and cis-regulatory analyses performed in bones of mouse, chick, frog, and zebrafish (Ducy et al., 1997; Komori et al., 1997; Lecanda et al., 1997; Zhao et al., 1997; Bridgewater et al., 1998; Drissi et al., 2000; Sekiya et al., 2000; Akiyama et al., 2002; Zhang et al., 2003; Zheng et al., 2003; Akiyama et al., 2004; Bastepe et al., 2004; Stock et al., 2004; Yang et al., 2004; Hong et al., 2005; Magee et al., 2005; Meech et al., 2005; Yagi et al., 2005; Arnold et al., 2007; Holleville et al., 2007; Liu et al., 2007; Yun and Im 2007; Grogan et al., 2008; Higashihori et al., 2008; Vincourt et al., 2008; Teplyuk et al., 2009; Wang et al., 2009; Dao et al., 2010; Fazenda et al., 2010; Higashiyama et al., 2010; Leung et al., 2011; Nagy et al., 2011; Peacock et al., 2011; Gu et al., 2012; Ionescu et al., 2012; McGee-Lawrence et al., 2013; Oh et al., 2014; Liu and Lefebvre 2015; Ohba et al., 2015; Heilig et al., 2016; Takegami et al., 2016; Watanabe et al., 2016; Komori 2017; Yao et al., 2017; Kawane et al., 2018; Komori 2018; Liu et al., 2018; Qin et al., 2018; Tan et al., 2018; Xu et al., 2018; Komori 2019; Kurakazu et al., 2019; Mokuda et al., 2019; Yamashita et al., 2019; Wuelling et al., 2020).

### GO Analysis

DAVID v6.8 (http://david.abcc.ncifcrf.gov/home.jsp) functional annotation analysis was performed on genes expressed above threshold in head and limb. The GO term biological process (BP) in DAVID was used to perform the gene-annotation enrichment analysis.

### Gene Co-Expression Network Analyses

For constructing GCNs, the Pearson correlation between genes was calculated using the TMM normalized gene expression data. Thresholding the edge weights (+/−0.85) was then performed to remove potentially irrelevant edge weights before visualization. All processing and normalization of the RNA-seq counts were performed using the edgeR package using R version 4.0.0. GCNs were visualized in Cytoscape version 3.8.2 and all color coding of edges and nodes was performed using Cytoscape.

## Data Availability

The datasets presented in this study can be found in online repositories. The names of the repository/repositories and accession number(s) can be found below: NCBI’s Gene Expression Omnibus (https://www.ncbi.nlm.nih.gov/geo/) and are accessible through GEO Series accession number GSE186980.
