# Supplementary material for: Limb Mesoderm and Head Ectomesenchyme Both Express a Core Transcriptional Program During Chondrocyte Differentiation
Source: Front Cell Dev Biol. 2022 Jun 17;10:876825. doi: 10.3389/fcell.2022.876825 (PMC9247276; doi:10.3389/fcell.2022.876825)
Supplement: Supplementary file 1 [file DataSheet1.pdf]

# Supplementary material

**Limb mesoderm and head ectomesenchyme both express a core transcriptional program during chondrocyte differentiation**

Patsy Gomez-Picos[1], Katie Ovens[2], and B. Frank Eames[1*]

1 Department of Anatomy, Physiology, and Pharmacology, University of Saskatchewan, Saskatoon, SK, Canada

2 Department of Computer Science, University of Calgary, Calgary, AB, Canada

* corresponding author:

B. Frank Eames

2D01-107 Wiggins Rd

Saskatoon, SK

S7N 5E5

Canada

(306)966-6534

b.frank@usask.ca

eameslab.ca

**Supplementary Figures and tables**

Supplementary Figure S1. PCA analysis emphasized similarities and differences in gene expression. .......... 3

Supplementary Figure S2. Model-based clustering demonstrated that groups of genes in limb and head chondrocytes had discrete categories of expression. .......... 4

Supplementary Figure S3. Cartilage GRN when limb and head data are not combined shows six discrete portions of enriched genes. .......... 5

Supplementary table S1. Upregulated genes in limb IMM compared to limb MAT.......... 6

Supplemental table S2. Upregulated genes in limb MAT compared to limb IMM.......... 12

Supplementary table S3. Upregulated genes in head IMM compared to head MAT .......... 27

Supplemental table S4. Upregulated genes in head MAT compared to head IMM .......... 29

Supplementary table S5. Upregulated genes in combined limb + head IMM compared to limb + head MAT .......... 31

Supplementary table S6. Upregulated genes in combined limb + head MAT compared to limb + head IMM.......... 34

Supplementary table S7. Upregulated genes in limb IMM compared to head IMM.......... 38

Supplementary table S8. Upregulated genes in head IMM compared to limb IMM.......... 43

Supplementary table S9. Upregulated genes in limb IMM compared to head IMM.......... 69

Supplementary table S10. Upregulated genes in limb MAT compared to head MAT.......... 86

## SUPPLEMENTARY FIGURES

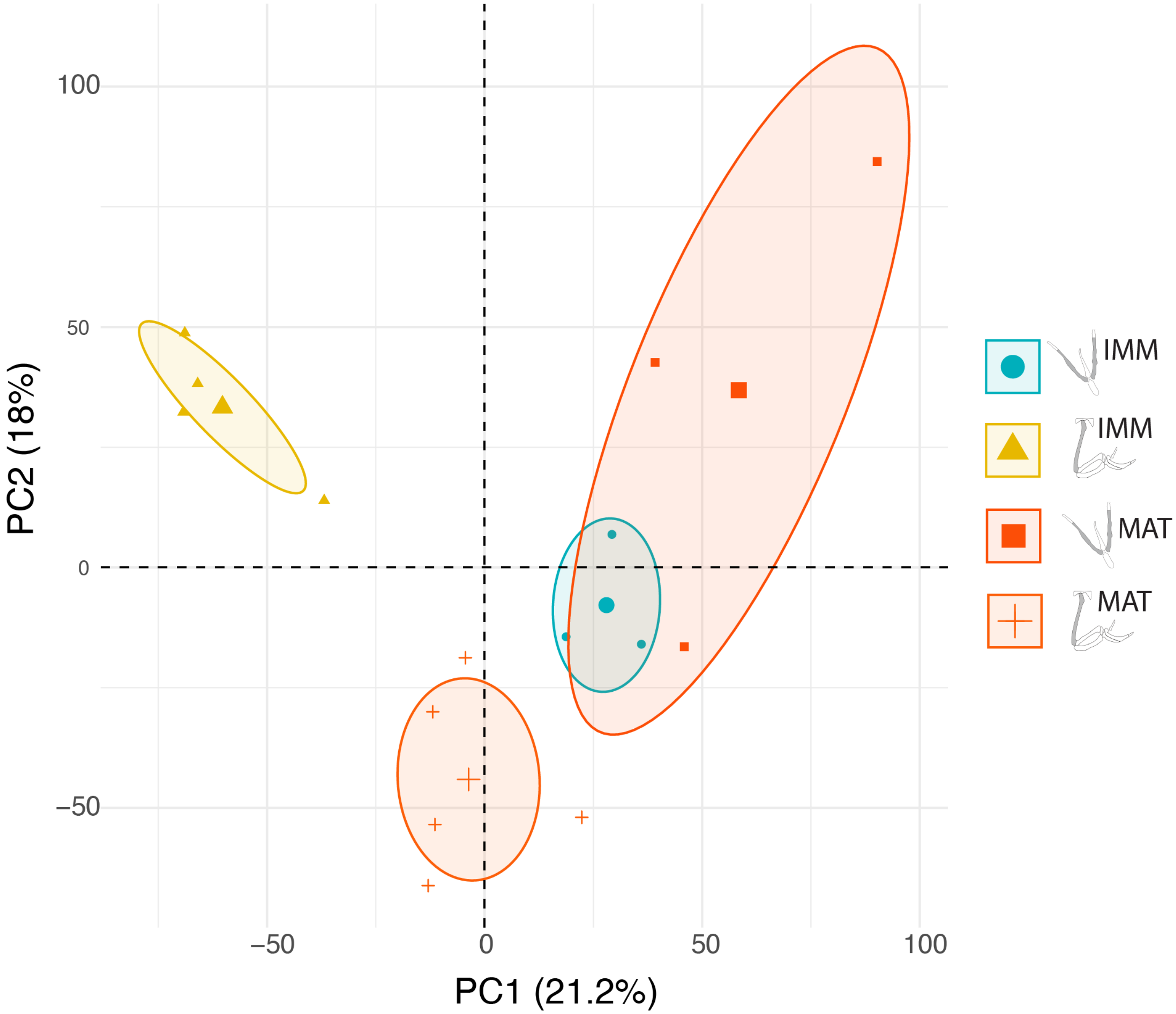


**Supplementary Figure S1. PCA analysis emphasized similarities and differences in gene expression**. The variation in the samples was captured well with two components (39% variance explained by PC1 and PC2). Head IMM and MAT transcriptomes showed the least variation along PC1/PC2 compared to the limb IMM and MAT. The limb IMM and MAT transcriptomes were separated from the head IMM and MAT transcriptomes in PC1/PC2, suggesting overlapping and distinct gene expression patterns among all cell types.

A

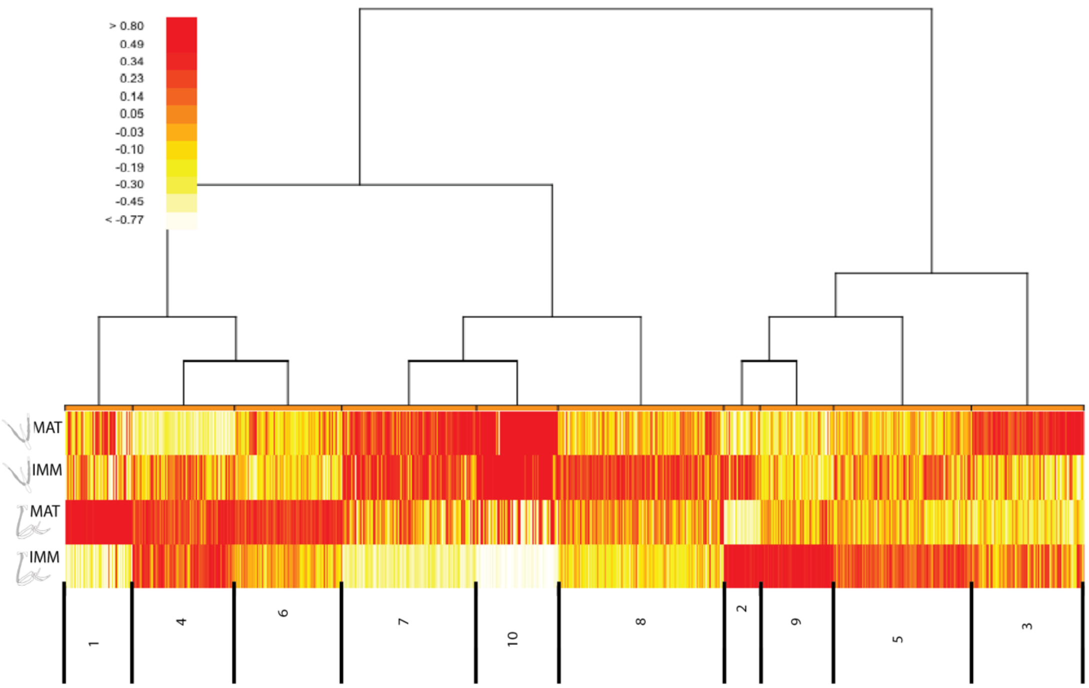


B

| CLUSTER 1 | CLUSTER 2 | CLUSTER 3 | CLUSTER 4 | CLUSTER 5 | CLUSTER 6 | CLUSTER 7 | CLUSTER 8 | CLUSTER 9 | CLUSTER 10 |
|---|---|---|---|---|---|---|---|---|---|
| BMP4 | BMPR1B | GM4076 | ACAN | DDX1 | BCL2 | HHIP | BCL10 | COL11A1 | ADAMTS15 |
| BMP6 | CENPI* | MT-ATP6 | COL2A1 | FOXA2 | CTS3 | MMP2 | BMPR2 | COL9A2 | ADAMTS18 |
| COL10A1 | CLEC18A* | MT-CO1 | COL9A1 | GLG1 | CTS6 | NHEJ1 | CHPF | HOXA10 | ADAMTS5 |
| DLX5 | CSF1* | MT-CO2 | COL9A1 | HOXA11 | CTS7 | PTH1R | CHPF2 | HOXA4 | ARSI |
| FAM20C* | CTHRC1* | MT-CO3 | COL9A3 | LNPK | CTS8 | PTHLH | CHST11 | HOXA5 | FN1 |
| FGF9 | FBLN7* | MT-CYTB | COL9A3 | MATN4 | CTSJ | TGFBR3* | DSE | HOXA6 | GALNS |
| INSRR* | GLI2 | MT-ND1 | COMP | SOX6 | CTSL | TWIST1 | SOX10 | HOXA9 | GATA2 |
| IRX6* | GLI3 | MT-ND2 | | SOX9 | CTSLL3 | | UGDH | HOXD12 | HES5 |
| MAP3K5* | GPR37L1* | MT-ND3 | | TRPS1 | CTSM | | | HOXD4 | HHEX |
| MMP13 | HOXD10 | MT-ND4 | | | CTSQ | | | HOXD8 | IBSP |
| PTGS2 | HOXD11 | MT-ND4L | | | CTSR | | | UNC5C* | IFITM5 |
| SATB1 | MATN3 | MT-ND5 | | | MATN1 | | | | MEF2C |
| SLC6A2* | MMP16* | MT-ND6 | | | MMP23 | | | | MYBPC1 |
| SPARC | PTN* | PRRX1 | | | RUNX2 | | | | MYBPC3 |
| SPP1 | RUNX3 | SIX1 | | | SP1 | | | | MYO6 |
| TCF7L2* | TBX5 | SIX4 | | | | | | | PAX7 |
| TEK | TNC* | SOX5 | | | | | | | PRRX2 |
| TGFBR2* | | | | | | | | | VDR |
| VEGFA | | | | | | | | | VEGFC |
| WNT5B | | | | | | | | | WNT11 |

**Supplementary Figure S2. Model-based clustering demonstrated that groups of genes in limb and head chondrocytes had discrete categories of expression. (A)** Heatmap showed the distribution of gene expression change among cell types compared to the average expression across all three cell types. . Cluster 4 and 5 show enriched expression in chondrocytes of limb (IMM and MAT) and head (IMM) including classic cartilage differentiation genes, such as *SOX9, SOX6, ACAN*, and *COL2A1*. Cluster 1 shows enriched expression in limb MAT including classic maturation genes such as *COL10A1, MMP13*, and *SPP1*, whereas cluster 10 shows enriched expression of MAT genes in the head including *IBSP* and *MEF2C*. Cluster 10 also showed enriched expression of cranial neural crest development genes in both head IMM and MAT including *FN1, GATA2, PAX7*, and MYB genes. Cluster 3 showed enriched expression in head MAT including typical cartilage genes such as *SOX5* and classic cranial neural crest markers *SIX1, SIX4*, and *PRRX1*. Clusters 2 and 9 show enriched expression in limb IMM, and it includes several limb differentiation markers, such as *TBX5* and HOX genes and classic cartilage genes including *COL9A2* and *COL11A1*. **(B)** Examples of representative candidate genes located included in each cluster. Novel putative cartilage genes are indicated with an asterisk next to the gene name.

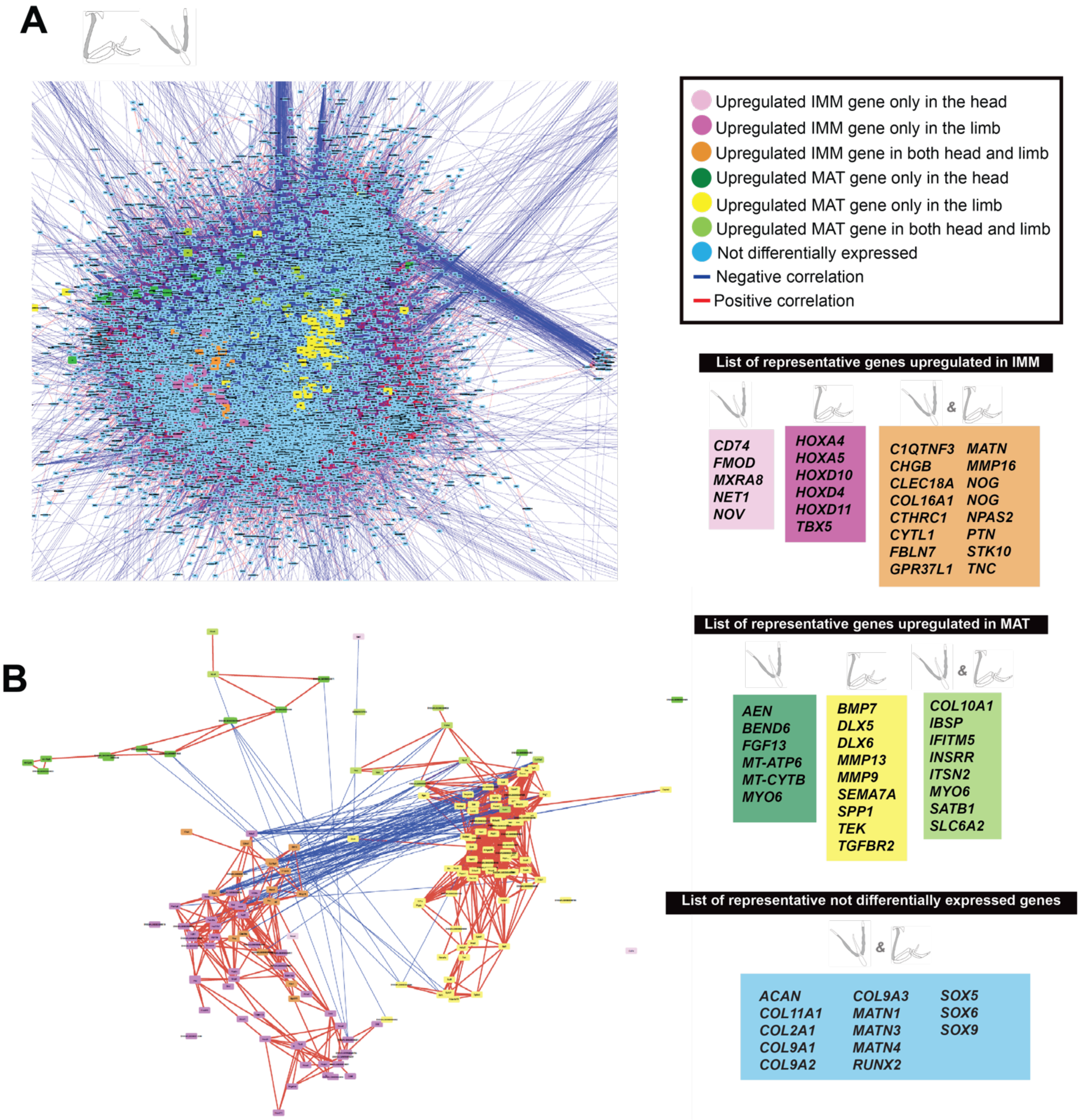


**Supplementary Figure S3. Cartilage GRN when limb and head data are not combined shows six discrete portions of enriched genes.** In this case, the humerus and the ceratobranchial were considered as separate skeletal elements before normalization. Most positive correlations included in this estimated GRN were between genes enriched within the same cell type (i.e. IMM or MAT), but a few negative correlations are also observed between genes from the same cell type. Most negative interactions occur between genes upregulated in different cell types.

# SUPPLEMENTARY TABLES

**Supplementary table S1.** Upregulated genes in limb IMM compared to limb MAT

| Gene names | logFC | logCPM | PValue | p_adjusted |
|---|---|---|---|---|
| *NRIP3* | 9.84910465 | 6.20971531 | 1.47E-33 | 2.92E-29 |
| *CLEC18A* | 9.42887558 | 2.79931748 | 7.29E-15 | 4.52E-12 |
| *ENSGALG00000030861* | 8.36791161 | 0.65830726 | 8.72E-09 | 9.88E-07 |
| *ENSGALG00000014501* | 7.19735065 | 8.69605087 | 1.30E-12 | 4.38E-10 |
| *NPAS2* | 5.9490581 | 4.02476233 | 1.53E-13 | 6.61E-11 |
| *ENSGALG00000034381* | 5.93488166 | 2.48512578 | 7.03E-05 | 0.0016486 |
| *ENSGALG00000029942* | 5.90692282 | 2.32873052 | 1.64E-07 | 1.17E-05 |
| *PTN* | 5.69924071 | 10.8053697 | 4.05E-19 | 5.73E-16 |
| *SCUBE2* | 5.64293659 | 6.11040771 | 1.54E-12 | 5.01E-10 |
| *BARX1* | 5.50074677 | 4.06507771 | 1.82E-15 | 1.34E-12 |
| *PRSS56* | 5.43466501 | 0.70158565 | 6.95E-06 | 0.00025131 |
| *MATN3* | 5.36327529 | 4.92478517 | 2.85E-07 | 1.79E-05 |
| *ENSGALG00000035945* | 5.36213197 | 1.38777337 | 1.21E-08 | 1.32E-06 |
| *RALYL* | 5.16783803 | 1.69949636 | 6.64E-05 | 0.00158334 |
| *ENSGALG00000029601* | 5.13585682 | 6.585364 | 1.48E-16 | 1.40E-13 |
| *PLEKHG5* | 5.10341206 | 4.87297573 | 1.18E-13 | 5.31E-11 |
| *FHAD1* | 5.09894118 | 1.45904646 | 1.63E-06 | 7.69E-05 |
| *ENSGALG00000029504* | 5.07365161 | -0.3044606 | 0.00065203 | 0.0095884 |
| *ALOX5AP* | 5.03812063 | 3.7450949 | 2.79E-09 | 3.57E-07 |
| *CYTL1* | 4.75161582 | 9.2384729 | 1.58E-10 | 2.91E-08 |
| *ASB9* | 4.70667278 | 6.88729807 | 9.29E-27 | 3.68E-23 |
| *COL16A1* | 4.64810426 | 4.95799881 | 2.04E-13 | 8.60E-11 |
| *ENSGALG00000010002* | 4.62110469 | 1.92430669 | 2.08E-05 | 0.00061974 |
| *ENSGALG00000005790* | 4.57172137 | 4.80785658 | 2.21E-12 | 6.84E-10 |
| *RLBP1* | 4.56669763 | 6.17913212 | 1.23E-09 | 1.72E-07 |
| *SLC6A7* | 4.53040519 | 1.65127589 | 1.03E-07 | 7.91E-06 |
| *TNC* | 4.43357534 | 8.13431872 | 5.09E-12 | 1.51E-09 |
| *ENSGALG00000038134* | 4.39452973 | 3.03349764 | 3.99E-12 | 1.20E-09 |
| *ENSGALG00000010003* | 4.33132887 | 4.09492788 | 6.25E-07 | 3.43E-05 |
| *DPYSL4* | 4.29111655 | 1.4706522 | 2.46E-06 | 0.00010888 |
| *ENSGALG00000025241* | 4.28851491 | 3.3366274 | 5.25E-08 | 4.55E-06 |
| *ADCY2* | 4.23572125 | 5.34446731 | 8.25E-10 | 1.24E-07 |
| *ENSGALG00000032611* | 4.22714981 | 4.35516956 | 3.09E-14 | 1.61E-11 |
| *TBX5* | 4.2251969 | 5.79417709 | 2.31E-08 | 2.23E-06 |

| | | | | |
|---|---|---|---|---|
| *FAM84A* | 4.13778119 | 4.35599713 | 1.95E-10 | 3.48E-08 |
| *HOXD11* | 4.12784105 | 6.29643925 | 0.00018111 | 0.00354408 |
| *CSPG5* | 4.08943575 | 2.04066332 | 4.61E-06 | 0.00018222 |
| *ENSGALG00000046192* | 4.08208038 | 1.86151826 | 7.43E-06 | 0.00026285 |
| *SLC13A3* | 4.05678017 | 3.83610737 | 4.47E-08 | 4.05E-06 |
| *ENSGALG00000043332* | 3.95212443 | 4.82683458 | 8.13E-12 | 2.27E-09 |
| *DDR2* | 3.94195527 | 4.91503507 | 1.29E-10 | 2.42E-08 |
| *D630039A03RIK* | 3.93605507 | 0.48459502 | 0.00028639 | 0.00510999 |
| *TRABD2B* | 3.93248794 | 3.22021558 | 8.47E-09 | 9.67E-07 |
| *ENSGALG00000033971* | 3.90081396 | 2.87157681 | 2.75E-07 | 1.77E-05 |
| *RIMS2* | 3.86557924 | 4.51517881 | 1.34E-07 | 9.97E-06 |
| *SORBS2* | 3.82839298 | 4.98082182 | 5.45E-11 | 1.19E-08 |
| *TSPAN15* | 3.82484132 | 4.90816523 | 8.49E-09 | 9.67E-07 |
| *CSF1* | 3.77178502 | 2.05107137 | 1.40E-09 | 1.94E-07 |
| *ARHGEF28* | 3.7693748 | 2.58095843 | 1.92E-05 | 0.00057946 |
| *ENSGALG00000034648* | 3.74319786 | 4.12070336 | 7.01E-11 | 1.49E-08 |
| *ENSGALG00000044619* | 3.72299019 | 3.12934644 | 1.19E-07 | 9.07E-06 |
| *BARX2* | 3.72014701 | 4.35026631 | 2.14E-07 | 1.43E-05 |
| *P4HA3* | 3.71592632 | 7.25818665 | 2.51E-15 | 1.78E-12 |
| *GPR37L1* | 3.65838425 | 3.43002107 | 2.73E-05 | 0.00077016 |
| *NOG* | 3.62375979 | 4.94344596 | 4.34E-08 | 3.97E-06 |
| *SLC4A7* | 3.61458886 | 8.46369617 | 1.73E-20 | 3.13E-17 |
| *KANK3* | 3.6074883 | 3.1812829 | 5.28E-07 | 2.95E-05 |
| *ENSGALG00000035027* | 3.5740603 | 7.74416069 | 0.0002583 | 0.00473655 |
| *E2F1* | 3.56327996 | 5.86873178 | 6.13E-18 | 6.76E-15 |
| *PATL2* | 3.53583352 | 1.62662499 | 2.69E-07 | 1.74E-05 |
| *ZFP703* | 3.50432665 | 0.29441788 | 0.00018196 | 0.00355361 |
| *ENSGALG00000006658* | 3.4861618 | 4.96370419 | 6.14E-14 | 2.90E-11 |
| *COL13A1* | 3.47035303 | 4.56249204 | 7.75E-06 | 0.00027382 |
| *ENSGALG00000023953* | 3.4521106 | 1.31910728 | 0.00011739 | 0.0024942 |
| *NCAPG2* | 3.44018105 | 5.02321129 | 1.47E-06 | 7.06E-05 |
| *RASL11B* | 3.41363601 | 6.1841366 | 3.72E-07 | 2.23E-05 |
| *EFHD2* | 3.39876796 | 5.07237367 | 2.24E-14 | 1.22E-11 |
| *AJAP1* | 3.38502893 | 2.5466056 | 0.00029516 | 0.00521017 |
| *GAS2L3* | 3.34805911 | 3.39753308 | 1.52E-05 | 0.0004772 |
| *CACNA1C* | 3.34610322 | 4.38247265 | 7.19E-07 | 3.84E-05 |
| *KIF26B* | 3.34266213 | 4.07329467 | 4.95E-06 | 0.00019158 |
| *CDT1* | 3.32535827 | 2.67993911 | 1.48E-06 | 7.07E-05 |
| *PRODH* | 3.31332376 | 4.93913398 | 6.91E-12 | 1.98E-09 |
| *HSD17B7* | 3.3125965 | 6.10234099 | 7.07E-12 | 2.00E-09 |

| | | | | |
|---|---|---|---|---|
| *ENSGALG00000023193* | 3.30037803 | 5.06135057 | 3.81E-14 | 1.89E-11 |
| *HOXD10* | 3.29469324 | 3.39852972 | 3.96E-06 | 0.00016046 |
| *NLGN3* | 3.28583538 | 2.28267302 | 5.38E-08 | 4.58E-06 |
| *ENSGALG00000003446* | 3.27739272 | 5.4177482 | 1.29E-12 | 4.38E-10 |
| *CIT* | 3.274061 | 4.35604136 | 1.17E-10 | 2.23E-08 |
| *ENSGALG00000036517* | 3.26196635 | 0.95668937 | 0.00015976 | 0.00320857 |
| *B4GALNT4* | 3.23877445 | 2.4043677 | 0.00011947 | 0.00252474 |
| *ENSGALG00000010741* | 3.20766351 | 5.96723675 | 2.29E-08 | 2.23E-06 |
| *GM7173* | 3.16389294 | 3.36667155 | 2.05E-05 | 0.00061401 |
| *ENSGALG00000016636* | 3.15959121 | 7.64224957 | 1.41E-08 | 1.50E-06 |
| *ENSGALG00000029239* | 3.15139327 | 2.67390321 | 0.00045733 | 0.00726859 |
| *NPR1* | 3.13971785 | 2.66106296 | 0.00030476 | 0.00532738 |
| *ENSGALG00000030769* | 3.13503865 | 2.22134762 | 3.94E-06 | 0.00015991 |
| *ENSGALG00000003161* | 3.13148167 | 1.4973566 | 9.50E-06 | 0.0003254 |
| *ENSGALG00000032990* | 3.12781038 | 4.42187248 | 1.51E-07 | 1.09E-05 |
| *ENSGALG00000018331* | 3.12497553 | 2.45078073 | 3.95E-05 | 0.00104274 |
| *SLC35D1* | 3.11986509 | 1.79925706 | 0.00025655 | 0.00470896 |
| *SRPX* | 3.1155544 | 7.42784633 | 4.65E-10 | 7.56E-08 |
| *ENSGALG00000039384* | 3.10501673 | 4.654101 | 4.29E-13 | 1.67E-10 |
| *SRGAP3* | 3.10199357 | 2.40333226 | 1.57E-05 | 0.0004878 |
| *WDR76* | 3.08892027 | 3.69409187 | 2.30E-08 | 2.23E-06 |
| *UBE2C* | 3.08446966 | 5.39170538 | 1.25E-06 | 6.09E-05 |
| *ENSGALG00000036572* | 3.07938372 | 2.80141357 | 3.30E-07 | 2.04E-05 |
| *ZGRF1* | 3.0672447 | 4.62211194 | 1.10E-11 | 2.94E-09 |
| *KCNA4* | 3.0494356 | 3.6552458 | 6.52E-05 | 0.00156007 |
| *ENSGALG00000035579* | 3.04639356 | 4.71431092 | 4.45E-07 | 2.58E-05 |
| *BRF2* | 3.0420738 | 6.73636081 | 2.11E-08 | 2.11E-06 |
| *ENSGALG00000040348* | 3.01532204 | 7.84929578 | 2.71E-11 | 6.32E-09 |
| *ENSGALG00000036011* | 3.01195574 | 2.00725346 | 0.00014559 | 0.00297536 |
| *MCM5* | 3.00992462 | 5.89730329 | 1.11E-15 | 8.45E-13 |
| *TRAIP* | 3.00513006 | 4.89561707 | 1.09E-08 | 1.20E-06 |
| *SHMT1* | 3.00129972 | 1.95897404 | 5.82E-05 | 0.00141119 |
| *SHROOM4* | 2.97790666 | 5.3775435 | 7.70E-11 | 1.61E-08 |
| *ENSGALG00000032444* | 2.96732791 | 6.57531938 | 8.89E-07 | 4.58E-05 |
| *TTC34* | 2.95572411 | 2.12460785 | 0.00019059 | 0.0037077 |
| *BRCA1* | 2.9528571 | 5.98971807 | 5.13E-07 | 2.91E-05 |
| *ESPL1* | 2.94873668 | 3.79251131 | 6.99E-08 | 5.68E-06 |
| *ENSGALG00000034155* | 2.94583163 | 2.83152018 | 1.21E-07 | 9.16E-06 |
| *ZFP651* | 2.94538646 | 0.9072331 | 4.41E-05 | 0.00113388 |
| *NEK2* | 2.92531058 | 4.93398474 | 3.87E-08 | 3.59E-06 |

| | | | | |
|---|---|---|---|---|
| *CALCA* | 2.92274995 | 3.60395446 | 6.60E-09 | 7.79E-07 |
| *DNA2* | 2.90559388 | 4.31959797 | 7.75E-07 | 4.07E-05 |
| *FANCD2* | 2.89657021 | 3.31653336 | 1.16E-06 | 5.71E-05 |
| *ENSGALG00000038920* | 2.87936233 | 2.28043338 | 0.00061615 | 0.00919034 |
| *CCNB3* | 2.8695263 | 4.95528464 | 5.93E-06 | 0.0002198 |
| *CDCA7* | 2.86921118 | 6.90454524 | 1.20E-08 | 1.32E-06 |
| *SCN8A* | 2.8613839 | 4.75999829 | 4.44E-11 | 9.85E-09 |
| *ORC1* | 2.85877394 | 4.19265948 | 5.22E-07 | 2.93E-05 |
| *GLI2* | 2.82327095 | 5.90499452 | 2.44E-05 | 0.00070643 |
| *SDK2* | 2.81712149 | 4.02253901 | 3.85E-07 | 2.28E-05 |
| *B3GNT7* | 2.81420539 | 6.32739701 | 1.96E-05 | 0.00058935 |
| *THBS1* | 2.80247009 | 9.16481638 | 1.61E-07 | 1.15E-05 |
| *SMOC2* | 2.78782417 | 4.07253443 | 8.92E-05 | 0.00198705 |
| *FBLN7* | 2.78660966 | 5.26082245 | 3.06E-07 | 1.91E-05 |
| *SLC6A9* | 2.78247778 | 4.54060864 | 9.76E-07 | 4.97E-05 |
| *ENSGALG00000042543* | 2.7651652 | 2.70810452 | 0.00035985 | 0.00610212 |
| *ENSGALG00000034631* | 2.76293616 | 3.99065767 | 1.43E-07 | 1.05E-05 |
| *ENSGALG00000000407* | 2.75182807 | 3.82480274 | 4.56E-05 | 0.0011637 |
| *DIAPH3* | 2.73513086 | 5.31570792 | 1.91E-06 | 8.70E-05 |
| *ENSGALG00000012836* | 2.73138077 | 5.3973316 | 1.09E-11 | 2.94E-09 |
| *ERBB3* | 2.6889763 | 4.99388914 | 6.37E-07 | 3.47E-05 |
| *KIF2C* | 2.6864714 | 5.527067 | 9.88E-06 | 0.00033235 |
| *PICALM* | 2.68133417 | 3.65661139 | 2.63E-05 | 0.00075006 |
| *MCM2* | 2.67930322 | 6.38051259 | 7.15E-08 | 5.78E-06 |
| *ENSGALG00000030673* | 2.67435835 | 4.96772335 | 5.18E-05 | 0.00127995 |
| *ZFPM1* | 2.6710559 | 3.33438481 | 8.22E-07 | 4.31E-05 |
| *CDC6* | 2.65662232 | 2.12769704 | 5.50E-05 | 0.00135102 |
| *ENSGALG00000006958* | 2.63959766 | 6.90260747 | 7.07E-05 | 0.00165458 |
| *GINS1* | 2.63677009 | 6.32831954 | 1.37E-11 | 3.57E-09 |
| *ASPM* | 2.63542834 | 7.20547576 | 1.81E-05 | 0.00055552 |
| *TK1* | 2.62906441 | 6.37123357 | 9.89E-06 | 0.00033235 |
| *KHDRBS2* | 2.6245146 | 3.09839617 | 0.00033791 | 0.00580957 |
| *OIT3* | 2.62251758 | 3.33125069 | 0.00024806 | 0.00458268 |
| *KNL1* | 2.60692761 | 3.68472436 | 3.48E-05 | 0.00093882 |
| *CDCA3* | 2.60361709 | 5.87592993 | 7.28E-07 | 3.88E-05 |
| *CACNG5* | 2.58945448 | 2.75630177 | 4.37E-05 | 0.00113093 |
| *UHRF1* | 2.58500964 | 7.92780473 | 1.85E-08 | 1.90E-06 |
| *PHLDB1* | 2.56633522 | 2.5731047 | 0.00031928 | 0.00552764 |
| *CAB39L* | 2.55552303 | 7.54227373 | 4.41E-07 | 2.56E-05 |
| *EPB41L4B* | 2.55037445 | 4.14005267 | 3.76E-07 | 2.25E-05 |

| | | | | |
|---|---|---|---|---|
| *CCDC102A* | 2.54228108 | 2.76113051 | 4.17E-05 | 0.00108394 |
| *TMEM266* | 2.53076689 | 4.22598846 | 2.27E-06 | 0.00010125 |
| *ECD* | 2.51299341 | 7.41771612 | 1.97E-09 | 2.60E-07 |
| *ATP10A* | 2.50669344 | 5.02000656 | 3.32E-05 | 0.00090585 |
| *ROPN1* | 2.49345 | 1.34352781 | 0.0004893 | 0.00764935 |
| *ENSGALG00000038194* | 2.49238271 | 6.23794517 | 6.05E-07 | 3.34E-05 |
| *CHAF1B* | 2.4873105 | 5.21999309 | 7.57E-07 | 4.00E-05 |
| *FANCA* | 2.48680323 | 3.16795043 | 1.14E-05 | 0.00037651 |
| *OSBP* | 2.48446744 | 1.35692596 | 0.00035021 | 0.00597429 |
| *TOP2A* | 2.48280716 | 7.4295174 | 3.58E-06 | 0.00014886 |
| *CHGB* | 2.47296873 | 5.95058759 | 7.04E-06 | 0.00025376 |
| *BMPR1B* | 2.47247605 | 4.84985954 | 6.25E-05 | 0.00150377 |
| *TRIM71* | 2.45549368 | 5.48539435 | 7.73E-07 | 4.07E-05 |
| *WEE1* | 2.44906455 | 6.90195651 | 1.22E-09 | 1.72E-07 |
| *CENPI* | 2.44267804 | 4.98841095 | 6.19E-06 | 0.00022692 |
| *MAP2* | 2.44193351 | 3.45493008 | 0.0001522 | 0.00309599 |
| *CUTAL* | 2.43846612 | 4.67564726 | 6.80E-05 | 0.00161551 |
| *TENM2* | 2.43612246 | 4.31704147 | 6.56E-05 | 0.00156613 |
| *ENSGALG00000038135* | 2.43240695 | 3.64397719 | 0.00010538 | 0.00228302 |
| *RAD54L* | 2.42786557 | 4.11163283 | 6.30E-07 | 3.45E-05 |
| *GRIK1* | 2.41834828 | 3.32658524 | 1.60E-05 | 0.00049606 |
| *SIX4* | 2.41532734 | 4.52529164 | 0.00019363 | 0.00375578 |
| *EME1* | 2.41355468 | 4.32034896 | 1.94E-08 | 1.97E-06 |
| *CREB3L3* | 2.41099072 | 2.18085985 | 0.00031264 | 0.00543168 |
| *KIF14* | 2.40876402 | 3.05089924 | 0.00037387 | 0.00630612 |
| *ENSGALG00000026301* | 2.39588273 | 2.62130944 | 0.0002873 | 0.00511274 |
| *MAB21L1* | 2.3913279 | 6.02243256 | 1.89E-09 | 2.57E-07 |
| *WWC2* | 2.38384529 | 3.94960582 | 8.27E-09 | 9.53E-07 |
| *UNC13C* | 2.38365371 | 4.00070411 | 0.00043308 | 0.00698204 |
| *ADCY5* | 2.3804116 | 2.94012086 | 0.00023255 | 0.00434474 |
| *ENSGALG00000041603* | 2.37746762 | 8.43957898 | 2.41E-05 | 0.00070171 |
| *MAB21L2* | 2.37410931 | 4.80494166 | 0.00014942 | 0.00304738 |
| *ENSGALG00000021285* | 2.36887777 | 4.89480001 | 4.22E-07 | 2.47E-05 |
| *GHRL* | 2.36868778 | 4.93491298 | 9.13E-06 | 0.00031463 |
| *ENSGALG00000045842* | 2.36867672 | 4.26157331 | 1.03E-06 | 5.19E-05 |
| *CEP55* | 2.36475456 | 4.28635978 | 9.14E-10 | 1.35E-07 |
| *BLM* | 2.35081695 | 3.86347882 | 5.85E-06 | 0.00021835 |
| *CTHRC1* | 2.35009258 | 8.04577801 | 4.10E-06 | 0.00016522 |
| *SLC35B4* | 2.34478626 | 4.9651872 | 8.43E-06 | 0.00029372 |
| *LSS* | 2.34018104 | 6.93169376 | 8.95E-06 | 0.00030897 |

| | | | | |
|---|---|---|---|---|
| *MYCBPAP* | 2.33602424 | 2.31906462 | 3.58E-06 | 0.00014886 |
| *ENSGALG00000044031* | 2.32499372 | 1.54004444 | 0.00029117 | 0.00516273 |
| *ENSGALG00000035814* | 2.32140676 | 3.1425413 | 3.91E-06 | 0.00015907 |
| *CLSPN* | 2.31667621 | 4.40131999 | 8.20E-10 | 1.24E-07 |
| *PRPH* | 2.31315303 | 1.63446693 | 0.00012992 | 0.0027137 |
| *MMP16* | 2.31044849 | 6.31332994 | 9.06E-08 | 7.15E-06 |
| *GDAP1L1* | 2.30445792 | 3.18767477 | 0.00047674 | 0.00749295 |
| *NCAPG* | 2.30166874 | 5.15802095 | 1.90E-05 | 0.00057513 |
| *NPTX2* | 2.3013104 | 4.06650094 | 1.54E-05 | 0.00048249 |
| *PPRC1* | 2.29010439 | 4.26531867 | 1.31E-07 | 9.82E-06 |
| *STK10* | 2.28964821 | 5.51577034 | 3.67E-05 | 0.00098165 |
| *LSAMP* | 2.28735929 | 5.3636784 | 4.78E-05 | 0.00120641 |
| *ST7* | 2.2778975 | 4.77018201 | 2.37E-07 | 1.56E-05 |
| *MKI67* | 2.27634001 | 5.97759661 | 0.00028689 | 0.00511274 |
| *PSMC3IP* | 2.27213542 | 5.65625281 | 4.22E-09 | 5.19E-07 |
| *ENSGALG00000035675* | 2.26883071 | 2.0100281 | 0.0001022 | 0.00222138 |
| *SDC3* | 2.24788043 | 6.37872889 | 5.13E-05 | 0.00127525 |
| *CCNF* | 2.24528085 | 4.10973761 | 6.60E-06 | 0.00024062 |
| *ENSGALG00000042782* | 2.23699315 | 3.54184038 | 8.48E-05 | 0.00190713 |
| *SKA3* | 2.23020185 | 5.19817499 | 1.15E-06 | 5.66E-05 |
| *CENPL* | 2.22404773 | 4.60965483 | 3.46E-05 | 0.00093696 |
| *PBK* | 2.22234644 | 5.67704219 | 7.40E-06 | 0.00026285 |
| *ENSGALG00000032021* | 2.21964874 | 4.15578411 | 4.87E-07 | 2.80E-05 |
| *HOXA4* | 2.20882836 | 6.52569668 | 1.85E-06 | 8.51E-05 |
| *ENSGALG00000039528* | 2.20425963 | 3.99800385 | 1.25E-05 | 0.00040661 |
| *CCDC15* | 2.20340997 | 4.98623459 | 8.79E-11 | 1.80E-08 |
| *ENSGALG00000036783* | 2.20211128 | 5.44189651 | 4.38E-09 | 5.36E-07 |
| *ENSGALG00000012085* | 2.1994831 | 4.24422109 | 0.00013103 | 0.00273126 |
| *TMEM51* | 2.19928068 | 3.70152281 | 2.70E-05 | 0.00076363 |
| *DAB1* | 2.19389384 | 5.58808387 | 1.86E-06 | 8.51E-05 |
| *FDFT1* | 2.19085388 | 7.84607344 | 4.68E-06 | 0.00018326 |
| *TMEM173* | 2.18941214 | 2.67350046 | 0.00027849 | 0.00502317 |
| *THBS4* | 2.17732749 | 6.63838208 | 6.84E-05 | 0.00162268 |
| *GINS2* | 2.16649959 | 3.01878774 | 0.00018106 | 0.00354408 |
| *ENSGALG00000037945* | 2.16463862 | 4.19141547 | 9.12E-05 | 0.00201758 |
| *CRB2* | 2.16138036 | 4.10961097 | 3.61E-07 | 2.19E-05 |
| *PFKL* | 2.15561308 | 4.4620547 | 9.76E-06 | 0.0003301 |
| *GLI3* | 2.14608602 | 5.65046347 | 1.37E-06 | 6.59E-05 |
| *MB21D1* | 2.14549871 | 4.30874916 | 6.26E-08 | 5.19E-06 |
| *DTL* | 2.14215568 | 3.19223307 | 0.00010369 | 0.00225123 |

| | | | | |
|---|---|---|---|---|
| *DUSP14* | 2.13808108 | 5.41119634 | 1.17E-06 | 5.73E-05 |
| *ENSGALG00000002461* | 2.13729561 | 3.7868673 | 0.0002159 | 0.0040838 |
| *RAI2* | 2.13635845 | 4.52577151 | 2.22E-08 | 2.20E-06 |
| *HOXD4* | 2.12761415 | 4.78052352 | 9.18E-08 | 7.16E-06 |
| *NCAPD2* | 2.12588244 | 5.55942586 | 1.43E-10 | 2.64E-08 |
| *PER3* | 2.12484301 | 5.38296095 | 8.62E-08 | 6.86E-06 |
| *SLC35F2* | 2.12361707 | 4.47229756 | 5.09E-05 | 0.00126892 |
| *ASAP1* | 2.11851184 | 5.22051608 | 3.63E-06 | 0.00015015 |
| *CHAF1A* | 2.09732176 | 6.86119664 | 2.64E-09 | 3.42E-07 |
| *MCM3* | 2.08968747 | 5.53897912 | 1.30E-07 | 9.79E-06 |
| *ADGRG6* | 2.08940442 | 3.48440418 | 0.00011562 | 0.00246715 |
| *PHGDH* | 2.08390722 | 6.11607648 | 3.44E-06 | 0.00014377 |
| *PASK* | 2.07436474 | 2.99704026 | 0.00023957 | 0.0044508 |
| *VCPKMT* | 2.0682207 | 5.26617168 | 4.36E-07 | 2.54E-05 |
| *ENSGALG00000041219* | 2.06660103 | 4.52111507 | 7.41E-06 | 0.00026285 |
| *MDK* | 2.06470266 | 8.03052019 | 0.00037188 | 0.00627919 |
| *FAM179B* | 2.06169432 | 4.06603387 | 5.93E-06 | 0.0002198 |
| *ENSGALG00000045764* | 2.05482279 | 2.85825055 | 0.00037828 | 0.00634943 |
| *NUSAP1* | 2.04345255 | 5.36085206 | 0.00013768 | 0.00283412 |
| *PRIM2* | 2.03932703 | 5.438685 | 2.83E-07 | 1.79E-05 |
| *WWP2* | 2.03634166 | 10.2453678 | 0.00054148 | 0.0082694 |
| *ENSGALG00000039611* | 2.03156209 | 2.48714336 | 0.00067799 | 0.00987495 |
| *BUB1B* | 2.03067243 | 6.62286286 | 2.54E-06 | 0.00011185 |
| *HOXA5* | 2.02972171 | 7.67435374 | 8.73E-06 | 0.000303 |
| *CDC20* | 2.01803423 | 5.79872284 | 1.84E-05 | 0.00056103 |
| *DMD* | 2.01800346 | 7.27348132 | 1.85E-07 | 1.30E-05 |
| *SYTL4* | 2.01285967 | 3.32690893 | 0.00028226 | 0.00507283 |
| *TOP3B* | 2.00880697 | 6.49812369 | 3.37E-09 | 4.28E-07 |
| *ZCCHC17* | 2.00150978 | 7.12800921 | 3.63E-08 | 3.41E-06 |

**Supplemental table S2.** Upregulated genes in limb MAT compared to limb IMM

| Gene names | logFC | logCPM | PValue | p_adjusted |
|---|---|---|---|---|
| *CRHBP* | 11.67573 | 3.79523438 | 1.35E-07 | 1.00E-05 |
| *COL10A1* | 11.4947613 | 6.12798575 | 1.19E-18 | 1.57E-15 |
| *MMP13* | 11.0104141 | 10.1222498 | 1.87E-24 | 4.64E-21 |
| *SPP1* | 10.5899376 | 11.9902669 | 1.79E-23 | 3.95E-20 |
| *ENSGALG00000046293* | 10.0739702 | 2.60608178 | 4.34E-14 | 2.10E-11 |
| *IRX1* | 10.0247699 | 2.29139527 | 6.97E-05 | 0.00164242 |

| | | | | |
|---|---|---|---|---|
| *IRX6* | 9.88538157 | 5.03032033 | 5.63E-27 | 2.79E-23 |
| *SST* | 9.83512153 | 2.06401919 | 0.00034578 | 0.00592936 |
| *ENSGALG00000006453* | 9.42905024 | 7.9604235 | 1.13E-28 | 7.49E-25 |
| *ENSGALG00000019063* | 9.31601106 | 4.7993067 | 3.22E-15 | 2.20E-12 |
| *ENSGALG00000029931* | 9.18141215 | 1.30246646 | 1.53E-08 | 1.60E-06 |
| *NRG1* | 9.16568907 | 4.84405269 | 1.78E-21 | 3.53E-18 |
| *FST* | 8.95534797 | 4.22613761 | 7.09E-13 | 2.65E-10 |
| *CAV3* | 8.88723152 | 3.40936749 | 8.53E-06 | 0.00029675 |
| *ACVR1C* | 8.78535748 | 0.98236204 | 5.62E-05 | 0.00137515 |
| *FGF9* | 8.67846395 | 1.94288968 | 1.09E-06 | 5.42E-05 |
| *CLDN1* | 8.61817353 | 3.56066467 | 6.82E-09 | 8.00E-07 |
| *FHL2* | 8.44907674 | 4.26529111 | 7.99E-06 | 0.00027983 |
| *ENSGALG00000040442* | 8.23119914 | 1.89309575 | 4.55E-08 | 4.06E-06 |
| *ENSGALG00000031108* | 8.18978192 | 2.7933508 | 4.47E-11 | 9.85E-09 |
| *ENSGALG00000031496* | 8.18562164 | 1.79695162 | 4.80E-07 | 2.77E-05 |
| *IYD* | 8.13586992 | 3.20609186 | 4.53E-09 | 5.51E-07 |
| *MMP9* | 8.06662271 | 6.40605895 | 2.10E-13 | 8.68E-11 |
| *CDH5* | 8.03574849 | 6.06195102 | 2.77E-07 | 1.77E-05 |
| *PPARGC1A* | 7.93497286 | 1.29766408 | 3.82E-06 | 0.00015622 |
| *BRINP3* | 7.92695764 | 0.91073721 | 0.00011984 | 0.00252723 |
| *ARHGAP25* | 7.85819588 | 2.80022669 | 1.77E-08 | 1.83E-06 |
| *ZEB2* | 7.73968158 | 5.31824092 | 1.90E-07 | 1.32E-05 |
| *ENSGALG00000031090* | 7.68133399 | 1.09418268 | 0.00019855 | 0.00382131 |
| *TMEM8C* | 7.53787707 | 0.98324009 | 0.00052056 | 0.0079869 |
| *LOXL4* | 7.48016857 | 3.80467935 | 1.94E-09 | 2.60E-07 |
| *ENSGALG00000032851* | 7.45716144 | 1.98229643 | 2.61E-06 | 0.00011399 |
| *CD34* | 7.4508642 | 4.88271369 | 1.52E-13 | 6.61E-11 |
| *ENSGALG00000027323* | 7.36797787 | 6.50744285 | 2.69E-06 | 0.0001167 |
| *KRT19* | 7.31824834 | 3.09863047 | 2.20E-06 | 9.91E-05 |
| *NPL* | 7.28543586 | 3.59415637 | 1.39E-11 | 3.57E-09 |
| *FILIP1* | 7.28207229 | 6.33813047 | 7.05E-25 | 2.00E-21 |
| *CD200* | 7.27738929 | 6.12048159 | 1.56E-25 | 5.14E-22 |
| *TBX2* | 7.27097573 | 5.29609982 | 7.18E-33 | 7.11E-29 |
| *ENSGALG00000003465* | 7.18026023 | 7.61266713 | 1.03E-07 | 7.92E-06 |
| *TMEM182* | 7.15071169 | 5.43931937 | 3.08E-06 | 0.00013057 |
| *KCNJ2* | 7.08384528 | 2.56417742 | 4.63E-08 | 4.09E-06 |
| *ENSGALG00000009844* | 7.06160203 | 8.66387585 | 7.01E-07 | 3.76E-05 |
| *ASB2* | 6.94299075 | 0.15904537 | 0.00016059 | 0.0032187 |
| *TNNT3* | 6.92641318 | 6.59450551 | 2.50E-06 | 0.0001102 |
| *OLFML1* | 6.8829206 | 2.1961234 | 0.00057771 | 0.00868222 |

| | | | | |
|---|---|---|---|---|
| *GJD2* | 6.86202325 | 2.32683473 | 0.00025947 | 0.00475361 |
| *ENSGALG00000029446* | 6.84545219 | 2.76781729 | 1.99E-08 | 2.01E-06 |
| *ENSGALG00000033466* | 6.83705595 | 1.06593097 | 0.00011766 | 0.00249549 |
| *VWA3B* | 6.83213211 | 2.29256182 | 2.79E-06 | 0.00012004 |
| *C7* | 6.726742 | 1.45838382 | 3.04E-05 | 0.00084171 |
| *SLC38A4* | 6.72664692 | 4.20335734 | 1.28E-06 | 6.21E-05 |
| *ENSGALG00000004518* | 6.71266962 | 8.924676 | 3.33E-06 | 0.00013988 |
| *JAKMIP1* | 6.69876982 | 4.64319704 | 1.68E-12 | 5.37E-10 |
| *MYOT* | 6.63786536 | 3.32396469 | 0.00038316 | 0.00641507 |
| *SMYD1* | 6.61940561 | 4.16656979 | 4.78E-05 | 0.00120641 |
| *ENSGALG00000015599* | 6.61338077 | 2.09440654 | 3.60E-05 | 0.00096558 |
| *ENSGALG00000011579* | 6.6003385 | 2.27484948 | 6.05E-05 | 0.0014601 |
| *ENSGALG00000009089* | 6.5888184 | 4.76440845 | 5.10E-05 | 0.00126924 |
| *ENSGALG00000042257* | 6.55799434 | 5.07803273 | 9.52E-05 | 0.00209545 |
| *ENSGALG00000045632* | 6.53256807 | 3.92985286 | 0.00022769 | 0.00426602 |
| *IRX4* | 6.53256571 | 3.20470208 | 3.85E-05 | 0.00102133 |
| *ENSGALG00000039977* | 6.48886098 | 5.51856159 | 2.26E-05 | 0.00066372 |
| *XIRP1* | 6.48167872 | 4.32033483 | 6.48E-05 | 0.00155285 |
| *EGR1* | 6.46701752 | 5.09907398 | 3.07E-10 | 5.28E-08 |
| *ENSGALG00000004436* | 6.4609651 | 2.21345119 | 5.63E-12 | 1.64E-09 |
| *GM27761* | 6.45025073 | 1.15369328 | 0.00019789 | 0.00382131 |
| *ADPRHL1* | 6.44942763 | 4.44119531 | 3.14E-05 | 0.00086597 |
| *DLL1* | 6.44133328 | 1.17615471 | 4.12E-07 | 2.43E-05 |
| *CACNG1* | 6.43381932 | 2.89346275 | 0.00041529 | 0.0067588 |
| *PLXNA4* | 6.41226139 | 2.40808752 | 5.12E-06 | 0.00019595 |
| *ENSGALG00000045739* | 6.40597561 | 0.92385605 | 3.55E-05 | 0.00095461 |
| *WNT11* | 6.38725798 | 4.44522127 | 1.92E-07 | 1.33E-05 |
| *MEF2C* | 6.36734164 | 8.20362655 | 7.44E-11 | 1.57E-08 |
| *PLK2* | 6.30168355 | 3.43058874 | 4.76E-05 | 0.00120574 |
| *FAM83B* | 6.3006299 | 2.85593122 | 0.00012383 | 0.00260023 |
| *UPK1B* | 6.27059168 | 3.05791351 | 2.18E-08 | 2.17E-06 |
| *SRRM4* | 6.25541271 | 1.2540822 | 8.80E-07 | 4.54E-05 |
| *HPSE2* | 6.2552415 | 1.0839412 | 3.99E-05 | 0.00105084 |
| *ENSGALG00000036780* | 6.22894767 | 4.75367884 | 1.05E-07 | 8.02E-06 |
| *ENSGALG00000046283* | 6.22306962 | 0.14536617 | 0.00031879 | 0.0055239 |
| *TRIM55* | 6.2230299 | 3.49946347 | 2.89E-05 | 0.00080891 |
| *ANO6* | 6.2115595 | 5.72162189 | 2.00E-11 | 4.96E-09 |
| *MYOG* | 6.20860366 | 4.08377822 | 2.79E-07 | 1.77E-05 |
| *SUSD3* | 6.16854439 | 3.29056676 | 1.11E-14 | 6.64E-12 |
| *MYL1* | 6.14035697 | 7.77393345 | 4.35E-06 | 0.00017326 |

| | | | | |
|---|---|---|---|---|
| *PITX2* | 6.13462973 | 3.51561406 | 2.85E-06 | 0.00012214 |
| *SERPINB2* | 6.13218375 | 4.39466746 | 4.63E-06 | 0.0001828 |
| *SLC43A2* | 6.12124483 | 3.10257046 | 1.16E-08 | 1.28E-06 |
| *FABP4* | 6.11613536 | 2.52768965 | 0.00015527 | 0.003144 |
| *HS3ST5* | 6.07058943 | 1.79038997 | 2.37E-07 | 1.56E-05 |
| *SLCO2A1* | 6.05764784 | 2.29267398 | 9.20E-06 | 0.0003165 |
| *EN1* | 6.04449674 | 1.21519137 | 0.00048509 | 0.00758954 |
| *RHOBTB1* | 6.0283833 | 2.4448201 | 3.36E-07 | 2.07E-05 |
| *SH3BGR* | 6.01725332 | 3.79671026 | 4.91E-06 | 0.00019072 |
| *ENSGALG00000004274* | 5.97566733 | 5.49491449 | 1.70E-07 | 1.20E-05 |
| *UNC45B* | 5.95150064 | 1.80662691 | 0.00051164 | 0.00788054 |
| *FBP2* | 5.94142466 | 0.94662511 | 6.37E-08 | 5.24E-06 |
| *ENSGALG00000044656* | 5.93586194 | 2.04192083 | 1.03E-07 | 7.92E-06 |
| *EMX2* | 5.92346771 | 1.6486761 | 1.16E-05 | 0.0003833 |
| *EPAS1* | 5.9163528 | 3.98619669 | 9.25E-16 | 7.98E-13 |
| *PTGS2* | 5.89686102 | 3.67399535 | 3.37E-10 | 5.61E-08 |
| *ENSGALG00000045426* | 5.8892574 | 6.10094838 | 8.40E-11 | 1.74E-08 |
| *ENSGALG00000044318* | 5.87673126 | 4.81532168 | 2.25E-13 | 9.10E-11 |
| *ENSGALG00000032272* | 5.85975842 | 0.93678961 | 2.65E-06 | 0.00011521 |
| *CBFA2T3* | 5.85279089 | 3.36971858 | 3.66E-14 | 1.86E-11 |
| *HOXB7* | 5.83621967 | 2.20139487 | 2.77E-06 | 0.00011941 |
| *MYL2* | 5.81225211 | 6.05829475 | 7.50E-05 | 0.00172683 |
| *PLVAP* | 5.80361277 | 2.71068695 | 2.11E-05 | 0.0006257 |
| *OSR1* | 5.80210009 | 5.98285697 | 5.08E-07 | 2.89E-05 |
| *PLXNB2* | 5.78578306 | 4.78953805 | 1.84E-14 | 1.04E-11 |
| *CD93* | 5.78565546 | 2.98635858 | 0.00028392 | 0.00508422 |
| *ENSGALG00000044476* | 5.78300368 | 1.53520663 | 0.00054802 | 0.00834356 |
| *ARHGEF9* | 5.78141261 | 2.56332841 | 4.29E-06 | 0.00017162 |
| *ENSGALG00000021399* | 5.78138398 | 4.38075927 | 2.75E-09 | 3.54E-07 |
| *TNNI1* | 5.77271957 | 4.86750306 | 7.17E-05 | 0.00166796 |
| *ENSGALG00000035827* | 5.76089567 | 1.06150004 | 2.67E-05 | 0.00075699 |
| *ENSGALG00000012830* | 5.75810399 | 1.73629558 | 0.00039247 | 0.00651036 |
| *CRYAB* | 5.71806339 | 3.69649023 | 6.23E-07 | 3.43E-05 |
| *ENSGALG00000028612* | 5.71573259 | 5.99805965 | 0.00011318 | 0.00242809 |
| *ENSGALG00000032550* | 5.70300587 | 6.97099644 | 0.00013227 | 0.00274545 |
| *DCX* | 5.69868552 | 4.76156375 | 1.32E-08 | 1.42E-06 |
| *ENSGALG00000035438* | 5.69291906 | 3.30584777 | 2.80E-07 | 1.77E-05 |
| *SLC22A16* | 5.68896009 | 5.78771454 | 2.19E-18 | 2.71E-15 |
| *RGS4* | 5.688567 | 2.69574058 | 2.67E-11 | 6.32E-09 |
| *ENSGALG00000000400* | 5.68560069 | 0.83988999 | 2.54E-05 | 0.00073146 |

| | | | | |
|---|---|---|---|---|
| *HS3ST1* | 5.67965487 | 2.63972555 | 6.54E-09 | 7.76E-07 |
| *CACNG4* | 5.67862236 | 1.84673099 | 0.00016225 | 0.00324558 |
| *CXCL14* | 5.66918487 | 4.97496773 | 8.59E-07 | 4.47E-05 |
| *ENSGALG00000006343* | 5.62519716 | 6.00200394 | 4.92E-09 | 5.94E-07 |
| *ARAP3* | 5.60742169 | 1.40163651 | 0.00041693 | 0.00678004 |
| *ENSGALG00000041375* | 5.58687666 | 1.41011398 | 5.14E-05 | 0.00127525 |
| *BST1* | 5.57801118 | 3.70821172 | 5.94E-07 | 3.29E-05 |
| *UTS2B* | 5.57495005 | 2.00880132 | 5.48E-06 | 0.00020684 |
| *ENSGALG00000008040* | 5.55367174 | 0.72295722 | 3.72E-07 | 2.23E-05 |
| *ENSGALG00000039101* | 5.51723875 | 2.1048732 | 0.0006633 | 0.00971615 |
| *ENSGALG00000037329* | 5.50318631 | 1.04628431 | 0.0003469 | 0.00593326 |
| *ENSGALG00000010927* | 5.49732967 | 7.3409333 | 2.48E-10 | 4.34E-08 |
| *AMIGO2* | 5.46530068 | 0.72297002 | 0.00022042 | 0.00414946 |
| *ABLIM2* | 5.45968529 | 3.83376743 | 2.61E-07 | 1.69E-05 |
| *ENSGALG00000034511* | 5.44921261 | 1.87979096 | 7.02E-05 | 0.0016486 |
| *RBM24* | 5.42777763 | 7.67172247 | 3.76E-15 | 2.48E-12 |
| *DCLK3* | 5.42544677 | 3.66595015 | 5.21E-08 | 4.53E-06 |
| *MYOZ2* | 5.4226314 | 5.16431806 | 1.86E-05 | 0.00056596 |
| *EDNRB* | 5.4137234 | 2.5977766 | 1.83E-05 | 0.00055875 |
| *LUM* | 5.41196414 | 6.57376522 | 3.63E-06 | 0.00015015 |
| *PRKAR2B* | 5.36383497 | 0.78938263 | 0.0004961 | 0.00770361 |
| *ENSGALG00000004360* | 5.35880427 | 1.74309674 | 3.26E-07 | 2.02E-05 |
| *ENSGALG00000045584* | 5.3473114 | 5.28929422 | 2.56E-18 | 2.98E-15 |
| *COL4A1* | 5.34135795 | 6.35642707 | 6.16E-06 | 0.00022617 |
| *HIC1* | 5.3359344 | 2.88910763 | 3.54E-07 | 2.15E-05 |
| *MAT1A* | 5.32910279 | 5.54782325 | 2.62E-05 | 0.00074694 |
| *ATF3* | 5.32845154 | 1.79569175 | 5.92E-06 | 0.0002198 |
| *BMP6* | 5.31643805 | 3.67582626 | 2.80E-06 | 0.00012006 |
| *GJA4* | 5.312962 | 3.07260149 | 2.72E-07 | 1.76E-05 |
| *P3H2* | 5.30158489 | 4.0219428 | 1.29E-12 | 4.38E-10 |
| *NES* | 5.2755361 | 6.47081845 | 2.69E-05 | 0.00076363 |
| *ENSGALG00000027624* | 5.27425681 | 1.33356527 | 8.63E-05 | 0.00193822 |
| *ARHGDIG* | 5.27105301 | 1.5893124 | 1.32E-05 | 0.00042319 |
| *POSTN* | 5.27041065 | 6.77376075 | 5.29E-06 | 0.00020101 |
| *SPON1* | 5.26456436 | 5.60265001 | 1.87E-07 | 1.30E-05 |
| *PDZRN4* | 5.24899846 | 3.64745423 | 1.31E-05 | 0.00042144 |
| *MYBPC3* | 5.23693155 | 4.63988704 | 7.04E-05 | 0.00164876 |
| *BCHE* | 5.2284087 | 2.11557007 | 1.29E-07 | 9.74E-06 |
| *ENSGALG00000038683* | 5.22762857 | 1.10220879 | 0.00032922 | 0.00568479 |
| *RSPO1* | 5.2269559 | 1.04899104 | 0.00028628 | 0.00510999 |

| | | | | |
|---|---|---|---|---|
| *ENSGALG00000037425* | 5.2190495 | 2.81465109 | 3.82E-06 | 0.00015622 |
| *ADCYAP1* | 5.21546734 | 1.17717722 | 2.12E-05 | 0.0006257 |
| *FGF7* | 5.20706687 | 2.77297942 | 0.00030557 | 0.00533691 |
| *LMO2* | 5.19099243 | 5.4224877 | 2.27E-14 | 1.22E-11 |
| *MYOM2* | 5.18887112 | 5.14493889 | 4.10E-06 | 0.00016522 |
| *TFPI2* | 5.16396903 | 1.84338875 | 0.00028389 | 0.00508422 |
| *ADAMTS15* | 5.14720345 | 2.56600054 | 4.31E-05 | 0.00111756 |
| *FAM69C* | 5.14619705 | 3.86066885 | 3.46E-08 | 3.27E-06 |
| *ENSGALG00000026395* | 5.12990091 | 0.60230901 | 0.00043079 | 0.0069654 |
| *PDK4* | 5.12291751 | 5.17021296 | 6.49E-09 | 7.74E-07 |
| *ENSGALG00000038251* | 5.11085915 | 3.0992465 | 3.98E-06 | 0.00016118 |
| *MYL10* | 5.10572786 | 7.61077686 | 0.00010501 | 0.0022775 |
| *ENSGALG00000042686* | 5.07422724 | 2.08694963 | 0.00028491 | 0.00509722 |
| *FGF1* | 5.06677955 | 2.08525301 | 2.90E-08 | 2.75E-06 |
| *EFEMP1* | 5.06044706 | 3.96790675 | 2.10E-08 | 2.11E-06 |
| *NDP* | 5.04922667 | 1.2864599 | 0.00030285 | 0.00530806 |
| *ENSGALG00000040785* | 5.03360506 | 4.1271103 | 1.08E-09 | 1.57E-07 |
| *APOBEC2* | 5.01543813 | 3.94026587 | 3.02E-06 | 0.00012839 |
| *SCIN* | 5.01456177 | 9.58737375 | 6.75E-18 | 7.04E-15 |
| *ATP6V1G3* | 5.00667842 | 3.12146355 | 3.51E-07 | 2.15E-05 |
| *ENSGALG00000038056* | 4.99575969 | 3.91234629 | 2.70E-11 | 6.32E-09 |
| *SYNPR* | 4.974183 | 2.54642146 | 8.69E-07 | 4.51E-05 |
| *ATP6V0D2* | 4.95568412 | 5.04723088 | 0.00038686 | 0.00644562 |
| *ENSGALG00000002012* | 4.93676178 | 3.1927869 | 0.00043323 | 0.00698204 |
| *PENK* | 4.90469281 | 5.54365776 | 0.0005738 | 0.00865073 |
| *PODXL* | 4.887464 | 4.97660581 | 6.94E-05 | 0.00164051 |
| *ENSGALG00000039966* | 4.87302594 | 3.85442503 | 4.99E-05 | 0.00124614 |
| *SNCA* | 4.84641944 | 1.70221608 | 9.46E-05 | 0.00208398 |
| *ENSGALG00000000302* | 4.84622907 | 7.35753119 | 3.18E-05 | 0.00087346 |
| *ALDOB* | 4.83679759 | 2.04514687 | 5.26E-06 | 0.00020011 |
| *NRN1* | 4.82868861 | 2.31963525 | 6.40E-07 | 3.47E-05 |
| *TPPP* | 4.82401098 | 3.56519278 | 5.18E-07 | 2.92E-05 |
| *CELF2* | 4.81288278 | 2.54347953 | 0.00045761 | 0.00726859 |
| *RGS2* | 4.7880165 | 5.47844677 | 2.38E-20 | 3.93E-17 |
| *CNR1* | 4.78340816 | 5.67252277 | 9.02E-12 | 2.48E-09 |
| *PIK3R1* | 4.76691893 | 2.87521313 | 2.91E-06 | 0.0001242 |
| *ASPA* | 4.73403548 | 1.12417069 | 0.00032221 | 0.00556862 |
| *MST1R* | 4.71697706 | 1.62994246 | 6.94E-05 | 0.00164051 |
| *ZFP804A* | 4.71579756 | 1.07411001 | 0.00013358 | 0.00276412 |
| *ATP2B2* | 4.70561144 | 6.88515655 | 6.85E-11 | 1.48E-08 |

| | | | | |
|---|---|---|---|---|
| *ENSGALG00000016292* | 4.69639849 | 3.23721848 | 5.48E-05 | 0.00134733 |
| *PPP1R1C* | 4.67953105 | 3.05013181 | 3.15E-05 | 0.00086597 |
| *KCNJ11* | 4.66560755 | 1.61849725 | 0.00011885 | 0.00251433 |
| *PAX7* | 4.66270229 | 2.26943569 | 0.00049358 | 0.00768601 |
| *PTPN7* | 4.65612374 | 1.60923998 | 0.00029867 | 0.00524406 |
| *ISL2* | 4.64520002 | 2.62671067 | 0.00014476 | 0.00296442 |
| *MICAL2* | 4.62324627 | 1.84812559 | 0.00010113 | 0.00220299 |
| *ENSGALG00000039821* | 4.62304166 | 2.9397638 | 2.08E-11 | 5.10E-09 |
| *MOXD1* | 4.61808066 | 4.11406853 | 1.15E-09 | 1.64E-07 |
| *PDGFA* | 4.61608205 | 5.59500926 | 1.09E-15 | 8.45E-13 |
| *ENSGALG00000016325* | 4.61058678 | 3.11683184 | 5.68E-05 | 0.00138338 |
| *ENSGALG00000013031* | 4.61027762 | 1.83465929 | 2.75E-07 | 1.77E-05 |
| *RAMP3* | 4.6001045 | 2.00375319 | 6.93E-07 | 3.72E-05 |
| *ST8SIA4* | 4.60003359 | 2.33245788 | 0.00033211 | 0.00572463 |
| *GUCY1A3* | 4.58560271 | 2.02857987 | 9.70E-05 | 0.00212604 |
| *TNFSF15* | 4.58379238 | 1.17774898 | 0.0002232 | 0.00419388 |
| *ENSGALG00000034071* | 4.55599778 | 0.22566167 | 0.00061003 | 0.00912646 |
| *TNNC2* | 4.53792702 | 7.77644536 | 5.44E-05 | 0.00134063 |
| *MYOD1* | 4.53555702 | 5.74718688 | 6.54E-06 | 0.00023916 |
| *PLXND1* | 4.52871161 | 3.45336276 | 1.39E-05 | 0.00044404 |
| *ENSGALG00000030546* | 4.52810595 | 1.3968518 | 0.00056124 | 0.00850578 |
| *MYL3* | 4.5238322 | 5.21536674 | 0.00041479 | 0.00675631 |
| *ENSGALG00000023818* | 4.52123778 | 3.22514572 | 1.34E-08 | 1.43E-06 |
| *DUSP26* | 4.51535943 | 2.46000068 | 3.95E-09 | 4.92E-07 |
| *LSP1* | 4.51363757 | 6.49280306 | 5.16E-08 | 4.50E-06 |
| *FAS* | 4.51039388 | 3.76896537 | 1.10E-09 | 1.58E-07 |
| *ENSGALG00000038238* | 4.50928129 | 5.04138788 | 3.41E-11 | 7.86E-09 |
| *ENSGALG00000030765* | 4.50825853 | 1.90027463 | 0.00024044 | 0.00446285 |
| *HNMT* | 4.49977943 | 0.99462021 | 0.00010015 | 0.00218406 |
| *NSG2* | 4.49886576 | 3.4292755 | 2.79E-05 | 0.00078501 |
| *BMF* | 4.49401022 | 5.26782995 | 2.19E-11 | 5.29E-09 |
| *AKR1D1* | 4.48699 | 7.34813444 | 1.99E-09 | 2.61E-07 |
| *ADAMTS5* | 4.48635898 | 2.73464323 | 2.57E-05 | 0.00073743 |
| *WNT9A* | 4.47695303 | 2.17098567 | 0.00026414 | 0.00482138 |
| *TMEM211* | 4.47419793 | 4.2739828 | 3.04E-10 | 5.28E-08 |
| *SOX12* | 4.45818609 | 0.63929363 | 0.00037702 | 0.0063336 |
| *CACNB4* | 4.4475685 | 1.88849103 | 1.80E-05 | 0.0005534 |
| *AQP1* | 4.43556607 | 6.79486732 | 4.66E-06 | 0.00018316 |
| *PDE8B* | 4.43346719 | 0.72194391 | 0.00048055 | 0.00753637 |
| *FAM20C* | 4.43217189 | 5.11041652 | 1.15E-10 | 2.22E-08 |

| | | | | |
|---|---|---|---|---|
| *ENSGALG00000008439* | 4.42948527 | 4.59461778 | 0.00066952 | 0.00977314 |
| *COL4A2* | 4.39201813 | 2.89071012 | 0.00050413 | 0.00778296 |
| *GALNTL6* | 4.38578451 | 1.74355929 | 0.00011215 | 0.00241331 |
| *ASTN2* | 4.36757073 | 1.67589321 | 9.58E-06 | 0.00032615 |
| *IGSF6* | 4.33204722 | 1.8681524 | 0.00052607 | 0.00805347 |
| *ENSGALG00000014999* | 4.32870259 | 6.71013436 | 2.20E-09 | 2.87E-07 |
| *ENSGALG00000032304* | 4.31781309 | 4.81029633 | 1.60E-05 | 0.00049606 |
| *ENSGALG00000008518* | 4.29953294 | 1.45256702 | 0.00011225 | 0.00241331 |
| *LAPTM5* | 4.29576688 | 4.17168841 | 6.76E-10 | 1.04E-07 |
| *AMN* | 4.27564164 | 0.43335644 | 0.0005296 | 0.00810048 |
| *LY75* | 4.26379734 | 5.91689983 | 2.00E-11 | 4.96E-09 |
| *ENSGALG00000043205* | 4.25408545 | 1.00161174 | 0.00012161 | 0.00255645 |
| *EGFL6* | 4.24011513 | 7.2841932 | 5.16E-05 | 0.00127594 |
| *ENSGALG00000012644* | 4.23491306 | 3.67230528 | 3.94E-07 | 2.33E-05 |
| *HES5* | 4.2342724 | 1.82351723 | 0.00040629 | 0.00666163 |
| *ENSGALG00000031149* | 4.2241837 | 4.17813849 | 4.50E-08 | 4.05E-06 |
| *MURC* | 4.22396929 | 3.8395276 | 9.12E-08 | 7.16E-06 |
| *CACNA1S* | 4.21838879 | 2.43293481 | 0.00018352 | 0.00358068 |
| *GPR137B* | 4.19009596 | 3.34845341 | 2.12E-05 | 0.0006257 |
| *KCTD12B* | 4.17799335 | 3.55543998 | 0.00010944 | 0.00236312 |
| *CXCR4* | 4.16900258 | 2.96207826 | 0.00045082 | 0.00720109 |
| *LIMCH1* | 4.16885092 | 3.28354383 | 2.62E-08 | 2.51E-06 |
| *ARID5B* | 4.16251001 | 5.15066762 | 4.26E-20 | 6.50E-17 |
| *HMOX1* | 4.14626491 | 4.41557782 | 3.87E-08 | 3.59E-06 |
| *TNNI2* | 4.13304243 | 6.06165352 | 0.00035806 | 0.00609252 |
| *ENSGALG00000045548* | 4.10268209 | 6.77043019 | 6.44E-10 | 9.99E-08 |
| *JAG2* | 4.10263898 | 2.88862929 | 0.00021862 | 0.00412333 |
| *CAPNS2* | 4.10213356 | 3.33760098 | 1.40E-09 | 1.94E-07 |
| *GLRX* | 4.0929923 | 2.6263588 | 0.00028859 | 0.00512191 |
| *MCF2L* | 4.08076304 | 3.36343792 | 7.29E-08 | 5.87E-06 |
| *ENSGALG00000044428* | 4.07573413 | 1.68676099 | 2.40E-05 | 0.00069871 |
| *FAM26E* | 4.04076461 | 2.82643188 | 0.00052456 | 0.00804207 |
| *EEF1A2* | 4.03899421 | 4.20354188 | 2.12E-05 | 0.0006257 |
| *RGS18* | 4.01639031 | 2.98060978 | 0.00054529 | 0.00830838 |
| *SLC6A2* | 4.01508344 | 6.46907912 | 8.60E-10 | 1.28E-07 |
| *ITGB1BP2* | 4.01381189 | 1.8928123 | 0.00011975 | 0.00252723 |
| *MITF* | 4.01239491 | 4.84560152 | 1.66E-09 | 2.29E-07 |
| *HHEX* | 3.99682941 | 4.14328703 | 3.70E-07 | 2.23E-05 |
| *ANK* | 3.99185333 | 6.09155512 | 3.10E-13 | 1.23E-10 |
| *SYNPO2* | 3.99165836 | 4.45068267 | 5.13E-08 | 4.50E-06 |

| | | | | |
|---|---|---|---|---|
| *EPHA3* | 3.99070834 | 6.18031871 | 1.06E-09 | 1.55E-07 |
| *TNNC1* | 3.98075148 | 7.92338987 | 0.00025295 | 0.00466439 |
| *SRL* | 3.9735192 | 4.43243 | 5.10E-07 | 2.90E-05 |
| *RANBP3L* | 3.97202572 | 2.18535192 | 0.00011851 | 0.00250979 |
| *ENSGALG00000006608* | 3.96801525 | 5.5507486 | 2.01E-07 | 1.37E-05 |
| *CITED4* | 3.96444687 | 4.34121974 | 3.70E-09 | 4.68E-07 |
| *RGCC* | 3.94426895 | 5.27470412 | 4.88E-10 | 7.81E-08 |
| *4930578C19RIK* | 3.93548323 | 3.56068671 | 0.00014504 | 0.00296718 |
| *LCP1* | 3.92651698 | 4.83072665 | 2.13E-05 | 0.00062693 |
| *ADAMTS17* | 3.92329368 | 3.65348081 | 2.05E-05 | 0.00061409 |
| *NRAP* | 3.91751893 | 5.51345889 | 6.90E-05 | 0.00163704 |
| *ENSGALG00000033677* | 3.91712576 | 2.83942493 | 0.00046199 | 0.00732053 |
| *ENSGALG00000010722* | 3.91509453 | 5.91333251 | 4.64E-07 | 2.68E-05 |
| *TFCP2L1* | 3.91016532 | 1.83997689 | 0.00029949 | 0.00525372 |
| *ENSGALG00000045039* | 3.8959132 | 1.62006995 | 2.64E-06 | 0.00011521 |
| *IBSP* | 3.89410692 | 9.80903178 | 1.94E-07 | 1.34E-05 |
| *SATB1* | 3.89244467 | 5.84197123 | 7.21E-17 | 7.15E-14 |
| *DLK1* | 3.89211692 | 3.49295934 | 7.69E-05 | 0.0017644 |
| *PLA2R1* | 3.88683768 | 7.1139131 | 9.98E-16 | 8.24E-13 |
| *ARHGAP45* | 3.87125237 | 4.40468589 | 6.51E-08 | 5.34E-06 |
| *CLDN5* | 3.85801008 | 1.58603884 | 0.00056538 | 0.00855543 |
| *PHLDB2* | 3.84031636 | 3.75490413 | 3.88E-11 | 8.74E-09 |
| *SLC38A2* | 3.82089221 | 8.9020886 | 1.35E-14 | 7.87E-12 |
| *BHLHE40* | 3.81878309 | 5.91606816 | 5.49E-13 | 2.09E-10 |
| *LY86* | 3.81158439 | 2.64052839 | 1.18E-06 | 5.78E-05 |
| *ACTN2* | 3.80962612 | 7.74306039 | 1.08E-05 | 0.00036067 |
| *NEDD9* | 3.80101593 | 2.61375011 | 2.24E-06 | 0.0001001 |
| *TRPC3* | 3.78495998 | 3.33871234 | 3.66E-06 | 0.00015034 |
| *PTGDS* | 3.77209066 | 1.95473995 | 0.00043737 | 0.00703305 |
| *EPCAM* | 3.77146776 | 3.9453435 | 0.00034943 | 0.00596622 |
| *GAB1* | 3.76015609 | 7.76945148 | 6.66E-15 | 4.26E-12 |
| *HPS1* | 3.75945634 | 5.01452308 | 1.02E-10 | 2.05E-08 |
| *CHODL* | 3.74662454 | 6.34700606 | 0.00038548 | 0.00643751 |
| *FAM198B* | 3.74308967 | 2.53247174 | 0.00025454 | 0.00468509 |
| *ENSGALG00000028238* | 3.74040695 | 0.98469683 | 0.000396 | 0.00655805 |
| *ENSGALG00000004521* | 3.73732839 | 6.55203854 | 5.19E-10 | 8.17E-08 |
| *RAMP1* | 3.72580666 | 3.24945809 | 2.58E-05 | 0.00074047 |
| *TIMP3* | 3.72005896 | 6.91448505 | 1.90E-10 | 3.42E-08 |
| *VDR* | 3.70920593 | 2.01747193 | 8.02E-05 | 0.00182683 |
| *HABP2* | 3.70778248 | 4.62579942 | 0.00013179 | 0.00274324 |

| | | | | |
|---|---|---|---|---|
| *HTRA3* | 3.70045296 | 3.97975547 | 2.07E-05 | 0.0006166 |
| *CNKSR3* | 3.69901033 | 3.72267035 | 6.36E-09 | 7.64E-07 |
| *SYT8* | 3.68937424 | 2.83427348 | 7.25E-05 | 0.00168317 |
| *OXSR1* | 3.68920165 | 3.52651337 | 2.48E-05 | 0.00071374 |
| *ENSGALG00000034387* | 3.6767864 | 3.19198633 | 9.06E-07 | 4.65E-05 |
| *GEM* | 3.67293955 | 3.95057374 | 2.03E-06 | 9.19E-05 |
| *GUCY1A2* | 3.66487209 | 2.83604321 | 0.00045644 | 0.00726172 |
| *BVES* | 3.65710802 | 4.14484296 | 9.15E-09 | 1.03E-06 |
| *ENSGALG00000003690* | 3.65558692 | 1.9623487 | 0.00037569 | 0.0063167 |
| *ENSGALG00000033433* | 3.64735849 | 3.46326554 | 0.00040554 | 0.00665477 |
| *ENSGALG00000034300* | 3.63147713 | 0.28956753 | 5.97E-05 | 0.00144407 |
| *ENSGALG00000005473* | 3.62496817 | 1.72662751 | 0.00016501 | 0.00327755 |
| *CADM3* | 3.61584071 | 0.64058644 | 0.00056527 | 0.00855543 |
| *ENSGALG00000043269* | 3.60703724 | 2.37537516 | 8.18E-05 | 0.00185279 |
| *MYOM1* | 3.60267795 | 6.18335881 | 0.00068609 | 0.00997091 |
| *ALDH1A3* | 3.59626044 | 4.36203174 | 1.29E-05 | 0.00041609 |
| *SMOX* | 3.59550757 | 3.72156688 | 1.33E-11 | 3.51E-09 |
| *ENSGALG00000036726* | 3.59289631 | 5.08180519 | 4.31E-08 | 3.95E-06 |
| *PCMTD1* | 3.5906549 | 6.36775996 | 1.06E-12 | 3.81E-10 |
| *FILIP1L* | 3.57615034 | 3.90740867 | 9.11E-05 | 0.00201719 |
| *WISP1* | 3.5759786 | 5.30229462 | 3.84E-09 | 4.82E-07 |
| *G0S2* | 3.57596153 | 2.1592504 | 7.23E-05 | 0.00168091 |
| *ENSGALG00000011086* | 3.57435941 | 6.59278806 | 0.00036917 | 0.00623875 |
| *UTS2R* | 3.5716113 | 3.34624943 | 0.00064363 | 0.00950714 |
| *GNG11* | 3.57152093 | 4.8729392 | 3.78E-07 | 2.25E-05 |
| *USP13* | 3.55895247 | 1.94539122 | 0.00039719 | 0.00656675 |
| *FLT4* | 3.55668074 | 4.52759061 | 5.45E-06 | 0.00020635 |
| *ACACB* | 3.52605424 | 1.04743882 | 0.00016425 | 0.00327238 |
| *ENSGALG00000005204* | 3.52602551 | 3.34932719 | 3.54E-05 | 0.000953 |
| *HIST2H2AA1* | 3.52399325 | 3.85173844 | 4.50E-08 | 4.05E-06 |
| *MGLL* | 3.50637807 | 3.70068811 | 6.86E-06 | 0.00024935 |
| *MTCL1* | 3.50387506 | 2.6575088 | 1.25E-05 | 0.00040661 |
| *GRIA2* | 3.48734913 | 3.72148584 | 0.00025618 | 0.00470653 |
| *GNG10* | 3.48390886 | 5.06489802 | 4.77E-10 | 7.68E-08 |
| *RAB31* | 3.47808904 | 3.1105484 | 1.36E-07 | 1.00E-05 |
| *ENSGALG00000038748* | 3.4749117 | 6.40579488 | 1.10E-06 | 5.48E-05 |
| *STEAP1* | 3.46354211 | 1.87047177 | 4.04E-05 | 0.00105973 |
| *ENSGALG00000042810* | 3.46164216 | 4.07459601 | 0.0002139 | 0.00405757 |
| *CHST9* | 3.45815796 | 3.54473426 | 4.57E-08 | 4.07E-06 |
| *1810011O10RIK* | 3.44444767 | 3.84403539 | 9.91E-06 | 0.00033237 |

| | | | | |
|---|---|---|---|---|
| *ENSGALG00000023936* | 3.44411357 | 4.22668479 | 0.00011065 | 0.00238412 |
| *SOD3* | 3.43326525 | 3.7492486 | 0.00020813 | 0.00396333 |
| *F13A1* | 3.42413119 | 3.50036314 | 5.46E-08 | 4.61E-06 |
| *TGFBR2* | 3.42394606 | 3.93195739 | 0.00044635 | 0.00714198 |
| *PDGFB* | 3.41884674 | 3.00307727 | 4.76E-05 | 0.00120574 |
| *PPFIBP2* | 3.41648427 | 5.2187839 | 1.07E-06 | 5.32E-05 |
| *CYP4V3* | 3.41364546 | 3.01161777 | 3.79E-05 | 0.00100742 |
| *ENSGALG00000042715* | 3.40744085 | 1.07960743 | 0.00040317 | 0.00662691 |
| *PLPP7* | 3.40676976 | 1.82115393 | 1.95E-05 | 0.00058755 |
| *NPR3* | 3.40167052 | 3.90010661 | 3.14E-06 | 0.00013287 |
| *ANKS1B* | 3.39728054 | 4.56314876 | 2.47E-06 | 0.00010918 |
| *ENSGALG00000033428* | 3.39444974 | 2.57944248 | 0.00013273 | 0.00274924 |
| *SLC38A1* | 3.38628706 | 5.50351811 | 1.19E-10 | 2.25E-08 |
| *PCOLCE2* | 3.3704394 | 4.04713744 | 4.26E-05 | 0.00110748 |
| *HOPX* | 3.36557171 | 2.71278914 | 2.49E-07 | 1.62E-05 |
| *EBF2* | 3.36349432 | 4.9285166 | 0.00017121 | 0.00338375 |
| *ENSGALG00000006190* | 3.36029825 | 5.19052119 | 0.00038627 | 0.00644533 |
| *DUSP6* | 3.34747843 | 4.15547045 | 1.49E-06 | 7.12E-05 |
| *CYYR1* | 3.34491115 | 1.97996084 | 6.01E-06 | 0.00022194 |
| *HPCAL1* | 3.33888587 | 6.93604252 | 1.15E-10 | 2.22E-08 |
| *PDGFC* | 3.33717686 | 4.95803214 | 2.89E-07 | 1.81E-05 |
| *VTI1A* | 3.3311834 | 5.9239519 | 2.17E-12 | 6.84E-10 |
| *CKB* | 3.3107209 | 9.06099848 | 4.07E-08 | 3.75E-06 |
| *ENSGALG00000025898* | 3.30468696 | 2.97923696 | 0.00019661 | 0.00380242 |
| *ENSGALG00000039455* | 3.30403585 | 1.84034485 | 6.87E-06 | 0.00024935 |
| *RBM38* | 3.30286069 | 5.61795647 | 2.87E-08 | 2.74E-06 |
| *CORO2B* | 3.29908574 | 4.03100057 | 1.46E-08 | 1.53E-06 |
| *SERPINF1* | 3.28444136 | 9.10626201 | 0.00013214 | 0.00274545 |
| *INSRR* | 3.2830711 | 1.89215916 | 0.0001998 | 0.00383787 |
| *ENSGALG00000001136* | 3.27643448 | 3.54432271 | 0.00041796 | 0.00679115 |
| *ENSGALG00000029644* | 3.27250557 | 1.35419469 | 0.00012112 | 0.00254889 |
| *THRB* | 3.26648973 | 1.82363334 | 0.00064197 | 0.00948971 |
| *TMC3* | 3.25151176 | 3.42618564 | 1.27E-05 | 0.00041397 |
| *HIVEP3* | 3.24942749 | 2.25435506 | 0.00041866 | 0.00679479 |
| *ENSGALG00000031177* | 3.24035808 | 3.55151831 | 1.85E-08 | 1.90E-06 |
| *RCSD1* | 3.23889811 | 4.12881068 | 1.24E-06 | 6.04E-05 |
| *FAM20A* | 3.23697278 | 4.7249683 | 8.78E-05 | 0.00196351 |
| *SPRY2* | 3.21925509 | 4.83071802 | 8.89E-11 | 1.80E-08 |
| *ITSN2* | 3.21898602 | 5.86910868 | 1.04E-09 | 1.52E-07 |
| *ENSGALG00000033694* | 3.20371265 | 5.62156759 | 1.72E-06 | 8.02E-05 |

| | | | | |
|---|---|---|---|---|
| *TNFRSF1B* | 3.20339968 | 2.93123047 | 0.0005605 | 0.00850102 |
| *WNT5B* | 3.19377326 | 2.61179448 | 4.60E-05 | 0.00116997 |
| *SFRP2* | 3.19268066 | 7.12471153 | 0.00017642 | 0.00346264 |
| *F2RL2* | 3.19253673 | 3.77030163 | 8.11E-06 | 0.00028337 |
| *CPM* | 3.18885928 | 5.04277859 | 9.38E-07 | 4.79E-05 |
| *PHYHIPL* | 3.17643732 | 2.42101917 | 0.00042462 | 0.00687123 |
| *VSTM4* | 3.1519067 | 5.4025429 | 3.52E-07 | 2.15E-05 |
| *TMEM26* | 3.12965838 | 6.10475543 | 6.48E-16 | 5.84E-13 |
| *LGI1* | 3.12124693 | 3.87281881 | 4.98E-05 | 0.00124614 |
| *ENSGALG00000033522* | 3.1183554 | 3.34442679 | 1.03E-08 | 1.15E-06 |
| *SGK1* | 3.11436394 | 6.27108674 | 7.27E-13 | 2.67E-10 |
| *DLX6* | 3.11054523 | 2.18636939 | 4.11E-05 | 0.00107624 |
| *ENSGALG00000007526* | 3.10811178 | 4.20245747 | 2.41E-05 | 0.00070058 |
| *ENSGALG00000044797* | 3.09217716 | 2.85992729 | 3.24E-06 | 0.00013655 |
| *FAM134B* | 3.08722535 | 2.50792939 | 0.00013188 | 0.00274324 |
| *ENSGALG00000032369* | 3.08627098 | 5.16302928 | 1.67E-11 | 4.25E-09 |
| *PLCL2* | 3.08269645 | 3.26167094 | 3.13E-07 | 1.95E-05 |
| *MXRA7* | 3.07816092 | 2.93083658 | 0.00011665 | 0.00248379 |
| *MAP3K5* | 3.07519864 | 8.92324446 | 3.15E-10 | 5.33E-08 |
| *TRIM36* | 3.06559559 | 3.05047924 | 0.00017468 | 0.0034386 |
| *TOX2* | 3.06400923 | 3.39592898 | 0.00010738 | 0.00232123 |
| *PKIB* | 3.06181245 | 2.47538271 | 0.00023305 | 0.00434999 |
| *SERINC5* | 3.05957771 | 4.29399647 | 3.34E-05 | 0.00091149 |
| *ENSGALG00000032186* | 3.02925167 | 2.46183553 | 2.92E-05 | 0.00081697 |
| *PCYOX1* | 3.01554132 | 6.01998037 | 5.33E-08 | 4.57E-06 |
| *ENSGALG00000012420* | 3.01456313 | 6.0650705 | 0.00042109 | 0.00682532 |
| *CDKN1A* | 3.00640808 | 2.55997419 | 8.20E-05 | 0.00185374 |
| *GLIPR2* | 3.00539396 | 4.97328949 | 4.48E-06 | 0.00017779 |
| *ENSGALG00000020626* | 2.98637985 | 5.79574283 | 1.07E-08 | 1.19E-06 |
| *ENSGALG00000031365* | 2.98568763 | 4.36183827 | 7.94E-08 | 6.35E-06 |
| *FBXO31* | 2.98550849 | 2.45945521 | 0.00019341 | 0.00375518 |
| *ENPP6* | 2.98085372 | 3.6246147 | 0.00038542 | 0.00643751 |
| *ARSB* | 2.98084709 | 4.04107031 | 2.05E-05 | 0.00061389 |
| *HIP1* | 2.97199798 | 4.62363204 | 2.09E-07 | 1.41E-05 |
| *EGFR* | 2.97043358 | 5.25821165 | 1.43E-07 | 1.05E-05 |
| *CNRIP1* | 2.96924792 | 1.9763655 | 8.23E-05 | 0.00185795 |
| *AHR* | 2.9637964 | 5.46875602 | 1.08E-10 | 2.13E-08 |
| *ENSGALG00000013655* | 2.95133077 | 2.87633761 | 6.04E-05 | 0.0014601 |
| *AIG1* | 2.94935379 | 5.25692409 | 1.91E-08 | 1.95E-06 |
| *FZD4* | 2.94860033 | 4.26319612 | 0.00025318 | 0.00466439 |

| | | | | |
|---|---|---|---|---|
| *RSAD2* | 2.94403318 | 3.09660622 | 1.86E-05 | 0.00056596 |
| *ENSGALG00000031934* | 2.94271551 | 6.55862742 | 4.90E-05 | 0.00123131 |
| *ENSGALG00000031844* | 2.92572783 | 4.32942504 | 1.37E-06 | 6.59E-05 |
| *MUSTN1* | 2.91858164 | 3.98371036 | 0.0005089 | 0.00784439 |
| *ANXA1* | 2.9185087 | 4.85248606 | 7.56E-07 | 4.00E-05 |
| *INHBA* | 2.91682918 | 3.45500256 | 0.000573 | 0.00865073 |
| *ENSGALG00000036014* | 2.91528573 | 2.86935175 | 0.00015779 | 0.00317876 |
| *PPDPF* | 2.90273064 | 5.687926 | 2.88E-06 | 0.00012294 |
| *ENSGALG00000024296* | 2.90125658 | 2.40893396 | 0.00039547 | 0.00655475 |
| *CMIP* | 2.89926163 | 4.46370559 | 3.09E-10 | 5.28E-08 |
| *ENSGALG00000043750* | 2.89451169 | 3.509127 | 2.93E-05 | 0.00081697 |
| *ID1* | 2.87400833 | 5.42322148 | 6.68E-07 | 3.60E-05 |
| *ENSGALG00000025937* | 2.86317309 | 6.33965182 | 1.08E-10 | 2.13E-08 |
| *COL6A3* | 2.8397396 | 6.95189234 | 1.68E-05 | 0.00051833 |
| *TMEM229B* | 2.83713207 | 3.43392055 | 5.19E-06 | 0.00019811 |
| *SLC13A5* | 2.82958521 | 6.04593465 | 1.46E-05 | 0.00046205 |
| *TGFBR3* | 2.8292288 | 3.332384 | 8.95E-05 | 0.00198942 |
| *SLC9A3R1* | 2.81317435 | 3.89297658 | 1.01E-06 | 5.10E-05 |
| *MYH11* | 2.81235336 | 5.25555068 | 9.61E-06 | 0.00032615 |
| *ENSGALG00000043956* | 2.80684257 | 5.4970676 | 6.45E-10 | 9.99E-08 |
| *ENSGALG00000009689* | 2.80431657 | 4.72750007 | 0.00016486 | 0.00327755 |
| *ENSGALG00000046534* | 2.80319325 | 3.32698565 | 7.37E-08 | 5.92E-06 |
| *CSRP2* | 2.80214868 | 6.2345634 | 1.48E-05 | 0.00046534 |
| *ENSGALG00000034967* | 2.7992933 | 5.78018697 | 1.08E-12 | 3.83E-10 |
| *TEK* | 2.79090186 | 3.41649125 | 2.44E-05 | 0.00070643 |
| *MAP1LC3A* | 2.78391009 | 6.45947062 | 2.85E-12 | 8.70E-10 |
| *PRRX2* | 2.78053093 | 5.37687951 | 7.18E-06 | 0.0002568 |
| *TNFSF10* | 2.77447452 | 5.3660529 | 1.22E-08 | 1.32E-06 |
| *TGM2* | 2.77091472 | 5.26124118 | 2.96E-06 | 0.00012601 |
| *TBC1D4* | 2.76991459 | 5.31879603 | 3.27E-06 | 0.00013753 |
| *PDGFD* | 2.76806905 | 7.69674985 | 0.00016195 | 0.00324276 |
| *CENPV* | 2.76734399 | 4.25217183 | 9.70E-08 | 7.54E-06 |
| *HS6ST1* | 2.75441418 | 5.15976883 | 1.14E-05 | 0.0003778 |
| *PITPNC1* | 2.74870378 | 2.34632974 | 0.00063201 | 0.00937746 |
| *SLC15A2* | 2.74799274 | 3.08184389 | 0.00055371 | 0.00841737 |
| *BAMBI* | 2.74766067 | 8.10748177 | 4.58E-05 | 0.00116813 |
| *FHL3* | 2.74676735 | 6.99057732 | 6.07E-08 | 5.06E-06 |
| *CXCL12* | 2.74011112 | 5.94356581 | 2.07E-05 | 0.00061824 |
| *ENSGALG00000041228* | 2.73463873 | 6.41715716 | 6.54E-05 | 0.00156295 |
| *ENSGALG00000031593* | 2.72671153 | 8.65435114 | 9.76E-06 | 0.0003301 |

| | | | | |
|---|---|---|---|---|
| *MAOA* | 2.7213264 | 5.41686968 | 9.34E-07 | 4.79E-05 |
| *ENSGALG00000015219* | 2.7160468 | 1.83842612 | 0.00019285 | 0.00374793 |
| *CYTH1* | 2.71261511 | 4.91531272 | 1.23E-08 | 1.32E-06 |
| *TTC9* | 2.70870019 | 4.49606691 | 8.19E-05 | 0.00185374 |
| *EPB41* | 2.70819914 | 4.33805717 | 3.47E-05 | 0.00093839 |
| *CMTM8* | 2.70755994 | 3.0902988 | 4.11E-06 | 0.00016522 |
| *NRADD* | 2.70148322 | 3.89685773 | 0.000494 | 0.00768648 |
| *FOS* | 2.69874574 | 5.98874088 | 8.31E-05 | 0.00187395 |
| *DYNC1I1* | 2.69849431 | 4.09604128 | 1.03E-06 | 5.19E-05 |
| *TRP53I11* | 2.68511871 | 5.9575445 | 2.09E-05 | 0.00062206 |
| *SHE* | 2.68148292 | 3.83269091 | 7.35E-05 | 0.00170098 |
| *TCP11L2* | 2.66836976 | 7.22224637 | 3.35E-10 | 5.61E-08 |
| *AASDHPPT* | 2.65919127 | 3.81177889 | 2.98E-05 | 0.00083036 |
| *YPEL5* | 2.65888704 | 6.78789732 | 7.71E-14 | 3.55E-11 |
| *NELL2* | 2.65219775 | 6.17107594 | 2.23E-08 | 2.20E-06 |
| *RHOB* | 2.65146403 | 7.59114252 | 4.37E-10 | 7.16E-08 |
| *PDE5A* | 2.65083366 | 3.41633377 | 6.44E-07 | 3.49E-05 |
| *ENSGALG00000037332* | 2.64431043 | 4.33061173 | 0.00013263 | 0.00274924 |
| *ETS1* | 2.64076663 | 5.91747255 | 1.46E-12 | 4.82E-10 |
| *ENSGALG00000003670* | 2.63754802 | 4.63241514 | 0.00011381 | 0.00243901 |
| *ENSGALG00000027571* | 2.62285674 | 5.26557638 | 3.81E-10 | 6.29E-08 |
| *TIMP4* | 2.6001061 | 6.77792309 | 1.59E-06 | 7.50E-05 |
| *RAD51B* | 2.59311574 | 3.33375696 | 6.97E-05 | 0.00164242 |
| *INF2* | 2.58672044 | 2.81987482 | 8.15E-05 | 0.00185032 |
| *CRMP1* | 2.58316938 | 5.38708495 | 3.83E-11 | 8.72E-09 |
| *ALCAM* | 2.54481331 | 6.44641273 | 0.00012675 | 0.00265327 |
| *VAT1* | 2.53646507 | 2.93894872 | 5.85E-06 | 0.00021835 |
| *ENSGALG00000006344* | 2.53633316 | 4.25277682 | 5.66E-05 | 0.00138278 |
| *SPARC* | 2.51995883 | 11.590411 | 5.95E-08 | 4.98E-06 |
| *FOXO1* | 2.51959396 | 6.48272756 | 1.45E-08 | 1.53E-06 |
| *ENSGALG00000033148* | 2.51679389 | 2.23232089 | 0.00015743 | 0.00317464 |
| *CD44* | 2.51300097 | 6.52951191 | 0.00020658 | 0.00394519 |
| *FBXO32* | 2.51199424 | 4.91501774 | 7.68E-09 | 8.95E-07 |
| *SLK* | 2.51157348 | 5.70586033 | 5.53E-05 | 0.00135674 |
| *RAPH1* | 2.51038727 | 4.58554097 | 7.63E-05 | 0.00175199 |
| *PIK3AP1* | 2.49947371 | 2.51736313 | 0.00016688 | 0.00330806 |
| *ENSGALG00000012821* | 2.49157763 | 5.35144047 | 2.18E-07 | 1.45E-05 |
| *TCF7L2* | 2.48379448 | 9.87221852 | 2.07E-10 | 3.67E-08 |
| *FBXL5* | 2.47061372 | 6.7736379 | 9.75E-08 | 7.55E-06 |
| *COL3A1* | 2.45748006 | 9.64359395 | 0.00016544 | 0.00328278 |

| | | | | |
|---|---|---|---|---|
| *ENSGALG00000011695* | 2.44572318 | 7.89624555 | 5.92E-08 | 4.97E-06 |
| *ENSGALG00000015293* | 2.44561027 | 3.13237906 | 7.50E-05 | 0.00172683 |
| *GM13889* | 2.41211575 | 2.81702744 | 0.00028861 | 0.00512191 |
| *OSBPL5* | 2.41175056 | 3.37030529 | 0.00033872 | 0.00581844 |
| *RHOC* | 2.40767968 | 3.52682971 | 4.86E-05 | 0.00122193 |
| *PRR5* | 2.40432642 | 4.44349938 | 9.43E-06 | 0.00032326 |
| *KHDRBS3* | 2.39902915 | 5.54735101 | 1.02E-06 | 5.17E-05 |
| *CTGF* | 2.39423486 | 8.82857743 | 5.22E-06 | 0.00019885 |
| *SSBP2* | 2.39396598 | 6.45297121 | 4.74E-08 | 4.17E-06 |
| *AIFM2* | 2.38894072 | 5.92016333 | 1.30E-05 | 0.00041799 |
| *ENSGALG00000029731* | 2.38397266 | 4.41612743 | 3.04E-05 | 0.00084171 |
| *WNK2* | 2.37570795 | 4.49419371 | 8.94E-06 | 0.00030897 |
| *PLCXD1* | 2.37540384 | 2.26540165 | 0.00014782 | 0.00301776 |
| *WBP1L* | 2.37286665 | 6.26060388 | 5.69E-06 | 0.00021339 |
| *ENSGALG00000030583* | 2.36460247 | 5.05472764 | 1.97E-06 | 8.94E-05 |
| *ENSGALG00000030635* | 2.34744943 | 6.6979172 | 1.68E-06 | 7.89E-05 |
| *SCG5* | 2.34462622 | 5.02986675 | 1.96E-09 | 2.60E-07 |
| *TIPARP* | 2.34122545 | 5.61756389 | 3.66E-08 | 3.42E-06 |
| *FN1* | 2.33767927 | 8.12865984 | 5.87E-07 | 3.27E-05 |
| *PTCHD1* | 2.31152159 | 4.08516604 | 1.84E-06 | 8.50E-05 |
| *STON1* | 2.31015364 | 5.17024968 | 5.04E-10 | 7.99E-08 |
| *SLC8B1* | 2.30853536 | 3.56301378 | 0.00011406 | 0.00244164 |
| *GM2A* | 2.30654594 | 4.57301676 | 2.32E-05 | 0.00067827 |
| *EEPD1* | 2.30351282 | 4.93315268 | 3.66E-06 | 0.00015034 |
| *ENSGALG00000015416* | 2.30098134 | 4.2737377 | 1.58E-06 | 7.49E-05 |
| *ENSGALG00000032933* | 2.2932764 | 4.44606704 | 7.07E-06 | 0.00025448 |
| *ENSGALG00000015019* | 2.29056762 | 4.15817152 | 5.08E-05 | 0.00126832 |
| *ELOVL6* | 2.28258331 | 6.12864906 | 4.90E-06 | 0.00019072 |
| *RTN1* | 2.2779027 | 4.11504297 | 0.00019477 | 0.00377404 |
| *ENSGALG00000024332* | 2.27702433 | 2.30063803 | 0.00065531 | 0.00962952 |
| *NTNG1* | 2.25321474 | 4.86940272 | 1.71E-06 | 7.99E-05 |
| *FSTL4* | 2.24730536 | 6.55587761 | 0.00043861 | 0.0070429 |
| *BNIP3* | 2.24315196 | 6.6754281 | 6.56E-07 | 3.54E-05 |
| *ENSGALG00000037459* | 2.23287819 | 3.83347803 | 0.00015166 | 0.00308969 |
| *PLPP3* | 2.23183037 | 5.70281766 | 5.34E-08 | 4.57E-06 |
| *LDAH* | 2.23182547 | 5.40571263 | 1.82E-09 | 2.49E-07 |
| *RASSF2* | 2.21909714 | 4.90630063 | 0.00052612 | 0.00805347 |
| *SEMA3A* | 2.21084723 | 4.87046297 | 7.89E-09 | 9.15E-07 |
| *ACTN1* | 2.20451146 | 7.69989118 | 1.52E-07 | 1.09E-05 |
| *ID2* | 2.20189666 | 8.56015003 | 1.71E-10 | 3.11E-08 |

| | | | | |
|---|---|---|---|---|
| *AAED1* | 2.19613011 | 4.02200138 | 0.0004464 | 0.00714198 |
| *MYLIP* | 2.19172992 | 6.6121203 | 2.74E-06 | 0.00011846 |
| *ADHFE1* | 2.18543658 | 3.12028079 | 0.00038914 | 0.00646592 |
| *MYO6* | 2.17668232 | 8.17868098 | 0.00017072 | 0.00337748 |
| *TBC1D1* | 2.17146411 | 6.83687542 | 1.91E-09 | 2.57E-07 |
| *RALGPS2* | 2.16765259 | 6.554713 | 2.25E-08 | 2.21E-06 |
| *HSPB1* | 2.15996385 | 3.41381729 | 3.98E-05 | 0.00104941 |
| *ANGPT2* | 2.15909181 | 5.03015733 | 0.00051519 | 0.0079229 |
| *FBXO8* | 2.15801108 | 3.91511077 | 5.43E-06 | 0.00020562 |
| *MEX3B* | 2.15655971 | 4.19949593 | 5.10E-05 | 0.00126892 |
| *SESN1* | 2.15009395 | 5.55955072 | 4.79E-05 | 0.0012076 |
| *JAGN1* | 2.14282161 | 3.71646894 | 0.00062059 | 0.00923577 |
| *H1F0* | 2.12942187 | 3.39726793 | 0.00042165 | 0.0068287 |
| *KANK1* | 2.12936186 | 7.43721169 | 7.82E-10 | 1.19E-07 |
| *PCDH18* | 2.12401009 | 5.31038825 | 0.00018464 | 0.00359889 |
| *RND3* | 2.12321265 | 5.16018162 | 7.27E-06 | 0.00025858 |
| *CPZ* | 2.11857313 | 6.4900433 | 0.0003121 | 0.00542699 |
| *KLHL24* | 2.11691938 | 6.14629172 | 2.09E-07 | 1.41E-05 |
| *IFITM5* | 2.11431561 | 8.56243483 | 3.42E-05 | 0.00092747 |
| *ENSGALG00000043484* | 2.1108226 | 3.37532327 | 5.93E-06 | 0.0002198 |
| *ENSGALG00000041998* | 2.08733852 | 5.62117705 | 3.82E-05 | 0.0010153 |
| *MEF2A* | 2.08410669 | 8.55894945 | 1.52E-06 | 7.24E-05 |
| *S100A11* | 2.0669603 | 6.32727541 | 0.00040147 | 0.00661549 |
| *MAF* | 2.06305456 | 4.98425907 | 4.50E-06 | 0.00017834 |
| *RASA3* | 2.06004549 | 6.31306247 | 1.94E-07 | 1.34E-05 |
| *ENSGALG00000043817* | 2.04486669 | 5.1968412 | 1.77E-06 | 8.21E-05 |
| *AMACR* | 2.04252541 | 4.00374487 | 2.81E-05 | 0.00078996 |
| *TEX264* | 2.02618392 | 5.59613976 | 2.00E-05 | 0.00060099 |
| *MXRA8* | 2.02070569 | 6.87628105 | 0.00022987 | 0.00430291 |
| *ENSGALG00000030902* | 2.00420653 | 7.66638115 | 1.35E-07 | 1.00E-05 |

**Supplementary table S3.** Upregulated genes in head IMM compared to head MAT

| Gene names | logFC | logCPM | PValue | p_adjusted |
|---|---|---|---|---|
| *NOV* | 10.832989 | 8.41875019 | 1.96E-09 | 4.86E-06 |
| *ENSGALG00000042795* | 8.95434862 | 2.87469299 | 1.14E-10 | 4.53E-07 |
| *PEF1* | 8.5601374 | 2.97065538 | 5.10E-08 | 5.28E-05 |
| *CHST12* | 8.28329738 | 2.26077146 | 2.93E-06 | 0.00101898 |
| *ENSGALG00000035945* | 8.21657561 | 1.38777337 | 8.11E-06 | 0.00217293 |

| | | | | |
|---|---|---|---|---|
| *ENSGALG00000017815* | 8.05705901 | 3.34955614 | 6.60E-09 | 1.09E-05 |
| *LRP2BP* | 7.6563228 | 2.24426227 | 8.39E-07 | 0.00037821 |
| *SOCS1* | 7.57435056 | 1.4805549 | 1.25E-05 | 0.0030137 |
| *ENSGALG00000008032* | 7.41180738 | 1.96970883 | 9.38E-07 | 0.00041299 |
| *FMOD* | 6.83270053 | 4.1910396 | 9.00E-13 | 5.95E-09 |
| *CLEC18A* | 6.68665862 | 2.79931748 | 5.77E-06 | 0.00175654 |
| *ENSGALG00000014501* | 5.99249328 | 8.69605087 | 1.33E-06 | 0.00054683 |
| *ENSGALG00000043064* | 5.98056422 | 5.99045146 | 6.54E-07 | 0.000314 |
| *MGST2* | 5.52530733 | 1.57506155 | 6.52E-05 | 0.00964455 |
| *LOXL3* | 5.36762586 | 2.25692948 | 4.48E-05 | 0.00753149 |
| *GPR37L1* | 5.32067135 | 3.43002107 | 1.74E-05 | 0.0039231 |
| *ENSGALG00000036034* | 4.72939428 | 6.46973251 | 3.99E-06 | 0.00131752 |
| *ENSGALG00000010801* | 4.70334831 | 1.81699861 | 5.88E-05 | 0.00897507 |
| *RHBDF1* | 4.6206216 | 4.29378306 | 2.61E-09 | 5.76E-06 |
| *ENSGALG00000026650* | 4.59597033 | 4.9260032 | 1.97E-05 | 0.00428715 |
| *COMP* | 4.27771151 | 3.64577347 | 3.09E-05 | 0.00592059 |
| *FBLN7* | 4.22429005 | 5.26082245 | 1.31E-08 | 1.86E-05 |
| *ENSGALG00000021171* | 4.16879325 | 5.13396943 | 1.94E-08 | 2.57E-05 |
| *ENSGALG00000032803* | 4.15553343 | 3.94684199 | 1.38E-06 | 0.00054683 |
| *ENSGALG00000024498* | 4.12707094 | 2.03784695 | 2.43E-06 | 0.00086037 |
| *PTN* | 4.12687656 | 10.8053697 | 5.33E-08 | 5.28E-05 |
| *ENSGALG00000046360* | 4.12478246 | 4.34549189 | 1.10E-07 | 8.75E-05 |
| *GTF3C4* | 4.06580024 | 2.52761147 | 7.73E-06 | 0.00212843 |
| *NLK* | 4.03476892 | 1.57233369 | 6.51E-05 | 0.00964455 |
| *CAMK4* | 4.02065697 | 3.87646136 | 7.46E-09 | 1.14E-05 |
| *ITM2C* | 3.94868732 | 4.37420641 | 3.06E-08 | 3.57E-05 |
| *ENSGALG00000007526* | 3.8059983 | 4.20245747 | 2.53E-05 | 0.00523089 |
| *ELL2* | 3.58284684 | 3.23250961 | 2.45E-05 | 0.00510616 |
| *RANBP10* | 3.56833405 | 1.92305169 | 5.89E-05 | 0.00897507 |
| *GPR171* | 3.50962717 | 2.73286635 | 1.16E-05 | 0.00284802 |
| *TMEM39B* | 3.50299679 | 3.81748274 | 6.32E-09 | 1.09E-05 |
| *IKBIP* | 3.40935564 | 3.25137346 | 1.49E-05 | 0.00338401 |
| *YIPF1* | 3.24304725 | 3.39292378 | 5.12E-06 | 0.00161149 |
| *CTHRC1* | 3.14737278 | 8.04577801 | 2.09E-06 | 0.00078077 |
| *TNC* | 3.11989054 | 8.13431872 | 5.26E-05 | 0.00840634 |
| *ANGPTL1* | 3.05454917 | 5.76105 | 3.76E-07 | 0.00021946 |
| *CD9* | 2.99343701 | 4.99904661 | 4.33E-07 | 0.00023565 |
| *ENPP2* | 2.98622886 | 6.06454431 | 6.28E-09 | 1.09E-05 |
| *NET1* | 2.90702808 | 7.76965903 | 1.11E-05 | 0.00281981 |
| *YIPF3* | 2.86501617 | 4.7716289 | 5.42E-07 | 0.00027549 |

| | | | | |
|---|---|---|---|---|
| *ENSGALG00000011894* | 2.82247519 | 5.31497868 | 2.04E-07 | 0.00013916 |
| *ENSGALG00000035496* | 2.79755977 | 6.22555298 | 4.40E-07 | 0.00023565 |
| *MXRA8* | 2.7082439 | 6.87628105 | 4.90E-05 | 0.00809204 |
| *ENSGALG00000032177* | 2.69271928 | 3.55025958 | 3.91E-05 | 0.00675082 |
| *CINP* | 2.68266446 | 3.26073893 | 2.42E-05 | 0.00509937 |
| *FGFRL1* | 2.61646813 | 7.28963894 | 3.38E-07 | 0.00021589 |
| *ENSGALG00000006300* | 2.58124153 | 8.46293881 | 3.03E-05 | 0.00592059 |
| *PDGFRL* | 2.54110689 | 5.5121173 | 6.20E-08 | 5.34E-05 |
| *CTSB* | 2.46257727 | 4.80982715 | 2.25E-05 | 0.00478751 |
| *ENSGALG00000036847* | 2.42426177 | 7.23389613 | 6.16E-06 | 0.00179451 |
| *KDELR3* | 2.40366776 | 6.60733578 | 3.90E-05 | 0.00675082 |
| *SNTB1* | 2.34315506 | 6.8473437 | 6.81E-07 | 0.000314 |
| *CD151* | 2.33328608 | 6.35696249 | 2.18E-06 | 0.00080153 |
| *PTTG1IP* | 2.30580199 | 6.17629221 | 9.01E-06 | 0.0023812 |
| *NINJ1* | 2.29681012 | 4.55794103 | 1.28E-06 | 0.00054683 |
| *MMP16* | 2.2746367 | 6.31332994 | 2.70E-05 | 0.00552381 |
| *FSTL1* | 2.23648803 | 8.46960799 | 6.75E-06 | 0.00191105 |
| *MSRA* | 2.22250632 | 4.25500758 | 7.50E-06 | 0.00209337 |
| *KLF11* | 2.2107926 | 4.79112176 | 4.29E-05 | 0.00726915 |
| *ENSGALG00000008021* | 2.16364155 | 5.12199058 | 1.13E-05 | 0.00281981 |
| *GPC4* | 2.14688952 | 8.24601672 | 4.29E-08 | 4.73E-05 |
| *ENSGALG00000040371* | 2.12477111 | 6.69497181 | 3.30E-05 | 0.00616503 |
| *DYNLT3* | 2.1001551 | 5.96518756 | 3.44E-05 | 0.00631232 |
| *MYDGF* | 2.06697856 | 6.70028387 | 1.89E-07 | 0.00013408 |
| *UTP15* | 2.03294663 | 5.65950327 | 5.51E-05 | 0.00866177 |

**Supplemental table S4.** Upregulated genes in head MAT compared to head IMM

| Gene names | logFC | logCPM | PValue | p_adjusted |
|---|---|---|---|---|
| *IBSP* | 8.08550799 | 9.80903178 | 7.76E-13 | 5.95E-09 |
| *INSRR* | 7.32594626 | 1.89215916 | 6.68E-07 | 0.000314 |
| *ENSGALG00000044866* | 6.62200811 | 5.34344274 | 3.61E-11 | 1.79E-07 |
| *IFITM5* | 5.9188543 | 8.56243483 | 2.91E-17 | 5.77E-13 |
| *SPP1* | 5.41463601 | 11.9902669 | 6.46E-08 | 5.34E-05 |
| *IRX6* | 5.2709771 | 5.03032033 | 1.36E-09 | 3.85E-06 |
| *ENSGALG00000043071* | 5.26843783 | 7.53176477 | 7.88E-06 | 0.00214087 |
| *ADAMTSL5* | 5.17024734 | 1.84013533 | 3.64E-06 | 0.00122367 |
| *ENSGALG00000040306* | 4.60640838 | 9.09877407 | 3.55E-07 | 0.00021788 |
| *NHEJ1* | 4.49428137 | 4.1473014 | 4.13E-07 | 0.00023419 |

| | | | | |
|---|---|---|---|---|
| *COL10A1* | 4.42253366 | 6.12798575 | 3.34E-05 | 0.00618712 |
| *ENSGALG00000032449* | 4.41673215 | 7.52780518 | 2.24E-06 | 0.00080667 |
| *ENSGALG00000044239* | 4.21815192 | 17.1002618 | 7.10E-10 | 2.35E-06 |
| *MYO6* | 4.19905643 | 8.17868098 | 2.13E-08 | 2.63E-05 |
| *COL22A1* | 4.12628608 | 7.98368694 | 6.25E-08 | 5.34E-05 |
| *SLC6A2* | 3.81594138 | 6.46907912 | 5.65E-07 | 0.00027993 |
| *G6PC3* | 3.63021197 | 5.16543319 | 1.79E-06 | 0.00069399 |
| *RUNX2* | 3.36069328 | 6.9213575 | 3.40E-06 | 0.00116051 |
| *ENSGALG00000035956* | 3.31507609 | 2.65380725 | 2.97E-05 | 0.00592059 |
| *ENSGALG00000032465* | 3.19385716 | 5.3790424 | 6.55E-06 | 0.00188256 |
| *ENSGALG00000041603* | 3.19246502 | 8.43957898 | 1.33E-05 | 0.00309532 |
| *BEND6* | 3.18450639 | 4.21753642 | 3.85E-05 | 0.00675082 |
| *MT-ATP6* | 3.17440554 | 13.0986567 | 1.14E-05 | 0.00281981 |
| *MT-CO2* | 3.03956414 | 14.0212698 | 3.81E-05 | 0.00675082 |
| *PTH1R* | 3.01948384 | 7.63030658 | 1.32E-05 | 0.00309532 |
| *MAP3K5* | 2.92187004 | 8.92324446 | 3.63E-07 | 0.00021788 |
| *MT-CO3* | 2.91858082 | 14.3824571 | 2.16E-05 | 0.00465563 |
| *SFMBT2* | 2.84839417 | 5.79051853 | 1.20E-07 | 9.12E-05 |
| *DLX5* | 2.84603432 | 6.50186103 | 4.19E-05 | 0.00716715 |
| *MT-CO1* | 2.80870207 | 13.6394448 | 4.98E-05 | 0.00809204 |
| *ENSGALG00000036956* | 2.79442794 | 11.4552725 | 1.66E-07 | 0.00012207 |
| *ENSGALG00000037976* | 2.77711053 | 3.92952326 | 3.27E-05 | 0.00616503 |
| *PPP1R17* | 2.76307395 | 8.2649359 | 6.01E-05 | 0.00909633 |
| *LRRC49* | 2.73120371 | 4.9732767 | 1.27E-05 | 0.0030353 |
| *GPR146* | 2.58547641 | 5.11835166 | 3.81E-05 | 0.00675082 |
| *FBXW7* | 2.52344672 | 7.15038498 | 5.72E-08 | 5.34E-05 |
| *ENSGALG00000042835* | 2.48773006 | 6.56308166 | 5.94E-06 | 0.00175654 |
| *ENSGALG00000012556* | 2.45275714 | 8.6603745 | 1.37E-06 | 0.00054683 |
| *MAP9* | 2.37088478 | 6.70224091 | 5.88E-06 | 0.00175654 |
| *PRRC2C* | 2.30819849 | 9.1820652 | 1.35E-06 | 0.00054683 |
| *CHD7* | 2.30344462 | 7.38398802 | 3.34E-07 | 0.00021589 |
| *AKT3* | 2.2962147 | 4.95603331 | 4.74E-06 | 0.00153876 |
| *LMO7* | 2.26639683 | 6.96299326 | 4.98E-05 | 0.00809204 |
| *SYNE2* | 2.18458852 | 8.50930084 | 4.59E-07 | 0.00023963 |
| *ANK3* | 2.13073825 | 6.55517413 | 1.84E-06 | 0.00070072 |
| *RAB3GAP1* | 2.09984568 | 6.29838453 | 1.01E-05 | 0.00264321 |
| *NOP53* | 2.06804683 | 6.6939995 | 3.11E-05 | 0.00592059 |
| *WSB1* | 2.01202573 | 10.0159965 | 3.92E-05 | 0.00675082 |

**Supplementary table S5.** Upregulated genes in combined limb + head IMM compared to limb + head MAT

| Gene names | logFC | logCPM | PValue | p.adj |
|---|---|---|---|---|
| *NRIP3* | 9.60546494 | 6.20979508 | 7.35E-10 | 3.94E-07 |
| *CLEC18A* | 8.52662964 | 2.79844423 | 1.46E-16 | 5.80E-13 |
| *NOV* | 7.38001739 | 8.41860561 | 7.34E-09 | 2.41E-06 |
| *ENSGALG00000014501* | 6.63367389 | 8.69601754 | 4.14E-17 | 2.49E-13 |
| *PTN* | 5.3871395 | 10.8053611 | 5.02E-17 | 2.49E-13 |
| *NPAS2* | 5.1205556 | 4.02462162 | 2.86E-12 | 3.99E-09 |
| *SCUBE2* | 5.07374326 | 6.11042398 | 2.37E-10 | 1.62E-07 |
| *MATN3* | 4.95381933 | 4.92413836 | 1.02E-09 | 5.16E-07 |
| *ENSGALG00000029942* | 4.74409239 | 2.33146058 | 3.61E-06 | 0.00040634 |
| *C1QTNF3* | 4.12804636 | 2.12985104 | 3.04E-05 | 0.00212322 |
| *CYTL1* | 4.03232286 | 9.23844841 | 7.06E-12 | 8.24E-09 |
| *FMOD* | 3.92796619 | 4.18911229 | 1.21E-07 | 2.65E-05 |
| *GPR37L1* | 3.92187636 | 3.42851332 | 2.38E-07 | 4.29E-05 |
| *KCNB2* | 3.88411528 | 2.43422849 | 1.83E-05 | 0.00146014 |
| *ASB9* | 3.8761523 | 6.88727327 | 1.28E-15 | 4.23E-12 |
| *COL16A1* | 3.81078419 | 4.95753694 | 7.18E-13 | 1.23E-09 |
| *TNC* | 3.78093491 | 8.13425502 | 1.39E-12 | 2.13E-09 |
| *SLC13A3* | 3.65529441 | 3.83618424 | 1.37E-08 | 4.17E-06 |
| *ENSGALG00000032611* | 3.63270342 | 4.35511964 | 2.01E-11 | 2.09E-08 |
| *DDR2* | 3.63269434 | 4.91444819 | 5.32E-09 | 1.85E-06 |
| *RLBP1* | 3.53293038 | 6.17908244 | 2.05E-08 | 5.81E-06 |
| *ENSGALG00000010003* | 3.52404911 | 4.09497046 | 2.54E-06 | 0.00030892 |
| *ENSGALG00000029601* | 3.51041989 | 6.58530428 | 2.28E-08 | 6.19E-06 |
| *NOG* | 3.44602605 | 4.94314631 | 1.64E-09 | 7.40E-07 |
| *ENSGALG00000046192* | 3.39629916 | 1.85760293 | 3.86E-05 | 0.00254126 |
| *FAM84A* | 3.32853617 | 4.35636704 | 1.83E-06 | 0.00022942 |
| *ENSGALG00000005790* | 3.32849959 | 4.80778005 | 1.64E-08 | 4.78E-06 |
| *ENSGALG00000038134* | 3.29812156 | 3.03415656 | 3.67E-07 | 5.97E-05 |
| *ENSGALG00000033971* | 3.27277478 | 2.87114488 | 3.28E-07 | 5.42E-05 |
| *CSPG5* | 3.27175332 | 2.04036054 | 5.95E-05 | 0.00356178 |
| *ALOX5AP* | 3.24829629 | 3.74531747 | 2.02E-05 | 0.00155166 |
| *SORBS2* | 3.14739156 | 4.98069743 | 8.96E-09 | 2.86E-06 |
| *ENSGALG00000025241* | 3.12729767 | 3.33709561 | 1.86E-05 | 0.00146594 |
| *ENSGALG00000044619* | 3.10545068 | 3.1279005 | 1.07E-07 | 2.43E-05 |
| *FBLN7* | 3.04572413 | 5.2603911 | 3.16E-09 | 1.28E-06 |
| *ARHGEF28* | 2.90036148 | 2.57906941 | 4.58E-05 | 0.00291735 |
| *EFHD2* | 2.88765097 | 5.07223061 | 7.44E-13 | 1.23E-09 |

| | | | | |
|---|---|---|---|---|
| *TSPAN15* | 2.8634615 | 4.90817675 | 3.24E-07 | 5.39E-05 |
| *TRABD2B* | 2.85891241 | 3.22036011 | 1.63E-06 | 0.00021599 |
| *SRPX2* | 2.85399296 | 2.90138832 | 0.00013287 | 0.00662429 |
| *ENSGALG00000034648* | 2.82586957 | 4.12066763 | 5.98E-08 | 1.41E-05 |
| *CDT1* | 2.82165319 | 2.6783515 | 5.93E-06 | 0.00059952 |
| *PLEKHG5* | 2.81302618 | 4.87390699 | 0.00022542 | 0.00979915 |
| *ENSGALG00000011962* | 2.81278694 | 5.26109353 | 1.61E-05 | 0.00136155 |
| *ENSGALG00000032990* | 2.77412681 | 4.42154432 | 7.41E-09 | 2.41E-06 |
| *ENSGALG00000003446* | 2.76741968 | 5.41775923 | 6.86E-11 | 5.67E-08 |
| *GLI2* | 2.72911264 | 5.90476439 | 1.80E-07 | 3.53E-05 |
| *ADCY2* | 2.71365218 | 5.34450388 | 2.62E-05 | 0.00190596 |
| *LOXL3* | 2.69784185 | 2.25141931 | 0.000133 | 0.00662429 |
| *KCNA4* | 2.69569557 | 3.65456097 | 8.04E-05 | 0.00447883 |
| *P4HA3* | 2.69103911 | 7.25813399 | 1.09E-09 | 5.27E-07 |
| *RIMS2* | 2.69032687 | 4.51516585 | 6.62E-05 | 0.00386161 |
| *CTHRC1* | 2.68900149 | 8.04570551 | 1.07E-10 | 8.08E-08 |
| *HSD17B7* | 2.68718084 | 6.10232778 | 1.80E-07 | 3.53E-05 |
| *CACNG5* | 2.64308267 | 2.75334595 | 1.94E-06 | 0.00024071 |
| *NET1* | 2.58445938 | 7.76955463 | 2.65E-05 | 0.00191617 |
| *CHGB* | 2.57552074 | 5.95041907 | 4.26E-09 | 1.59E-06 |
| *ORC1* | 2.55330523 | 4.19224592 | 1.22E-07 | 2.65E-05 |
| *ENSGALG00000032444* | 2.54768083 | 6.57525459 | 1.39E-07 | 2.96E-05 |
| *CCDC102A* | 2.54073441 | 2.75866061 | 7.04E-06 | 0.000691 |
| *GAS2L3* | 2.53816178 | 3.39804541 | 0.00011281 | 0.00588502 |
| *CSF1* | 2.52556199 | 2.0494054 | 3.62E-05 | 0.00242313 |
| *STK10* | 2.52140478 | 5.51542629 | 1.58E-08 | 4.74E-06 |
| *SRPX* | 2.50663991 | 7.42775011 | 1.71E-09 | 7.53E-07 |
| *NPTX2* | 2.50228408 | 4.06546858 | 1.64E-08 | 4.78E-06 |
| *E2F1* | 2.48957475 | 5.86878479 | 1.81E-08 | 5.20E-06 |
| *ENSGALG00000034155* | 2.48837542 | 2.83032521 | 1.23E-06 | 0.00016788 |
| *ENSGALG00000010741* | 2.46485294 | 5.96719353 | 2.12E-07 | 3.96E-05 |
| *LRAT* | 2.42795925 | 2.67601682 | 0.0001013 | 0.00536909 |
| *ENSGALG00000043332* | 2.40761406 | 4.82638333 | 1.53E-05 | 0.00130532 |
| *SLC4A7* | 2.40407835 | 8.46373422 | 2.01E-07 | 3.79E-05 |
| *ENSGALG00000006658* | 2.40312177 | 4.96382939 | 2.98E-07 | 5.09E-05 |
| *KHDRBS2* | 2.39692034 | 3.09671269 | 0.00014054 | 0.00689603 |
| *THBS1* | 2.3965438 | 9.16479695 | 7.20E-06 | 0.00070277 |
| *NCAPG2* | 2.39464796 | 5.02333748 | 9.17E-05 | 0.00497952 |
| *ENSGALG00000039008* | 2.38474986 | 2.47775721 | 0.00019542 | 0.00878404 |
| *CDCA7* | 2.3777261 | 6.90446577 | 2.93E-08 | 7.54E-06 |

| | | | | |
|---|---|---|---|---|
| *ENSGALG00000016636* | 2.33168306 | 7.64227337 | 1.74E-05 | 0.00142291 |
| *RASL11B* | 2.31738742 | 6.18418718 | 3.69E-05 | 0.00245207 |
| *NEK2* | 2.30380812 | 4.9340743 | 1.86E-06 | 0.00023153 |
| *CHAF1B* | 2.30204253 | 5.21979631 | 5.40E-06 | 0.00055136 |
| *SHROOM4* | 2.30182085 | 5.3775527 | 6.51E-07 | 9.78E-05 |
| *MMP16* | 2.29728565 | 6.31309034 | 6.76E-11 | 5.67E-08 |
| *PRODH* | 2.29032874 | 4.93939205 | 1.83E-06 | 0.00022942 |
| *FANCA* | 2.28542412 | 3.16764606 | 7.87E-06 | 0.00075715 |
| *WDR76* | 2.26488612 | 3.6938464 | 8.53E-06 | 0.00080489 |
| *ENSGALG00000036572* | 2.24650241 | 2.80261647 | 3.04E-05 | 0.00212322 |
| *ENSGALG00000013420* | 2.23696199 | 2.69233312 | 6.51E-05 | 0.00383004 |
| *ENSGALG00000023193* | 2.22173386 | 5.06144562 | 1.84E-07 | 3.57E-05 |
| *SDC3* | 2.20485258 | 6.37844839 | 1.16E-06 | 0.00016014 |
| *MCM2* | 2.18884095 | 6.38042948 | 2.35E-07 | 4.28E-05 |
| *THBS4* | 2.18880631 | 6.63826282 | 9.05E-07 | 0.00012903 |
| *ENSGALG00000036847* | 2.17647068 | 7.23383091 | 6.21E-09 | 2.12E-06 |
| *ENPP2* | 2.17129704 | 6.06432887 | 5.59E-10 | 3.17E-07 |
| *NLGN3* | 2.13473861 | 2.28263173 | 0.00012209 | 0.00627014 |
| *UBE2C* | 2.128121 | 5.3916446 | 7.40E-05 | 0.00420403 |
| *ENSGALG00000034631* | 2.12473458 | 3.99025345 | 6.32E-06 | 0.0006325 |
| *TK1* | 2.10632277 | 6.37111304 | 1.83E-05 | 0.00146014 |
| *GLI3* | 2.0904505 | 5.65018842 | 3.70E-08 | 9.16E-06 |
| *KIF2C* | 2.09012299 | 5.52697011 | 4.19E-05 | 0.00271953 |
| *CIT* | 2.07502478 | 4.35618057 | 1.94E-05 | 0.00150133 |
| *BLM* | 2.07176694 | 3.8634239 | 5.18E-06 | 0.00054029 |
| *BRCA1* | 2.06865653 | 5.98964536 | 6.56E-05 | 0.00384623 |
| *RAD54L* | 2.06461985 | 4.11134799 | 5.29E-06 | 0.00054713 |
| *MCM5* | 2.06059267 | 5.897243 | 6.69E-08 | 1.56E-05 |
| *ATP10A* | 2.05955023 | 5.0201078 | 0.00019938 | 0.00889454 |
| *PIF1* | 2.0460181 | 2.68892571 | 0.00016063 | 0.00762675 |
| *ENSGALG00000037711* | 2.04340442 | 6.57972664 | 9.37E-06 | 0.00087649 |
| *GHRL* | 2.03041333 | 4.93515672 | 9.66E-05 | 0.00518598 |
| *ENSGALG00000032177* | 2.0209414 | 3.54888312 | 4.92E-05 | 0.00310894 |
| *ENSGALG00000038194* | 2.01716723 | 6.23776387 | 8.60E-07 | 0.00012357 |
| *ASPM* | 2.01551805 | 7.20543591 | 5.01E-05 | 0.00314237 |
| *FANCD2* | 2.01458618 | 3.31672827 | 0.00017869 | 0.00823743 |
| *PHGDH* | 2.00236801 | 6.11570354 | 1.44E-07 | 3.03E-05 |
| *ESPL1* | 2.00234672 | 3.79303603 | 5.30E-05 | 0.00323748 |

**Supplementary table S6.** Upregulated genes in combined limb + head MAT compared to limb + head IMM

| Gene names | logFC | logCPM | PValue | p_adjusted |
|---|---|---|---|---|
| *SPP1* | 10.21782475 | 11.99026238 | 1.21E-18 | 1.20E-14 |
| *MMP13* | 8.992061783 | 10.12224928 | 4.13E-14 | 9.09E-11 |
| *ENSGALG00000019063* | 8.542267642 | 4.79930242 | 2.24E-10 | 1.58E-07 |
| *IRX6* | 8.270928725 | 5.030262014 | 9.70E-22 | 1.92E-17 |
| *MMP9* | 7.728843011 | 6.405784422 | 2.36E-08 | 6.33E-06 |
| *ENSGALG00000006453* | 7.010058381 | 7.960414992 | 5.56E-10 | 3.17E-07 |
| *IYD* | 6.841069264 | 3.206778259 | 1.15E-09 | 5.43E-07 |
| *LOXL4* | 6.723181896 | 3.803460877 | 1.92E-09 | 8.28E-07 |
| *ENSGALG00000029931* | 6.537287259 | 1.307264556 | 2.92E-07 | 5.03E-05 |
| *COL10A1* | 6.452015301 | 6.128278184 | 1.04E-09 | 5.16E-07 |
| *ENSGALG00000046293* | 6.387884202 | 2.606194912 | 6.84E-10 | 3.77E-07 |
| *UPK1B* | 5.702323203 | 3.058153226 | 1.21E-08 | 3.76E-06 |
| *ENSGALG00000044318* | 5.270620483 | 4.815458838 | 7.16E-11 | 5.68E-08 |
| *SERPINB2* | 5.113200586 | 4.393903776 | 1.25E-05 | 0.001124839 |
| *IRX4* | 5.109156924 | 3.200563989 | 4.56E-05 | 0.00291735 |
| *IBSP* | 5.066086758 | 9.80899404 | 6.52E-13 | 1.23E-09 |
| *CLDN1* | 5.006667164 | 3.560272547 | 2.14E-05 | 0.001621908 |
| *ENSGALG00000043071* | 4.82518386 | 7.532681946 | 3.42E-06 | 0.000391748 |
| *ENSGALG00000010927* | 4.741838792 | 7.340699122 | 3.43E-10 | 2.13E-07 |
| *ENSGALG00000044656* | 4.734327925 | 2.04111052 | 2.63E-06 | 0.000317306 |
| *SLC22A16* | 4.719794335 | 5.787853973 | 1.44E-11 | 1.58E-08 |
| *HS3ST1* | 4.62270135 | 2.638955579 | 2.65E-06 | 0.00031862 |
| *ENSGALG00000000136* | 4.473807588 | 1.635663661 | 0.000164782 | 0.007795896 |
| *FGF9* | 4.407414522 | 1.939984129 | 9.10E-05 | 0.004953737 |
| *SRRM4* | 4.394839072 | 1.259427797 | 2.81E-06 | 0.000331943 |
| *FGF13* | 4.329887842 | 3.573470087 | 1.42E-05 | 0.001237269 |
| *SCIN* | 4.238521768 | 9.587394184 | 5.26E-12 | 6.52E-09 |
| *CD200* | 4.225762094 | 6.120520298 | 2.53E-08 | 6.69E-06 |
| *IFITM5* | 4.168192823 | 8.562355612 | 2.44E-11 | 2.42E-08 |
| *BST1* | 4.129181326 | 3.707605817 | 2.32E-05 | 0.001732147 |
| *INSRR* | 4.082272667 | 1.901874371 | 5.36E-07 | 8.31E-05 |
| *HS3ST5* | 4.066420901 | 1.791412152 | 3.02E-05 | 0.002123216 |
| *ATP2B2* | 4.049190929 | 6.885187438 | 1.41E-09 | 6.52E-07 |
| *ENSGALG00000035438* | 4.039114149 | 3.306336815 | 0.000136071 | 0.006726508 |
| *NRG1* | 4.016848862 | 4.844316792 | 2.57E-05 | 0.001879482 |
| *UTS2B* | 4.008116873 | 2.014121249 | 1.49E-05 | 0.00127241 |
| *SLC43A2* | 4.001282047 | 3.102148747 | 3.73E-06 | 0.00041579 |

| | | | | |
|---|---|---|---|---|
| *PDK4* | 3.988708728 | 5.170019175 | 3.23E-07 | 5.39E-05 |
| *SLC38A4* | 3.960219306 | 4.203955161 | 0.000209929 | 0.00930623 |
| *SLC6A2* | 3.921544725 | 6.469476633 | 5.26E-15 | 1.49E-11 |
| *FAS* | 3.853000618 | 3.769789754 | 4.03E-09 | 1.54E-06 |
| *ARHGDIG* | 3.848457122 | 1.585292524 | 4.00E-05 | 0.002624035 |
| *ENSGALG00000036780* | 3.815519375 | 4.753711207 | 5.82E-05 | 0.003498547 |
| *ISL2* | 3.811555094 | 2.632812996 | 3.16E-05 | 0.002191252 |
| *ENSGALG00000040306* | 3.797165937 | 9.099036327 | 3.98E-06 | 0.000429001 |
| *MST1R* | 3.742402118 | 1.63028263 | 7.55E-05 | 0.00426253 |
| *ENSGALG00000045039* | 3.737249662 | 1.619056957 | 2.81E-06 | 0.000331943 |
| *ENSGALG00000004436* | 3.727959787 | 2.2159199 | 1.38E-06 | 0.000186087 |
| *ENSGALG00000006608* | 3.70522827 | 5.55048867 | 1.10E-10 | 8.08E-08 |
| *ENSGALG00000038056* | 3.676217619 | 3.912614502 | 2.79E-08 | 7.27E-06 |
| *SYT8* | 3.612001134 | 2.832029451 | 3.07E-07 | 5.20E-05 |
| *ANO6* | 3.579124574 | 5.721462252 | 1.17E-05 | 0.001068194 |
| *MEF2C* | 3.548347465 | 8.203876164 | 4.21E-05 | 0.002719529 |
| *SYNPR* | 3.540398583 | 2.543810195 | 3.09E-05 | 0.002152579 |
| *CAPNS2* | 3.521606639 | 3.33716241 | 4.62E-09 | 1.67E-06 |
| *LY75* | 3.50983487 | 5.917032118 | 5.80E-11 | 5.22E-08 |
| *CNKSR3* | 3.499917955 | 3.722635915 | 3.90E-07 | 6.29E-05 |
| *RGCC* | 3.49024418 | 5.274813792 | 3.20E-08 | 8.14E-06 |
| *FGF1* | 3.412226812 | 2.082989389 | 5.36E-06 | 0.000550997 |
| *PTGS2* | 3.396694459 | 3.674132425 | 6.53E-06 | 0.000647402 |
| *SATB1* | 3.382549373 | 5.841978105 | 2.64E-14 | 6.53E-11 |
| *ARHGAP45* | 3.381364736 | 4.404308817 | 1.48E-07 | 3.09E-05 |
| *VDR* | 3.377888259 | 2.015114698 | 3.87E-06 | 0.00042118 |
| *ENSGALG00000045584* | 3.362571334 | 5.28915694 | 6.99E-09 | 2.35E-06 |
| *TBX2* | 3.342329472 | 5.296026137 | 1.65E-06 | 0.000216368 |
| *MYO6* | 3.315380731 | 8.178990008 | 4.88E-09 | 1.73E-06 |
| *JAKMIP1* | 3.291868137 | 4.643763604 | 4.88E-05 | 0.003090356 |
| *SUSD3* | 3.262171401 | 3.291630824 | 5.30E-06 | 0.000547129 |
| *4930523C07RIK* | 3.248231002 | 1.420204369 | 2.97E-05 | 0.002103821 |
| *PCOLCE2* | 3.234230684 | 4.046735489 | 8.44E-07 | 0.000122163 |
| *ENSGALG00000021399* | 3.225410897 | 4.380007805 | 2.89E-05 | 0.002062969 |
| *ABLIM2* | 3.209868423 | 3.833090025 | 0.000222962 | 0.009713791 |
| *PLA2R1* | 3.207183728 | 7.113943079 | 4.48E-09 | 1.65E-06 |
| *BHLHE40* | 3.158396426 | 5.916166666 | 3.42E-10 | 2.13E-07 |
| *DLX6* | 3.125563216 | 2.185925676 | 5.22E-05 | 0.003200812 |
| *MAP3K5* | 3.120174835 | 8.923283337 | 3.41E-10 | 2.13E-07 |
| *SLC38A2* | 3.086968722 | 8.902101249 | 8.32E-10 | 4.34E-07 |

| | | | | |
|---|---|---|---|---|
| *HPS1* | 3.080070266 | 5.014859504 | 3.98E-10 | 2.39E-07 |
| *SLC38A1* | 3.047268414 | 5.503527411 | 3.90E-08 | 9.53E-06 |
| *WNT5B* | 3.014101006 | 2.613247576 | 7.71E-07 | 0.00011324 |
| *P3H2* | 3.00977764 | 4.023548236 | 3.82E-06 | 0.000420799 |
| *ASTN2* | 3.008989021 | 1.672270699 | 0.000189233 | 0.008623365 |
| *DUSP26* | 3.007866579 | 2.459697894 | 3.76E-06 | 0.00041648 |
| *NRAP* | 2.987955573 | 5.513681948 | 0.000186114 | 0.008500799 |
| *ITSN2* | 2.959940747 | 5.869468501 | 3.02E-12 | 3.99E-09 |
| *TRIM36* | 2.947300015 | 3.054181025 | 3.45E-06 | 0.000391748 |
| *ENSGALG00000038740* | 2.926396939 | 1.992486461 | 4.08E-05 | 0.00267071 |
| *STEAP1* | 2.920695814 | 1.87315377 | 3.18E-05 | 0.002194756 |
| *SMPD3* | 2.880417333 | 4.941423549 | 0.000106841 | 0.005617796 |
| *ENSGALG00000041228* | 2.875227885 | 6.417363833 | 1.73E-05 | 0.001422906 |
| *PHLDB2* | 2.861627319 | 3.75639545 | 3.98E-09 | 1.54E-06 |
| *ENSGALG00000038238* | 2.834746134 | 5.041707751 | 1.36E-05 | 0.001186781 |
| *BAMBI* | 2.809007003 | 8.107384074 | 2.28E-06 | 0.000279078 |
| *ENSGALG00000031844* | 2.769353142 | 4.330496685 | 1.14E-08 | 3.58E-06 |
| *GAB1* | 2.76080613 | 7.769487742 | 1.13E-07 | 2.54E-05 |
| *ENPP6* | 2.756899674 | 3.625635684 | 1.90E-05 | 0.00148347 |
| *CBFA2T3* | 2.698903313 | 3.370556564 | 0.000131017 | 0.00659175 |
| *ENSGALG00000031177* | 2.695150662 | 3.552248563 | 1.78E-06 | 0.000227502 |
| *ANK* | 2.6851528 | 6.091205011 | 6.13E-07 | 9.40E-05 |
| *ENSGALG00000043269* | 2.659539969 | 2.377741572 | 0.000126444 | 0.00637785 |
| *DYNC1I1* | 2.657386988 | 4.096096075 | 3.45E-08 | 8.66E-06 |
| *RGS2* | 2.654790646 | 5.478202945 | 3.63E-06 | 0.000406337 |
| *FAM20C* | 2.648912224 | 5.109751424 | 4.80E-06 | 0.000506251 |
| *ENSGALG00000039821* | 2.643232642 | 2.943102752 | 2.06E-05 | 0.001570111 |
| *OXSR1* | 2.633085095 | 3.526902706 | 8.00E-05 | 0.004478829 |
| *NHEJ1* | 2.631913316 | 4.151434241 | 1.88E-05 | 0.001479578 |
| *PLCL2* | 2.620502122 | 3.261003116 | 1.59E-06 | 0.000213579 |
| *ENSGALG00000013655* | 2.598263525 | 2.876752835 | 5.53E-05 | 0.003339977 |
| *COL22A1* | 2.586565754 | 7.983822763 | 6.16E-07 | 9.40E-05 |
| *BMF* | 2.582027699 | 5.267881218 | 0.000135833 | 0.006726508 |
| *F13A1* | 2.581170789 | 3.501427306 | 4.49E-07 | 7.02E-05 |
| *PCMTD1* | 2.569289996 | 6.367775697 | 2.62E-07 | 4.55E-05 |
| *CITED4* | 2.560749104 | 4.340466679 | 2.27E-06 | 0.000279078 |
| *ENSGALG00000043750* | 2.554249751 | 3.510791128 | 1.63E-05 | 0.001370604 |
| *ENSGALG00000020626* | 2.541637862 | 5.795815394 | 1.39E-07 | 2.96E-05 |
| *ENSGALG00000032186* | 2.538580194 | 2.463414639 | 1.79E-05 | 0.001448315 |
| *SLC13A5* | 2.527136229 | 6.045683652 | 3.98E-06 | 0.000429001 |

| | | | | |
|---|---|---|---|---|
| *PPP1R16B* | 2.519023135 | 2.987335827 | 0.000122509 | 0.006275207 |
| *MOXD1* | 2.515431587 | 4.114552944 | 9.91E-05 | 0.005297248 |
| *NPR3* | 2.510480038 | 3.900416199 | 9.07E-05 | 0.004950664 |
| *EPB41* | 2.499825982 | 4.337818196 | 7.25E-05 | 0.004153806 |
| *ENSGALG00000041329* | 2.471137335 | 2.000461402 | 0.000133339 | 0.006624527 |
| *TBC1D4* | 2.452706931 | 5.318919077 | 5.64E-05 | 0.003400328 |
| *TMC1* | 2.451998023 | 3.580270259 | 1.84E-05 | 0.001460143 |
| *ENSGALG00000038748* | 2.433387963 | 6.405982698 | 1.79E-05 | 0.001448315 |
| *PCYOX1* | 2.430595524 | 6.019964225 | 8.07E-05 | 0.004478829 |
| *ARID5B* | 2.418698126 | 5.150578234 | 1.74E-06 | 0.000225838 |
| *RSAD2* | 2.410186674 | 3.098194597 | 1.04E-05 | 0.000954715 |
| *PDGFA* | 2.409565335 | 5.594863877 | 1.65E-05 | 0.001374599 |
| *CKB* | 2.409037361 | 9.060998502 | 9.31E-07 | 0.000131872 |
| *FBXO31* | 2.408639628 | 2.460351384 | 0.00015938 | 0.007594678 |
| *CYTH1* | 2.384473998 | 4.915736924 | 3.16E-09 | 1.28E-06 |
| *CPM* | 2.365838075 | 5.043463939 | 4.44E-06 | 0.000473292 |
| *G6PC3* | 2.360233192 | 5.166967843 | 3.46E-06 | 0.000391748 |
| *VTI1A* | 2.346620209 | 5.924123458 | 7.41E-07 | 0.000109692 |
| *1810011O10RIK* | 2.32676554 | 3.84260295 | 0.000126371 | 0.00637785 |
| *OSBPL5* | 2.304135471 | 3.37085 | 3.68E-05 | 0.00245207 |
| *AIFM2* | 2.294175692 | 5.920386118 | 1.77E-06 | 0.000227502 |
| *MITF* | 2.28826433 | 4.845260992 | 6.94E-05 | 0.004018215 |
| *MYH11* | 2.276047367 | 5.255706828 | 3.49E-05 | 0.002347454 |
| *ANKS1B* | 2.268609273 | 4.562864978 | 0.000126128 | 0.00637785 |
| *TGM2* | 2.241085378 | 5.262035319 | 7.94E-06 | 0.000760169 |
| *HIP1* | 2.213117287 | 4.624070455 | 3.14E-06 | 0.000364576 |
| *TIMP3* | 2.196700686 | 6.914364983 | 9.24E-05 | 0.005005375 |
| *PPDPF* | 2.193007058 | 5.688161631 | 3.31E-05 | 0.002244881 |
| *ENSGALG00000031365* | 2.191378128 | 4.36170377 | 3.14E-06 | 0.000364576 |
| *ENSGALG00000034967* | 2.190890731 | 5.78028372 | 2.63E-09 | 1.11E-06 |
| *HPCAL1* | 2.185500247 | 6.935972108 | 2.87E-05 | 0.002053184 |
| *ENSGALG00000035956* | 2.15291724 | 2.65682237 | 6.97E-05 | 0.004018215 |
| *ENSGALG00000030635* | 2.109694738 | 6.698136272 | 9.11E-08 | 2.10E-05 |
| *PPP1R17* | 2.100237308 | 8.264998348 | 4.17E-05 | 0.002716308 |
| *SGK1* | 2.08045003 | 6.270987814 | 1.52E-07 | 3.15E-05 |
| *ALCAM* | 2.062562049 | 6.446366758 | 6.97E-05 | 0.004018215 |
| *TCP11L2* | 2.04624055 | 7.222264785 | 1.98E-07 | 3.77E-05 |
| *FSTL4* | 2.039346676 | 6.556003514 | 4.67E-05 | 0.002967274 |
| *ENSGALG00000015019* | 2.037056207 | 4.1605551 | 4.38E-06 | 0.000469384 |
| *LMO7* | 2.026217723 | 6.96313979 | 5.69E-08 | 1.36E-05 |

| | | | | |
|---|---|---|---|---|
| *TCF7L2* | 2.014618233 | 9.872227986 | 2.22E-07 | 4.10E-05 |

**Supplementary table S7.** Upregulated genes in limb IMM compared to head IMM

| Gene name | logFC | logCPM | PValue | p.adj |
|---|---|---|---|---|
| *HOXA10* | 9.80660058 | 1.8977002 | 2.03E-11 | 9.89E-09 |
| *NRIP3* | 9.56934225 | 6.16910351 | 1.32E-21 | 6.24E-18 |
| *ENSGALG00000039629* | 9.49425249 | 6.25196417 | 9.32E-08 | 1.17E-05 |
| *ENSGALG00000028983* | 9.15186054 | 7.88868494 | 1.59E-08 | 2.71E-06 |
| *HOXA5* | 8.96497782 | 7.64637016 | 6.09E-30 | 8.62E-26 |
| *HOXD10* | 8.62110972 | 3.36141004 | 1.68E-12 | 1.40E-09 |
| *TBX5* | 7.60321692 | 5.75443885 | 3.12E-12 | 2.16E-09 |
| *HOXA7* | 7.45173646 | 5.79185713 | 1.17E-17 | 3.32E-14 |
| *ENSGALG00000022622* | 7.28055551 | 6.49751212 | 1.21E-23 | 8.57E-20 |
| *ENSGALG00000038539* | 6.91078564 | -0.0541158 | 0.00206425 | 0.02110058 |
| *HOXA6* | 6.87958908 | 2.37313726 | 1.33E-12 | 1.17E-09 |
| *ENSGALG00000037173* | 6.71031266 | 2.08370258 | 3.07E-05 | 0.00110473 |
| *ENSGALG00000039600* | 6.65565307 | -0.5903113 | 0.00112705 | 0.01439796 |
| *TTYH2* | 6.60812589 | 0.11404068 | 0.00393149 | 0.03232454 |
| *ENSGALG00000044057* | 6.58803745 | 1.99205081 | 4.13E-05 | 0.00136573 |
| *ENSGALG00000046647* | 6.417954 | 0.46842933 | 0.00249167 | 0.02388189 |
| *ENSGALG00000044866* | 5.78037482 | 5.36384512 | 1.45E-09 | 3.88E-07 |
| *LHX9* | 5.57788616 | 0.46606927 | 0.0045434 | 0.03564368 |
| *HOXD8* | 5.1528461 | 4.08651713 | 4.31E-14 | 5.08E-11 |
| *ENSGALG00000029104* | 5.13897503 | 0.50247432 | 7.79E-05 | 0.00223985 |
| *ENSGALG00000032375* | 4.92959015 | -0.8570711 | 0.00240583 | 0.02340069 |
| *PTK2B* | 4.90760702 | 0.16927637 | 0.00139 | 0.01658268 |
| *ENSGALG00000023419* | 4.89214514 | 4.75084462 | 9.68E-19 | 3.42E-15 |
| *ENSGALG00000039577* | 4.87771628 | 0.6801747 | 0.0003058 | 0.00576829 |
| *ENSGALG00000005648* | 4.84211311 | 1.00842999 | 0.00275834 | 0.02548807 |
| *FAM81A* | 4.79414293 | 1.86569761 | 5.08E-05 | 0.00158555 |
| *HOXD12* | 4.73647663 | 1.80453546 | 0.00481482 | 0.03710039 |
| *ENSGALG00000042590* | 4.73191704 | 0.43582692 | 0.00314204 | 0.02779888 |
| *ENSGALG00000043030* | 4.72020234 | -0.4066629 | 0.00281853 | 0.02590306 |
| *ENSGALG00000036752* | 4.6728605 | 0.19562813 | 0.00186444 | 0.01980195 |
| *HK2* | 4.59651185 | 0.70555326 | 0.00248614 | 0.02384502 |
| *ENSGALG00000000669* | 4.37061939 | 0.70013429 | 0.00116142 | 0.01465705 |
| *ENSGALG00000044917* | 4.33639498 | 1.52971889 | 0.00344378 | 0.02943758 |
| *NME9* | 4.29101309 | -0.2057339 | 0.00131916 | 0.01602352 |

| | | | | |
|---|---|---|---|---|
| *ENSGALG00000018331* | 4.26273571 | 2.41884607 | 2.12E-05 | 0.00083044 |
| *ENSGALG00000045117* | 4.24984543 | -0.4530853 | 0.00229108 | 0.02261823 |
| *ENSGALG00000029942* | 4.21757238 | 2.29110478 | 0.00112323 | 0.01439338 |
| *ADAMTSL5* | 4.21202494 | 1.84970831 | 6.89E-05 | 0.00201854 |
| *PARP6* | 4.20255763 | 0.6544748 | 0.00022965 | 0.0047847 |
| *ENSGALG00000043606* | 4.18496436 | 0.63197501 | 0.0073082 | 0.04825248 |
| *FAM84A* | 4.15807074 | 4.32927237 | 3.43E-07 | 3.28E-05 |
| *SCUBE2* | 4.15295196 | 6.0777413 | 8.17E-06 | 0.00039981 |
| *PLEKHG5* | 4.13481043 | 4.85370028 | 5.35E-07 | 4.51E-05 |
| *ENSGALG00000045752* | 4.07742729 | 0.42915464 | 0.00323369 | 0.02834384 |
| *ENSGALG00000028659* | 4.04814492 | 1.16703886 | 0.00576199 | 0.04150455 |
| *PHF19* | 4.04473758 | -0.0046779 | 0.00111355 | 0.0143379 |
| *ENSGALG00000035362* | 4.00696161 | -0.4477016 | 0.0018729 | 0.01987689 |
| *ENSGALG00000013253* | 3.98298782 | 1.90786895 | 0.00746853 | 0.04881311 |
| *ENSGALG00000025692* | 3.94698377 | 1.24983854 | 0.00357982 | 0.03023507 |
| *ENSGALG00000044168* | 3.91045575 | 0.35494304 | 0.00583094 | 0.04174608 |
| *SHOX2* | 3.89910237 | -0.2416237 | 0.00213172 | 0.02164482 |
| *ENSGALG00000043544* | 3.78772338 | 0.32152726 | 0.00500554 | 0.03790863 |
| *ENSGALG00000010002* | 3.70997524 | 1.89094549 | 0.00449978 | 0.03548401 |
| *ENSGALG00000025680* | 3.68446831 | 0.08540774 | 0.00183069 | 0.01958571 |
| *ENSGALG00000037284* | 3.66770056 | 0.26117831 | 0.00137337 | 0.01647852 |
| *ENSGALG00000035579* | 3.64388 | 4.68759561 | 1.56E-06 | 0.00010346 |
| *ENSGALG00000033342* | 3.62260697 | -0.3251187 | 0.00321649 | 0.0282456 |
| *0610009O20RIK* | 3.60536663 | 0.4434747 | 0.0051105 | 0.03845653 |
| *ENSGALG00000032731* | 3.60467547 | 1.12366024 | 0.00075296 | 0.01092531 |
| *WNK4* | 3.57576295 | 2.41006697 | 0.00019127 | 0.00419514 |
| *ENSGALG00000030781* | 3.56522784 | 7.41029901 | 6.13E-05 | 0.00182119 |
| *B4GALNT4* | 3.51406565 | 2.38319674 | 0.00102772 | 0.0135374 |
| *NPR2* | 3.50997171 | 1.3111115 | 0.00098822 | 0.01326885 |
| *ENSGALG00000041527* | 3.44140076 | -0.1277481 | 0.00488675 | 0.03745004 |
| *PLPPR1* | 3.41692685 | 6.71303389 | 2.83E-06 | 0.00016802 |
| *ENSGALG00000043133* | 3.35758191 | 1.53926996 | 0.00165336 | 0.01847555 |
| *CUTAL* | 3.34163196 | 4.65336142 | 1.62E-05 | 0.00068892 |
| *ENSGALG00000039810* | 3.34059056 | 0.86380815 | 0.00202475 | 0.02086247 |
| *ENSGALG00000029182* | 3.30795605 | 4.95638951 | 5.16E-05 | 0.00160132 |
| *ENSGALG00000034639* | 3.29947881 | 2.27959811 | 0.00090006 | 0.01233832 |
| *SCARF2* | 3.29016968 | 0.9551968 | 0.00496189 | 0.03771939 |
| *ENSGALG00000034738* | 3.28819056 | 3.37262435 | 1.53E-06 | 0.00010332 |
| *MEX3A* | 3.25920222 | 0.04615716 | 0.00565249 | 0.04102916 |
| *PIK3R2* | 3.2032547 | 0.5041175 | 0.00671919 | 0.04581779 |

| | | | | |
|---|---|---|---|---|
| *ENSGALG00000017872* | 3.19763715 | 0.46124335 | 0.00238743 | 0.02333769 |
| *ENSGALG00000010928* | 3.18535698 | 9.79596838 | 0.00408002 | 0.0331724 |
| *RIMS2* | 3.18012171 | 4.4862622 | 0.000247 | 0.00501765 |
| *CHSY3* | 3.17337321 | 0.81686603 | 0.00217104 | 0.02186031 |
| *ENSGALG00000043546* | 3.16953265 | 1.75874341 | 0.00046547 | 0.00782523 |
| *ENSGALG00000024609* | 3.15918881 | 0.83278132 | 0.00570403 | 0.04123399 |
| *SHMT1* | 3.15103922 | 1.93715451 | 0.00082692 | 0.01161707 |
| *DLX5* | 3.11121615 | 6.49467693 | 4.34E-06 | 0.0002402 |
| *ENSGALG00000030723* | 3.11014541 | 2.03618357 | 0.00740176 | 0.04855583 |
| *PICALM* | 3.10246981 | 3.63379976 | 9.45E-05 | 0.00258633 |
| *ENSGALG00000045875* | 3.08554485 | 3.77558249 | 0.00043991 | 0.00753439 |
| *ENSGALG00000038134* | 3.04435738 | 3.00894532 | 3.05E-05 | 0.0010992 |
| *UNC119* | 3.0358582 | 0.60912354 | 0.00462065 | 0.03611508 |
| *ENSGALG00000025241* | 3.03086685 | 3.31724379 | 0.00112623 | 0.01439796 |
| *TMEM74* | 3.01242868 | 4.1999841 | 0.00529222 | 0.03923768 |
| *ENSGALG00000005927* | 2.97498896 | 3.67387554 | 9.73E-06 | 0.00045597 |
| *ALDH6A1* | 2.97261095 | 0.69966411 | 0.00574307 | 0.0414277 |
| *ENSGALG00000038166* | 2.96177454 | 1.54144935 | 0.00150219 | 0.01743354 |
| *NKX3-2* | 2.94978794 | 6.8127778 | 0.00193006 | 0.02018077 |
| *PHLDB1* | 2.94574215 | 2.55139718 | 0.00099331 | 0.01330712 |
| *ERBB3* | 2.93871693 | 4.99148992 | 1.33E-05 | 0.00058658 |
| *PFKL* | 2.93378137 | 4.4422156 | 1.59E-06 | 0.00010475 |
| *FBXO15* | 2.91532946 | 3.7232892 | 0.00475944 | 0.03681341 |
| *9930104L06RIK* | 2.90948795 | 3.0665411 | 4.45E-06 | 0.00024513 |
| *ENSGALG00000034355* | 2.90417385 | 1.22607865 | 0.00299271 | 0.02691535 |
| *OIT3* | 2.8936764 | 3.30832449 | 0.00106983 | 0.01392359 |
| *ENSGALG00000012085* | 2.89127895 | 4.22314666 | 6.06E-05 | 0.001805 |
| *ENSGALG00000036187* | 2.88788999 | 0.21480601 | 0.00656568 | 0.04517737 |
| *ENSGALG00000045672* | 2.88759323 | 5.47649955 | 1.07E-05 | 0.00048885 |
| *ZBTB17* | 2.86187664 | 1.87480168 | 0.00057438 | 0.00905878 |
| *ENSGALG00000035870* | 2.83977827 | 8.32034149 | 4.50E-08 | 6.18E-06 |
| *HSD17B7* | 2.83390693 | 6.08168827 | 1.51E-06 | 0.00010247 |
| *ENSGALG00000040518* | 2.82513918 | 2.75149286 | 0.00168106 | 0.01856517 |
| *ENSGALG00000016636* | 2.81503251 | 7.62283089 | 3.66E-05 | 0.00126179 |
| *MAB21L1* | 2.81423663 | 6.00979801 | 1.86E-08 | 3.10E-06 |
| *TMEM243* | 2.8064393 | 2.73309135 | 0.00031041 | 0.00583951 |
| *FUS* | 2.78357955 | 7.11496211 | 0.00322338 | 0.02828852 |
| *ENSGALG00000042435* | 2.75979289 | 2.64328687 | 0.0013378 | 0.01613793 |
| *ENSGALG00000041409* | 2.75669853 | 2.03453416 | 0.00041457 | 0.00716984 |
| *RNF215* | 2.75662145 | 1.14313997 | 0.00225434 | 0.02236471 |

| | | | | |
|---|---|---|---|---|
| *RBFOX3* | 2.72600059 | 1.90401195 | 0.00290295 | 0.02642726 |
| *FUK* | 2.72355539 | 2.56437345 | 0.00063198 | 0.00974979 |
| *PTN* | 2.70692859 | 10.7782618 | 8.92E-05 | 0.00247921 |
| *GZF1* | 2.7046521 | 3.66402033 | 0.00089927 | 0.01233832 |
| *ENSGALG00000026396* | 2.6982058 | 5.15965031 | 0.00242539 | 0.02351747 |
| *CREB3L3* | 2.67248329 | 2.16462742 | 0.00157809 | 0.01797295 |
| *DDR2* | 2.66325004 | 4.89229186 | 0.00017539 | 0.00396368 |
| *MCAM* | 2.66015053 | 1.97655834 | 0.00368738 | 0.03090366 |
| *KANK3* | 2.65071903 | 3.16572301 | 0.00160978 | 0.01821886 |
| *ENSGALG00000038135* | 2.63317821 | 3.63242985 | 0.00070339 | 0.0104635 |
| *ZFP385B* | 2.62889222 | 4.76081677 | 0.0017234 | 0.01882701 |
| *ENSGALG00000006584* | 2.61851526 | 7.57822678 | 6.27E-07 | 5.01E-05 |
| *ENSGALG00000044504* | 2.60061471 | 5.00074582 | 5.56E-05 | 0.00169446 |
| *RXRA* | 2.59414723 | 4.17283187 | 0.0001206 | 0.00306301 |
| *ENSGALG00000029381* | 2.58417658 | 2.15067105 | 0.00092732 | 0.01262641 |
| *HSD17B3* | 2.55086774 | 1.89662013 | 0.0027392 | 0.02543505 |
| *ENSGALG00000023237* | 2.54904805 | 2.57374187 | 0.00165791 | 0.01850918 |
| *ENSGALG00000016774* | 2.535463 | 3.99840044 | 0.00259285 | 0.0245522 |
| *ENSGALG00000032940* | 2.52789923 | 7.00123755 | 0.0003697 | 0.00657058 |
| *CCNL1* | 2.5133469 | 6.26022402 | 8.32E-07 | 6.18E-05 |
| *ZFP651* | 2.50106809 | 0.89014679 | 0.00466906 | 0.03631288 |
| *ASB9* | 2.49928565 | 6.86282218 | 2.95E-07 | 2.90E-05 |
| *ENSGALG00000041228* | 2.48457905 | 6.42557766 | 0.00177368 | 0.01914103 |
| *KIF26B* | 2.46742789 | 4.05993054 | 0.00411139 | 0.03328276 |
| *ENSGALG00000012142* | 2.46572714 | 3.636083 | 0.00660338 | 0.04530458 |
| *ENSGALG00000000407* | 2.4648699 | 3.80375802 | 0.00249526 | 0.02389294 |
| *ENSGALG00000044678* | 2.46241597 | 3.11594022 | 5.23E-05 | 0.00161463 |
| *ADCY2* | 2.45944944 | 5.32262249 | 0.00160671 | 0.01819864 |
| *ABHD12B* | 2.4571493 | 1.58638625 | 0.00489617 | 0.03750194 |
| *HORMAD1* | 2.45283964 | 1.26959382 | 0.00743201 | 0.0486312 |
| *E4F1* | 2.44676279 | 3.04507434 | 0.00231991 | 0.02284333 |
| *SORBS2* | 2.42737002 | 4.95543558 | 0.00023408 | 0.00486021 |
| *ENSGALG00000038149* | 2.42531613 | 2.12687209 | 0.00595304 | 0.04236302 |
| *SOX8* | 2.41276512 | 5.62284031 | 0.00068732 | 0.01028936 |
| *ENSGALG00000017493* | 2.41267337 | 2.63900263 | 0.00411476 | 0.03328276 |
| *SLC4A7* | 2.40697578 | 8.44745227 | 1.74E-07 | 1.91E-05 |
| *RBM15* | 2.40126631 | 1.54586702 | 0.00493625 | 0.03766616 |
| *ENSGALG00000044031* | 2.39482116 | 1.52187782 | 0.00264982 | 0.0248423 |
| *ATP10A* | 2.39184054 | 4.99633446 | 0.00103312 | 0.01358316 |
| *ENSGALG00000044834* | 2.36587477 | 1.90649376 | 0.00461072 | 0.03605739 |

| | | | | |
|---|---|---|---|---|
| *CNGA3* | 2.36216139 | 4.50259257 | 4.24E-05 | 0.00139604 |
| *CALCA* | 2.36078672 | 3.58437762 | 5.74E-05 | 0.0017264 |
| *ENSGALG00000038429* | 2.34788092 | 4.85551012 | 0.00017132 | 0.00389651 |
| *FBXW8* | 2.34732005 | 3.68009758 | 1.41E-05 | 0.0006149 |
| *DDIAS* | 2.34627757 | 4.27742445 | 0.00046985 | 0.00787057 |
| *TOP3B* | 2.34160366 | 6.48392483 | 1.72E-08 | 2.89E-06 |
| *ENSGALG00000009026* | 2.33717204 | 5.42860125 | 0.00013383 | 0.00329831 |
| *ENSGALG00000037945* | 2.32529591 | 4.17659173 | 0.00053962 | 0.00866861 |
| *FDFT1* | 2.32352692 | 7.83465381 | 0.00010378 | 0.00274723 |
| *ENSGALG00000024701* | 2.31231721 | 2.93340913 | 0.00246145 | 0.02372678 |
| *MERTK* | 2.30647469 | 4.55221002 | 5.65E-06 | 0.00029163 |
| *TMEM173* | 2.3024583 | 2.65703635 | 0.00189531 | 0.02000966 |
| *ENSGALG00000032611* | 2.2987866 | 4.3327315 | 0.00021137 | 0.00449655 |
| *LUC7L* | 2.29504586 | 6.03713162 | 3.47E-07 | 3.29E-05 |
| *ENSGALG00000013858* | 2.28631187 | 5.22908865 | 2.76E-05 | 0.00102313 |
| *ATG9B* | 2.28409204 | 3.02384459 | 0.0008355 | 0.01170282 |
| *SDK2* | 2.28395482 | 4.01168047 | 0.00056923 | 0.00899758 |
| *ENSGALG00000031313* | 2.26761039 | 2.968713 | 0.00246557 | 0.02372678 |
| *ENSGALG00000046656* | 2.2674764 | 5.62900671 | 0.00052212 | 0.00849013 |
| *SHROOM4* | 2.26680905 | 5.36141999 | 3.42E-05 | 0.00119956 |
| *ENSGALG00000008411* | 2.26143414 | 4.91719522 | 0.00050941 | 0.00830875 |
| *SLC39A14* | 2.26118613 | 6.50914402 | 0.00010618 | 0.00279211 |
| *ENSGALG00000034870* | 2.25636048 | 3.0338269 | 0.00126165 | 0.01560088 |
| *ENSGALG00000002167* | 2.2514829 | 1.61214738 | 0.00528845 | 0.0392328 |
| *SLC38A3* | 2.24435515 | 3.96905199 | 0.00191514 | 0.02010702 |
| *ENSGALG00000040837* | 2.23944092 | 1.77975191 | 0.00167522 | 0.01855855 |
| *EPB41L4B* | 2.21891638 | 4.12379544 | 0.00018586 | 0.00411471 |
| *ENSGALG00000032781* | 2.21674353 | 1.95255004 | 0.00463121 | 0.03613772 |
| *ENSGALG00000030673* | 2.19605535 | 4.9602218 | 0.00617844 | 0.04339938 |
| *ENSGALG00000043364* | 2.18944277 | 2.72057633 | 0.00297011 | 0.02675665 |
| *FKBP5* | 2.18820312 | 5.61327548 | 9.86E-06 | 0.00046046 |
| *ENSGALG00000033303* | 2.18340279 | 3.39090503 | 0.00334067 | 0.02883496 |
| *SLC35B4* | 2.17637625 | 4.95136238 | 0.00058779 | 0.0092394 |
| *ENSGALG00000032465* | 2.17381339 | 5.40436213 | 0.0009658 | 0.01303738 |
| *ENSGALG00000032910* | 2.16755855 | 3.97690767 | 0.00179932 | 0.01931945 |
| *ENSGALG00000009372* | 2.16629183 | 2.067593 | 0.00593486 | 0.04228941 |
| *GRK6* | 2.16450721 | 4.70344093 | 9.16E-06 | 0.00043366 |
| *ENSGALG00000021171* | 2.15718533 | 5.11399439 | 0.00054747 | 0.00875149 |
| *RUNX2* | 2.15506802 | 6.93985742 | 0.00128151 | 0.01573738 |
| *ENSGALG00000044540* | 2.15462084 | 2.48555936 | 0.0014206 | 0.01680306 |

| | | | | |
|---|---|---|---|---|
| *ID4* | 2.14344857 | 7.00296948 | 0.00116578 | 0.01469906 |
| *ENSGALG00000044344* | 2.13804136 | 2.96634507 | 0.00540294 | 0.03983088 |
| *ENSGALG00000002461* | 2.12435691 | 3.78067824 | 0.002392 | 0.02333769 |
| *ENSGALG00000033758* | 2.12380993 | 4.5841023 | 0.00579264 | 0.04161932 |
| *MT-ND4L* | 2.12324678 | 5.50760256 | 0.002205 | 0.02208736 |
| *ENSGALG00000041287* | 2.11274955 | 4.24093277 | 0.00011525 | 0.00297514 |
| *PNPLA6* | 2.10403855 | 3.66694465 | 0.00291868 | 0.02646034 |
| *ENSGALG00000030881* | 2.10258785 | 5.83551592 | 0.00062029 | 0.00963247 |
| *KLHDC1* | 2.09449324 | 7.49903858 | 8.23E-06 | 0.00040007 |
| *ENSGALG00000014508* | 2.06876846 | 4.61055657 | 0.00490359 | 0.0375199 |
| *ENSGALG00000045764* | 2.05977915 | 2.83733145 | 0.00327596 | 0.02857271 |
| *FRY* | 2.05363386 | 6.95179119 | 0.00024747 | 0.00501765 |
| *ENSGALG00000032816* | 2.04961972 | 3.07630348 | 0.00593971 | 0.04228941 |
| *PANX3* | 2.04003997 | 7.40978117 | 0.00101792 | 0.01348366 |
| *ENSGALG00000001734* | 2.03654663 | 2.75563805 | 0.00681512 | 0.0462193 |
| *TRPC4AP* | 2.03039385 | 7.31635599 | 3.79E-06 | 0.00021794 |
| *ENSGALG00000037976* | 2.03012492 | 3.94945807 | 0.00115411 | 0.01461702 |
| *LSS* | 2.02482504 | 6.92703743 | 0.00156296 | 0.01787891 |
| *AR* | 2.01812297 | 4.39552075 | 3.72E-05 | 0.00127238 |
| *TEAD3* | 2.00925147 | 3.66402753 | 0.00532725 | 0.03941662 |
| *ANAPC5* | 2.00888171 | 4.65580329 | 0.00282289 | 0.02591528 |
| *SRSF4* | 2.00041402 | 3.05795562 | 0.00146214 | 0.01715163 |

**Supplementary table S8.** Upregulated genes in head IMM compared to limb IMM

| GENE NAME | LOGFC | LOGCPM | PVALUE | P.ADJ |
|---|---|---|---|---|
| *CAV3* | 10.5885373 | 3.46259516 | 2.93E-07 | 2.90E-05 |
| *ENSGALG00000039977* | 9.96063758 | 5.57103418 | 9.22E-09 | 1.74E-06 |
| *ENSGALG00000032404* | 9.75762615 | 1.87841602 | 2.03E-05 | 0.00081553 |
| *FHL2* | 9.68778699 | 4.26096148 | 5.33E-07 | 4.51E-05 |
| *GJD2* | 9.50594011 | 2.36972975 | 6.75E-07 | 5.30E-05 |
| *TMEM182* | 9.43590592 | 5.49856343 | 3.07E-08 | 4.52E-06 |
| *ENSGALG00000032304* | 9.41232699 | 4.87717549 | 7.91E-15 | 1.12E-11 |
| *ENSGALG00000004518* | 9.33249464 | 8.99458198 | 1.23E-08 | 2.23E-06 |
| *ENSGALG00000003465* | 9.31515992 | 7.67106563 | 4.33E-10 | 1.39E-07 |
| *APOB* | 9.2925827 | 1.90948876 | 0.00043232 | 0.00742238 |
| *ENSGALG00000042257* | 9.17948718 | 5.13772168 | 1.20E-06 | 8.55E-05 |
| *ACTC1* | 9.14007017 | 8.71533357 | 6.34E-09 | 1.25E-06 |
| *ENSGALG00000027323* | 8.87902511 | 6.5599603 | 1.08E-07 | 1.32E-05 |

| | | | | |
|---|---|---|---|---|
| *TNNT3* | 8.7883652 | 6.64615848 | 4.16E-08 | 5.78E-06 |
| *MYOG* | 8.76595247 | 4.13907813 | 8.29E-12 | 4.63E-09 |
| *ENSGALG00000028612* | 8.65670722 | 6.05253221 | 3.22E-07 | 3.10E-05 |
| *ENSGALG00000015599* | 8.60661232 | 2.12846798 | 5.18E-08 | 6.92E-06 |
| *TRIM55* | 8.56684945 | 3.54908023 | 6.65E-08 | 8.63E-06 |
| *MYL2* | 8.50509004 | 6.11552326 | 3.03E-07 | 2.94E-05 |
| *FST* | 8.49252454 | 4.2235345 | 8.51E-12 | 4.63E-09 |
| *CDH5* | 8.47645034 | 6.0480373 | 9.61E-08 | 1.19E-05 |
| *SLC6A11* | 8.47163143 | 1.61297167 | 0.00030068 | 0.00572021 |
| *ADPRHL1* | 8.33850507 | 4.48486799 | 8.50E-07 | 6.26E-05 |
| *CRB1* | 8.32717221 | 0.57470946 | 0.0001997 | 0.00433974 |
| *SMYD1* | 8.31152936 | 4.21209766 | 1.40E-06 | 9.73E-05 |
| *MYL1* | 8.21959149 | 7.82595415 | 3.45E-08 | 4.93E-06 |
| *XIRP1* | 8.12097426 | 4.36833602 | 4.18E-06 | 0.00023306 |
| *ENSGALG00000030894* | 8.07959452 | 3.58092759 | 0.00083959 | 0.01173682 |
| *DHRS7C* | 8.0704828 | 0.97769543 | 4.87E-06 | 0.00026197 |
| *CDH17* | 8.03797098 | 0.73164838 | 2.62E-05 | 0.00097962 |
| *CRHBP* | 8.0316956 | 3.76017587 | 0.000127 | 0.00319601 |
| *STMN2* | 8.02323788 | 3.61530751 | 2.76E-06 | 0.000165 |
| *MYOZ2* | 7.98401573 | 5.22279688 | 2.09E-08 | 3.39E-06 |
| *ENSGALG00000001177* | 7.97246146 | 4.89278282 | 9.18E-15 | 1.18E-11 |
| *PPARGC1A* | 7.87029487 | 1.28481541 | 5.99E-06 | 0.00030358 |
| *ENSGALG00000030025* | 7.86067347 | 2.52280731 | 7.21E-07 | 5.51E-05 |
| *ENSGALG00000032851* | 7.8112757 | 1.99395375 | 1.34E-06 | 9.37E-05 |
| *FREM2* | 7.78182669 | 0.44958204 | 0.00075962 | 0.01096562 |
| *CACNG1* | 7.77084037 | 2.92865561 | 4.30E-05 | 0.00140169 |
| *ENSGALG00000032550* | 7.74670623 | 7.00706374 | 2.76E-06 | 0.000165 |
| *ARHGAP25* | 7.74170159 | 2.82574752 | 6.80E-08 | 8.75E-06 |
| *DPYS* | 7.73435173 | 0.70182635 | 4.03E-05 | 0.0013441 |
| *ENSGALG00000027165* | 7.72014771 | 0.9536356 | 7.52E-06 | 0.00037187 |
| *ENSGALG00000031496* | 7.71870295 | 1.79815487 | 3.70E-06 | 0.00021389 |
| *TTN* | 7.70014697 | 4.78403451 | 5.82E-06 | 0.00029812 |
| *TMPRSS2* | 7.68631881 | 0.18661082 | 0.00478478 | 0.036969 |
| *KERA* | 7.68419617 | 3.55637018 | 8.85E-05 | 0.00246984 |
| *MYMK* | 7.66175058 | 1.00074819 | 0.0004965 | 0.00816739 |
| *GRM1* | 7.6413357 | -0.0112934 | 0.00167355 | 0.01855855 |
| *STMN4* | 7.61367548 | 0.4846601 | 1.16E-05 | 0.00052466 |
| *ENSGALG00000037864* | 7.57568597 | 1.75823373 | 3.72E-05 | 0.00127238 |
| *SLITRK1* | 7.55677814 | -0.1852808 | 0.00085306 | 0.01185484 |
| *AQP9* | 7.54189295 | 1.70303462 | 3.36E-06 | 0.00019562 |

| | | | | |
|---|---|---|---|---|
| *SLC10A2* | 7.52455557 | 0.5488305 | 0.00087889 | 0.01213042 |
| *MAP3K7CL* | 7.51289331 | -0.184876 | 0.00013201 | 0.00327647 |
| *SH3BGR* | 7.48803435 | 3.83472814 | 6.96E-08 | 8.87E-06 |
| *ISL1* | 7.48203274 | 1.13775782 | 0.00115692 | 0.0146395 |
| *IL18RAP* | 7.46774449 | 0.36411696 | 0.00216573 | 0.0218379 |
| *MYOT* | 7.45474198 | 3.34006986 | 9.78E-05 | 0.00265098 |
| *MYL10* | 7.43444082 | 7.66560149 | 5.83E-07 | 4.80E-05 |
| *IRX1* | 7.42497472 | 2.24769059 | 0.00215213 | 0.02176283 |
| *ACTA2* | 7.41919915 | 6.02568577 | 1.79E-12 | 1.41E-09 |
| *ENSGALG00000005242* | 7.41692944 | 0.29280827 | 0.00660245 | 0.04530458 |
| *CRYAB* | 7.41430337 | 3.73609505 | 2.10E-09 | 5.12E-07 |
| *ENSGALG00000013239* | 7.40823508 | 6.52484069 | 1.48E-07 | 1.68E-05 |
| *KLHL31* | 7.3887219 | 1.04532434 | 0.00299193 | 0.02691535 |
| *KBTBD12* | 7.38081559 | -0.0762585 | 0.00075577 | 0.01093071 |
| *PTCHD4* | 7.36262323 | -0.2684816 | 0.00753139 | 0.04909982 |
| *BRINP3* | 7.36055028 | 0.89628866 | 0.00038136 | 0.00670189 |
| *ENSGALG00000007361* | 7.35924141 | 2.02181505 | 0.00498316 | 0.03782015 |
| *HYDIN* | 7.35645151 | 0.408752 | 6.98E-06 | 0.00034757 |
| *TNNI1* | 7.35547715 | 4.91858476 | 2.70E-06 | 0.00016328 |
| *ENSGALG00000029510* | 7.2821356 | -0.1488274 | 0.00261366 | 0.02464407 |
| *DES* | 7.27662771 | 0.65818578 | 0.00064236 | 0.00985628 |
| *FBXO43* | 7.27310796 | -0.0963626 | 0.00357823 | 0.03023507 |
| *ENSGALG00000016473* | 7.26836886 | 6.02942169 | 4.99E-09 | 1.04E-06 |
| *CHODL* | 7.19837586 | 6.37100194 | 8.09E-09 | 1.55E-06 |
| *MYOM2* | 7.1823179 | 5.1968873 | 5.78E-09 | 1.19E-06 |
| *GHRHR* | 7.13253737 | -0.0401863 | 0.0024671 | 0.02372678 |
| *TRDN* | 7.13173097 | 3.35494861 | 1.14E-05 | 0.00051517 |
| *ENSGALG00000007727* | 7.10673986 | 0.00604238 | 0.00601952 | 0.0425851 |
| *PITX2* | 7.08717158 | 3.55236217 | 3.03E-07 | 2.94E-05 |
| *FGA* | 7.06337262 | -0.6106642 | 0.00039338 | 0.00688763 |
| *ENSGALG00000033591* | 7.06290784 | 0.23190576 | 0.00218021 | 0.021937 |
| *VIP* | 7.04249368 | 3.27528891 | 2.79E-05 | 0.00102498 |
| *ENSGALG00000001977* | 7.02806554 | 3.73798248 | 6.63E-05 | 0.00194903 |
| *TMC2* | 7.01746042 | 0.41077197 | 0.00521189 | 0.03883636 |
| *GUCY2D* | 7.01519951 | 0.07015753 | 0.00194155 | 0.02022186 |
| *MYBPC3* | 7.00589066 | 4.67747686 | 1.02E-06 | 7.43E-05 |
| *ENSGALG00000000302* | 7.00150134 | 7.41146989 | 1.02E-07 | 1.25E-05 |
| *ENSGALG00000040413* | 7.00074774 | -0.6251202 | 0.00220176 | 0.02207529 |
| *ENSGALG00000028539* | 6.99175607 | 0.88726334 | 0.00028715 | 0.00557235 |
| *C1QA* | 6.97976503 | 1.63249569 | 4.63E-05 | 0.00148717 |

| | | | | |
|---|---|---|---|---|
| *SLITRK4* | 6.9580806 | -0.1179616 | 0.00141721 | 0.01680306 |
| *ENSGALG00000020084* | 6.91296741 | 0.05050974 | 0.00113164 | 0.01440599 |
| *ENSGALG00000023781* | 6.90563145 | 1.2351745 | 0.00015921 | 0.0037356 |
| *MARCHF11* | 6.89429033 | -0.4765069 | 0.00555512 | 0.04050939 |
| *MAT1A* | 6.89424022 | 5.59230954 | 6.54E-07 | 5.17E-05 |
| *FGF4* | 6.84193264 | -0.6379621 | 0.00709329 | 0.04755698 |
| *ENSGALG00000046062* | 6.84120846 | 0.22052363 | 0.00747238 | 0.04881311 |
| *ENSGALG00000035784* | 6.8365501 | 0.81154452 | 0.00159169 | 0.01807189 |
| *OTC* | 6.82539615 | -0.3455415 | 0.00114017 | 0.0144534 |
| *BEST3* | 6.82496036 | 1.18793896 | 0.00181165 | 0.01941619 |
| *ENSGALG00000005448* | 6.81953893 | 5.25971228 | 2.08E-06 | 0.00013215 |
| *ENSGALG00000037349* | 6.81475549 | -0.6771688 | 0.00162801 | 0.01830881 |
| *ENSGALG00000007511* | 6.81111647 | 1.93107616 | 8.01E-05 | 0.00228458 |
| *ZIC1* | 6.80632266 | 1.22054139 | 0.00073066 | 0.01076854 |
| *CNN1* | 6.79344113 | -0.1516954 | 0.00034907 | 0.00632294 |
| *ENSGALG00000019221* | 6.78846035 | -0.3226345 | 0.00623882 | 0.04369338 |
| *ENSGALG00000031869* | 6.78810715 | 1.20369427 | 0.00625041 | 0.04373122 |
| *ENSGALG00000037227* | 6.78286085 | 0.04921017 | 0.00375524 | 0.03134237 |
| *ENSGALG00000034892* | 6.77299662 | 1.9879648 | 0.00176666 | 0.01912033 |
| *ENSGALG00000015517* | 6.74397372 | 0.07915041 | 0.00116097 | 0.01465705 |
| *ENSGALG00000032908* | 6.74375515 | 3.00666361 | 0.00079508 | 0.01137309 |
| *ENSGALG00000010391* | 6.73041749 | 0.24690067 | 0.00191155 | 0.02010702 |
| *ENSGALG00000032066* | 6.728999 | -0.326327 | 0.00010463 | 0.0027616 |
| *ENSGALG00000033505* | 6.72564528 | 1.1350326 | 3.28E-05 | 0.00117197 |
| *TNNC1* | 6.70066368 | 7.98228882 | 1.19E-07 | 1.43E-05 |
| *ENSGALG00000043523* | 6.7004638 | 1.47485496 | 0.00312283 | 0.02768091 |
| *P2RX7* | 6.68438049 | -0.3457196 | 0.00309895 | 0.02753825 |
| *UNC45B* | 6.67593941 | 1.82818651 | 0.00013911 | 0.00338904 |
| *IGF1* | 6.67260808 | -0.1336837 | 0.00281973 | 0.02590306 |
| *CHRNA9* | 6.66972827 | 0.38509068 | 0.00724025 | 0.04802279 |
| *GYS2* | 6.65365049 | 0.80173828 | 0.00199808 | 0.02064777 |
| *LVRN* | 6.6462546 | 2.81748296 | 7.85E-06 | 0.00038677 |
| *AQP1* | 6.63460455 | 6.79863455 | 9.35E-10 | 2.59E-07 |
| *MUSTN1* | 6.62791247 | 4.05195898 | 7.88E-12 | 4.63E-09 |
| *ENSGALG00000041858* | 6.62316374 | -0.4163737 | 0.0002077 | 0.00445958 |
| *EDNRB* | 6.61748257 | 2.62087312 | 4.08E-07 | 3.68E-05 |
| *CHRNG* | 6.61680407 | 2.98950323 | 3.51E-05 | 0.00122075 |
| *ENSGALG00000031717* | 6.59798623 | 0.49708742 | 0.00260168 | 0.02460591 |
| *KY* | 6.59295136 | -0.2903856 | 0.00076929 | 0.01108258 |
| *TLR4* | 6.58441932 | 0.86752678 | 0.00027113 | 0.00533843 |

| | | | | |
|---|---|---|---|---|
| *BCL11B* | 6.5808162 | -0.0267091 | 0.0035215 | 0.0299211 |
| *SYT10* | 6.5800137 | 0.22223536 | 0.00127099 | 0.01567628 |
| *ENSGALG00000045426* | 6.57352184 | 6.1145717 | 4.52E-12 | 2.91E-09 |
| *ENSGALG00000031479* | 6.57329943 | 1.1873117 | 0.00740649 | 0.04855583 |
| *MYH7B* | 6.57270134 | 3.39139013 | 0.00033415 | 0.00612339 |
| *ENSGALG00000015276* | 6.56999048 | -0.6242605 | 0.0043523 | 0.03457157 |
| *RBM44* | 6.56547607 | -0.4624594 | 0.00084054 | 0.01173845 |
| *ENSGALG00000044013* | 6.5367138 | -0.1071613 | 0.00287976 | 0.02630087 |
| *ENSGALG00000006835* | 6.53111839 | 7.8313238 | 2.22E-07 | 2.33E-05 |
| *ENSGALG00000027483* | 6.52488417 | 2.64320185 | 1.17E-08 | 2.15E-06 |
| *ENSGALG00000016693* | 6.52222228 | 1.31761668 | 2.04E-05 | 0.000816 |
| *DBX1* | 6.52114265 | 1.01658747 | 0.00152441 | 0.01761918 |
| *ENSGALG00000039293* | 6.50416765 | 0.32053995 | 0.00490904 | 0.03753705 |
| *DLX2* | 6.50397892 | -0.0729698 | 0.00017754 | 0.00399944 |
| *LSP1* | 6.48179662 | 6.53134721 | 2.65E-12 | 1.98E-09 |
| *ENSGALG00000031211* | 6.47169186 | 0.41736839 | 0.00117975 | 0.01482226 |
| *DCX* | 6.47153047 | 4.78431661 | 5.54E-10 | 1.67E-07 |
| *ENSGALG00000012418* | 6.47042633 | -0.1002215 | 0.0074039 | 0.04855583 |
| *NOV* | 6.46922494 | 8.49871739 | 7.97E-07 | 6.00E-05 |
| *CDH23* | 6.44994868 | -0.6481129 | 0.00215809 | 0.02179197 |
| *ZEB2* | 6.44516793 | 5.30088382 | 5.87E-06 | 0.00029878 |
| *ENSGALG00000032425* | 6.44197447 | 3.29160263 | 0.00143925 | 0.01696758 |
| *VSNL1* | 6.4007618 | 0.4299369 | 0.00018865 | 0.00415362 |
| *ENSGALG00000010379* | 6.38595508 | 1.71611699 | 5.26E-07 | 4.48E-05 |
| *FHDC1* | 6.37693472 | 0.37976238 | 0.00159624 | 0.01810904 |
| *COL4A1* | 6.36204635 | 6.36515342 | 4.42E-07 | 3.91E-05 |
| *HTR2C* | 6.35265083 | 0.29615182 | 0.00045846 | 0.00778609 |
| *ASB2* | 6.34471993 | 0.14911114 | 0.00090839 | 0.01241639 |
| *FABP1* | 6.31865064 | 1.03059135 | 0.00020371 | 0.00440654 |
| *ENSGALG00000032453* | 6.31377518 | -0.7636989 | 0.00194257 | 0.02022186 |
| *ENPEP* | 6.30235857 | 2.75549496 | 0.00089112 | 0.01226323 |
| *ENSGALG00000042686* | 6.29451402 | 2.12680082 | 1.35E-05 | 0.00059189 |
| *CORO6* | 6.27901338 | 0.38700203 | 0.00203378 | 0.02092495 |
| *ENSGALG00000031108* | 6.27526983 | 2.79168133 | 3.78E-07 | 3.47E-05 |
| *ENSGALG00000037544* | 6.27312181 | 3.17514341 | 0.00157773 | 0.01797295 |
| *BIRC7* | 6.2683587 | -0.535151 | 0.00726901 | 0.0481887 |
| *ENSGALG00000033476* | 6.26813145 | 0.63898422 | 3.31E-05 | 0.0011777 |
| *ENSGALG00000045849* | 6.26794972 | -0.1813153 | 0.00719367 | 0.04784618 |
| *ENSGALG00000035290* | 6.2517748 | 4.0398556 | 0.00036489 | 0.0065178 |
| *ENSGALG00000035930* | 6.24930324 | 1.31347021 | 8.28E-05 | 0.00233211 |

| | | | | |
|---|---|---|---|---|
| *DCSTAMP* | 6.23001286 | 0.68998184 | 0.00118736 | 0.0148937 |
| *ENSGALG00000044599* | 6.21031255 | 0.91786128 | 0.00386272 | 0.03193799 |
| *ENSGALG00000033305* | 6.20703798 | 1.78080257 | 0.00222766 | 0.02217787 |
| *CCM2L* | 6.20132981 | 0.60127482 | 0.00061195 | 0.0095239 |
| *ALDOB* | 6.18171594 | 2.07721188 | 1.28E-08 | 2.30E-06 |
| *TNNI2* | 6.1621706 | 6.1159296 | 2.02E-06 | 0.00012951 |
| *ENSGALG00000043438* | 6.16126491 | 1.70289648 | 0.00466069 | 0.03626773 |
| *C1QC* | 6.16069186 | 1.09218851 | 0.00063389 | 0.00976869 |
| *HIC1* | 6.14689667 | 2.91661728 | 2.42E-08 | 3.80E-06 |
| *ENSGALG00000030522* | 6.12878772 | 3.12328235 | 0.0010063 | 0.01337989 |
| *FGF7* | 6.12413345 | 2.77816899 | 5.31E-05 | 0.00163218 |
| *MYOD1* | 6.11487839 | 5.79601303 | 2.73E-08 | 4.11E-06 |
| *ENSGALG00000044263* | 6.08452881 | 1.25011075 | 0.00046285 | 0.00781384 |
| *GATA5* | 6.07826174 | -0.6542852 | 0.00605237 | 0.0427687 |
| *ENSGALG00000042491* | 6.06122191 | -0.0762583 | 0.00028968 | 0.00559846 |
| *1700019D03RIK* | 6.06023907 | -0.5030558 | 0.00258131 | 0.02446698 |
| *ENSGALG00000031929* | 6.05735812 | 1.76539867 | 0.0001508 | 0.00359766 |
| *ENSGALG00000002118* | 6.05716544 | 0.47306942 | 0.00141915 | 0.01680306 |
| *ENSGALG00000015345* | 6.02368358 | 0.81353142 | 0.00238998 | 0.02333769 |
| *CHRND* | 6.00428044 | 1.15850467 | 0.00080403 | 0.01144324 |
| *ENSGALG00000041978* | 5.99377513 | 0.22660099 | 0.0033445 | 0.02885035 |
| *MLIP* | 5.98424405 | 1.31498697 | 0.00121899 | 0.01518052 |
| *3425401B19RIK* | 5.97333509 | 0.95170542 | 0.00132014 | 0.01602352 |
| *ENSGALG00000042752* | 5.97262824 | -0.742057 | 0.00241339 | 0.02343459 |
| *ENSGALG00000042352* | 5.97176914 | 3.93468068 | 0.00039583 | 0.00691341 |
| *COL10A1* | 5.97095112 | 6.14188559 | 2.70E-07 | 2.71E-05 |
| *LZTS3* | 5.96334529 | -0.9105561 | 0.00342377 | 0.02930195 |
| *RBM24* | 5.95889505 | 7.69902722 | 2.59E-16 | 4.58E-13 |
| *SMPX* | 5.95132235 | 0.58537472 | 0.00246411 | 0.02372678 |
| *APOBEC2* | 5.94476991 | 3.96876018 | 1.32E-07 | 1.55E-05 |
| *UCN3* | 5.94079302 | -1.2732044 | 0.00764495 | 0.04961151 |
| *SFRP2* | 5.93692582 | 7.15960765 | 2.32E-09 | 5.38E-07 |
| *ENSGALG00000014126* | 5.91124967 | -0.1424398 | 0.00440473 | 0.03489012 |
| *ENSGALG00000037077* | 5.89916681 | 1.24746197 | 0.00013143 | 0.00326769 |
| *ENSGALG00000036155* | 5.89787473 | 0.62051825 | 0.00318614 | 0.02801391 |
| *ENSGALG00000015395* | 5.88515865 | -0.508263 | 0.00751342 | 0.0490052 |
| *ENSGALG00000037425* | 5.88165841 | 2.8278114 | 8.58E-07 | 6.29E-05 |
| *ADAMTS15* | 5.87097352 | 2.58067834 | 4.93E-06 | 0.00026432 |
| *DPT* | 5.8366594 | -0.1931866 | 0.0009156 | 0.01250283 |
| *PAX7* | 5.82642111 | 2.30646936 | 5.17E-05 | 0.00160132 |

| | | | | |
|---|---|---|---|---|
| *ENSGALG00000015422* | 5.82087246 | 4.84879048 | 1.15E-10 | 4.41E-08 |
| *ENSGALG00000038251* | 5.81338142 | 3.13145806 | 5.96E-07 | 4.85E-05 |
| *ENSGALG00000040785* | 5.81189681 | 4.13642684 | 1.73E-11 | 9.06E-09 |
| *MYOM1* | 5.7959144 | 6.23086464 | 1.47E-06 | 0.00010073 |
| *ENSGALG00000012420* | 5.78840103 | 6.07133333 | 4.36E-09 | 9.20E-07 |
| *ENSGALG00000039452* | 5.78411058 | -0.2695247 | 0.0052388 | 0.03898648 |
| *ENSGALG00000011277* | 5.78126912 | -0.902363 | 0.00222448 | 0.02217024 |
| *OTOG* | 5.78072114 | 1.94124944 | 0.00335442 | 0.02890075 |
| *ARHGEF9* | 5.77078721 | 2.55770952 | 6.55E-06 | 0.00032957 |
| *CYP11A1* | 5.76036426 | -0.5891918 | 0.00029474 | 0.0056424 |
| *ENSGALG00000008359* | 5.74945018 | 2.97684636 | 0.00032354 | 0.00601993 |
| *ENSGALG00000034806* | 5.74582557 | 0.57853916 | 0.00702641 | 0.0472596 |
| *ENSGALG00000019147* | 5.73610485 | 1.45241293 | 0.00053492 | 0.00863326 |
| *ENSGALG00000029310* | 5.7278571 | 2.9076891 | 0.00145887 | 0.01712754 |
| *SATB2* | 5.72386423 | 4.4129566 | 5.54E-07 | 4.64E-05 |
| *KCNJ2* | 5.71839129 | 2.55886275 | 1.25E-05 | 0.00055918 |
| *ACTN2* | 5.6684689 | 7.78528019 | 5.92E-09 | 1.20E-06 |
| *ENSGALG00000039152* | 5.66652159 | 1.14800464 | 0.00226433 | 0.02241668 |
| *ENSGALG00000027090* | 5.66394161 | 1.74405959 | 0.00025472 | 0.00509691 |
| *PCDH20* | 5.65981353 | 0.7408147 | 0.0075434 | 0.04913283 |
| *SPON1* | 5.64737288 | 5.5857537 | 2.66E-08 | 4.05E-06 |
| *POSTN* | 5.64368146 | 6.77673611 | 2.61E-06 | 0.00015903 |
| *ITIH5* | 5.62829878 | 0.96807099 | 3.40E-05 | 0.00119602 |
| *CAMK1D* | 5.61763824 | 0.45813821 | 0.0002538 | 0.00508928 |
| *TBX1* | 5.61396297 | -0.3188367 | 0.00015433 | 0.00364483 |
| *CAVIN4* | 5.61174062 | 3.86605797 | 3.58E-11 | 1.64E-08 |
| *ENSGALG00000045170* | 5.61025331 | -0.4344871 | 0.00040702 | 0.00706519 |
| *UPF3A* | 5.60761015 | -0.1944225 | 0.00581152 | 0.04169711 |
| *GPR65* | 5.6069534 | -0.4836161 | 0.00742881 | 0.0486312 |
| *ENSGALG00000030546* | 5.60479936 | 1.42600911 | 4.01E-05 | 0.00134021 |
| *CYP24A1* | 5.60081304 | 2.06252758 | 2.11E-05 | 0.00083044 |
| *FILIP1* | 5.60029767 | 6.33898155 | 1.18E-16 | 2.38E-13 |
| *ENSGALG00000040442* | 5.59788298 | 1.87741098 | 0.00081174 | 0.01150663 |
| *ENSGALG00000016499* | 5.59584368 | 0.23722147 | 0.00366288 | 0.03073476 |
| *PLCXD2* | 5.59354153 | -0.389945 | 0.00718712 | 0.04784618 |
| *WISP1* | 5.58743609 | 5.30272637 | 8.18E-17 | 1.93E-13 |
| *ENSGALG00000044476* | 5.57252595 | 1.52501851 | 0.00150992 | 0.01748027 |
| *ENSGALG00000035594* | 5.56949521 | 2.45341762 | 3.83E-06 | 0.00021908 |
| *FBLN5* | 5.56848019 | 2.20107096 | 3.85E-05 | 0.0013031 |
| *LMOD2* | 5.55983185 | 0.57557054 | 0.00196081 | 0.02036267 |

| | | | | |
|---|---|---|---|---|
| *RGS18* | 5.55206789 | 2.97874114 | 8.41E-06 | 0.00040682 |
| *AMIGO2* | 5.55095762 | 0.72136429 | 0.00022298 | 0.00470823 |
| *PAH* | 5.53747172 | 0.76182411 | 0.00148342 | 0.01730084 |
| *ENSGALG00000008427* | 5.53589584 | 0.1417774 | 0.00419822 | 0.03374554 |
| *GATM* | 5.5328186 | 4.31760037 | 4.73E-06 | 0.00025725 |
| *ENSGALG00000032541* | 5.53129322 | 0.71142672 | 0.00186275 | 0.0197989 |
| *RASGRF2* | 5.52996855 | 1.79511193 | 1.45E-05 | 0.00062754 |
| *WDFY4* | 5.52784922 | 2.95107411 | 4.03E-06 | 0.0002288 |
| *LMOD1* | 5.52542231 | 1.01375397 | 6.88E-05 | 0.00201804 |
| *ENSGALG00000030510* | 5.52214178 | 1.61773969 | 0.00096055 | 0.01297889 |
| *CNR1* | 5.51645635 | 5.69068874 | 9.74E-14 | 1.06E-10 |
| *ENSGALG00000045632* | 5.51313094 | 3.92715848 | 0.00133808 | 0.01613793 |
| *ENSGALG00000016325* | 5.51304733 | 3.13684658 | 2.95E-06 | 0.00017436 |
| *ENSGALG00000035505* | 5.50766435 | 1.21009854 | 0.00727403 | 0.04819938 |
| *EN1* | 5.49797421 | 1.21315471 | 0.00166341 | 0.0185439 |
| *ENSGALG00000001191* | 5.49706631 | 2.95682651 | 1.82E-07 | 1.98E-05 |
| *TFEC* | 5.49418297 | 0.89451148 | 0.00286746 | 0.0262393 |
| *ENSGALG00000009511* | 5.49228392 | 1.69851577 | 0.00522699 | 0.03891907 |
| *PCDH19* | 5.49214825 | -0.185879 | 0.00302802 | 0.02716382 |
| *NME8* | 5.48841648 | 0.72623243 | 0.00287738 | 0.02630087 |
| *RPS6KA2* | 5.47941475 | 1.47346556 | 0.00028806 | 0.00558238 |
| *MYF6* | 5.4535863 | 2.18443303 | 0.00016843 | 0.00386185 |
| *PSTPIP2* | 5.44944731 | -0.2955247 | 0.00582994 | 0.04174608 |
| *ENSGALG00000039973* | 5.44438811 | 1.18290466 | 4.50E-05 | 0.00145741 |
| *FAM26E* | 5.44030281 | 2.8471059 | 1.05E-05 | 0.00048677 |
| *ENSGALG00000029660* | 5.43424273 | 1.61755717 | 0.00067542 | 0.01015428 |
| *ENSGALG00000012644* | 5.42049301 | 3.75033926 | 1.80E-09 | 4.46E-07 |
| *EEF1A2* | 5.4204712 | 4.2423589 | 1.36E-07 | 1.58E-05 |
| *NPL* | 5.41465481 | 3.59046696 | 1.34E-07 | 1.57E-05 |
| *ENSGALG00000040151* | 5.4060465 | -0.0285415 | 0.00395284 | 0.03238029 |
| *PRKCQ* | 5.40336995 | 1.18158194 | 0.00174886 | 0.019017 |
| *ENSGALG00000010338* | 5.40054009 | -0.3858496 | 0.00696164 | 0.04689823 |
| *ENSGALG00000040995* | 5.39864068 | 2.35246002 | 0.0032788 | 0.02857919 |
| *PTPRQ* | 5.38740465 | 2.18738562 | 0.00213807 | 0.02165953 |
| *ENSGALG00000002012* | 5.38543104 | 3.17901766 | 0.00024198 | 0.0049542 |
| *KEL* | 5.36842962 | 0.60914385 | 0.00407521 | 0.03315716 |
| *ENSGALG00000006190* | 5.36127066 | 5.2398656 | 4.29E-07 | 3.82E-05 |
| *ENSGALG00000034053* | 5.3570808 | 1.16897046 | 7.26E-05 | 0.00210947 |
| *PLXNA4* | 5.35497831 | 2.38505165 | 0.00022467 | 0.00473467 |
| *C1QB* | 5.35416158 | 1.91717035 | 3.37E-05 | 0.00119048 |

| | | | | |
|---|---|---|---|---|
| *TXLNB* | 5.34746824 | 1.73900834 | 0.00011649 | 0.00299085 |
| *ENSGALG00000007268* | 5.34035574 | 0.25972493 | 0.00085104 | 0.01185001 |
| *MYT1L* | 5.32699706 | 1.40679579 | 0.00262232 | 0.02466615 |
| *PLXNB2* | 5.32686281 | 4.78160927 | 9.70E-13 | 9.15E-10 |
| *KLHL4* | 5.32224324 | 1.15692525 | 0.00244848 | 0.02364413 |
| *ARAP3* | 5.31954896 | 1.39323009 | 0.00206821 | 0.02111041 |
| *GEM* | 5.31772387 | 3.98479611 | 8.90E-11 | 3.50E-08 |
| *ENSGALG00000019719* | 5.31662657 | 3.08105252 | 0.0002592 | 0.00517921 |
| *BMX* | 5.30997006 | 0.48366319 | 0.00642849 | 0.04455844 |
| *ATP1B4* | 5.3036505 | 0.5357047 | 9.93E-05 | 0.00267581 |
| *LCP1* | 5.30341771 | 4.8236852 | 6.39E-08 | 8.37E-06 |
| *ENSGALG00000012522* | 5.29804929 | 1.67696225 | 0.00049965 | 0.0082097 |
| *SPRYD4* | 5.29320677 | 2.01602535 | 1.09E-05 | 0.00049857 |
| *MACC1* | 5.2916002 | 0.32015689 | 0.0066175 | 0.04535745 |
| *CDH12* | 5.28461283 | 1.3888665 | 0.0063038 | 0.04401772 |
| *ST8SIA2* | 5.27791927 | 0.02632174 | 0.0056984 | 0.04123399 |
| *A* | 5.26235988 | 2.09891856 | 0.00106607 | 0.01391082 |
| *ENSGALG00000042713* | 5.25376624 | 2.71828566 | 0.00518833 | 0.03873314 |
| *ENSGALG00000041003* | 5.24456203 | 1.68474595 | 0.00578006 | 0.04156351 |
| *CHN2* | 5.24174762 | 0.8675416 | 0.00148502 | 0.01730528 |
| *TNFAIP6* | 5.24081167 | 2.55135452 | 2.11E-05 | 0.00083014 |
| *DCLK3* | 5.23899836 | 3.67506208 | 1.74E-07 | 1.91E-05 |
| *PDLIM3* | 5.23836092 | 2.76497566 | 0.00031338 | 0.00587197 |
| *SLC30A8* | 5.23765731 | 0.0876648 | 0.0001449 | 0.00349219 |
| *ENSGALG00000007028* | 5.23753447 | 1.59174116 | 0.00749998 | 0.04894014 |
| *ENSGALG00000011086* | 5.23464061 | 6.64015421 | 2.68E-06 | 0.00016293 |
| *UMODL1* | 5.22936227 | 0.2058066 | 0.00333672 | 0.02881845 |
| *ENSGALG00000045796* | 5.2238323 | 0.16822446 | 0.00424127 | 0.03395653 |
| *ENSGALG00000034144* | 5.22212266 | 0.71660899 | 0.00518689 | 0.03873314 |
| *ENSGALG00000014585* | 5.22078862 | 1.29217143 | 0.00111383 | 0.0143379 |
| *PTPRC* | 5.21384456 | 1.67420698 | 0.00026484 | 0.00524017 |
| *ENSGALG00000005349* | 5.21276945 | 1.90074407 | 0.00248217 | 0.02382314 |
| *ENSGALG00000000164* | 5.2073522 | 2.49690559 | 0.00074993 | 0.01090362 |
| *AMPD1* | 5.19567665 | 1.37754358 | 0.00177825 | 0.01915989 |
| *TRPC6* | 5.17760199 | 0.37139199 | 0.00742556 | 0.0486312 |
| *ENSGALG00000036544* | 5.17711556 | 1.5819103 | 8.17E-05 | 0.00231534 |
| *ANTXR1* | 5.17481446 | 0.43104797 | 0.00513267 | 0.03856183 |
| *ENSGALG00000038375* | 5.17035873 | 0.69353263 | 0.00098858 | 0.01326885 |
| *STK31* | 5.16198841 | 0.74122008 | 0.00295857 | 0.02672726 |
| *ENSGALG00000017168* | 5.15858713 | 0.70409038 | 0.00066176 | 0.0100557 |

| | | | | |
|---|---|---|---|---|
| *NSG2* | 5.15600429 | 3.4632162 | 4.12E-06 | 0.00023142 |
| *DUSP27* | 5.14817982 | -0.3450429 | 0.00584838 | 0.04184788 |
| *CACNA1S* | 5.1473573 | 2.46443368 | 1.76E-05 | 0.00073244 |
| *ENSGALG00000012382* | 5.14185332 | 0.72742062 | 0.00701471 | 0.0472333 |
| *LUM* | 5.13758551 | 6.56395605 | 1.38E-05 | 0.00060408 |
| *ENSGALG00000029827* | 5.13464783 | 0.58148049 | 0.00110169 | 0.01422041 |
| *ENSGALG00000029446* | 5.11926413 | 2.76463367 | 2.05E-05 | 0.00081831 |
| *ENSGALG00000016805* | 5.11261005 | 0.47960351 | 0.00292154 | 0.02646034 |
| *EPHA4* | 5.10508469 | 0.82779077 | 0.00492002 | 0.03757683 |
| *ENSGALG00000038824* | 5.10417744 | 3.03614401 | 0.00730326 | 0.04825248 |
| *ANO2* | 5.10317585 | 1.2821498 | 0.0065485 | 0.04508105 |
| *ABI3BP* | 5.09646113 | 2.84253203 | 0.00018084 | 0.00406083 |
| *ENSGALG00000002570* | 5.09452171 | 1.53319793 | 3.50E-05 | 0.00121811 |
| *ENSGALG00000006717* | 5.09156096 | 1.97369458 | 0.00123477 | 0.01533656 |
| *ENSGALG00000045739* | 5.08788386 | 0.91513034 | 0.00106691 | 0.01391082 |
| *ENSGALG00000011322* | 5.07300062 | 1.32122358 | 0.00376844 | 0.03143405 |
| *ENSGALG00000037911* | 5.07213611 | 0.53806581 | 0.00104746 | 0.01373343 |
| *ENSGALG00000015568* | 5.06807437 | 2.83778729 | 3.02E-05 | 0.00109141 |
| *ENSGALG00000041031* | 5.06719147 | 0.09042446 | 0.00049079 | 0.00809229 |
| *PCBP3* | 5.06653843 | -0.1871065 | 0.00462904 | 0.03613772 |
| *ENSGALG00000008439* | 5.05705846 | 4.57417393 | 0.00012822 | 0.0032147 |
| *ENSGALG00000037439* | 5.05617183 | 0.31794013 | 0.0071884 | 0.04784618 |
| *ENSGALG00000030765* | 5.05264302 | 1.89393014 | 4.29E-05 | 0.00140169 |
| *ENSGALG00000014999* | 5.04818696 | 6.7327018 | 6.38E-11 | 2.74E-08 |
| *COL14A1* | 5.03593697 | 8.68683577 | 2.03E-05 | 0.00081553 |
| *AKAP6* | 5.01937322 | 2.4392398 | 2.50E-05 | 0.00095231 |
| *TXK* | 5.01211043 | 0.6863144 | 0.00634188 | 0.04419635 |
| *ADAMTS18* | 5.00800932 | 3.33205605 | 0.00046574 | 0.00782523 |
| *ADGRG2* | 4.99459878 | 2.29602521 | 1.93E-05 | 0.00078753 |
| *POPDC2* | 4.99128865 | 2.13277189 | 1.29E-05 | 0.0005758 |
| *ENSGALG00000042664* | 4.98877754 | 3.33836181 | 1.52E-05 | 0.00065441 |
| *XKRX* | 4.98678004 | 1.85245807 | 0.00038125 | 0.00670189 |
| *RGS7* | 4.98521604 | 0.66774357 | 0.00306784 | 0.02734843 |
| *TPPP* | 4.97726864 | 3.58865028 | 4.47E-07 | 3.93E-05 |
| *ASZ1* | 4.96506485 | 1.47553076 | 0.00499576 | 0.03785485 |
| *ITGB1BP2* | 4.96100028 | 1.92183748 | 7.13E-06 | 0.00035395 |
| *ARHGAP15* | 4.95912886 | 2.82317999 | 0.00033499 | 0.00613076 |
| *ENSGALG00000023812* | 4.95909081 | 0.33213196 | 0.00053984 | 0.00866861 |
| *GRM8* | 4.95522011 | 0.22338995 | 0.00730271 | 0.04825248 |
| *ENSGALG00000021627* | 4.95494045 | 1.76168225 | 1.71E-05 | 0.00071869 |

| | | | | |
|---|---|---|---|---|
| *CSMD3* | 4.95271367 | 3.27280137 | 0.0009707 | 0.01307854 |
| *PENK* | 4.92364868 | 5.55479802 | 0.00073266 | 0.01077753 |
| *MYBPC1* | 4.91818372 | 3.48200211 | 0.00019677 | 0.00429578 |
| *ENSGALG00000031737* | 4.90744611 | 2.37857816 | 0.00734442 | 0.04834876 |
| *OVCH2* | 4.90716336 | 1.3121429 | 0.0019933 | 0.02061348 |
| *ENSGALG00000034387* | 4.90516736 | 3.20863417 | 7.21E-10 | 2.04E-07 |
| *UPB1* | 4.90097053 | 0.73492331 | 0.00534352 | 0.03951293 |
| *ENSGALG00000010224* | 4.89894628 | 1.9389992 | 4.94E-05 | 0.00155347 |
| *ENSGALG00000013575* | 4.89686839 | -0.5531585 | 0.00162808 | 0.01830881 |
| *PPP1R1C* | 4.89637309 | 3.06135896 | 2.61E-05 | 0.00097952 |
| *MARCHF3* | 4.88863937 | -0.056852 | 0.00013908 | 0.00338904 |
| *ANGPT1* | 4.88213219 | 4.28104494 | 2.79E-05 | 0.00102498 |
| *RAMP3* | 4.87977787 | 2.0049634 | 6.54E-07 | 5.17E-05 |
| *ENSGALG00000035309* | 4.87699545 | 2.29330683 | 1.99E-06 | 0.00012874 |
| *MYCT1* | 4.87174622 | 1.23266462 | 0.0019163 | 0.02010702 |
| *NKAIN4* | 4.86402191 | 5.21166571 | 1.96E-05 | 0.00079851 |
| *TBX2* | 4.85754147 | 5.29543068 | 2.10E-15 | 3.31E-12 |
| *ENSGALG00000043880* | 4.83405978 | 2.03725698 | 8.07E-05 | 0.00229156 |
| *MGLL* | 4.80372671 | 3.68898264 | 3.59E-09 | 7.81E-07 |
| *MALRD1* | 4.800178 | 2.27308648 | 0.00275541 | 0.02547767 |
| *VWA3B* | 4.79532429 | 2.28764742 | 0.00088698 | 0.01223016 |
| *LMO2* | 4.79425207 | 5.42375089 | 3.20E-12 | 2.16E-09 |
| *ENSGALG00000044563* | 4.79131325 | -0.5399999 | 0.00081016 | 0.01149578 |
| *ENSGALG00000016292* | 4.79093131 | 3.2146254 | 6.32E-05 | 0.00187474 |
| *ADAMTS5* | 4.75874877 | 2.73467718 | 1.64E-05 | 0.00069517 |
| *RAMP1* | 4.75152647 | 3.2762721 | 6.92E-07 | 5.38E-05 |
| *ENSGALG00000036488* | 4.74665758 | -0.2125602 | 0.00510828 | 0.03845653 |
| *ANO1* | 4.74388643 | 1.39672539 | 0.00095762 | 0.01296318 |
| *ENSGALG00000008127* | 4.7342931 | 3.90502345 | 5.13E-08 | 6.92E-06 |
| *JAG2* | 4.73220145 | 2.90886319 | 4.56E-05 | 0.00147159 |
| *COL4A2* | 4.72599397 | 2.88011435 | 0.00029141 | 0.0056079 |
| *PIK3R1* | 4.72371415 | 2.8832339 | 5.80E-06 | 0.00029812 |
| *VWF* | 4.72097054 | 3.75286937 | 0.00095355 | 0.01294557 |
| *MAP3K8* | 4.71466047 | 0.38261909 | 0.00333359 | 0.02880893 |
| *EPAS1* | 4.71023385 | 3.98508536 | 7.41E-11 | 2.99E-08 |
| *ENSGALG00000036838* | 4.71015891 | 0.42482367 | 0.00470619 | 0.03646745 |
| *GABBR2* | 4.70684556 | 1.80189608 | 0.00163331 | 0.01833844 |
| *FNDC1* | 4.70672878 | 3.68923676 | 0.00016954 | 0.00387473 |
| *ENSGALG00000039966* | 4.70156847 | 3.83830807 | 0.00011123 | 0.00289786 |
| *ENSGALG00000045305* | 4.70095595 | -0.2562123 | 0.00389632 | 0.03210324 |

| | | | | |
|---|---|---|---|---|
| *MEF2C* | 4.69984954 | 8.26254691 | 7.12E-07 | 5.50E-05 |
| *COL6A2* | 4.69771817 | 6.51915674 | 5.57E-06 | 0.00028951 |
| *IGSF6* | 4.68885829 | 1.85848314 | 0.00020861 | 0.00446484 |
| *CASQ2* | 4.68525902 | 2.7742137 | 0.00113705 | 0.01442674 |
| *WNT11* | 4.68264133 | 4.45083168 | 6.04E-05 | 0.001805 |
| *TLR7* | 4.67976314 | 1.48724802 | 0.00045182 | 0.00768256 |
| *COL3A1* | 4.6759091 | 9.6614315 | 4.85E-10 | 1.50E-07 |
| *STEAP4* | 4.67489826 | 1.71763954 | 0.00387901 | 0.03201655 |
| *ENSGALG00000033466* | 4.67222418 | 1.07215157 | 0.00736405 | 0.04843295 |
| *LAPTM4B* | 4.66546674 | -0.0717689 | 0.00580485 | 0.04168591 |
| *ENSGALG00000013223* | 4.66536299 | 5.0709943 | 0.00176745 | 0.01912033 |
| *PKIB* | 4.65862905 | 2.48217026 | 2.54E-07 | 2.57E-05 |
| *EGFL6* | 4.65690027 | 7.30063666 | 2.21E-05 | 0.0008591 |
| *ENSGALG00000039354* | 4.65048725 | 1.66343586 | 0.00113185 | 0.01440599 |
| *ENSGALG00000045956* | 4.64686833 | 2.93277354 | 1.23E-06 | 8.70E-05 |
| *CXCL12* | 4.64177243 | 5.98930494 | 1.28E-10 | 4.78E-08 |
| *ENSGALG00000042136* | 4.6407775 | 1.7013521 | 0.00149494 | 0.01737791 |
| *COLEC11* | 4.6376952 | -0.00958 | 0.00127983 | 0.01573738 |
| *ENSGALG00000029899* | 4.6358971 | 4.90674453 | 9.72E-10 | 2.64E-07 |
| *ENSGALG00000032701* | 4.63123356 | 1.07540656 | 0.00406229 | 0.03308531 |
| *ATP2C2* | 4.62923349 | 0.22623573 | 0.00610739 | 0.04307143 |
| *PODXL* | 4.6275464 | 4.96150781 | 0.00018477 | 0.00410948 |
| *UTS2R* | 4.62032051 | 3.38877317 | 4.10E-05 | 0.00136276 |
| *EGR1* | 4.61907005 | 5.09538752 | 2.39E-06 | 0.00014763 |
| *HHEX* | 4.61827707 | 4.14097789 | 2.32E-08 | 3.69E-06 |
| *CLDN5* | 4.61804839 | 1.58325978 | 0.00011004 | 0.00287751 |
| *ENSGALG00000032740* | 4.61634456 | 2.20938881 | 0.00033848 | 0.00618662 |
| *ENSGALG00000010901* | 4.61621747 | 2.01898757 | 0.00239114 | 0.02333769 |
| *ENSGALG00000040010* | 4.61324481 | 2.53183519 | 8.80E-06 | 0.00042368 |
| *CSRP2* | 4.61168989 | 6.24945012 | 2.13E-10 | 7.37E-08 |
| *ENSGALG00000044238* | 4.60682812 | 0.46127963 | 0.00592966 | 0.04228169 |
| *ENSGALG00000030728* | 4.6041112 | 1.7598513 | 0.00016392 | 0.00380788 |
| *RYR2* | 4.60018048 | 3.84842429 | 0.00188035 | 0.01992612 |
| *ENSGALG00000038950* | 4.59559724 | 2.05116059 | 0.00554477 | 0.04045481 |
| *ENSGALG00000005540* | 4.58869263 | 1.83830373 | 0.00011579 | 0.00297842 |
| *PDE1A* | 4.58837645 | 1.1142241 | 0.00388422 | 0.03204084 |
| *CYBB* | 4.58518562 | -0.3248269 | 0.0074102 | 0.04855583 |
| *ATF3* | 4.57703668 | 1.80802049 | 0.00018504 | 0.00410948 |
| *ENSGALG00000043810* | 4.5767975 | -0.1396777 | 0.00384184 | 0.03185841 |
| *DMGDH* | 4.56954139 | 2.26515265 | 3.27E-05 | 0.00116966 |

| | | | | |
|---|---|---|---|---|
| *ENSGALG00000036726* | 4.5650107 | 5.08369232 | 7.10E-11 | 2.96E-08 |
| *RGS4* | 4.55785993 | 2.69478848 | 2.52E-07 | 2.57E-05 |
| *ENSGALG00000032195* | 4.55730233 | 2.31764374 | 0.00120211 | 0.01500987 |
| *ENSGALG00000031398* | 4.55108192 | 1.63137175 | 3.04E-06 | 0.00017947 |
| *ATP6V0D2* | 4.54022534 | 5.01838347 | 0.00141427 | 0.01680306 |
| *SERPINF1* | 4.53633294 | 9.09043534 | 1.53E-06 | 0.00010332 |
| *GATA3* | 4.53196174 | 1.38439592 | 0.00160017 | 0.01813914 |
| *GPR158* | 4.5290697 | 0.91060129 | 0.00737934 | 0.04849488 |
| *RHOBTB1* | 4.5246025 | 2.4229475 | 0.00011233 | 0.00292108 |
| *ENSGALG00000031597* | 4.51824346 | 8.5794544 | 6.14E-07 | 4.93E-05 |
| *KIF6* | 4.49787261 | 1.11664302 | 0.00323826 | 0.02836633 |
| *CD44* | 4.49737927 | 6.51827104 | 2.36E-09 | 5.38E-07 |
| *FER1L6* | 4.4948297 | 1.0560321 | 0.00316737 | 0.02790668 |
| *ENSGALG00000045429* | 4.49473247 | 0.33203046 | 0.00337819 | 0.02903481 |
| *FMN2* | 4.49220702 | 1.67339944 | 0.00223825 | 0.02223678 |
| *CCDC141* | 4.49162153 | 3.22523618 | 0.0003313 | 0.00609186 |
| *ENSGALG00000012801* | 4.48640775 | 2.64807 | 5.97E-08 | 7.89E-06 |
| *KCTD12B* | 4.47767708 | 3.54875449 | 4.99E-05 | 0.00156407 |
| *EGF* | 4.46579289 | 0.65956992 | 0.00338996 | 0.02907939 |
| *MSX2* | 4.46528239 | 0.91752423 | 0.00037766 | 0.00666171 |
| *ENSGALG00000034133* | 4.46472159 | -0.0185603 | 0.00356528 | 0.03020243 |
| *GRIA4* | 4.46231014 | 3.9114567 | 3.14E-05 | 0.00112595 |
| *ENSGALG00000039688* | 4.46050968 | 2.15629822 | 0.00048682 | 0.00806444 |
| *ENSGALG00000021395* | 4.44695084 | 2.25412451 | 0.00029356 | 0.00562731 |
| *PEX5L* | 4.42871506 | 1.00941445 | 0.00316558 | 0.02790668 |
| *CACNG4* | 4.42300231 | 1.85881779 | 0.00263593 | 0.02474486 |
| *TMPRSS7* | 4.41454976 | 2.63552695 | 0.00452031 | 0.03554683 |
| *SLA* | 4.41064775 | 0.84602123 | 0.00014347 | 0.00346954 |
| *FZD4* | 4.40762613 | 4.27376104 | 5.58E-07 | 4.64E-05 |
| *COL8A1* | 4.40756859 | 4.04107745 | 2.14E-06 | 0.00013527 |
| *NELL1* | 4.40637632 | 0.99808178 | 0.00429519 | 0.03425254 |
| *SYNPO2L* | 4.40376516 | 0.95128851 | 0.00682586 | 0.04626997 |
| *INHBA* | 4.39718768 | 3.45573363 | 1.21E-06 | 8.63E-05 |
| *MRVI1* | 4.39377028 | 2.03751547 | 0.00024863 | 0.00501765 |
| *PTPRZ1* | 4.38805435 | 2.54034641 | 0.0008296 | 0.0116316 |
| *SRL* | 4.38751047 | 4.45304595 | 1.39E-07 | 1.60E-05 |
| *EBF2* | 4.38456533 | 4.96332639 | 4.99E-06 | 0.00026634 |
| *MYO18B* | 4.36809726 | 0.89525662 | 0.00307962 | 0.02741814 |
| *NRN1* | 4.35790511 | 2.34456859 | 9.17E-06 | 0.00043366 |
| *SYNPO2* | 4.35008167 | 4.48494914 | 1.34E-08 | 2.37E-06 |

| | | | | |
|---|---|---|---|---|
| *KCNJ15* | 4.34257075 | 1.08514446 | 0.00164207 | 0.0183639 |
| *ENSGALG00000041375* | 4.3342935 | 1.39553307 | 0.00170876 | 0.01876853 |
| *OCA2* | 4.32513602 | 2.79843529 | 0.00052287 | 0.00849256 |
| *PDGFD* | 4.3219959 | 7.69391014 | 1.53E-07 | 1.71E-05 |
| *PDZRN4* | 4.31784079 | 3.62997049 | 0.00034507 | 0.00628267 |
| *ENSGALG00000012872* | 4.31291111 | -0.2087502 | 0.00421404 | 0.03381513 |
| *ESRP2* | 4.31286151 | 2.95674601 | 0.00510357 | 0.03845282 |
| *TNFSF15* | 4.30941881 | 1.16974297 | 0.00067092 | 0.01012962 |
| *SOAT1* | 4.30933801 | 0.56604837 | 0.00261243 | 0.02464407 |
| *ENSGALG00000004274* | 4.30672062 | 5.4729936 | 7.07E-05 | 0.00205767 |
| *ENSGALG00000008629* | 4.29693527 | 2.37968721 | 0.00129451 | 0.01582836 |
| *COL17A1* | 4.29624627 | 0.79902573 | 0.00709744 | 0.04755698 |
| *MUSK* | 4.2929857 | 1.00001573 | 0.00270202 | 0.02520627 |
| *KCNT2* | 4.29160933 | 2.41911328 | 0.00078531 | 0.01125606 |
| *ENSGALG00000045548* | 4.28944815 | 6.7808346 | 6.04E-10 | 1.74E-07 |
| *LIMCH1* | 4.28900629 | 3.2807973 | 2.50E-08 | 3.85E-06 |
| *FAM198B* | 4.28450713 | 2.52465591 | 6.92E-05 | 0.00202188 |
| *GLIPR2* | 4.28200844 | 5.00108623 | 1.65E-09 | 4.25E-07 |
| *GDPD1* | 4.2750932 | 0.1029569 | 9.48E-05 | 0.00258927 |
| *LAPTM5* | 4.274291 | 4.16406958 | 2.67E-09 | 5.90E-07 |
| *EPHA3* | 4.2601432 | 6.19732376 | 5.74E-10 | 1.69E-07 |
| *NCF4* | 4.26000028 | 1.62081961 | 0.00030723 | 0.00578739 |
| *CAMK1G* | 4.2581459 | -0.2140329 | 0.00333145 | 0.02880806 |
| *ENSGALG00000033925* | 4.25775414 | 1.27165272 | 0.00167749 | 0.01856517 |
| *GPR137B* | 4.25336213 | 3.32837447 | 1.75E-05 | 0.00073244 |
| *JAKMIP1* | 4.24917204 | 4.64882962 | 1.60E-06 | 0.00010475 |
| *ENSGALG00000038316* | 4.2324481 | 2.67139493 | 3.61E-07 | 3.38E-05 |
| *ESAM* | 4.230334 | 3.02206355 | 5.68E-05 | 0.00171968 |
| *MXRA8* | 4.22432555 | 6.87206786 | 3.53E-11 | 1.64E-08 |
| *ENSGALG00000004167* | 4.21926838 | 0.26319265 | 0.0045659 | 0.03576624 |
| *KCNJ11* | 4.21747349 | 1.63485655 | 0.0008168 | 0.01154372 |
| *ENSGALG00000032534* | 4.21040169 | 2.90815463 | 0.00207433 | 0.02115759 |
| *GABRB2* | 4.20980684 | 0.4344085 | 0.00692224 | 0.04669954 |
| *ADCY8* | 4.20914866 | 1.67235029 | 0.0020593 | 0.02109548 |
| *EFCAB6* | 4.19085741 | 2.19646923 | 0.00069297 | 0.01035215 |
| *CYTH4* | 4.18995887 | 2.17945381 | 0.00102216 | 0.01350922 |
| *ENSGALG00000028273* | 4.17831845 | 6.6113368 | 3.39E-06 | 0.00019661 |
| *CXCL14* | 4.17479836 | 4.96237168 | 0.0001536 | 0.00363864 |
| *ENSGALG00000039432* | 4.17378595 | 2.01977059 | 4.86E-06 | 0.00026197 |
| *TFPI2* | 4.16763963 | 1.83183477 | 0.00360148 | 0.03038169 |

| | | | | |
|---|---|---|---|---|
| *DLL1* | 4.1662398 | 1.16023021 | 0.00183597 | 0.01960947 |
| *MYOZ1* | 4.16357198 | 2.71129448 | 4.13E-05 | 0.00136573 |
| *CLDN1* | 4.15611302 | 3.54413383 | 0.00170136 | 0.01871623 |
| *TRPM5* | 4.15476793 | 0.94203599 | 0.00276448 | 0.02552814 |
| *RSPO1* | 4.15392704 | 1.03107421 | 0.00565667 | 0.04103843 |
| *CELF2* | 4.14560826 | 2.52170733 | 0.00239506 | 0.02335137 |
| *TRAF3IP3* | 4.14226617 | 0.04320283 | 0.00290115 | 0.02642726 |
| *ADGRL4* | 4.14048439 | 1.22793813 | 0.00286153 | 0.02620201 |
| *ENSGALG00000011283* | 4.13687785 | 2.21090545 | 0.0022885 | 0.02261128 |
| *USH1C* | 4.1341408 | 1.34884485 | 0.00345799 | 0.02951833 |
| *RBM38* | 4.1288058 | 5.65258289 | 1.36E-10 | 4.95E-08 |
| *CNRIP1* | 4.12269941 | 1.97375707 | 2.43E-07 | 2.49E-05 |
| *CLVS2* | 4.12263856 | 0.54572572 | 0.00363344 | 0.03052394 |
| *ENSGALG00000005204* | 4.12067878 | 3.34548779 | 3.30E-06 | 0.00019377 |
| *ENSGALG00000044973* | 4.11856165 | 1.06151759 | 0.00121094 | 0.01510688 |
| *ENSGALG00000042416* | 4.1172267 | 0.36140026 | 0.00244364 | 0.02362962 |
| *ENSGALG00000031149* | 4.11582919 | 4.1578171 | 2.15E-07 | 2.27E-05 |
| *ENSGALG00000042638* | 4.10592072 | 2.29825902 | 0.00439338 | 0.03481965 |
| *CALB2* | 4.10425326 | 1.92205941 | 0.00015381 | 0.00363864 |
| *FHL3* | 4.10405778 | 7.02511885 | 4.94E-13 | 4.99E-10 |
| *ENSGALG00000014412* | 4.09009656 | 4.93667143 | 0.000275 | 0.00538089 |
| *ABCC9* | 4.07668807 | 2.88970786 | 0.00166957 | 0.01855551 |
| *TTC29* | 4.0715459 | 1.29927188 | 0.00674144 | 0.04591343 |
| *TNN* | 4.06887446 | 4.27393415 | 0.00037489 | 0.00663772 |
| *ENSGALG00000007917* | 4.06171681 | 2.29920607 | 0.00170771 | 0.01876853 |
| *ENSGALG00000004155* | 4.0612586 | 3.12829372 | 0.00046285 | 0.00781384 |
| *ST18* | 4.05989369 | 2.19297351 | 0.00257978 | 0.02446698 |
| *VSTM4* | 4.05789736 | 5.40530248 | 1.69E-09 | 4.28E-07 |
| *EMILIN2* | 4.05420968 | 3.8385415 | 0.00017932 | 0.00403307 |
| *ENSGALG00000002207* | 4.05392635 | 3.1143834 | 5.70E-05 | 0.00172183 |
| *ENSGALG00000035525* | 4.05270329 | 0.00566948 | 0.00713317 | 0.04771297 |
| *GATA2* | 4.05178908 | 2.67087099 | 5.00E-05 | 0.00156407 |
| *ITGBL1* | 4.04536652 | 2.29636857 | 0.0002282 | 0.00476856 |
| *GREB1* | 4.04194267 | 2.34370967 | 0.00089758 | 0.01232824 |
| *TMEM229B* | 4.03680229 | 3.4467362 | 2.16E-09 | 5.18E-07 |
| *CPA6* | 4.0237615 | 2.93990112 | 0.00339157 | 0.02907939 |
| *PAX9* | 4.02344199 | 1.16300439 | 2.14E-05 | 0.00083758 |
| *CERKL* | 4.01523441 | 1.46049936 | 0.00241353 | 0.02343459 |
| *PPP2R2C* | 4.01475243 | 1.73583477 | 0.00087724 | 0.01211948 |
| *ENSGALG00000015147* | 4.01256334 | 1.72558659 | 0.00773625 | 0.04999758 |

| | | | | |
|---|---|---|---|---|
| *ABCA1* | 4.000273 | 0.49375382 | 0.00385833 | 0.03193799 |
| *CBFA2T3* | 3.99988061 | 3.37737308 | 2.10E-07 | 2.24E-05 |
| *ANO4* | 3.99352928 | 1.55785195 | 0.00576759 | 0.0415237 |
| *TAGLN3* | 3.98994666 | 4.31292692 | 1.60E-06 | 0.00010475 |
| *ENSGALG00000026613* | 3.98613079 | 3.440883 | 1.93E-05 | 0.00078753 |
| *ENSGALG00000035946* | 3.98281221 | 2.00678693 | 0.00090602 | 0.01239593 |
| *ENSGALG00000005473* | 3.97971522 | 1.72802551 | 9.38E-05 | 0.00257574 |
| *CREB5* | 3.97661477 | 1.4297992 | 0.00193839 | 0.02020814 |
| *CIITA* | 3.972347 | 0.76428569 | 0.00657287 | 0.04520484 |
| *ENSGALG00000032272* | 3.9655858 | 0.93929751 | 0.00274182 | 0.02543505 |
| *AMN* | 3.96463574 | 0.44384902 | 0.00188577 | 0.01995358 |
| *FAM69C* | 3.96184296 | 3.8501278 | 1.65E-05 | 0.00069855 |
| *ENSGALG00000036711* | 3.95870112 | 6.34943004 | 2.33E-09 | 5.38E-07 |
| *IKZF1* | 3.95758367 | 1.73263152 | 0.00027147 | 0.00533843 |
| *ENSGALG00000004360* | 3.95489334 | 1.7464847 | 0.00026467 | 0.00524017 |
| *ENSGALG00000040422* | 3.95159715 | 2.22289468 | 0.00420948 | 0.03379764 |
| *BATF3* | 3.95095066 | 0.23688328 | 0.00585107 | 0.04184788 |
| *ANXA1* | 3.9453885 | 4.8452388 | 3.07E-10 | 1.03E-07 |
| *ENSGALG00000030747* | 3.94004263 | -0.0604364 | 0.00287977 | 0.02630087 |
| *GATA6* | 3.93753549 | -0.1503861 | 0.00156875 | 0.01791213 |
| *FBP2* | 3.932767 | 0.94916214 | 0.00062889 | 0.00972817 |
| *ENSGALG00000005638* | 3.92659173 | 1.82808588 | 0.00081481 | 0.01152709 |
| *4930578C19RIK* | 3.91430395 | 3.54635683 | 0.0002249 | 0.00473467 |
| *ENSGALG00000043759* | 3.90955198 | -0.3315478 | 0.0027679 | 0.02554304 |
| *WFDC1* | 3.9059698 | 5.0030949 | 2.07E-05 | 0.0008208 |
| *PALMD* | 3.9003705 | 3.11921501 | 2.10E-05 | 0.00082898 |
| *RASGEF1C* | 3.89939127 | 0.23627925 | 0.00647158 | 0.04474752 |
| *ENSGALG00000034632* | 3.89128823 | -0.6322287 | 0.00608502 | 0.04295649 |
| *NPY* | 3.8886805 | 0.19120579 | 0.00582398 | 0.04173855 |
| *ENSGALG00000029256* | 3.88845098 | 0.89123353 | 0.00428127 | 0.03419939 |
| *FHL5* | 3.88098773 | 3.47912953 | 0.00010338 | 0.00274395 |
| *SCARA5* | 3.87983306 | 2.63553976 | 6.54E-05 | 0.00193013 |
| *KIT* | 3.87827613 | 2.65067503 | 0.00307863 | 0.02741814 |
| *ENSGALG00000004521* | 3.87432265 | 6.5519227 | 1.58E-09 | 4.13E-07 |
| *HECW1* | 3.87276909 | 3.00937725 | 0.00027495 | 0.00538089 |
| *ENSGALG00000032682* | 3.8673451 | 0.1287974 | 0.00065031 | 0.0099459 |
| *HDAC9* | 3.86190062 | 2.77322317 | 0.00136759 | 0.01642389 |
| *ENSGALG00000001136* | 3.85509313 | 3.55123086 | 8.03E-05 | 0.00228458 |
| *DOK5* | 3.85383215 | 2.19208679 | 0.00024336 | 0.00497516 |
| *DLX1* | 3.85234047 | 1.29490804 | 0.00013369 | 0.00329831 |

| | | | | |
|---|---|---|---|---|
| *ZPLD1* | 3.84492123 | 0.51567893 | 0.00723872 | 0.04802279 |
| *USP13* | 3.84315259 | 1.93716267 | 0.00021113 | 0.00449655 |
| *ENSGALG00000033433* | 3.83834988 | 3.50072969 | 0.00029768 | 0.00567549 |
| *ARHGAP28* | 3.8375024 | 0.48021063 | 0.00501429 | 0.03795459 |
| *PTPRB* | 3.83612965 | 2.59812024 | 0.00273211 | 0.0254284 |
| *ALDH1A3* | 3.83410947 | 4.38252594 | 9.15E-06 | 0.00043366 |
| *GDF2* | 3.83250518 | 2.41517003 | 0.00354779 | 0.03010835 |
| *WWTR1* | 3.83212254 | 0.18193708 | 0.00032826 | 0.00605924 |
| *ENSGALG00000028304* | 3.82643874 | 1.17866754 | 0.00638631 | 0.0443748 |
| *ENSGALG00000041344* | 3.82529746 | 4.87399976 | 1.31E-05 | 0.00058075 |
| *NTRK3* | 3.82301072 | 2.35827182 | 0.00018282 | 0.00408119 |
| *ENSGALG00000043234* | 3.82210535 | 9.87412204 | 5.37E-06 | 0.00028221 |
| *HGF* | 3.81814685 | 1.84794915 | 0.00326055 | 0.02848351 |
| *PLXNA2* | 3.81722447 | 2.06917165 | 0.00240363 | 0.02340069 |
| *ENSGALG00000027624* | 3.81383676 | 1.31822375 | 0.00468058 | 0.03638247 |
| *TNFRSF18* | 3.81178712 | 1.15047262 | 0.00411307 | 0.03328276 |
| *PROX1* | 3.80510888 | 1.47249878 | 0.00274942 | 0.02547215 |
| *ENSGALG00000036373* | 3.79320128 | -0.1891799 | 0.00338796 | 0.02907939 |
| *ENSGALG00000000433* | 3.79245361 | 1.10715924 | 0.00022561 | 0.00474254 |
| *ENSGALG00000007526* | 3.77825637 | 4.20362354 | 1.28E-06 | 9.02E-05 |
| *ENSGALG00000016324* | 3.76697101 | 2.77358589 | 1.32E-05 | 0.00058399 |
| *VCAM1* | 3.75274327 | 4.29282269 | 0.00235384 | 0.02309272 |
| *BHLHE22* | 3.74839891 | 1.13892277 | 0.00010056 | 0.00270456 |
| *HS6ST1* | 3.73687966 | 5.18241561 | 3.10E-08 | 4.52E-06 |
| *ENSGALG00000042810* | 3.73585316 | 4.08606753 | 0.00012719 | 0.00319601 |
| *EMX2* | 3.72933047 | 1.61859096 | 0.00413508 | 0.03337668 |
| *ENSGALG00000033942* | 3.7286534 | -0.1982434 | 0.00663438 | 0.04540716 |
| *SLCO2B1* | 3.72653242 | 3.62761697 | 0.00034629 | 0.0062888 |
| *GDF10* | 3.72182595 | 0.39844971 | 0.0035643 | 0.03020243 |
| *ENSGALG00000021017* | 3.7168996 | 0.79069558 | 0.00189047 | 0.0199884 |
| *TMEM268* | 3.7161392 | 2.12459371 | 0.00148063 | 0.01729689 |
| *BCHE* | 3.69705011 | 2.11061733 | 0.00029129 | 0.0056079 |
| *ECSCR* | 3.69070147 | 1.98972023 | 0.0019851 | 0.02057375 |
| *ENSGALG00000043073* | 3.68964089 | 0.28995363 | 0.00232156 | 0.02284333 |
| *EBF3* | 3.67838292 | 5.46278152 | 2.55E-09 | 5.72E-07 |
| *WNT9A* | 3.66990918 | 2.1477072 | 0.0033156 | 0.02876716 |
| *BLNK* | 3.66552758 | 3.46066032 | 2.28E-05 | 0.0008779 |
| *ENSGALG00000011206* | 3.65905469 | 1.14259685 | 0.00717544 | 0.04784329 |
| *GUCY1A2* | 3.65076937 | 2.8250307 | 0.00061839 | 0.00961357 |
| *EPHA5* | 3.63400785 | 2.61811731 | 7.01E-05 | 0.00204539 |

| | | | | |
|---|---|---|---|---|
| *P2RY6* | 3.63251416 | 1.59795588 | 0.00289749 | 0.0264116 |
| *ENSGALG00000042020* | 3.63162267 | 7.38670039 | 1.55E-07 | 1.73E-05 |
| *ENSGALG00000012377* | 3.62630175 | 0.99464032 | 0.00248194 | 0.02382314 |
| *CD200* | 3.62587792 | 6.12000752 | 1.40E-08 | 2.45E-06 |
| *SLC2A12* | 3.62080773 | 1.50908086 | 0.00120022 | 0.01499959 |
| *H1F0* | 3.61587216 | 3.4050323 | 2.23E-08 | 3.58E-06 |
| *ENSGALG00000015833* | 3.61353481 | 0.9514161 | 0.00335112 | 0.02888985 |
| *SUSD3* | 3.61351926 | 3.29335375 | 6.62E-06 | 0.00033215 |
| *ENSGALG00000002431* | 3.60913317 | 3.76158335 | 0.00118754 | 0.0148937 |
| *RD3L* | 3.60809676 | 1.92263626 | 0.00429048 | 0.0342361 |
| *ADGRB1* | 3.60128726 | 1.57668631 | 0.00477806 | 0.03693724 |
| *ENSGALG00000010722* | 3.59811293 | 5.92706151 | 5.61E-06 | 0.00029057 |
| *VIPR2* | 3.59758283 | 0.61465349 | 0.00439329 | 0.03481965 |
| *ENSGALG00000040445* | 3.59204376 | 2.56271478 | 0.00102247 | 0.01350922 |
| *ENSGALG00000017347* | 3.5919998 | 5.54533724 | 0.00012567 | 0.00317463 |
| *ENSGALG00000031593* | 3.59039327 | 8.67590373 | 1.16E-07 | 1.40E-05 |
| *TNFRSF19* | 3.58710956 | 1.64964696 | 0.00261474 | 0.02464407 |
| *CRIP1* | 3.58692646 | 4.31740728 | 1.04E-05 | 0.0004817 |
| *ENSGALG00000033694* | 3.58433816 | 5.60806759 | 4.56E-07 | 3.97E-05 |
| *ENSGALG00000041611* | 3.57731189 | 0.57973259 | 0.00400877 | 0.03269002 |
| *FMOD* | 3.57226341 | 4.23847709 | 4.57E-07 | 3.97E-05 |
| *ZFP106* | 3.56169113 | 4.92046316 | 4.17E-08 | 5.78E-06 |
| *DUSP6* | 3.55856054 | 4.16100312 | 6.91E-07 | 5.38E-05 |
| *ENSGALG00000012821* | 3.55641476 | 5.38472127 | 1.84E-11 | 9.30E-09 |
| *ENSGALG00000033428* | 3.55207801 | 2.58347673 | 0.00012045 | 0.00306301 |
| *TMEM248* | 3.54769787 | 0.49363555 | 0.00188451 | 0.01995358 |
| *TSPO2* | 3.54605999 | 2.88217585 | 0.00013772 | 0.00337302 |
| *SLC1A2* | 3.54504781 | 1.78828486 | 0.0001031 | 0.00274395 |
| *ENSGALG00000045042* | 3.54435211 | 0.15065594 | 0.00543224 | 0.03996915 |
| *ENSGALG00000044794* | 3.54408474 | 0.17253058 | 0.00485167 | 0.03724176 |
| *NELL2* | 3.54210955 | 6.17822001 | 5.99E-12 | 3.69E-09 |
| *SYNC* | 3.54077591 | 2.16118123 | 0.00254575 | 0.02421968 |
| *BLVRA* | 3.52609249 | 2.62638445 | 0.00069524 | 0.01037512 |
| *RAPSN* | 3.52298784 | 2.44932362 | 0.00400913 | 0.03269002 |
| *ENSGALG00000008912* | 3.51191006 | 1.61882078 | 0.0023809 | 0.02332586 |
| *TFCP2L1* | 3.5026788 | 1.83523864 | 0.00142488 | 0.01682617 |
| *EPSTI1* | 3.49746611 | 1.18108551 | 0.0075566 | 0.04917349 |
| *ENSGALG00000035951* | 3.49653125 | 0.63512443 | 0.0064473 | 0.04466697 |
| *NET1* | 3.49350977 | 7.81720683 | 9.85E-09 | 1.83E-06 |
| *CAPN3* | 3.48932642 | 2.13136755 | 0.00011806 | 0.0030202 |

| | | | | |
|---|---|---|---|---|
| *ENSGALG00000036073* | 3.48523537 | 12.7118614 | 2.01E-06 | 0.00012951 |
| *CENPV* | 3.48187528 | 4.27373184 | 4.87E-10 | 1.50E-07 |
| *COL19A1* | 3.47934517 | 2.37307226 | 0.00285642 | 0.0261891 |
| *COL6A3* | 3.47640796 | 6.94712635 | 1.02E-06 | 7.43E-05 |
| *ENSGALG00000004113* | 3.47291468 | 3.64640877 | 0.00042143 | 0.00725463 |
| *TMOD1* | 3.47124896 | 3.14160022 | 0.00011839 | 0.00302317 |
| *ST6GAL1* | 3.46556115 | 1.53723504 | 0.00468494 | 0.03639638 |
| *NOX4* | 3.46544538 | 1.74168386 | 0.00273681 | 0.02543505 |
| *NECAB2* | 3.45914754 | 0.99218218 | 0.00152894 | 0.01764263 |
| *NR5A2* | 3.4567448 | 1.69534356 | 0.00513775 | 0.03857927 |
| *TNFRSF1B* | 3.44895345 | 2.91953256 | 0.00035306 | 0.00638721 |
| *ENSGALG00000013031* | 3.44712581 | 1.83265331 | 0.00027174 | 0.00533843 |
| *ENSGALG00000004436* | 3.44261835 | 2.21467526 | 0.00030232 | 0.00574079 |
| *TEX47* | 3.43965434 | -0.2857205 | 0.00635518 | 0.04426722 |
| *CPZ* | 3.43812223 | 6.47276373 | 3.83E-08 | 5.42E-06 |
| *COL5A2* | 3.43535024 | 9.97808021 | 1.34E-05 | 0.00059092 |
| *ENSGALG00000005776* | 3.43020601 | 0.56142813 | 0.00531683 | 0.03936009 |
| *MCF2L* | 3.42927285 | 3.35122062 | 5.42E-06 | 0.0002838 |
| *FGF18* | 3.42483137 | 2.08037993 | 0.0006303 | 0.00973447 |
| *SLITRK3* | 3.41752388 | 1.53349027 | 0.00121552 | 0.01515057 |
| *ENSGALG00000015685* | 3.41620475 | 0.21677264 | 0.00672029 | 0.04581779 |
| *RCSD1* | 3.41352378 | 4.13792004 | 1.06E-06 | 7.59E-05 |
| *PTPRO* | 3.40570399 | 2.20850643 | 0.00059261 | 0.00929443 |
| *ENSGALG00000043632* | 3.3979626 | 2.16278671 | 0.00263136 | 0.02471831 |
| *FAM107B* | 3.3975138 | 2.95367657 | 0.00033281 | 0.00610674 |
| *TAL1* | 3.39424837 | 1.51159154 | 0.00134236 | 0.01616196 |
| *ENSGALG00000011930* | 3.39226313 | 2.95350966 | 0.00740724 | 0.04855583 |
| *PDGFB* | 3.38979616 | 2.99751201 | 0.0001072 | 0.00281366 |
| *TMEM211* | 3.37351071 | 4.2700696 | 1.95E-06 | 0.00012639 |
| *ENSGALG00000034986* | 3.36804539 | 3.2813807 | 0.0007526 | 0.01092531 |
| *CALN1* | 3.35743484 | 1.36502247 | 0.00529474 | 0.03923768 |
| *PLXND1* | 3.35696868 | 3.44550162 | 0.00166862 | 0.01855551 |
| *ENSGALG00000006344* | 3.3518439 | 4.24492175 | 4.74E-07 | 4.07E-05 |
| *ENSGALG00000002113* | 3.34606785 | 1.87797598 | 0.00548192 | 0.0401564 |
| *SPRY2* | 3.33739143 | 4.84773226 | 1.43E-10 | 5.07E-08 |
| *ENSGALG00000035219* | 3.33729584 | 1.77075789 | 9.34E-05 | 0.0025714 |
| *DLK1* | 3.33697156 | 3.4777098 | 0.00085204 | 0.01185234 |
| *ENSGALG00000034310* | 3.32830282 | 0.24477282 | 0.00733808 | 0.04832955 |
| *KHDRBS3* | 3.32404631 | 5.56904863 | 3.79E-10 | 1.25E-07 |
| *ACKR4* | 3.31993179 | 1.18538417 | 0.00084985 | 0.0118452 |

| | | | | |
|---|---|---|---|---|
| *CNNM2* | 3.31919417 | 0.54203442 | 0.00731615 | 0.04825248 |
| *ENSGALG00000039209* | 3.31705857 | 4.59509975 | 0.0001109 | 0.00289462 |
| *PTGS2* | 3.31685776 | 3.66714565 | 0.00021353 | 0.0045221 |
| *PPFIBP2* | 3.31677348 | 5.20353322 | 4.52E-06 | 0.00024812 |
| *PLPP7* | 3.30839237 | 1.81581082 | 0.00011992 | 0.00305667 |
| *ENSGALG00000040032* | 3.30653997 | 0.172313 | 0.00250048 | 0.02390235 |
| *GJA4* | 3.28883794 | 3.06779322 | 0.00107222 | 0.01393104 |
| *ENSGALG00000023819* | 3.28702744 | 2.68502896 | 3.72E-05 | 0.00127238 |
| *ENSGALG00000023936* | 3.2861623 | 4.20843106 | 0.00035597 | 0.00642336 |
| *BRINP1* | 3.27078876 | 2.56778924 | 0.00508846 | 0.03843514 |
| *ENSGALG00000028238* | 3.26988294 | 0.98846424 | 0.00357935 | 0.03023507 |
| *P3H2* | 3.26277392 | 4.04041087 | 6.30E-06 | 0.00031835 |
| *ENSGALG00000010760* | 3.25227416 | 1.31501103 | 0.00441218 | 0.03492951 |
| *KDR* | 3.23918862 | 3.33225345 | 0.00274594 | 0.02545666 |
| *ENSGALG00000005078* | 3.23321383 | 2.8213543 | 0.00184228 | 0.01964029 |
| *ENSGALG00000008113* | 3.22705878 | 0.6953835 | 0.00150391 | 0.01743922 |
| *ENSGALG00000015080* | 3.22566319 | 1.61322258 | 0.00711875 | 0.04763902 |
| *ASB12* | 3.21189519 | 2.46732522 | 0.00291805 | 0.02646034 |
| *CXCR4* | 3.21156863 | 2.95158661 | 0.00674497 | 0.04591343 |
| *ENSGALG00000030583* | 3.1995187 | 5.05283181 | 4.19E-09 | 8.97E-07 |
| *CACNA1B* | 3.19527258 | 1.63369675 | 0.00669813 | 0.04575493 |
| *ENSGALG00000032779* | 3.19301919 | 6.77144495 | 7.80E-09 | 1.51E-06 |
| *ENSGALG00000021399* | 3.18934551 | 4.37110557 | 0.00062544 | 0.00969118 |
| *HSPB1* | 3.18165771 | 3.45508746 | 2.47E-08 | 3.84E-06 |
| *ENSGALG00000039821* | 3.17295565 | 2.96382755 | 9.00E-06 | 0.00043004 |
| *RGS6* | 3.17121923 | 1.38839867 | 0.00324779 | 0.02841463 |
| *ANO6* | 3.17057237 | 5.70844626 | 0.00015 | 0.00358461 |
| *PDGFA* | 3.16921522 | 5.60341883 | 2.82E-08 | 4.20E-06 |
| *ENSGALG00000012550* | 3.16490707 | 4.41414922 | 2.77E-05 | 0.00102313 |
| *STOM* | 3.16282252 | 4.19075911 | 0.00012604 | 0.00317835 |
| *MTERF2* | 3.15651636 | -0.3127997 | 0.00225294 | 0.02236471 |
| *RGS7BP* | 3.1543343 | 0.32833809 | 0.00690729 | 0.04662092 |
| *THSD7A* | 3.15064628 | 2.38251848 | 0.00175271 | 0.01904422 |
| *KLHL41* | 3.14808612 | 4.91471563 | 3.63E-05 | 0.00125924 |
| *SORCS2* | 3.14634786 | 3.07874129 | 0.00064084 | 0.00985341 |
| *CLEC3B* | 3.14475582 | 4.66776586 | 0.00019461 | 0.00425526 |
| *ENSGALG00000005472* | 3.1312472 | 0.55359535 | 0.00178479 | 0.01921568 |
| *ETNPPL* | 3.12701166 | 1.50877121 | 0.00032383 | 0.00601993 |
| *LAMB1* | 3.11799751 | 6.3947375 | 0.00036957 | 0.00657058 |
| *KCNAB1* | 3.10959296 | 3.90166582 | 2.76E-05 | 0.00102313 |

| | | | | |
|---|---|---|---|---|
| *EDNRA* | 3.10528146 | 5.68488785 | 0.00082139 | 0.01156236 |
| *ENSGALG00000040051* | 3.09372315 | 1.55620945 | 0.00179989 | 0.01931945 |
| *SHE* | 3.08308192 | 3.833879 | 1.75E-05 | 0.00073244 |
| *COL6A1* | 3.08127842 | 7.50921355 | 0.0001903 | 0.00418028 |
| *ENSGALG00000031934* | 3.07824176 | 6.56446353 | 5.51E-05 | 0.0016839 |
| *ENSGALG00000029072* | 3.07130228 | 1.82938468 | 0.00351077 | 0.02987711 |
| *CELSR1* | 3.05825307 | 0.75827988 | 0.00769373 | 0.04976827 |
| *FILIP1L* | 3.05244787 | 3.90431461 | 0.0009416 | 0.01279619 |
| *ENSGALG00000039727* | 3.04265036 | 0.00447368 | 0.00702968 | 0.0472596 |
| *COL20A1* | 3.03930422 | 3.07642537 | 0.00492188 | 0.03757683 |
| *FLT4* | 3.03778833 | 4.51754843 | 0.00013918 | 0.00338904 |
| *ENSGALG00000034868* | 3.03218092 | 5.0579938 | 0.00172316 | 0.01882701 |
| *SMOX* | 3.02807453 | 3.71653383 | 4.69E-08 | 6.39E-06 |
| *ENSGALG00000037332* | 3.02626752 | 4.33640554 | 3.89E-05 | 0.00131226 |
| *ENSGALG00000016285* | 3.01356132 | 1.85553563 | 0.00512191 | 0.03852185 |
| *ENSGALG00000036270* | 3.01153408 | 2.33844977 | 0.00378686 | 0.03155043 |
| *ENSGALG00000044333* | 3.00490054 | 0.4423336 | 0.00308525 | 0.02745093 |
| *ENSGALG00000011287* | 3.00434281 | 0.7517829 | 0.00412926 | 0.03336183 |
| *SOCS2* | 3.0033957 | 4.35975815 | 0.00380049 | 0.03162679 |
| *MET* | 3.0030279 | 1.86453822 | 0.00517404 | 0.03870814 |
| *ENSGALG00000009689* | 3.0002514 | 4.72513463 | 0.00011555 | 0.00297759 |
| *SGCG* | 2.98518528 | 2.36159766 | 0.00480065 | 0.03705117 |
| *DCN* | 2.98499971 | 10.6834512 | 3.98E-05 | 0.00133387 |
| *TPM1* | 2.96015519 | 8.73512256 | 2.81E-07 | 2.80E-05 |
| *MMP2* | 2.95882878 | 8.71545528 | 3.64E-05 | 0.00125924 |
| *FN1* | 2.95682806 | 8.15808433 | 1.58E-08 | 2.71E-06 |
| *PDLIM1* | 2.9538221 | 3.51321273 | 0.00022923 | 0.00478315 |
| *FGD4* | 2.94807102 | 2.54186702 | 5.62E-05 | 0.00170594 |
| *GRIA2* | 2.93615698 | 3.71341623 | 0.0022383 | 0.02223678 |
| *IMPG1* | 2.93400344 | 1.52768966 | 0.00549809 | 0.04017637 |
| *ENSGALG00000038343* | 2.93170089 | 3.85556531 | 1.06E-05 | 0.00048855 |
| *CRHR2* | 2.92451815 | 2.17375871 | 0.0070997 | 0.04755698 |
| *ENSGALG00000000498* | 2.90470136 | 1.82986752 | 0.00240673 | 0.02340069 |
| *HHIP* | 2.90439616 | 2.44554828 | 0.00312769 | 0.02770663 |
| *ENSGALG00000036780* | 2.89921809 | 4.75187425 | 0.00596948 | 0.04240492 |
| *DKK3* | 2.89371971 | 6.90637521 | 4.01E-07 | 3.64E-05 |
| *CD74* | 2.89347131 | 6.04818617 | 4.12E-06 | 0.00023142 |
| *ENSGALG00000012834* | 2.89270677 | 7.34996958 | 9.14E-05 | 0.00252658 |
| *GSC* | 2.89257245 | 4.47316723 | 0.00014177 | 0.00344023 |
| *CORO2B* | 2.8858028 | 4.02893931 | 1.56E-06 | 0.00010346 |

| | | | | |
|---|---|---|---|---|
| *MOXD1* | 2.88133618 | 4.11980981 | 0.00010333 | 0.00274395 |
| *ENSGALG00000006751* | 2.87938029 | 3.40901083 | 0.00013016 | 0.00324192 |
| *CFL2* | 2.87512919 | 7.09645198 | 4.33E-11 | 1.91E-08 |
| *RASSF2* | 2.87101835 | 4.90259893 | 2.38E-05 | 0.00091242 |
| *GNG10* | 2.86423999 | 5.06089959 | 5.96E-07 | 4.85E-05 |
| *TDRD12* | 2.86345815 | 2.23550194 | 0.00383636 | 0.03185033 |
| *ENSGALG00000031117* | 2.85354384 | 5.10600948 | 3.20E-08 | 4.63E-06 |
| *KCNJ5* | 2.84752554 | 2.6188583 | 0.00103536 | 0.01358859 |
| *ENSGALG00000035244* | 2.82560319 | 2.82511483 | 0.0015539 | 0.01786797 |
| *IMPG2* | 2.82481253 | 1.3570468 | 0.00361814 | 0.03048589 |
| *ENSGALG00000023818* | 2.81918749 | 3.21383497 | 0.00032851 | 0.00605924 |
| *ENSGALG00000009594* | 2.81328764 | 4.14638117 | 0.00173114 | 0.0188824 |
| *STRA6* | 2.8099999 | 0.99922673 | 0.0068443 | 0.04631074 |
| *MAFA* | 2.8055638 | 1.5489969 | 0.00505826 | 0.03824651 |
| *NANOS1* | 2.79996343 | 1.91961592 | 0.00036801 | 0.0065652 |
| *PTHLH* | 2.79836682 | 3.59146798 | 0.005253 | 0.03907159 |
| *TPM2* | 2.77531519 | 8.01207581 | 5.48E-06 | 0.00028622 |
| *GPM6B* | 2.77265542 | 5.03709059 | 7.16E-07 | 5.50E-05 |
| *ENSGALG00000006453* | 2.75842566 | 7.9403168 | 7.36E-05 | 0.00213307 |
| *RASGRP3* | 2.75730473 | 4.22556747 | 0.00028328 | 0.00551244 |
| *TXNL1* | 2.75491554 | 5.79782798 | 8.14E-07 | 6.10E-05 |
| *ADAMTS17* | 2.73998175 | 3.63960869 | 0.0026709 | 0.02500674 |
| *MYO16* | 2.73951243 | 3.55474659 | 0.0065464 | 0.04508105 |
| *F2RL2* | 2.73845109 | 3.77051013 | 0.0002343 | 0.00486021 |
| *VILL* | 2.73733639 | 2.6830288 | 0.00405083 | 0.03301101 |
| *AKAP12* | 2.73724427 | 7.18093045 | 3.33E-05 | 0.00117942 |
| *PDGFC* | 2.73101655 | 4.95263069 | 3.96E-05 | 0.00132984 |
| *ENSGALG00000044797* | 2.71859282 | 2.85513604 | 8.29E-05 | 0.00233211 |
| *ENSGALG00000011812* | 2.71722903 | 4.14599791 | 0.00016526 | 0.00382333 |
| *BVES* | 2.70601927 | 4.15999116 | 2.37E-05 | 0.00091189 |
| *NETO2* | 2.70417404 | 2.67111816 | 0.00025283 | 0.00508071 |
| *CDC26* | 2.69817787 | 2.90725666 | 0.00061095 | 0.00951882 |
| *POPDC3* | 2.69676912 | 2.8685637 | 0.00015923 | 0.0037356 |
| *ADAMTSL3* | 2.69036385 | 3.05219579 | 0.00177633 | 0.0191538 |
| *CMIP* | 2.68503693 | 4.46065833 | 1.93E-08 | 3.18E-06 |
| *ENSGALG00000032422* | 2.68335908 | 5.0302821 | 0.00047904 | 0.0079635 |
| *ENSGALG00000014128* | 2.68248979 | 2.24654341 | 0.00317619 | 0.02796114 |
| *ENSGALG00000032905* | 2.68154092 | 1.73510446 | 0.00242144 | 0.02349526 |
| *ABLIM2* | 2.67515959 | 3.8171913 | 0.00666277 | 0.04557939 |
| *ENSGALG00000031853* | 2.674953 | 2.51460222 | 0.00410707 | 0.03328276 |

| | | | | |
|---|---|---|---|---|
| *LYN* | 2.67304564 | 3.60857933 | 2.03E-05 | 0.00081553 |
| *ENSGALG00000011962* | 2.66499153 | 5.28547011 | 0.00109951 | 0.01421831 |
| *PRRX2* | 2.66355982 | 5.40061677 | 4.72E-05 | 0.0015045 |
| *MEOX2* | 2.66113645 | 5.73751233 | 0.00291268 | 0.02646034 |
| *PAMR1* | 2.65320416 | 3.78178012 | 0.00568259 | 0.0411421 |
| *LRRC17* | 2.6484631 | 7.54200672 | 0.00128562 | 0.01576289 |
| *RGS2* | 2.64427168 | 5.47237111 | 1.52E-07 | 1.71E-05 |
| *PCGF5* | 2.62796395 | 1.14806039 | 0.00703198 | 0.0472596 |
| *TMX1* | 2.61513274 | 0.72456823 | 0.00562165 | 0.04084721 |
| *CRABP1* | 2.61373949 | 6.25502253 | 0.00754892 | 0.04914615 |
| *EFEMP1* | 2.61185224 | 3.95876816 | 0.00286083 | 0.02620201 |
| *CD82* | 2.60718843 | 4.81870362 | 0.00073074 | 0.01076854 |
| *ENSGALG00000040023* | 2.60415119 | 3.32180239 | 0.00082794 | 0.01161993 |
| *COTL1* | 2.59374099 | 7.93751703 | 3.31E-05 | 0.0011777 |
| *ENSGALG00000034722* | 2.58995933 | 2.66919777 | 0.00329391 | 0.02867628 |
| *LPAR3* | 2.58483118 | 5.44262317 | 8.94E-05 | 0.00247921 |
| *CDKN1A* | 2.58074327 | 2.56576936 | 0.00130622 | 0.01590279 |
| *ENSGALG00000045584* | 2.56729657 | 5.28775678 | 1.05E-05 | 0.00048598 |
| *RASSF3* | 2.56701318 | 5.31549484 | 1.58E-05 | 0.00067908 |
| *ID1* | 2.5563666 | 5.4230156 | 2.23E-05 | 0.00086327 |
| *PRR5* | 2.54974304 | 4.46675328 | 9.56E-06 | 0.00045084 |
| *ITM2C* | 2.5479168 | 4.36806314 | 1.06E-05 | 0.0004878 |
| *PHYHIPL* | 2.54766538 | 2.42678991 | 0.00581231 | 0.04169711 |
| *VEGFC* | 2.54116936 | 3.24320715 | 5.05E-06 | 0.00026838 |
| *PCDH18* | 2.53097519 | 5.30354063 | 2.84E-05 | 0.00103693 |
| *ANGPTL2* | 2.52121851 | 5.85139643 | 0.0001654 | 0.00382333 |
| *RPL22L1* | 2.51561589 | 8.59665564 | 7.08E-08 | 8.95E-06 |
| *MRPL43* | 2.5152683 | 3.61753804 | 0.00020546 | 0.00443762 |
| *ENSGALG00000008444* | 2.51371522 | 6.3386581 | 0.00605039 | 0.0427687 |
| *ENSGALG00000043889* | 2.50643355 | 2.27011088 | 0.00260282 | 0.02460591 |
| *ENSGALG00000028573* | 2.50196999 | 2.76765162 | 0.00014696 | 0.0035297 |
| *MYLK* | 2.50108089 | 6.73232563 | 7.97E-07 | 6.00E-05 |
| *STEAP3* | 2.49714123 | 0.96450381 | 0.00645855 | 0.04472301 |
| *IGSF21* | 2.49631845 | 1.67351249 | 0.00225838 | 0.02237794 |
| *ANGPT2* | 2.48696499 | 5.04019089 | 0.00016607 | 0.00382823 |
| *NR2F2* | 2.48395268 | 6.03026992 | 0.00495025 | 0.03771203 |
| *CMTM8* | 2.47963559 | 3.10300983 | 8.01E-05 | 0.00228458 |
| *CRISPLD2* | 2.47882958 | 6.00645047 | 1.82E-05 | 0.00074864 |
| *BBIP1* | 2.47372492 | 3.20804479 | 0.00019919 | 0.00433518 |
| *MSRB1* | 2.46954213 | 4.06771313 | 0.00073288 | 0.01077753 |

| | | | | |
|---|---|---|---|---|
| *CITED4* | 2.46868245 | 4.33094155 | 0.00039853 | 0.00694335 |
| *ENSGALG00000016391* | 2.46684957 | 2.81239668 | 0.00716701 | 0.04784329 |
| *NDUFS4* | 2.46179654 | 6.28397634 | 4.72E-07 | 4.07E-05 |
| *ENSGALG00000008677* | 2.46175622 | 10.9648287 | 3.98E-07 | 3.64E-05 |
| *ENSGALG00000028466* | 2.45556382 | 2.80582725 | 0.00220608 | 0.02208736 |
| *FAM20C* | 2.45198583 | 5.09804543 | 0.00031113 | 0.00584541 |
| *ENSGALG00000035996* | 2.44979028 | 6.83659376 | 2.25E-06 | 0.0001399 |
| *ENSGALG00000007007* | 2.44681245 | 3.48126182 | 0.00055766 | 0.00883442 |
| *ENSGALG00000035677* | 2.44416833 | 9.74769841 | 1.99E-07 | 2.13E-05 |
| *JAGN1* | 2.43106714 | 3.72153193 | 0.00027036 | 0.00533445 |
| *EGFR* | 2.41012367 | 5.25313495 | 2.84E-05 | 0.00103693 |
| *PDE5A* | 2.40564253 | 3.42697531 | 1.86E-05 | 0.00076324 |
| *ENSGALG00000044664* | 2.40002031 | 4.09196415 | 0.00016117 | 0.0037688 |
| *CRMP1* | 2.39984664 | 5.39520957 | 6.32E-09 | 1.25E-06 |
| *ENSGALG00000043377* | 2.3794296 | 6.51131071 | 0.00099999 | 0.01334515 |
| *NBL1* | 2.37165014 | 5.85900269 | 0.00060247 | 0.00940746 |
| *TSPAN9* | 2.37152542 | 2.27951804 | 0.0046349 | 0.03614657 |
| *MITF* | 2.37017232 | 4.84144368 | 0.00026229 | 0.00521253 |
| *FADD* | 2.36435134 | 1.8329588 | 0.00623389 | 0.04369338 |
| *ENSGALG00000015704* | 2.36380725 | 8.74485717 | 0.00014796 | 0.00354187 |
| *ENSGALG00000003670* | 2.35884522 | 4.63925007 | 0.00101603 | 0.01347123 |
| *PHACTR1* | 2.35047534 | 2.65522373 | 0.0037082 | 0.0310414 |
| *ARSB* | 2.34957478 | 4.03573368 | 0.00126408 | 0.01560469 |
| *ENSGALG00000038226* | 2.34870492 | 5.81710232 | 2.55E-05 | 0.00095761 |
| *TM6SF1* | 2.34843074 | 3.51473175 | 0.00139331 | 0.01660591 |
| *ENSGALG00000004769* | 2.34253892 | 1.50572438 | 0.00268271 | 0.02508126 |
| *CYP4V3* | 2.33881303 | 3.00602655 | 0.00509366 | 0.03845203 |
| *ENSGALG00000028551* | 2.33368743 | 4.33613352 | 0.00016876 | 0.00386315 |
| *ENSGALG00000033522* | 2.33230839 | 3.35046218 | 5.12E-05 | 0.0015954 |
| *COX7A2* | 2.31817283 | 6.95062552 | 1.42E-06 | 9.78E-05 |
| *SLC9A3R1* | 2.3169871 | 3.9183265 | 0.00010227 | 0.00273501 |
| *GPR27* | 2.31022517 | 5.21635395 | 5.85E-06 | 0.00029859 |
| *ENSGALG00000019489* | 2.3077374 | 3.54372409 | 0.0003457 | 0.00628618 |
| *CSF1R* | 2.3024677 | 6.09419863 | 0.00035727 | 0.00643863 |
| *MARCKS* | 2.29051495 | 8.52605515 | 7.66E-05 | 0.00221548 |
| *XRCC2* | 2.29036579 | 3.78148329 | 1.77E-05 | 0.0007336 |
| *ENSGALG00000032369* | 2.2903523 | 5.16149435 | 8.34E-07 | 6.18E-05 |
| *ENSGALG00000041277* | 2.27628324 | 8.45881661 | 7.96E-05 | 0.00227983 |
| *ENSGALG00000013512* | 2.27592056 | 7.28092935 | 1.26E-07 | 1.50E-05 |
| *COX7C* | 2.27504685 | 7.42262156 | 0.00026034 | 0.00519474 |

| | | | | |
|---|---|---|---|---|
| *WBP1L* | 2.27349472 | 6.2747283 | 5.50E-05 | 0.0016839 |
| *MMP13* | 2.26154103 | 10.1097618 | 0.00515029 | 0.03861213 |
| *BMF* | 2.25840034 | 5.26634656 | 0.00047092 | 0.00787483 |
| *DTX2* | 2.24980463 | 2.87853844 | 0.0003602 | 0.00646539 |
| *ENSGALG00000045788* | 2.24857245 | 3.82103818 | 0.00190231 | 0.02006169 |
| *ENSGALG00000003966* | 2.24516396 | 9.45399371 | 2.43E-07 | 2.49E-05 |
| *SH3GL3* | 2.24287827 | 2.6361542 | 0.00549618 | 0.04017637 |
| *NQO1* | 2.23862959 | 2.6248012 | 0.00028954 | 0.00559846 |
| *ENSGALG00000028774* | 2.2368281 | 7.08108953 | 5.50E-05 | 0.0016839 |
| *ENSGALG00000032220* | 2.23473073 | 3.38727933 | 0.00266589 | 0.02497636 |
| *RPS28* | 2.23329638 | 8.59715932 | 0.00072964 | 0.01076854 |
| *EYA4* | 2.22753911 | 4.76315574 | 0.00066461 | 0.01006658 |
| *PLA2G4A* | 2.22570163 | 3.065495 | 0.0008199 | 0.01156236 |
| *ENSGALG00000014708* | 2.21321484 | 5.07910329 | 3.63E-07 | 3.38E-05 |
| *RAB31* | 2.21279017 | 3.10260449 | 0.00099437 | 0.01330875 |
| *PIK3AP1* | 2.21277406 | 2.51440104 | 0.00168441 | 0.01857314 |
| *CHST15* | 2.20660663 | 5.90188775 | 0.00747361 | 0.04881311 |
| *ENSGALG00000038229* | 2.20505446 | 2.96861047 | 0.00295242 | 0.0266887 |
| *ENSGALG00000027891* | 2.2030457 | 9.87990863 | 9.01E-05 | 0.00249551 |
| *ARL15* | 2.20222283 | 1.07768343 | 0.00722233 | 0.04799169 |
| *ENSGALG00000030587* | 2.20205102 | 4.18783618 | 0.00237733 | 0.02330707 |
| *TBC1D7* | 2.19750241 | 4.99181061 | 1.86E-07 | 2.00E-05 |
| *ENSGALG00000004588* | 2.19546274 | 9.87767559 | 8.43E-06 | 0.00040682 |
| *ITGB8* | 2.19170832 | 2.38667326 | 0.00593802 | 0.04228941 |
| *SEC61G* | 2.1886081 | 7.26912901 | 1.27E-05 | 0.00056702 |
| *ARID5B* | 2.17785409 | 5.14493463 | 7.28E-07 | 5.54E-05 |
| *ELMO1* | 2.17602517 | 5.68582415 | 8.55E-05 | 0.00239972 |
| *RTN1* | 2.17548195 | 4.13658854 | 0.00073649 | 0.01079708 |
| *ENSGALG00000035809* | 2.17475467 | 3.76979297 | 0.00020981 | 0.00448371 |
| *UQCC2* | 2.16446563 | 5.14800246 | 1.31E-06 | 9.14E-05 |
| *TOLLIP* | 2.15739611 | 3.54642104 | 0.00268418 | 0.02508126 |
| *CALML4* | 2.15734664 | 4.15631747 | 0.00020774 | 0.00445958 |
| *ENSGALG00000032183* | 2.15612183 | 4.07631227 | 7.70E-05 | 0.00222313 |
| *ENSGALG00000039455* | 2.14985401 | 1.82618731 | 0.00495689 | 0.03771939 |
| *MYH15* | 2.14589196 | 5.07159676 | 0.00075621 | 0.01093071 |
| *ENSGALG00000028451* | 2.14527639 | 4.05632533 | 0.00291356 | 0.02646034 |
| *MGST3* | 2.14292284 | 5.88214754 | 2.04E-06 | 0.00012995 |
| *ENSGALG00000043142* | 2.13638428 | 2.56222716 | 0.00314907 | 0.02784365 |
| *RAD51B* | 2.12127002 | 3.33266155 | 0.00190516 | 0.02006868 |
| *VAT1* | 2.11034818 | 2.94869567 | 0.00039805 | 0.00694335 |

| | | | | |
|---|---|---|---|---|
| *SH3BP2* | 2.10910724 | 3.16416678 | 0.00058273 | 0.00917001 |
| *SERINC5* | 2.10785469 | 4.30561253 | 0.0054388 | 0.03996915 |
| *ENSGALG00000000474* | 2.10278357 | 8.96392147 | 5.73E-05 | 0.0017264 |
| *ELAVL4* | 2.10231978 | 3.68447225 | 0.00147405 | 0.01723415 |
| *ENSGALG00000035836* | 2.10035539 | 8.38673992 | 3.65E-07 | 3.38E-05 |
| *UCHL1* | 2.09975316 | 6.98456025 | 1.55E-06 | 0.00010346 |
| *RPS25* | 2.09003306 | 9.6268475 | 1.76E-05 | 0.00073312 |
| *DESI1* | 2.08968471 | 2.3149037 | 0.00347159 | 0.02960374 |
| *PRSS12* | 2.08796899 | 2.58425865 | 0.00351409 | 0.02987711 |
| *ENSGALG00000027571* | 2.08649754 | 5.27034037 | 2.73E-06 | 0.00016408 |
| *ATP5J* | 2.08424701 | 7.60483915 | 1.06E-06 | 7.59E-05 |
| *CYYR1* | 2.08062705 | 1.97022694 | 0.00617172 | 0.04337997 |
| *ATP5G1* | 2.07992328 | 7.49512091 | 2.54E-05 | 0.00095761 |
| *PHLDA2* | 2.07634296 | 6.13719655 | 0.00578193 | 0.04156351 |
| *TRP53I11* | 2.07150854 | 5.94894944 | 0.00171632 | 0.01880577 |
| *SIM2* | 2.070149 | 3.52268732 | 0.00608234 | 0.04295649 |
| *ENSGALG00000008425* | 2.06413616 | 6.58390829 | 9.44E-05 | 0.00258633 |
| *FAM53A* | 2.05587239 | 5.10589544 | 1.46E-05 | 0.00063377 |
| *ENSGALG00000037880* | 2.04674208 | 8.09977706 | 0.00014724 | 0.00353042 |
| *ENSGALG00000041478* | 2.04397887 | 6.60801594 | 0.00018316 | 0.00408119 |
| *ENSGALG00000032933* | 2.04023229 | 4.4510238 | 0.00014664 | 0.00352802 |
| *CISD1* | 2.03675231 | 5.50494561 | 4.85E-05 | 0.00153362 |
| *ENSGALG00000016635* | 2.03055677 | 5.17426104 | 0.00279317 | 0.02571156 |
| *ENSGALG00000013624* | 2.02285586 | 3.89681719 | 0.00763549 | 0.04957629 |
| *DUSP10* | 2.02235857 | 5.22950039 | 0.00018273 | 0.00408119 |
| *SLC25A21* | 2.01875892 | 3.49223107 | 5.24E-06 | 0.00027671 |
| *LBH* | 2.0174094 | 5.58718854 | 2.16E-06 | 0.00013565 |
| *LAMA2* | 2.01584836 | 5.06377423 | 0.00595847 | 0.04236477 |
| *ENSGALG00000044832* | 2.01553008 | 4.21035571 | 0.0029509 | 0.0266887 |
| *ENSGALG00000006771* | 2.01497556 | 10.9668943 | 2.66E-05 | 0.00099069 |
| *NT5C1B* | 2.0126223 | 3.9210165 | 0.00010503 | 0.00276691 |
| *3110043O21RIK* | 2.00520656 | 3.6695171 | 0.00016726 | 0.00384752 |
| *INIP* | 2.0021303 | 5.48768062 | 9.31E-05 | 0.00256878 |
| *ENSGALG00000020999* | 2.00172946 | 3.70084956 | 0.00029175 | 0.0056079 |

**Supplementary table S9.** Upregulated genes in limb IMM compared to head IMM

| Gene name | logFC | logCPM | PValue | p.adj |
|---|---|---|---|---|
| *MMP9* | 12.2564044 | 6.38824165 | 2.85E-12 | 3.36E-09 |
| *ENSGALG00000017815* | 9.865704 | 3.32981472 | 1.54E-14 | 3.11E-11 |
| *ENSGALG00000030886* | 9.54441992 | 1.94210894 | 4.39E-06 | 0.00039772 |
| *ENSGALG00000043064* | 9.43314373 | 5.9690901 | 2.32E-12 | 3.29E-09 |
| *FAM81A* | 9.16715037 | 1.86569761 | 7.00E-08 | 1.71E-05 |
| *EMX2* | 9.14604349 | 1.61859096 | 9.66E-06 | 0.00066692 |
| *ENSGALG00000034511* | 9.06449296 | 1.84532495 | 3.32E-05 | 0.00167709 |
| *HOXA7* | 8.98932789 | 5.79185713 | 1.23E-19 | 8.67E-16 |
| *LRP2BP* | 8.85099126 | 2.23123932 | 5.76E-10 | 4.79E-07 |
| *HOXA5* | 8.82043138 | 7.64637016 | 2.96E-27 | 4.19E-23 |
| *IRS2* | 8.74896187 | 1.64662673 | 6.05E-08 | 1.56E-05 |
| *ENSGALG00000018264* | 8.55155265 | 2.19462759 | 1.04E-05 | 0.00071055 |
| *AGBL3* | 8.50649517 | 1.07224195 | 0.00026665 | 0.00647038 |
| *HOXA6* | 8.42993695 | 2.37313726 | 2.76E-09 | 1.35E-06 |
| *ENSGALG00000042795* | 8.39678226 | 2.85573869 | 1.10E-09 | 7.77E-07 |
| *ENSGALG00000037914* | 8.22256902 | 1.06965589 | 3.74E-06 | 0.00034807 |
| *ENSGALG00000008040* | 8.16964024 | 0.70881917 | 7.11E-06 | 0.00053283 |
| *ENSGALG00000038683* | 8.0702793 | 1.08960075 | 0.00021693 | 0.00572556 |
| *PEF1* | 8.04634663 | 2.95160406 | 1.95E-07 | 3.67E-05 |
| *ENSGALG00000030139* | 8.01240607 | 2.34740955 | 9.78E-08 | 2.27E-05 |
| *ENSGALG00000003213* | 7.95232851 | 1.90243428 | 1.52E-06 | 0.00017574 |
| *HOXA10* | 7.94172409 | 1.8977002 | 9.64E-07 | 0.00012403 |
| *ENSGALG00000044766* | 7.90603483 | 0.36120264 | 0.00567593 | 0.0441741 |
| *ENSGALG00000008032* | 7.86887752 | 1.9560273 | 3.03E-08 | 8.57E-06 |
| *CHST12* | 7.85793843 | 2.24799341 | 7.35E-06 | 0.00054145 |
| *ENSGALG00000032411* | 7.84563403 | 1.82964027 | 0.00037811 | 0.00825478 |
| *SUSD4* | 7.80467654 | 1.99787832 | 7.45E-05 | 0.0028509 |
| *TMC6* | 7.74903571 | 1.70960778 | 6.07E-05 | 0.00248332 |
| *ENSGALG00000006453* | 7.71776561 | 7.9403168 | 1.31E-17 | 6.18E-14 |
| *CEBPD* | 7.63104107 | 0.33275841 | 0.00025461 | 0.00634013 |
| *ENSGALG00000031090* | 7.44616045 | 1.07194207 | 0.0025041 | 0.02667584 |
| *GPRC5C* | 7.4432058 | 0.92458197 | 0.00028519 | 0.00673194 |
| *NRBP2* | 7.40723092 | 1.03589647 | 0.00048182 | 0.00973764 |
| *ENSGALG00000013723* | 7.39881741 | 0.6194026 | 6.17E-05 | 0.0025095 |
| *SRPK3* | 7.35397556 | 1.11763724 | 1.72E-05 | 0.00105385 |
| *SERPINF2* | 7.31074874 | 1.66698574 | 0.00010285 | 0.00351456 |
| *ENSGALG00000036725* | 7.28578883 | 1.5831241 | 0.0001219 | 0.00395524 |
| *ENSGALG00000019063* | 7.26905807 | 4.78622902 | 1.62E-09 | 9.99E-07 |

| | | | | |
|---|---|---|---|---|
| *HHIPL1* | 7.26892233 | 0.50000873 | 0.00015981 | 0.00473982 |
| *ENSGALG00000030871* | 7.26110067 | 1.21351102 | 0.00023079 | 0.00591477 |
| *ENSGALG00000038228* | 7.25477288 | 0.02167264 | 0.00027149 | 0.0065509 |
| *HERPUD1* | 7.24928124 | 1.84668572 | 6.62E-06 | 0.00051485 |
| *ENSGALG00000039794* | 7.20875406 | 0.39961859 | 1.08E-05 | 0.00072903 |
| *ENSGALG00000036822* | 7.18260132 | 0.52441143 | 0.00064988 | 0.0116526 |
| *KCNE2* | 7.14423858 | -0.0096454 | 0.00049169 | 0.00988049 |
| *ENSGALG00000030982* | 7.13791482 | 0.2065499 | 0.00093855 | 0.01455886 |
| *BMP7* | 7.12321364 | 1.49157514 | 0.00060408 | 0.01115837 |
| *ENSGALG00000039304* | 7.1008835 | 0.55417151 | 0.00040286 | 0.00864827 |
| *ENSGALG00000001862* | 7.09516737 | 0.41803708 | 0.00108677 | 0.01583368 |
| *ELMOD3* | 7.01239446 | 0.85288414 | 0.0001635 | 0.0048188 |
| *VSX1* | 7.01118938 | 0.27915875 | 7.66E-05 | 0.00292004 |
| *ENSGALG00000044574* | 7.00752435 | 1.45440464 | 0.0005837 | 0.01098091 |
| *CCDC134* | 6.97894709 | 1.93149413 | 0.00028047 | 0.00664633 |
| *PLXNA1* | 6.97785473 | 1.67284159 | 0.00052337 | 0.0102126 |
| *ENSGALG00000025541* | 6.93873927 | 0.80172374 | 2.38E-05 | 0.00132645 |
| *ENSGALG00000046402* | 6.92903087 | 0.39569602 | 0.00092299 | 0.01442827 |
| *PRIMA1* | 6.9024183 | -0.1468125 | 0.00070873 | 0.01218529 |
| *ENSGALG00000041380* | 6.8991841 | 1.35225416 | 0.00086021 | 0.01379745 |
| *BTG2* | 6.86406388 | -0.3343634 | 0.00598485 | 0.0458598 |
| *EPHB3* | 6.8214485 | 1.41953364 | 0.00113278 | 0.01630254 |
| *CYB561* | 6.81338377 | 0.68700496 | 0.00059117 | 0.01107717 |
| *ENSGALG00000037948* | 6.80125983 | 0.76485518 | 0.00089793 | 0.01419091 |
| *ENSGALG00000038873* | 6.79731734 | 0.16779487 | 0.00061617 | 0.01126217 |
| *ENSGALG00000035420* | 6.76156765 | 0.19677039 | 0.00172516 | 0.02080631 |
| *GM23202* | 6.74814789 | 0.67082862 | 0.00073282 | 0.01247079 |
| *ENSGALG00000045557* | 6.69203681 | -0.2510375 | 0.00521198 | 0.04206157 |
| *KLF4* | 6.68646469 | 0.10048858 | 0.00096171 | 0.01483679 |
| *ENSGALG00000025662* | 6.67731347 | 1.04269616 | 0.00164761 | 0.02016082 |
| *FZD8* | 6.62918302 | 2.71361803 | 0.00047382 | 0.00964474 |
| *4931428F04RIK* | 6.60339158 | 0.28559322 | 0.00196722 | 0.02264464 |
| *ENSGALG00000035588* | 6.55686812 | 1.16452355 | 0.00131394 | 0.01768443 |
| *ENSGALG00000037291* | 6.53102358 | -0.4101865 | 0.00036784 | 0.00808462 |
| *SCN4A* | 6.51260652 | -0.0875204 | 0.00431266 | 0.03736144 |
| *ENSGALG00000037160* | 6.48716322 | 0.78639517 | 0.00084133 | 0.01360854 |
| *ENSGALG00000038681* | 6.46476802 | 0.11944816 | 0.00055576 | 0.01065364 |
| *FKBP10* | 6.44679501 | 2.17414732 | 0.00125727 | 0.01729111 |
| *ENSGALG00000031683* | 6.43957998 | 0.49266355 | 0.00144694 | 0.01847458 |
| *SOCS1* | 6.43364026 | 1.47397761 | 0.00056379 | 0.01070587 |

| | | | | |
|---|---|---|---|---|
| *HOXD9* | 6.42939416 | 0.73290229 | 0.00057167 | 0.01081207 |
| *ENSGALG00000008107* | 6.42640517 | -0.3473541 | 0.00059075 | 0.01107717 |
| *KLHDC10* | 6.42064991 | 0.49875299 | 0.00127972 | 0.01750887 |
| *KLF5* | 6.41944197 | -0.038502 | 0.0011311 | 0.01629495 |
| *ZBTB37* | 6.39275176 | -0.4235496 | 0.00312864 | 0.0305669 |
| *ENSGALG00000030480* | 6.38805359 | -0.214782 | 0.0037304 | 0.03426292 |
| *MLXIPL* | 6.36064248 | -0.3442358 | 0.00095396 | 0.01474942 |
| *ENSGALG00000043938* | 6.35855229 | 1.56181655 | 0.00052072 | 0.01017539 |
| *ENSGALG00000027949* | 6.35074207 | 0.62820498 | 0.00138947 | 0.01805831 |
| *ENSGALG00000014589* | 6.34732521 | 2.06094892 | 0.00109451 | 0.01589741 |
| *ENSGALG00000035047* | 6.33879679 | 0.06954319 | 0.00075465 | 0.01272464 |
| *ENSGALG00000046284* | 6.33561408 | -0.6358601 | 0.00211254 | 0.02378415 |
| *NEU4* | 6.32919678 | 0.43340462 | 0.00291655 | 0.02938276 |
| *FAM168A* | 6.32581363 | 0.61832045 | 0.00019518 | 0.00539288 |
| *ENSGALG00000028983* | 6.32468729 | 7.88868494 | 8.37E-06 | 0.00059492 |
| *ENSGALG00000003660* | 6.30642192 | 1.37872691 | 0.00219613 | 0.02442507 |
| *ABCA1* | 6.3057643 | 0.49375382 | 0.00367669 | 0.03401841 |
| *ENSGALG00000044181* | 6.28832848 | 0.72609529 | 0.0012852 | 0.01753301 |
| *ENSGALG00000017810* | 6.26152656 | 0.82311041 | 0.0008559 | 0.0137752 |
| *RHBDF1* | 6.19109921 | 4.27956871 | 1.72E-15 | 6.09E-12 |
| *ENSGALG00000040232* | 6.18854064 | 0.92256581 | 0.00136125 | 0.01786165 |
| *ENSGALG00000007304* | 6.11524911 | 0.47899679 | 0.00178726 | 0.02126519 |
| *ENSGALG00000003025* | 6.10256115 | 0.47644721 | 0.00202339 | 0.02308464 |
| *ENSGALG00000001529* | 6.09993245 | 0.55324747 | 0.0008861 | 0.01407647 |
| *ENSGALG00000030429* | 6.09894669 | 0.24786935 | 0.00435859 | 0.03769007 |
| *TBC1D22B* | 6.09525155 | 0.45889421 | 0.00117116 | 0.01665169 |
| *ANKRD13D* | 6.07039694 | 0.78574141 | 0.00072855 | 0.01243286 |
| *ENSGALG00000006160* | 6.03992266 | 0.51812835 | 0.00285888 | 0.02901359 |
| *ENSGALG00000041709* | 6.03956348 | -0.5797353 | 0.00601372 | 0.04596224 |
| *MMP13* | 6.02059019 | 10.1097618 | 1.62E-09 | 9.99E-07 |
| *ADAM33* | 6.0139905 | 1.93487763 | 0.00080101 | 0.01317663 |
| *GDPD1* | 5.9807769 | 0.1029569 | 0.00101475 | 0.01521107 |
| *ENSGALG00000029104* | 5.97596314 | 0.50247432 | 0.00163096 | 0.02002884 |
| *ENSGALG00000032739* | 5.96184671 | 0.00087786 | 0.0029225 | 0.02940583 |
| *LRFN5* | 5.96018275 | 0.19786716 | 0.00525581 | 0.04224657 |
| *ENSGALG00000033819* | 5.9574813 | 0.31038523 | 0.00454104 | 0.03872336 |
| *ENSGALG00000010315* | 5.95035481 | 0.39881022 | 0.00429601 | 0.03726283 |
| *WNT9A* | 5.94776676 | 2.1477072 | 0.00026993 | 0.00653886 |
| *ENSGALG00000042969* | 5.94243694 | 0.01753448 | 0.00162987 | 0.02002884 |
| *SLC20A1* | 5.92908576 | 0.31204858 | 0.00220416 | 0.02447585 |

| | | | | |
|---|---|---|---|---|
| *ENSGALG00000032961* | 5.92840946 | -0.1965373 | 0.00232202 | 0.02534696 |
| *NCEH1* | 5.91674281 | 0.05899261 | 0.00193882 | 0.02250877 |
| *ENSGALG00000010926* | 5.91107089 | 11.9738272 | 3.50E-09 | 1.55E-06 |
| *ENSGALG00000005739* | 5.91069745 | 0.12239127 | 0.00073242 | 0.01247079 |
| *ENSGALG00000042462* | 5.90246237 | 0.09270031 | 0.00507873 | 0.04131248 |
| *GRM4* | 5.90126445 | 1.99305547 | 0.00618411 | 0.04696003 |
| *ENSGALG00000033068* | 5.90088742 | -0.0374778 | 0.00165026 | 0.02016082 |
| *AGPAT2* | 5.88366328 | 0.44825524 | 0.0020444 | 0.02324924 |
| *TNPO2* | 5.87454058 | 0.45476015 | 0.00307156 | 0.03023896 |
| *ENSGALG00000032126* | 5.86876907 | 0.38323189 | 0.00184083 | 0.02171271 |
| *ENSGALG00000044014* | 5.84462307 | 0.06952096 | 0.00318369 | 0.03088927 |
| *ENSGALG00000046482* | 5.82092135 | -0.8879166 | 0.00602765 | 0.04604386 |
| *0610009O20RIK* | 5.80881932 | 0.4434747 | 0.00376504 | 0.0344468 |
| *ENSGALG00000035438* | 5.80043668 | 3.2937547 | 3.93E-06 | 0.00036331 |
| *ENSGALG00000045990* | 5.79894679 | 0.01968986 | 0.0062065 | 0.04707955 |
| *1700037H04RIK* | 5.77354354 | 0.00418596 | 0.00369398 | 0.03413372 |
| *ENSGALG00000015197* | 5.76267819 | 0.05694002 | 0.00129956 | 0.01764401 |
| *ENSGALG00000026038* | 5.75505432 | 1.97601946 | 2.17E-05 | 0.00125481 |
| *HPSE* | 5.74515103 | -0.2223134 | 0.00252359 | 0.02680271 |
| *TBX5* | 5.7317053 | 5.75443885 | 1.06E-07 | 2.35E-05 |
| *ENSGALG00000045032* | 5.72353879 | 1.82240068 | 3.88E-05 | 0.00185762 |
| *TEX47* | 5.71785561 | -0.2857205 | 0.00536481 | 0.04281589 |
| *ENSGALG00000038725* | 5.70364001 | 0.82478052 | 0.00090714 | 0.01424523 |
| *DIO1* | 5.7020822 | -0.2121059 | 0.00370361 | 0.0341569 |
| *ENSGALG00000025551* | 5.69210801 | -0.2190417 | 0.00662134 | 0.04901733 |
| *ENSGALG00000045982* | 5.68677359 | -0.4776372 | 0.00400317 | 0.03581144 |
| *ENSGALG00000029632* | 5.67671256 | 0.54389733 | 0.00201376 | 0.02306772 |
| *ENSGALG00000033364* | 5.6527931 | -0.1189022 | 0.00485411 | 0.04036693 |
| *ARFIP2* | 5.64543733 | 2.33966717 | 3.89E-05 | 0.00185762 |
| *GGA1* | 5.63564367 | -0.3907454 | 0.00585496 | 0.04499193 |
| *ENSGALG00000032071* | 5.61644068 | 0.07755167 | 0.00342105 | 0.03256901 |
| *ENSGALG00000034140* | 5.61213141 | -0.333543 | 0.00394198 | 0.03554314 |
| *SHOX2* | 5.59097311 | -0.2416237 | 0.00374285 | 0.03433856 |
| *PLAGL2* | 5.58668245 | -0.1701641 | 0.00368316 | 0.03405596 |
| *ENSGALG00000019552* | 5.57641301 | 4.37645796 | 0.00018987 | 0.00531913 |
| *ENSGALG00000030562* | 5.56572671 | -0.312947 | 0.00190326 | 0.0221791 |
| *RCAN2* | 5.53949296 | -0.2040549 | 0.00365865 | 0.03391477 |
| *ENSGALG00000025635* | 5.52469915 | -0.2537932 | 0.00355081 | 0.0333333 |
| *LDLRAD1* | 5.51594456 | 0.25603716 | 0.00155756 | 0.01939353 |
| *RNF150* | 5.51593349 | 0.97400503 | 4.65E-05 | 0.00203562 |

| | | | | |
|---|---|---|---|---|
| *ENSGALG00000041409* | 5.51273784 | 2.03453416 | 2.26E-06 | 0.00023689 |
| *ENSGALG00000021730* | 5.50935645 | 0.19070197 | 0.00575319 | 0.04449834 |
| *TPRN* | 5.50434152 | -0.347135 | 0.00497501 | 0.04082453 |
| *ENSGALG00000038428* | 5.47120275 | 0.83283557 | 0.00314519 | 0.03064396 |
| *ZFP804A* | 5.46469308 | 1.06097098 | 0.00039842 | 0.00857898 |
| *ENSGALG00000025357* | 5.4623838 | -0.1569882 | 0.00672478 | 0.04957555 |
| *HTRA1* | 5.41975134 | 4.82737652 | 0.00034986 | 0.00781895 |
| *ARAP3* | 5.40285961 | 1.39323009 | 0.0062645 | 0.04736096 |
| *ITPRIPL2* | 5.39671334 | 0.03143656 | 0.00636965 | 0.04788201 |
| *KLB* | 5.39500851 | 0.26658963 | 0.00552784 | 0.04346993 |
| *ENSGALG00000039735* | 5.3726239 | -0.3350168 | 0.00488799 | 0.04042735 |
| *ENSGALG00000034052* | 5.2899364 | 1.5372307 | 1.20E-05 | 0.00079778 |
| *ENSGALG00000009603* | 5.28885159 | 0.93431161 | 7.20E-05 | 0.00279127 |
| *ENSGALG00000017850* | 5.28577212 | 0.52002781 | 0.00473346 | 0.03971471 |
| *ENSGALG00000028165* | 5.28312679 | -0.2650333 | 0.00471212 | 0.03963277 |
| *ENSGALG00000021171* | 5.23306211 | 5.11399439 | 2.73E-12 | 3.36E-09 |
| *ENSGALG00000008905* | 5.22415238 | 4.60527662 | 8.06E-06 | 0.00057887 |
| *ENSGALG00000038456* | 5.15095641 | 0.70868829 | 5.84E-05 | 0.00240767 |
| *WNK4* | 5.12459237 | 2.41006697 | 1.12E-06 | 0.00013716 |
| *ELL2* | 5.10478313 | 3.22260372 | 2.74E-09 | 1.35E-06 |
| *SH3GL1* | 5.09430841 | 1.0865345 | 8.65E-05 | 0.00317681 |
| *ENSGALG00000042676* | 5.08864644 | 0.4122418 | 0.00293106 | 0.029471 |
| *ENSGALG00000010801* | 5.06109252 | 1.80179201 | 1.07E-05 | 0.00072503 |
| *ENSGALG00000014508* | 5.04435563 | 4.61055657 | 2.47E-09 | 1.35E-06 |
| *BTBD11* | 5.0280853 | 2.07122923 | 3.60E-05 | 0.00178533 |
| *SDC1* | 5.01617374 | 1.41718756 | 2.62E-05 | 0.00143661 |
| *SLCO2A1* | 4.99759799 | 2.2678566 | 0.00053851 | 0.01040755 |
| *RNF146* | 4.97564901 | 1.1929565 | 0.00015676 | 0.00469841 |
| *RND2* | 4.97301705 | 0.62713272 | 0.00138446 | 0.01801838 |
| *ENSGALG00000022622* | 4.96247416 | 6.49751212 | 2.08E-14 | 3.69E-11 |
| *FBP2* | 4.89763671 | 0.94916214 | 0.00019738 | 0.00542439 |
| *ENSGALG00000007416* | 4.87662932 | 2.08398177 | 0.00044552 | 0.00921822 |
| *PXN* | 4.86433142 | 1.52055505 | 0.00011611 | 0.00381995 |
| *SEMA3B* | 4.85831049 | 0.38880816 | 0.0016325 | 0.02003037 |
| *MDFI* | 4.85431473 | 2.65170912 | 0.0002292 | 0.00590029 |
| *ENSGALG00000039113* | 4.85134315 | 1.63335355 | 0.00038589 | 0.00834744 |
| *HS3ST1* | 4.79393696 | 2.62599636 | 4.13E-06 | 0.00037714 |
| *VIT* | 4.79225597 | 3.207623 | 1.80E-05 | 0.00109562 |
| *ENSGALG00000026650* | 4.7320519 | 4.91245367 | 7.16E-06 | 0.00053283 |
| *HOXD12* | 4.71336052 | 1.80453546 | 0.00549205 | 0.04326056 |

| | | | | |
|---|---|---|---|---|
| *B3GAT2* | 4.71090924 | 1.83109898 | 5.16E-07 | 7.77E-05 |
| *ENSGALG00000016813* | 4.70746855 | 2.92564888 | 5.99E-05 | 0.00246189 |
| *GPR171* | 4.69300714 | 2.72070158 | 6.07E-09 | 2.38E-06 |
| *SLC22A23* | 4.67786733 | 1.79619335 | 0.00123945 | 0.01717271 |
| *HPSE2* | 4.63142967 | 1.06999478 | 0.00302048 | 0.02994053 |
| *MMP15* | 4.61730575 | 1.80065029 | 0.00134237 | 0.01785699 |
| *MGST2* | 4.59214084 | 1.57217588 | 0.00088656 | 0.01407647 |
| *ENSGALG00000025438* | 4.5571044 | 2.0311416 | 0.00132974 | 0.01774785 |
| *ENSGALG00000028659* | 4.55256609 | 1.16703886 | 0.00391141 | 0.03538023 |
| *SPDYA* | 4.54702489 | 0.83313763 | 0.00227035 | 0.02501448 |
| *FBXW4* | 4.52246651 | 1.15679323 | 0.00099077 | 0.01502907 |
| *HORMAD1* | 4.50301002 | 1.26959382 | 5.70E-05 | 0.00238047 |
| *ENSGALG00000028653* | 4.50226074 | 0.91429505 | 0.00010109 | 0.00347123 |
| *ENSGALG00000014509* | 4.48105843 | 3.68392716 | 0.0001427 | 0.00435094 |
| *ENSGALG00000027796* | 4.47165019 | 0.78151227 | 0.00132202 | 0.01771081 |
| *OLAH* | 4.46253635 | 1.8531412 | 2.79E-05 | 0.00148586 |
| *TWF2* | 4.46025261 | 1.67336862 | 0.00085928 | 0.01379745 |
| *RGS1* | 4.4558975 | 0.5425121 | 0.00654259 | 0.04863797 |
| *SLC16A2* | 4.4530915 | 2.71939625 | 3.94E-07 | 6.33E-05 |
| *NDST2* | 4.44621613 | 1.51094744 | 0.00416554 | 0.03653435 |
| *ENSGALG00000026107* | 4.44295885 | 1.16706624 | 0.00022583 | 0.00584244 |
| *ENSGALG00000006609* | 4.43842658 | 3.4222907 | 4.62E-05 | 0.00203183 |
| *ENSGALG00000015627* | 4.43471029 | 5.39118817 | 0.0002895 | 0.00675834 |
| *RANBP10* | 4.41719909 | 1.91197485 | 4.67E-07 | 7.33E-05 |
| *ENKUR* | 4.41466752 | 1.47976447 | 4.11E-05 | 0.00190946 |
| *ENSGALG00000030479* | 4.40718342 | 0.03480703 | 0.004528 | 0.03865877 |
| *CYTIP* | 4.37971392 | 2.98150965 | 4.15E-05 | 0.00191949 |
| *SCARB1* | 4.37247157 | 0.7650324 | 0.00330322 | 0.03174635 |
| *GPR142* | 4.36516606 | -0.3608959 | 0.00444167 | 0.03822157 |
| *ENSGALG00000037329* | 4.3642489 | 1.03215456 | 0.0053751 | 0.04281621 |
| *ENSGALG00000044168* | 4.36263701 | 0.35494304 | 0.00171638 | 0.02075351 |
| *SLC37A4* | 4.34871774 | 0.87218861 | 0.00067647 | 0.01191779 |
| *ENSGALG00000039342* | 4.3432666 | 1.10599378 | 0.00158442 | 0.0195762 |
| *GID4* | 4.31455265 | 1.01254402 | 0.00136346 | 0.01786165 |
| *ENSGALG00000041512* | 4.26704433 | 1.14654094 | 0.00153506 | 0.01923521 |
| *DALRD3* | 4.23034285 | 1.40584467 | 4.73E-05 | 0.00205071 |
| *NDRG1* | 4.21911157 | 4.06771724 | 6.44E-07 | 9.02E-05 |
| *ENSGALG00000029931* | 4.21262946 | 1.2983822 | 0.00076459 | 0.01286168 |
| *SOD3* | 4.20896812 | 3.74417111 | 0.00010667 | 0.00361024 |
| *ENSGALG00000045096* | 4.19841581 | -0.522316 | 0.00195945 | 0.02262885 |

| | | | | |
|---|---|---|---|---|
| *MTMR6* | 4.19763111 | 1.26524239 | 0.00156087 | 0.01939353 |
| *ENSGALG00000008552* | 4.19573824 | 1.5147222 | 0.00215524 | 0.02405644 |
| *LOXL4* | 4.19210017 | 3.7870593 | 0.00036803 | 0.00808462 |
| *CCNG2* | 4.18720193 | 3.27196842 | 0.00013749 | 0.00424467 |
| *ENSGALG00000039629* | 4.17392408 | 6.25196417 | 0.00273829 | 0.028247 |
| *ENSGALG00000008309* | 4.16600301 | 0.06538409 | 0.00271073 | 0.0280532 |
| *ENSGALG00000044318* | 4.1556124 | 4.80060276 | 4.84E-07 | 7.37E-05 |
| *PPP1R15B* | 4.1362939 | 1.13251559 | 0.00144692 | 0.01847458 |
| *ENSGALG00000044656* | 4.12659443 | 2.03577245 | 0.00028767 | 0.00673194 |
| *ENSGALG00000025898* | 4.12451339 | 2.96697116 | 0.0001567 | 0.00469841 |
| *ANKRD11* | 4.08882984 | 1.65423958 | 0.00235581 | 0.02559724 |
| *CHSY3* | 4.07451117 | 0.81686603 | 0.00028472 | 0.00673194 |
| *ENSGALG00000040291* | 4.06917904 | 0.82677914 | 0.00037145 | 0.00814715 |
| *DHCR24* | 4.06536569 | 0.93487085 | 0.00260785 | 0.02742988 |
| *ENSGALG00000012055* | 4.03146528 | 2.73127984 | 0.00062773 | 0.01140619 |
| *KIF9* | 4.02074152 | 2.95481607 | 2.37E-05 | 0.00132645 |
| *ENSGALG00000025680* | 3.99870937 | 0.08540774 | 0.00179026 | 0.02128305 |
| *ABHD12B* | 3.99321175 | 1.58638625 | 0.00154218 | 0.01928422 |
| *PGAM5* | 3.99241931 | 0.41540847 | 0.00288434 | 0.02914901 |
| *TMX1* | 3.97395589 | 0.72456823 | 0.0004216 | 0.00894641 |
| *FAR2* | 3.96220907 | 2.48713649 | 1.34E-05 | 0.00087585 |
| *ENSGALG00000038656* | 3.96164851 | 3.7455916 | 2.63E-05 | 0.00143865 |
| *ENSGALG00000030096* | 3.95615135 | 2.67650806 | 0.0011493 | 0.01643991 |
| *ACKR3* | 3.94874966 | 4.51312875 | 1.77E-09 | 1.05E-06 |
| *GRIN3A* | 3.94835705 | 2.71106843 | 0.00060069 | 0.01115215 |
| *9930104L06RIK* | 3.93523104 | 3.0665411 | 8.25E-08 | 1.98E-05 |
| *ENSGALG00000040321* | 3.91730537 | 0.38074748 | 0.00157777 | 0.01952017 |
| *SUSD5* | 3.90053573 | 4.3173845 | 2.70E-06 | 0.0002687 |
| *ERMN* | 3.89404448 | 0.17877412 | 0.00491166 | 0.04049254 |
| *PCYOX1* | 3.88101437 | 6.01060761 | 5.07E-09 | 2.05E-06 |
| *CRELD1* | 3.88095913 | 1.68640727 | 0.00020551 | 0.00558024 |
| *ENSGALG00000016732* | 3.87936749 | 1.25066898 | 0.00055764 | 0.01066067 |
| *VASH1* | 3.87013367 | 0.40691557 | 0.00192532 | 0.0223992 |
| *ENSGALG00000046617* | 3.86492267 | 2.8121983 | 4.75E-06 | 0.00041701 |
| *ENSGALG00000023122* | 3.85536335 | 0.67768179 | 0.00100574 | 0.01517143 |
| *SRSF4* | 3.84877563 | 3.05795562 | 6.89E-08 | 1.71E-05 |
| *CLDN1* | 3.81588824 | 3.54413383 | 0.0026895 | 0.02791515 |
| *EGR1* | 3.80421234 | 5.09538752 | 0.00019662 | 0.0054223 |
| *DNALI1* | 3.79630525 | 2.53512937 | 6.80E-07 | 9.43E-05 |
| *TNK2* | 3.78969172 | 3.20010617 | 0.00014866 | 0.00451299 |

| | | | | |
|---|---|---|---|---|
| *ENSGALG00000006127* | 3.78726916 | 1.04297179 | 0.00171576 | 0.02075351 |
| *ENSGALG00000001412* | 3.77576787 | 2.51895553 | 5.10E-05 | 0.00218507 |
| *ENSGALG00000046360* | 3.76207534 | 4.32493319 | 1.87E-07 | 3.60E-05 |
| *ENSGALG00000046293* | 3.74653843 | 2.60079622 | 0.00010195 | 0.0034921 |
| *GNB3* | 3.7377117 | 0.90592593 | 0.0013483 | 0.01785699 |
| *CHSY1* | 3.73257159 | 0.62328119 | 0.00482488 | 0.04022248 |
| *KBTBD2* | 3.72767725 | 2.79051116 | 7.12E-07 | 9.69E-05 |
| *ZBTB20* | 3.71350745 | 3.19107636 | 5.77E-06 | 0.00047487 |
| *GM525* | 3.70588274 | 5.76743102 | 0.00426479 | 0.03710573 |
| *SLC22A16* | 3.70464006 | 5.78477734 | 1.34E-08 | 4.50E-06 |
| *SLITRK2* | 3.69977107 | 1.93231926 | 0.00184175 | 0.02171271 |
| *FUK* | 3.67003446 | 2.56437345 | 1.89E-05 | 0.00113933 |
| *ENSGALG00000000452* | 3.66806584 | 2.29474227 | 0.00011508 | 0.00379487 |
| *ENSGALG00000036034* | 3.66597087 | 6.44644771 | 9.90E-05 | 0.00344823 |
| *AK4* | 3.65812473 | 0.87344233 | 0.00353752 | 0.03325269 |
| *NBEAL2* | 3.65543931 | 1.29336277 | 0.00086517 | 0.01383871 |
| *PCOLCE* | 3.63529763 | 2.48995848 | 0.00089896 | 0.01419091 |
| *CILP* | 3.63163932 | 0.794374 | 0.00412408 | 0.036351 |
| *ZAR1L* | 3.62846774 | 3.50227175 | 0.0009034 | 0.01421624 |
| *ENSGALG00000017866* | 3.61643119 | 1.81390108 | 0.0004803 | 0.00973458 |
| *PLXNA4* | 3.60169437 | 2.38505165 | 0.00370373 | 0.0341569 |
| *ENSGALG00000043625* | 3.59745339 | 5.39411669 | 5.11E-11 | 4.82E-08 |
| *NLK* | 3.58937327 | 1.55326685 | 0.0002361 | 0.00600737 |
| *ENSGALG00000029248* | 3.57880135 | 1.3266832 | 0.00020877 | 0.00560435 |
| *UPK1B* | 3.57743004 | 3.06077754 | 0.00099885 | 0.0151292 |
| *ATXN1* | 3.57210783 | 0.15908051 | 0.00356827 | 0.03345278 |
| *ENSGALG00000003500* | 3.56909035 | 2.24842136 | 0.00018255 | 0.00521718 |
| *APOA1* | 3.5647861 | 6.8088441 | 0.00020976 | 0.00562026 |
| *SDCCAG3* | 3.55836539 | 0.7074505 | 0.00131887 | 0.01768535 |
| *PHKA1* | 3.54584376 | 2.75349562 | 0.00030834 | 0.00709284 |
| *ANXA6* | 3.5403738 | 4.23564626 | 4.52E-05 | 0.00201014 |
| *BDNF* | 3.53352021 | 1.24493104 | 0.00125246 | 0.01726953 |
| *DLL1* | 3.532048 | 1.16023021 | 0.00174074 | 0.0209088 |
| *PLCXD3* | 3.52060959 | 1.64341 | 0.00055738 | 0.01066067 |
| *SUFU* | 3.50400377 | 3.55493194 | 4.77E-07 | 7.37E-05 |
| *ENSGALG00000012468* | 3.50325948 | 1.62741828 | 0.00314937 | 0.03066352 |
| *G0S2* | 3.48702029 | 2.15171785 | 0.00060642 | 0.0111852 |
| *ADIPOQ* | 3.47887881 | 1.4410033 | 0.00410232 | 0.0362722 |
| *ENSGALG00000043364* | 3.46019528 | 2.72057633 | 1.36E-05 | 0.0008807 |
| *LRRC8C* | 3.45851807 | 3.35655858 | 6.21E-07 | 8.92E-05 |

| | | | | |
|---|---|---|---|---|
| *CRTAC1* | 3.45632373 | 2.29994793 | 0.00354343 | 0.03328614 |
| *EPB41L4A* | 3.45493649 | 3.50363636 | 2.20E-05 | 0.00126546 |
| *PDK4* | 3.45271479 | 5.15513679 | 0.00013963 | 0.00427547 |
| *DDIT4* | 3.4500025 | 2.96150226 | 0.0015285 | 0.019196 |
| *ENSGALG00000003690* | 3.44272788 | 1.94237934 | 0.00230508 | 0.02527899 |
| *ZHX2* | 3.42698824 | 1.00014899 | 0.00197619 | 0.02271095 |
| *ENSGALG00000027159* | 3.42609346 | 3.70918113 | 9.37E-07 | 0.00012165 |
| *PIGH* | 3.41986435 | 1.15600811 | 0.00066536 | 0.01184002 |
| *ZADH2* | 3.41936408 | 2.33094315 | 0.00376682 | 0.0344468 |
| *LRRC41* | 3.4127415 | 1.46228957 | 0.00011042 | 0.00368421 |
| *GOLGA2* | 3.4119318 | 2.91397627 | 6.39E-06 | 0.00050802 |
| *ENSGALG00000014659* | 3.40962066 | 2.03347999 | 0.00103635 | 0.01541672 |
| *ENSGALG00000045589* | 3.39699603 | 1.28714351 | 0.00460329 | 0.03907857 |
| *ENSGALG00000032114* | 3.39676713 | 3.08834852 | 7.46E-05 | 0.0028509 |
| *ENSGALG00000037631* | 3.38842123 | 2.63415086 | 0.00174252 | 0.0209088 |
| *NUDT12* | 3.38322253 | 1.12538198 | 0.00072196 | 0.01235011 |
| *FAAP100* | 3.3656917 | 1.58305821 | 0.00103787 | 0.01542304 |
| *SARS2* | 3.3636033 | 1.46302779 | 0.00046329 | 0.00948493 |
| *TIMP4* | 3.35153462 | 6.76974963 | 1.75E-07 | 3.44E-05 |
| *HOXD10* | 3.34708901 | 3.36141004 | 0.00035457 | 0.0078622 |
| *ENSGALG00000015422* | 3.34602193 | 4.84879048 | 1.61E-05 | 0.00100747 |
| *CDHR3* | 3.33307742 | 3.26636897 | 0.00108215 | 0.01579894 |
| *TEK* | 3.32052838 | 3.40857262 | 1.46E-05 | 0.00093605 |
| *TGFBR2* | 3.31355197 | 3.92634876 | 0.00228811 | 0.02515918 |
| *TTLL4* | 3.3109103 | 1.24798295 | 0.00330996 | 0.03178954 |
| *ADAMTS13* | 3.30856964 | 2.80466806 | 0.00154795 | 0.01932817 |
| *VIL1* | 3.30336603 | 3.08299328 | 5.44E-05 | 0.00229245 |
| *ENSGALG00000008153* | 3.30246012 | 0.75932867 | 0.00062087 | 0.01131895 |
| *ENSGALG00000034100* | 3.30225717 | 3.95650915 | 0.00019286 | 0.00536019 |
| *ENSGALG00000036780* | 3.29978177 | 4.75187425 | 0.00373081 | 0.03426292 |
| *FAM20A* | 3.29437652 | 4.71104336 | 0.00042511 | 0.00894978 |
| *ENSGALG00000006300* | 3.28928299 | 8.46411296 | 1.57E-07 | 3.14E-05 |
| *BMF* | 3.28401349 | 5.26634656 | 4.61E-06 | 0.00040989 |
| *SOCS3* | 3.28365343 | 2.8198034 | 0.00336472 | 0.03220513 |
| *ITM2C* | 3.27502291 | 4.36806314 | 8.19E-07 | 0.00011031 |
| *HTRA3* | 3.27183013 | 3.96414547 | 0.00060896 | 0.01121741 |
| *YIPF1* | 3.26769949 | 3.37683561 | 9.29E-07 | 0.00012165 |
| *FAM83H* | 3.26735107 | 1.30685555 | 0.00020752 | 0.00559197 |
| *NFKBIE* | 3.25705166 | 1.87448506 | 0.00110155 | 0.01593422 |
| *ENSGALG00000004769* | 3.25678003 | 1.50572438 | 0.00022939 | 0.00590029 |

| | | | | |
|---|---|---|---|---|
| *IKBIP* | 3.24868785 | 3.23447942 | 2.54E-05 | 0.00140402 |
| *TBC1D4* | 3.24847577 | 5.30970653 | 2.04E-06 | 0.00022227 |
| *RCN1* | 3.24299549 | 2.20665771 | 0.00063236 | 0.01145455 |
| *ENSGALG00000042216* | 3.23959869 | 2.25668969 | 0.00458589 | 0.03898837 |
| *ENSGALG00000009026* | 3.23338459 | 5.42860125 | 2.36E-07 | 4.23E-05 |
| *BOD1* | 3.22692975 | 2.44524464 | 0.00062808 | 0.01140619 |
| *TRAF3IP2* | 3.22686437 | 4.10120689 | 6.36E-06 | 0.00050802 |
| *SCIN* | 3.213437 | 9.59433685 | 1.41E-07 | 3.00E-05 |
| *LRRTM2* | 3.21098749 | 1.74910927 | 0.00020844 | 0.00560435 |
| *ENSGALG00000029446* | 3.20945919 | 2.76463367 | 0.00262447 | 0.02752292 |
| *RGCC* | 3.20429007 | 5.28007801 | 4.82E-06 | 0.00041874 |
| *SDC4* | 3.20299791 | 4.58852911 | 4.26E-08 | 1.16E-05 |
| *FAM69C* | 3.19384326 | 3.8501278 | 0.00052024 | 0.01017539 |
| *AMBRA1* | 3.19098185 | 2.11093457 | 0.00016153 | 0.00477065 |
| *TMEM39B* | 3.1707578 | 3.81067211 | 4.69E-08 | 1.25E-05 |
| *SFRP4* | 3.16451795 | 2.86611372 | 5.37E-05 | 0.00227118 |
| *SERINC5* | 3.15642595 | 4.30561253 | 0.00020741 | 0.00559197 |
| *ENSGALG00000039326* | 3.1501649 | 4.16718506 | 2.04E-06 | 0.00022227 |
| *CHD5* | 3.1451556 | 2.08937483 | 0.00057012 | 0.01080527 |
| *RGMB* | 3.13503016 | 0.96780267 | 0.00275428 | 0.0283793 |
| *MAPK1* | 3.13459484 | 3.25597955 | 2.38E-05 | 0.00132645 |
| *ENSGALG00000032803* | 3.12844026 | 3.93239039 | 0.00011209 | 0.00370502 |
| *ENSGALG00000014773* | 3.12026531 | 3.58508269 | 4.46E-05 | 0.0019947 |
| *ATP2B2* | 3.12019576 | 6.87774919 | 2.92E-05 | 0.0015387 |
| *ENSGALG00000021232* | 3.11516452 | 0.58495777 | 0.00246082 | 0.02635559 |
| *CTDNEP1* | 3.11290883 | 2.83113877 | 0.00171302 | 0.02074842 |
| *HSD17B3* | 3.11182762 | 1.89662013 | 0.00112054 | 0.01619226 |
| *ENSGALG00000033771* | 3.11049485 | 1.60738048 | 0.00100592 | 0.01517143 |
| *SLC26A11* | 3.10934946 | 2.2904658 | 0.00202316 | 0.02308464 |
| *ZFP827* | 3.10611348 | 2.31281955 | 0.00016579 | 0.00486601 |
| *TMBIM1* | 3.10496522 | 2.62404446 | 0.00077054 | 0.01289978 |
| *YIPF3* | 3.10332051 | 4.75808436 | 1.22E-08 | 4.22E-06 |
| *ELOVL6* | 3.1008222 | 6.11746903 | 1.44E-07 | 3.00E-05 |
| *BNIP3* | 3.09799949 | 6.67134399 | 1.00E-08 | 3.63E-06 |
| *PLA2R1* | 3.09242849 | 7.10737087 | 6.96E-09 | 2.66E-06 |
| *HOXD8* | 3.09016285 | 4.08651713 | 1.35E-06 | 0.00016074 |
| *ENSGALG00000007526* | 3.09013846 | 4.20362354 | 0.00022232 | 0.00579213 |
| *KIF13B* | 3.07297774 | 3.25095081 | 0.00054659 | 0.01052421 |
| *DHTKD1* | 3.07117256 | 3.7029192 | 9.46E-05 | 0.0033724 |
| *ENSGALG00000018242* | 3.06916264 | 3.13370534 | 9.11E-05 | 0.00327911 |

| | | | | |
|---|---|---|---|---|
| *AIFM1* | 3.06886503 | 2.85288806 | 0.00363917 | 0.03382614 |
| *ENSGALG00000039455* | 3.05727356 | 1.82618731 | 0.00024747 | 0.00626297 |
| *EFNA5* | 3.04876246 | 1.13151248 | 0.00387822 | 0.03513505 |
| *YDJC* | 3.04536822 | 1.49542354 | 0.00027857 | 0.00664403 |
| *ENSGALG00000013600* | 3.04308803 | 2.23558507 | 4.02E-05 | 0.00189729 |
| *ENSGALG00000044031* | 3.04249592 | 1.52187782 | 0.00196394 | 0.02264374 |
| *FAM174A* | 3.04230857 | 2.94523072 | 0.00037908 | 0.00826323 |
| *PLEKHA1* | 3.03998462 | 2.93268886 | 0.00550403 | 0.04333087 |
| *ENSGALG00000018331* | 3.03814598 | 2.41884607 | 0.00311091 | 0.03045677 |
| *MAP2K4* | 3.02181288 | 1.92427401 | 0.00083634 | 0.01358408 |
| *MCAM* | 3.01317546 | 1.97655834 | 0.00303209 | 0.02995457 |
| *PAM* | 3.01225756 | 3.33080312 | 4.75E-06 | 0.00041701 |
| *MUL1* | 3.0054523 | 1.65661663 | 0.00202009 | 0.02308464 |
| *ENSGALG00000000094* | 2.98917038 | 2.94334502 | 3.67E-05 | 0.00179006 |
| *IGF2BP2* | 2.98599893 | 1.84632137 | 0.00263519 | 0.02759231 |
| *TELO2* | 2.96806909 | 1.83289235 | 0.00099117 | 0.01502907 |
| *ACKR2* | 2.95962859 | 0.82333804 | 0.00263694 | 0.02759231 |
| *NRAP* | 2.95853084 | 5.50597494 | 0.00529085 | 0.04241012 |
| *ENSGALG00000024296* | 2.95311179 | 2.40507178 | 0.00182566 | 0.02160618 |
| *ENSGALG00000039722* | 2.946017 | 0.72975391 | 0.00305266 | 0.03007763 |
| *SPRTN* | 2.94045837 | 1.78845342 | 0.00121037 | 0.01692588 |
| *TTC28* | 2.9296523 | 2.31746139 | 0.00040157 | 0.00863376 |
| *CTBS* | 2.92829469 | 5.43766138 | 3.43E-09 | 1.55E-06 |
| *PDCD10* | 2.92503125 | 4.64291612 | 2.23E-07 | 4.04E-05 |
| *CTC1* | 2.91939051 | 2.68775938 | 0.00020129 | 0.00549727 |
| *SHC4* | 2.9165557 | 2.52151918 | 0.00058921 | 0.01106971 |
| *GTF3C4* | 2.91338549 | 2.53130011 | 0.00059788 | 0.01111602 |
| *NINJ2* | 2.91015034 | 7.2408287 | 3.13E-06 | 0.00029674 |
| *ENSGALG00000036014* | 2.90969342 | 2.86655255 | 0.00085293 | 0.01374306 |
| *EPHB2* | 2.90597275 | 2.14774683 | 0.0018989 | 0.02214654 |
| *NEDD9* | 2.89268928 | 2.60945197 | 0.00106482 | 0.0156719 |
| *CD200* | 2.89097072 | 6.12000752 | 4.46E-06 | 0.00040153 |
| *ZFP511* | 2.88571188 | 4.09934902 | 4.13E-06 | 0.00037714 |
| *SLC12A2* | 2.87172608 | 2.94181286 | 0.00038388 | 0.00832642 |
| *ENSGALG00000038238* | 2.87156635 | 5.04021993 | 4.56E-05 | 0.00202199 |
| *KDELC2* | 2.85839281 | 4.04678938 | 9.97E-05 | 0.00346454 |
| *DDIAS* | 2.8524824 | 4.27742445 | 2.59E-05 | 0.00142386 |
| *ENSGALG00000033044* | 2.83520578 | 3.19114382 | 5.36E-05 | 0.00227118 |
| *SNORD47* | 2.83463746 | 2.80976356 | 4.05E-05 | 0.00189742 |
| *FBXL5* | 2.81842998 | 6.76515299 | 1.88E-07 | 3.60E-05 |

| | | | | |
|---|---|---|---|---|
| *MBD5* | 2.81767335 | 2.98563774 | 0.0001331 | 0.0041585 |
| *RHOF* | 2.81613368 | 2.8014498 | 0.00089302 | 0.01414734 |
| *ENSGALG00000017204* | 2.81600978 | 0.99731431 | 0.0025201 | 0.02678572 |
| *ENSGALG00000046223* | 2.80083755 | 0.98052825 | 0.00423023 | 0.03691864 |
| *ENSGALG00000036992* | 2.79568652 | 1.50639659 | 0.00186329 | 0.02188325 |
| *ENSGALG00000031858* | 2.7954122 | 2.91583449 | 3.74E-05 | 0.00181703 |
| *SPIDR* | 2.79287356 | 3.7128226 | 0.00109986 | 0.01592594 |
| *SLC38A1* | 2.78998479 | 5.49781096 | 1.02E-06 | 0.00012895 |
| *NUMB* | 2.78468667 | 4.09441926 | 8.71E-08 | 2.05E-05 |
| *EPB41* | 2.78376867 | 4.3209711 | 0.00013274 | 0.0041585 |
| *MORN2* | 2.78331826 | 2.37853385 | 0.00178608 | 0.02126519 |
| *IRX6* | 2.77919093 | 5.02472531 | 3.44E-05 | 0.00172046 |
| *ENSGALG00000005043* | 2.77827725 | 1.03316765 | 0.00499349 | 0.04087872 |
| *ANGPTL1* | 2.77529236 | 5.75722252 | 1.08E-06 | 0.00013266 |
| *ENSGALG00000007382* | 2.77118075 | 1.85361441 | 0.00280309 | 0.02874121 |
| *CUTAL* | 2.7705209 | 4.65336142 | 0.00024949 | 0.00628025 |
| *ENSGALG00000002207* | 2.76831718 | 3.1143834 | 0.00449775 | 0.03854011 |
| *ANO6* | 2.76128923 | 5.70844626 | 0.00150828 | 0.01900654 |
| *DLX6* | 2.75718582 | 2.17759451 | 0.00118379 | 0.01674267 |
| *ENSGALG00000007178* | 2.75413955 | 3.1110038 | 0.00379806 | 0.03462062 |
| *FAM129B* | 2.74733298 | 2.36849006 | 0.00330174 | 0.03174635 |
| *ENSGALG00000039209* | 2.73476075 | 4.59509975 | 0.00280842 | 0.02874866 |
| *ENSGALG00000035332* | 2.73316461 | 0.76927057 | 0.00506439 | 0.04127065 |
| *CNMD* | 2.72667678 | 11.0939294 | 0.00018924 | 0.00531913 |
| *ZFP36L2* | 2.71540358 | 3.01026948 | 0.00013486 | 0.00419553 |
| *PYGL* | 2.71137688 | 3.72617403 | 0.0001298 | 0.00410513 |
| *ENSGALG00000006282* | 2.70510803 | 4.1749622 | 1.96E-05 | 0.001159 |
| *ENSGALG00000004646* | 2.70221688 | 2.19875337 | 0.00179614 | 0.021335 |
| *ENSGALG00000045452* | 2.69535431 | 1.27126051 | 0.00462676 | 0.03916668 |
| *GAB1* | 2.69461329 | 7.77005767 | 2.68E-07 | 4.62E-05 |
| *ATP8B5* | 2.69073017 | 2.14731335 | 0.00145388 | 0.01851305 |
| *CCDC83* | 2.68623538 | 3.36165169 | 0.00131477 | 0.01768443 |
| *RBM33* | 2.66899527 | 4.41780437 | 3.73E-07 | 6.07E-05 |
| *ENSGALG00000024498* | 2.66672107 | 2.04005614 | 0.0015785 | 0.01952017 |
| *ZMYND12* | 2.66148497 | 1.48038623 | 0.00083609 | 0.01358408 |
| *AMOTL1* | 2.66056686 | 1.47295592 | 0.00517445 | 0.04183022 |
| *CSPG4* | 2.65911828 | 3.33867934 | 0.00181071 | 0.02147197 |
| *RBM15* | 2.6564067 | 1.54586702 | 0.00529115 | 0.04241012 |
| *TRMT13* | 2.65164728 | 5.76721139 | 2.56E-06 | 0.00025685 |
| *WDR26* | 2.64835514 | 4.11896538 | 2.90E-05 | 0.00153669 |

| | | | | |
|---|---|---|---|---|
| *UBAP1* | 2.63992731 | 1.67227034 | 0.00305063 | 0.03007763 |
| *ENSGALG00000045039* | 2.62026616 | 1.61055393 | 0.00257 | 0.02716508 |
| *TLNRD1* | 2.61903889 | 4.68251366 | 0.00043317 | 0.00907852 |
| *SKIV2L2* | 2.61252978 | 3.92435915 | 0.00012833 | 0.00407045 |
| *SLC16A7* | 2.61206584 | 3.72332218 | 0.00032911 | 0.00750963 |
| *NPR3* | 2.61165658 | 3.89052414 | 0.00098657 | 0.01500919 |
| *TET2* | 2.60911495 | 3.56620768 | 0.00070046 | 0.01212987 |
| *ENSGALG00000039459* | 2.60843611 | 1.93541249 | 0.00035142 | 0.00784146 |
| *DCDC2B* | 2.60559587 | 2.88821541 | 0.0004195 | 0.00893924 |
| *EFEMP1* | 2.59678487 | 3.95876816 | 0.00334531 | 0.03206374 |
| *CDKN2AIP* | 2.58935657 | 3.22939564 | 0.00250667 | 0.02668311 |
| *ENSGALG00000044504* | 2.58547477 | 5.00074582 | 4.62E-05 | 0.00203183 |
| *TIMP2* | 2.57520121 | 4.77553042 | 0.0020312 | 0.02315502 |
| *CAMK4* | 2.57501712 | 3.88900454 | 3.85E-05 | 0.00185207 |
| *ENSGALG00000021475* | 2.57115411 | 3.18749238 | 0.00026548 | 0.00645323 |
| *EXT1* | 2.55463538 | 3.76693017 | 0.00468942 | 0.03951231 |
| *RANBP9* | 2.55447612 | 6.85556848 | 2.45E-07 | 4.30E-05 |
| *ARHGAP45* | 2.55292245 | 4.39361133 | 0.00069369 | 0.01208572 |
| *RAB31* | 2.55135438 | 3.10260449 | 0.00025553 | 0.00634013 |
| *FAM171A1* | 2.55044667 | 2.85636125 | 8.99E-05 | 0.0032536 |
| *ENSGALG00000044782* | 2.54822128 | 3.06796644 | 0.00419915 | 0.03678349 |
| *RNF215* | 2.54803146 | 1.14313997 | 0.00648983 | 0.04847496 |
| *ENSGALG00000023818* | 2.53899471 | 3.21383497 | 0.00150063 | 0.0189379 |
| *ENSGALG00000043779* | 2.53792538 | 3.56726558 | 0.0001393 | 0.00427482 |
| *DNAJB9* | 2.53149344 | 4.99328447 | 0.00049686 | 0.00995234 |
| *P3H1* | 2.53056485 | 5.90744538 | 1.95E-05 | 0.001159 |
| *DDX51* | 2.52051688 | 3.63715883 | 3.43E-05 | 0.00172033 |
| *RPS6KC1* | 2.5196407 | 2.21453607 | 0.0002193 | 0.00575913 |
| *ENSGALG00000041867* | 2.51643827 | 3.59461355 | 0.00462368 | 0.03916668 |
| *PTTG1IP* | 2.51074364 | 6.16874944 | 6.03E-07 | 8.79E-05 |
| *E4F1* | 2.50080851 | 3.04507434 | 0.00168277 | 0.02048716 |
| *ASAH1* | 2.49776978 | 6.36675204 | 1.20E-06 | 0.0001444 |
| *TMEM243* | 2.49712823 | 2.73309135 | 0.00087448 | 0.01395339 |
| *PM20D1* | 2.49259031 | 5.06471474 | 2.85E-09 | 1.35E-06 |
| *ENSGALG00000011740* | 2.4899734 | 8.69808762 | 0.00563923 | 0.04404723 |
| *ENSGALG00000030754* | 2.48638801 | 1.95039426 | 0.00282298 | 0.0287529 |
| *ENSGALG00000033362* | 2.48395771 | 2.12721422 | 0.00341116 | 0.03249677 |
| *PURA* | 2.48337321 | 2.10232628 | 0.00064348 | 0.01158176 |
| *ENSGALG00000031177* | 2.47681407 | 3.55090252 | 6.74E-05 | 0.00267142 |
| *GGCT* | 2.47320643 | 2.16909671 | 0.00140032 | 0.01810815 |

| | | | | |
|---|---|---|---|---|
| *ENSGALG00000042141* | 2.47258572 | 5.61946353 | 7.01E-07 | 9.63E-05 |
| *SCG5* | 2.46976014 | 5.02558613 | 1.91E-08 | 5.88E-06 |
| *BNIP2* | 2.46704341 | 3.57081854 | 0.00018969 | 0.00531913 |
| *HPCAL1* | 2.46144126 | 6.92807655 | 1.62E-05 | 0.00100747 |
| *ENSGALG00000040676* | 2.46036299 | 4.55682516 | 0.00489803 | 0.04042735 |
| *MAP3K1* | 2.45443778 | 6.42499474 | 4.85E-06 | 0.00041874 |
| *ZFP704* | 2.44662189 | 4.86260935 | 1.71E-05 | 0.00105362 |
| *FRRS1L* | 2.4394141 | 2.71966371 | 0.00650472 | 0.0485132 |
| *NPC2* | 2.43839747 | 6.20754804 | 9.92E-09 | 3.63E-06 |
| *GFPT2* | 2.43691048 | 5.67130363 | 0.00067137 | 0.01188422 |
| *SSBP2* | 2.43559111 | 6.45348546 | 1.08E-06 | 0.00013266 |
| *SLC15A2* | 2.42442556 | 3.0706432 | 0.00599957 | 0.04592829 |
| *ENSGALG00000038056* | 2.42257689 | 3.91175005 | 0.00112588 | 0.01623633 |
| *BFAR* | 2.41073569 | 2.5796863 | 0.00106956 | 0.0157124 |
| *APPL2* | 2.41023316 | 3.93116809 | 1.00E-05 | 0.00068687 |
| *TRPC3* | 2.39658698 | 3.3369822 | 0.00484411 | 0.04032756 |
| *ENSGALG00000031991* | 2.39621129 | 2.88790311 | 0.00101214 | 0.01520032 |
| *RAPH1* | 2.39114973 | 4.57539317 | 0.00057496 | 0.01084528 |
| *ENSGALG00000019489* | 2.38727882 | 3.54372409 | 0.00036753 | 0.00808462 |
| *SLC38A2* | 2.38603936 | 8.90214952 | 7.80E-06 | 0.00056733 |
| *RYK* | 2.38553437 | 4.52646215 | 0.00118775 | 0.01674968 |
| *KDM4B* | 2.37681164 | 4.7918369 | 2.94E-06 | 0.00028458 |
| *KIF1A* | 2.3742582 | 2.61019734 | 0.00605749 | 0.04622185 |
| *DOK3* | 2.37033072 | 4.11506335 | 0.00027944 | 0.00664403 |
| *ZFP385B* | 2.35632416 | 4.76081677 | 0.00400804 | 0.03581144 |
| *ARSB* | 2.3553672 | 4.03573368 | 0.00311578 | 0.0304834 |
| *ZFP488* | 2.35478662 | 2.60976706 | 0.00537204 | 0.04281589 |
| *ENSGALG00000033377* | 2.35356923 | 3.87307736 | 6.33E-05 | 0.00256096 |
| *1500015O10RIK* | 2.35339136 | 8.94101294 | 1.64E-05 | 0.00101237 |
| *ENSGALG00000015219* | 2.3518998 | 1.83004672 | 0.00413598 | 0.03638788 |
| *SALL4* | 2.35136105 | 3.30736327 | 0.00126796 | 0.0174076 |
| *LAMP2* | 2.35055545 | 3.29751348 | 0.00402409 | 0.03584937 |
| *AAED1* | 2.34989772 | 4.02847622 | 0.00097844 | 0.01495259 |
| *FAS* | 2.34790391 | 3.7675717 | 0.00146985 | 0.01866841 |
| *SLC38A3* | 2.34752616 | 3.96905199 | 0.0009683 | 0.01491194 |
| *ENSGALG00000010209* | 2.34664583 | 2.87965943 | 0.00625293 | 0.04733421 |
| *ENSGALG00000019921* | 2.34055236 | 3.9419614 | 2.93E-05 | 0.0015387 |
| *AGTRAP* | 2.33458458 | 4.45953311 | 9.06E-06 | 0.00063783 |
| *ENSGALG00000030712* | 2.33058957 | 10.1498463 | 0.00118241 | 0.01674267 |
| *ENSGALG00000023419* | 2.3219651 | 4.75084462 | 2.34E-06 | 0.00024143 |

| | | | | |
|---|---|---|---|---|
| *ENSGALG00000010927* | 2.31921359 | 7.32942704 | 0.00265437 | 0.02771319 |
| *ANK* | 2.31841164 | 6.07394907 | 3.28E-05 | 0.00167425 |
| *ENSGALG00000032933* | 2.3141784 | 4.4510238 | 4.33E-05 | 0.00197729 |
| *ENSGALG00000029681* | 2.30744572 | 4.32028987 | 3.95E-05 | 0.0018677 |
| *ENSGALG00000009372* | 2.30497189 | 2.067593 | 0.00286605 | 0.02906522 |
| *ENSGALG00000014873* | 2.30133974 | 8.44723182 | 0.00256306 | 0.02712016 |
| *TMEM26* | 2.2973494 | 6.09557342 | 4.24E-08 | 1.16E-05 |
| *PAPD4* | 2.29199752 | 3.72016329 | 0.0029591 | 0.02961982 |
| *TIMP3* | 2.28578762 | 6.90683782 | 0.00021606 | 0.00571788 |
| *FMOD* | 2.28402188 | 4.23847709 | 0.00365864 | 0.03391477 |
| *UHRF2* | 2.27420546 | 5.36516465 | 0.00081785 | 0.01339128 |
| *ENSGALG00000005652* | 2.27410629 | 5.77791568 | 4.82E-06 | 0.00041874 |
| *ENSGALG00000021611* | 2.27408699 | 3.06541594 | 0.00047291 | 0.00964014 |
| *ENSGALG00000004254* | 2.26985987 | 3.6640816 | 6.76E-05 | 0.00267142 |
| *CDK17* | 2.26736876 | 5.25015126 | 1.64E-05 | 0.00101237 |
| *METTL15* | 2.25665024 | 2.57059901 | 0.00262976 | 0.02755796 |
| *IL13RA2* | 2.25276654 | 3.01639955 | 0.00287083 | 0.02908157 |
| *MSRA* | 2.2515316 | 4.24695138 | 1.49E-06 | 0.00017453 |
| *ICE1* | 2.25143944 | 5.47273594 | 0.00025446 | 0.00634013 |
| *PTPRF* | 2.24660347 | 6.12706397 | 5.26E-06 | 0.00044004 |
| *ENSGALG00000042642* | 2.24491142 | 3.46559813 | 0.00305305 | 0.03007763 |
| *HOPX* | 2.24028228 | 2.71629509 | 0.00164972 | 0.02016082 |
| *KANK1* | 2.22906374 | 7.4346202 | 2.39E-08 | 7.04E-06 |
| *SUMF1* | 2.22762238 | 3.54614478 | 0.00012306 | 0.00395607 |
| *TNKS2* | 2.2273885 | 5.09724563 | 0.0001255 | 0.00399877 |
| *EME2* | 2.22016775 | 3.89163789 | 0.00096018 | 0.0148293 |
| *ENSGALG00000004661* | 2.21533445 | 6.79366934 | 2.33E-06 | 0.00024143 |
| *PCMTD1* | 2.21351722 | 6.36572123 | 3.15E-05 | 0.00162434 |
| *ENSGALG00000039754* | 2.21059447 | 1.84427805 | 0.00229069 | 0.02516025 |
| *POLH* | 2.20889258 | 2.96840971 | 0.00246654 | 0.02635559 |
| *ENSGALG00000032816* | 2.20631288 | 3.07630348 | 0.00569968 | 0.04429408 |
| *SLC2A1* | 2.20477667 | 5.61061589 | 1.30E-05 | 0.00085059 |
| *ARSK* | 2.20330959 | 4.03791647 | 5.48E-05 | 0.0022991 |
| *ENSGALG00000039558* | 2.1941097 | 2.07020006 | 0.00447326 | 0.03838441 |
| *4930453N24RIK* | 2.19331322 | 5.05838252 | 6.68E-08 | 1.69E-05 |
| *ENSGALG00000042779* | 2.19019716 | 2.91060736 | 0.0006689 | 0.0118732 |
| *USP24* | 2.18832023 | 6.2714334 | 1.57E-06 | 0.00017959 |
| *ARHGAP17* | 2.18569297 | 5.4832733 | 0.00131444 | 0.01768443 |
| *N4BP2L1* | 2.18510044 | 4.61017797 | 0.0008025 | 0.01318578 |
| *ENSGALG00000035717* | 2.18300139 | 5.19547857 | 0.00077724 | 0.01293785 |

| | | | | |
|---|---|---|---|---|
| *DLX5* | 2.18261713 | 6.49467693 | 0.00050748 | 0.01004083 |
| *ENSGALG00000045137* | 2.17765705 | 3.87814824 | 0.00025735 | 0.00636139 |
| *FGFRL1* | 2.17522935 | 7.27432837 | 7.16E-06 | 0.00053283 |
| *CAR9* | 2.17387617 | 4.0050225 | 0.00328945 | 0.03167856 |
| *ENSGALG00000011128* | 2.17227327 | 4.27892235 | 0.00124289 | 0.01718777 |
| *DTX3L* | 2.17213557 | 2.56057321 | 0.00489267 | 0.04042735 |
| *ENSGALG00000012109* | 2.16928444 | 5.1819297 | 6.68E-06 | 0.00051654 |
| *ENSGALG00000044678* | 2.16913325 | 3.11594022 | 0.00048416 | 0.00977085 |
| *ENSGALG00000008021* | 2.16778156 | 5.11018913 | 1.98E-06 | 0.00022107 |
| *SQSTM1* | 2.16646998 | 5.89276084 | 6.36E-06 | 0.00050802 |
| *ARPC5L* | 2.16483704 | 2.69841344 | 0.00100351 | 0.01516739 |
| *KAT2B* | 2.16178519 | 3.55258842 | 0.00141013 | 0.01815261 |
| *WRNIP1* | 2.15805779 | 3.90773859 | 0.00467157 | 0.0393854 |
| *CD9* | 2.15770945 | 4.99663363 | 7.34E-05 | 0.00282352 |
| *BHLHE40* | 2.15663789 | 5.91194624 | 7.16E-05 | 0.00278121 |
| *NFIL3* | 2.15384716 | 4.72897371 | 0.00135434 | 0.01785699 |
| *MLYCD* | 2.15296473 | 2.76162036 | 0.00147399 | 0.01870178 |
| *SERINC3* | 2.15047916 | 5.57112295 | 1.60E-06 | 0.0001807 |
| *ENSGALG00000037144* | 2.14946398 | 3.78714144 | 0.00387933 | 0.03513505 |
| *ENSGALG00000044385* | 2.14398399 | 2.27779053 | 0.00644134 | 0.04823661 |
| *ENSGALG00000036241* | 2.14087432 | 3.9953702 | 6.05E-05 | 0.00248287 |
| *KLHL11* | 2.13448792 | 3.37576637 | 0.0017206 | 0.02076906 |
| *BTBD3* | 2.1324901 | 1.68240285 | 0.00385826 | 0.03505641 |
| *MAN1C1* | 2.13201057 | 2.34098218 | 0.00463129 | 0.03916668 |
| *CYBRD1* | 2.12831338 | 5.01210532 | 0.00027548 | 0.00660714 |
| *ENSGALG00000013489* | 2.12128479 | 4.75908055 | 0.00011823 | 0.00384504 |
| *APLP2* | 2.11857729 | 7.97031252 | 1.84E-08 | 5.79E-06 |
| *CSNK2A2* | 2.11843307 | 3.83002637 | 0.00016832 | 0.00489962 |
| *HELZ* | 2.11387257 | 5.5527997 | 0.00014834 | 0.00451299 |
| *MSH4* | 2.10821739 | 5.28898264 | 0.00256131 | 0.02712016 |
| *RAB4A* | 2.10681214 | 4.91725427 | 6.28E-06 | 0.00050802 |
| *ENSGALG00000044130* | 2.10570211 | 4.51004643 | 0.00036663 | 0.00808462 |
| *ENSGALG00000023188* | 2.10446849 | 5.42228765 | 2.46E-07 | 4.30E-05 |
| *LRRFIP2* | 2.10443776 | 4.29590526 | 0.00067021 | 0.01188149 |
| *CCNL1* | 2.10148641 | 6.26022402 | 1.92E-05 | 0.00115253 |
| *CPN1* | 2.10126843 | 5.85470555 | 4.39E-05 | 0.00199162 |
| *UBXN10* | 2.0887618 | 2.42688527 | 0.00257535 | 0.02716885 |
| *CLN8* | 2.08803281 | 5.31452907 | 0.0003235 | 0.00739343 |
| *TTC14* | 2.08668913 | 6.10897526 | 5.24E-06 | 0.00044004 |
| *ENSGALG00000028204* | 2.08533743 | 9.80609514 | 0.00023047 | 0.00591477 |

| | | | | |
|---|---|---|---|---|
| *ENSGALG00000006520* | 2.08419651 | 5.2509673 | 0.00240531 | 0.02595112 |
| *CLIC3* | 2.08407551 | 3.19839982 | 0.00410884 | 0.03630717 |
| *NUP155* | 2.08334347 | 5.95875113 | 0.00023133 | 0.00591773 |
| *MAP1LC3A* | 2.08111026 | 6.45860355 | 2.04E-06 | 0.00022227 |
| *CTSO* | 2.07845161 | 4.96750035 | 5.31E-07 | 7.90E-05 |
| *ANKDD1A* | 2.07615631 | 1.83527107 | 0.00539456 | 0.04292286 |
| *FOXP1* | 2.0731124 | 3.6802649 | 0.00078304 | 0.01298798 |
| *VTI1A* | 2.07254676 | 5.92564998 | 3.93E-05 | 0.00186439 |
| *FRRS1* | 2.0707307 | 4.11940995 | 0.00513752 | 0.04160185 |
| *ENSGALG00000006805* | 2.068572 | 5.44049633 | 0.0009193 | 0.01438639 |
| *AFMID* | 2.06727892 | 3.55233123 | 0.001778 | 0.02122644 |
| *SUMF2* | 2.0658994 | 3.93134789 | 0.00023176 | 0.00591773 |
| *AMDHD1* | 2.06575054 | 2.78670898 | 0.0037089 | 0.03416006 |
| *FGF2* | 2.06251983 | 2.85642247 | 0.00049164 | 0.00988049 |
| *ENSGALG00000045764* | 2.0609511 | 2.83733145 | 0.00437922 | 0.03783714 |
| *RGS2* | 2.05793058 | 5.47237111 | 4.92E-05 | 0.00211354 |
| *ENSGALG00000044566* | 2.0574873 | 2.46436743 | 0.00362406 | 0.03377339 |
| *ENSGALG00000035496* | 2.0500002 | 6.21729883 | 6.34E-05 | 0.00256096 |
| *BBS10* | 2.04503273 | 2.61944908 | 0.00644428 | 0.04823661 |
| *ENSGALG00000008282* | 2.03394155 | 5.04172625 | 2.48E-06 | 0.00025234 |
| *ENSGALG00000023950* | 2.03149173 | 5.05756914 | 1.03E-06 | 0.00012903 |
| *PIEZO1* | 2.03006115 | 3.59073587 | 0.00214022 | 0.02399184 |
| *ENSGALG00000045050* | 2.02262894 | 3.33289004 | 0.00293364 | 0.02947598 |
| *NOMO1* | 2.02080947 | 5.35027234 | 5.62E-06 | 0.00046506 |
| *ACSS3* | 2.00815966 | 5.61748513 | 0.00023753 | 0.00603299 |
| *ENSGALG00000009145* | 2.0066756 | 3.58812343 | 0.00196931 | 0.0226503 |

**Supplementary table S10.** Upregulated genes in limb MAT compared to head MAT

| Gene name | logFC | logCPM | PValue | p.adj |
|---|---|---|---|---|
| *NOL4* | 8.04210298 | 0.71573753 | 0.00013279 | 0.0041585 |
| *ENSGALG00000020139* | 8.03005413 | 0.68317013 | 0.00043045 | 0.00903506 |
| *ENSGALG00000039187* | 7.93781219 | 0.04213116 | 0.00034849 | 0.00781313 |
| *ENSGALG00000011633* | 7.87311144 | 0.20885376 | 0.00316471 | 0.03079174 |
| *ENSGALG00000045947* | 7.8297874 | 1.05694017 | 0.0013296 | 0.01774785 |
| *ENSGALG00000010148* | 7.7973831 | -0.2841732 | 0.00098439 | 0.01500919 |
| *PTPN20* | 7.79089191 | 0.60665736 | 0.00070888 | 0.01218529 |
| *ENSGALG00000035553* | 7.77502595 | -0.2519348 | 0.00034214 | 0.00771962 |
| *SLC13A1* | 7.66796766 | 1.025397 | 1.87E-05 | 0.00113273 |
| *ENSGALG00000039859* | 7.60402894 | -0.0845603 | 0.00222995 | 0.02464612 |
| *ENSGALG00000043188* | 7.57546645 | 0.00409869 | 0.00134973 | 0.01785699 |
| *IL18RAP* | 7.56094977 | 0.36411696 | 0.00051864 | 0.01017539 |
| *ENSGALG00000015364* | 7.54428274 | -0.2715974 | 0.00015329 | 0.00462386 |
| *ENSGALG00000035914* | 7.51596095 | -0.0236155 | 0.00267499 | 0.02782583 |
| *ENSGALG00000015143* | 7.49246369 | 0.68639783 | 9.65E-05 | 0.0034052 |
| *ENSGALG00000039702* | 7.47029013 | 1.92676663 | 0.00017438 | 0.00505085 |
| *ENSGALG00000016667* | 7.42293558 | -0.2113919 | 0.00195726 | 0.02262885 |
| *PAPOLG* | 7.39898509 | 0.3754565 | 0.00122513 | 0.01707578 |
| *GCNT2* | 7.31775773 | -0.3084309 | 0.00049722 | 0.00995234 |
| *ENSGALG00000036204* | 7.18658226 | -0.5719246 | 0.00411411 | 0.03630833 |
| *CHRNA9* | 7.12259003 | 0.38509068 | 0.00025163 | 0.00631158 |
| *TMIE* | 7.07863233 | -0.5864971 | 0.0002292 | 0.00590029 |
| *ENSGALG00000038062* | 7.06669745 | 0.37684864 | 6.37E-05 | 0.00256714 |
| *ENSGALG00000035857* | 7.06337872 | 0.20799481 | 0.0048953 | 0.04042735 |
| *ENSGALG00000040400* | 7.06177176 | 2.84869166 | 6.31E-07 | 8.92E-05 |
| *TMPRSS15* | 6.9416578 | 1.32429784 | 0.00030037 | 0.00693205 |
| *SPNS2* | 6.88145944 | -0.1346839 | 0.00070209 | 0.01212987 |
| *ENSGALG00000007727* | 6.83915039 | 0.00604238 | 0.00300565 | 0.0298602 |
| *ENSGALG00000036131* | 6.80514823 | 0.34511638 | 8.86E-05 | 0.00321562 |
| *ENSGALG00000044352* | 6.80184178 | 1.20488578 | 8.76E-10 | 6.52E-07 |
| *ENSGALG00000017016* | 6.79698364 | -0.0716232 | 0.00062907 | 0.01140958 |
| *ENSGALG00000039352* | 6.78541236 | 0.7435848 | 0.0019411 | 0.02250877 |
| *ENSGALG00000043923* | 6.7711663 | -0.5644366 | 0.00566211 | 0.04410898 |
| *ENSGALG00000031869* | 6.75066015 | 1.20369427 | 0.00148346 | 0.01878831 |
| *ENSGALG00000043260* | 6.74132301 | 1.52713784 | 2.55E-06 | 0.00025685 |
| *ENSGALG00000033638* | 6.73459448 | 0.97227838 | 0.00012482 | 0.00398607 |
| *ENSGALG00000035916* | 6.70894796 | 1.27390183 | 7.83E-05 | 0.00296073 |
| *ABCA13* | 6.6917055 | 1.40509619 | 0.00203637 | 0.0231766 |

| | | | | |
|---|---|---|---|---|
| *ENSGALG00000045351* | 6.67830716 | -0.1201429 | 0.00124606 | 0.01721491 |
| *ENSGALG00000045563* | 6.62160811 | -0.3564623 | 0.00538066 | 0.04283631 |
| *ENSGALG00000044552* | 6.62081555 | -0.7136127 | 0.00169898 | 0.02064897 |
| *ENSGALG00000010222* | 6.58832697 | 0.20098001 | 0.00050191 | 0.01000354 |
| *ENSGALG00000014762* | 6.57929627 | -0.4997263 | 0.0013645 | 0.01786165 |
| *ENSGALG00000015153* | 6.53016037 | -0.041682 | 0.00399158 | 0.03581144 |
| *ENSGALG00000046062* | 6.50732639 | 0.22052363 | 0.00186672 | 0.02188325 |
| *ENSGALG00000013571* | 6.50512299 | 0.83931975 | 0.00194004 | 0.02250877 |
| *GRM1* | 6.49559206 | -0.0112934 | 0.00269716 | 0.02795365 |
| *VAT1L* | 6.41088069 | -0.4123279 | 0.00414878 | 0.03645516 |
| *ENSGALG00000037038* | 6.40576557 | 0.46712461 | 0.00353019 | 0.03320582 |
| *TFEC* | 6.39084827 | 0.89451148 | 0.00041957 | 0.00893924 |
| *ENSGALG00000042629* | 6.36725174 | 0.05144123 | 0.00071387 | 0.01224134 |
| *TMC2* | 6.36094995 | 0.41077197 | 0.00109678 | 0.01590002 |
| *ENSGALG00000043197* | 6.33340602 | -0.9060151 | 0.00388188 | 0.03513562 |
| *ENSGALG00000014754* | 6.3204464 | 0.02237885 | 0.00659407 | 0.04886753 |
| *ENSGALG00000034144* | 6.32031197 | 0.71660899 | 0.00026195 | 0.00641383 |
| *TNFRSF18* | 6.29355991 | 1.15047262 | 0.00013494 | 0.00419553 |
| *SLC7A11* | 6.27749262 | 0.1001885 | 0.00077267 | 0.01290546 |
| *ENSGALG00000039997* | 6.19437097 | -0.306758 | 0.00615173 | 0.0467644 |
| *ENSGALG00000039154* | 6.15978824 | 0.47613855 | 0.00083097 | 0.0135278 |
| *ENSGALG00000038824* | 6.09446585 | 3.03614401 | 0.00071286 | 0.01223883 |
| *ENSGALG00000041003* | 6.08474958 | 1.68474595 | 0.00013993 | 0.00427559 |
| *ENSGALG00000039738* | 6.0798747 | 1.9822074 | 0.00057055 | 0.01080527 |
| *ENSGALG00000037227* | 6.07612268 | 0.04921017 | 0.0013734 | 0.01792384 |
| *ENSGALG00000031817* | 6.02813943 | 1.60058944 | 1.06E-07 | 2.35E-05 |
| *ENSGALG00000046351* | 6.02272375 | -0.1961304 | 0.00089806 | 0.01419091 |
| *ENSGALG00000044510* | 6.02061733 | 0.50564086 | 0.00457105 | 0.03895501 |
| *RNF17* | 5.99671565 | 1.86936799 | 0.00044697 | 0.00921822 |
| *ENSGALG00000045531* | 5.97077373 | 1.16357197 | 2.71E-07 | 4.63E-05 |
| *RALYL* | 5.95729505 | 1.69768731 | 9.60E-06 | 0.00066579 |
| *ENSGALG00000039555* | 5.9559243 | 0.18392991 | 2.92E-06 | 0.00028458 |
| *ENSGALG00000042942* | 5.90635872 | 1.89613846 | 0.00296334 | 0.02961982 |
| *ENSGALG00000032136* | 5.90382704 | 2.482832 | 0.00385124 | 0.03501514 |
| *ENSGALG00000043839* | 5.89435483 | -0.0331619 | 0.00104168 | 0.01546339 |
| *ENSGALG00000043637* | 5.88752362 | -0.3820343 | 0.00258719 | 0.0272531 |
| *ENSGALG00000006469* | 5.88407618 | -0.6629836 | 0.003328 | 0.03191943 |
| *MSGN1* | 5.87245947 | -0.5128269 | 0.00403706 | 0.03589714 |
| *ENSGALG00000028357* | 5.85397939 | 1.56628538 | 0.00104571 | 0.01547415 |
| *DCSTAMP* | 5.84411756 | 0.68998184 | 0.00119761 | 0.0168129 |

| | | | | |
|---|---|---|---|---|
| *ENSGALG00000026501* | 5.8439518 | 5.85241779 | 0.00021555 | 0.00571788 |
| *ENSGALG00000043266* | 5.81952487 | 5.12089901 | 0.00165765 | 0.02023366 |
| *ENSGALG00000043123* | 5.81166811 | 0.53094829 | 0.00102585 | 0.01530447 |
| *ENSGALG00000046409* | 5.77794824 | -0.4445016 | 0.00316933 | 0.03081547 |
| *COL4A3* | 5.73214425 | 1.72017922 | 0.00083838 | 0.01360156 |
| *ENSGALG00000033333* | 5.72999734 | 0.47009276 | 0.00187579 | 0.02196758 |
| *AA986860* | 5.72809012 | -0.2190872 | 7.11E-05 | 0.00277282 |
| *ENSGALG00000006983* | 5.71352961 | 0.25663522 | 0.00403549 | 0.03589714 |
| *ENSGALG00000046297* | 5.68897988 | -0.0251753 | 0.00328875 | 0.03167856 |
| *ENSGALG00000041452* | 5.68093615 | -0.7200402 | 0.00511746 | 0.04151189 |
| *ENSGALG00000043523* | 5.66426416 | 1.47485496 | 0.00393898 | 0.03554314 |
| *ENSGALG00000020002* | 5.64275765 | 0.16538426 | 0.00027715 | 0.00662314 |
| *ENSGALG00000034581* | 5.63351377 | 4.68471071 | 0.00078975 | 0.01305214 |
| *ENSGALG00000041612* | 5.62188997 | 0.11641078 | 0.00639183 | 0.0479964 |
| *ENSGALG00000045260* | 5.62143622 | 0.47901866 | 0.00570306 | 0.04429408 |
| *OTOGL* | 5.61652272 | 2.54751598 | 0.0007678 | 0.0128698 |
| *PTPRN2* | 5.60061215 | 1.22541812 | 0.00044099 | 0.00918813 |
| *ENSGALG00000029913* | 5.59471584 | 4.3542388 | 0.00180351 | 0.02140462 |
| *ENSGALG00000015366* | 5.58980491 | 0.10765982 | 0.00280566 | 0.02874121 |
| *ATP7B* | 5.57644491 | 0.15274075 | 0.00261572 | 0.02747184 |
| *ENSGALG00000045796* | 5.54489884 | 0.16822446 | 0.00093417 | 0.01452699 |
| *ANXA10* | 5.52819481 | -0.4773533 | 0.00148155 | 0.01878095 |
| *ENSGALG00000033664* | 5.50623514 | 1.42206095 | 0.0002259 | 0.00584244 |
| *CNTN5* | 5.50252456 | 1.0333454 | 0.00580138 | 0.04477474 |
| *SLC6A11* | 5.49181772 | 1.61297167 | 0.00097661 | 0.01495259 |
| *BARX1* | 5.48853555 | 4.07244001 | 5.31E-15 | 1.25E-11 |
| *LMOD1* | 5.48464161 | 1.01375397 | 4.84E-07 | 7.37E-05 |
| *ASZ1* | 5.48452393 | 1.47553076 | 0.00069745 | 0.01212138 |
| *ENSGALG00000026810* | 5.46566893 | -0.1898113 | 0.00405227 | 0.03596457 |
| *ENSGALG00000028652* | 5.46377343 | -0.5471655 | 0.00010778 | 0.00363022 |
| *SLC10A2* | 5.44513959 | 0.5488305 | 0.00077142 | 0.01289978 |
| *ENSGALG00000039271* | 5.4426334 | 1.92541617 | 0.00678758 | 0.04990844 |
| *MYT1L* | 5.43320133 | 1.40679579 | 0.0005079 | 0.01004083 |
| *ENSGALG00000037813* | 5.4314617 | -0.0515076 | 0.00268361 | 0.02787446 |
| *ENSGALG00000011022* | 5.42670233 | 0.03216459 | 0.00351552 | 0.03311193 |
| *ENSGALG00000040377* | 5.41314182 | 1.29692127 | 0.00360226 | 0.0336439 |
| *ANKRD34B* | 5.38935452 | -0.2046497 | 6.82E-05 | 0.00268145 |
| *ENSGALG00000042713* | 5.38686275 | 2.71828566 | 0.00160842 | 0.01982081 |
| *ENSGALG00000043172* | 5.38607562 | 0.36163719 | 0.00059298 | 0.01107917 |
| *CDH12* | 5.37264324 | 1.3888665 | 0.00125708 | 0.01729111 |

| | | | | |
|---|---|---|---|---|
| *ENSGALG00000035692* | 5.36230004 | 0.80782541 | 0.00481949 | 0.04020128 |
| *SLCO1C1* | 5.34302388 | -0.2760154 | 0.00249484 | 0.0265972 |
| *DNAH5* | 5.31430478 | 3.34277131 | 0.00116449 | 0.01659357 |
| *ENSGALG00000043225* | 5.30283126 | 1.00761253 | 0.00541735 | 0.04295924 |
| *ENSGALG00000039175* | 5.29147271 | 1.4110244 | 0.00346987 | 0.03281304 |
| *ENSGALG00000041735* | 5.28863194 | 2.19869087 | 0.00548161 | 0.04325062 |
| *MLC1* | 5.28667663 | 1.33344135 | 0.00016066 | 0.00475494 |
| *ENSGALG00000043438* | 5.28393872 | 1.70289648 | 0.00358688 | 0.03356054 |
| *ENSGALG00000031367* | 5.28074226 | 0.23372041 | 7.28E-06 | 0.00053903 |
| *STEAP4* | 5.2770893 | 1.71763954 | 0.00022049 | 0.00576924 |
| *ENSGALG00000030017* | 5.25976998 | 1.30299726 | 0.00421154 | 0.03684915 |
| *ENSGALG00000010901* | 5.25488947 | 2.01898757 | 0.00019025 | 0.00531913 |
| *ENSGALG00000005078* | 5.24643648 | 2.8213543 | 3.17E-07 | 5.33E-05 |
| *VIPR2* | 5.24601978 | 0.61465349 | 2.21E-05 | 0.00126669 |
| *ENSGALG00000016183* | 5.23439798 | 1.21447996 | 0.00051094 | 0.01005715 |
| *TTC29* | 5.23435659 | 1.29927188 | 0.00010097 | 0.00347123 |
| *GADL1* | 5.22956299 | 1.04153779 | 0.00276231 | 0.02842067 |
| *ENSGALG00000038950* | 5.22951483 | 2.05116059 | 0.00081557 | 0.0133694 |
| *SLC5A12* | 5.21957803 | 1.3655923 | 0.0001867 | 0.00530904 |
| *PTPRQ* | 5.20282898 | 2.18738562 | 0.0006722 | 0.01188422 |
| *ANO2* | 5.19023147 | 1.2821498 | 0.00149463 | 0.01887901 |
| *ENPEP* | 5.1695879 | 2.75549496 | 0.00060418 | 0.01115837 |
| *ENSGALG00000040338* | 5.16652688 | 1.56163541 | 3.50E-07 | 5.76E-05 |
| *ENSGALG00000030861* | 5.15263273 | 0.63836732 | 0.00305038 | 0.03007763 |
| *ENSGALG00000024469* | 5.14796558 | -0.7650943 | 0.00148705 | 0.01881691 |
| *GHRHR* | 5.1475239 | -0.0401863 | 0.00400291 | 0.03581144 |
| *GLRA3* | 5.14606635 | 0.37112322 | 0.00280427 | 0.02874121 |
| *SLC7A10* | 5.13301556 | -0.2744789 | 0.0036498 | 0.03388035 |
| *TRIM66* | 5.11669641 | 1.59430413 | 0.00134263 | 0.01785699 |
| *KBTBD12* | 5.10306414 | -0.0762585 | 0.00151309 | 0.01902724 |
| *ENSGALG00000043348* | 5.09312059 | 1.54553045 | 0.00313132 | 0.03057197 |
| *ENSGALG00000006072* | 5.08399703 | 1.24728928 | 0.00668325 | 0.04934648 |
| *PDC* | 5.07820152 | 0.79037129 | 0.00061101 | 0.01122791 |
| *GLP2R* | 5.07514791 | 0.89415446 | 0.00546435 | 0.04320428 |
| *ENSGALG00000041603* | 5.07293136 | 8.51500059 | 4.54E-15 | 1.25E-11 |
| *ENSGALG00000043705* | 5.05117412 | 1.59488949 | 0.0052671 | 0.04229436 |
| *ENSGALG00000002496* | 5.04390934 | -0.4127319 | 0.00578798 | 0.04469568 |
| *GZMA* | 5.02542973 | -0.4266043 | 0.00295879 | 0.02961982 |
| *SLC2A9* | 5.01455069 | 0.5700474 | 0.0026693 | 0.02782002 |
| *STMN2* | 4.99480175 | 3.61530751 | 0.00010345 | 0.00352636 |

| | | | | |
|---|---|---|---|---|
| *ENSGALG00000011322* | 4.94964951 | 1.32122358 | 0.00116473 | 0.01659357 |
| *ENSGALG00000020084* | 4.92583966 | 0.05050974 | 0.00240672 | 0.02595112 |
| *ENSGALG00000038596* | 4.92545115 | -0.0549362 | 0.00652778 | 0.0486045 |
| *ENSGALG00000015336* | 4.92084947 | -0.6709263 | 0.00424909 | 0.03702303 |
| *ENSGALG00000026297* | 4.91671592 | 0.23809581 | 0.00442735 | 0.03814474 |
| *ENSGALG00000042108* | 4.91501571 | -0.4825189 | 0.0012837 | 0.01752937 |
| *ENSGALG00000032541* | 4.91304998 | 0.71142672 | 0.00054437 | 0.01050644 |
| *CPLX1* | 4.9007237 | -0.2880977 | 0.00140696 | 0.01814421 |
| *ENSGALG00000031293* | 4.87492971 | 0.86786437 | 0.0053491 | 0.04275349 |
| *FAM19A2* | 4.86346781 | -0.5310119 | 0.0010239 | 0.0152958 |
| *ENSGALG00000039853* | 4.86334179 | 0.37651065 | 8.70E-05 | 0.00318244 |
| *ENSGALG00000031737* | 4.86196312 | 2.37857816 | 0.00310398 | 0.03045259 |
| *ENSGALG00000013268* | 4.85606003 | 3.29189206 | 0.00067295 | 0.01188422 |
| *ENSGALG00000040505* | 4.84823439 | 0.92958874 | 0.00042513 | 0.00894978 |
| *ENSGALG00000038366* | 4.83759729 | -0.2580142 | 0.00064106 | 0.01156771 |
| *ENSGALG00000044307* | 4.82898615 | 0.54617536 | 0.00135246 | 0.01785699 |
| *GRM8* | 4.82185357 | 0.22338995 | 0.00513185 | 0.04158092 |
| *ENSGALG00000033837* | 4.82086299 | 3.95241164 | 0.0065942 | 0.04886753 |
| *SLC4A10* | 4.81799783 | 2.2279711 | 6.41E-05 | 0.00256938 |
| *ENSGALG00000011080* | 4.8132046 | 0.02611481 | 0.00070603 | 0.01216589 |
| *ENSGALG00000019509* | 4.8076416 | 1.15707067 | 0.00460479 | 0.03907857 |
| *ROS1* | 4.7601527 | 2.56751494 | 0.00010809 | 0.00363226 |
| *ASTN1* | 4.74430247 | 0.62921852 | 6.61E-06 | 0.00051485 |
| *ENSGALG00000043071* | 4.73153346 | 7.64129231 | 3.23E-07 | 5.38E-05 |
| *ENSGALG00000010867* | 4.71811602 | 0.0114731 | 0.00127168 | 0.01743267 |
| *KLHL1* | 4.71199813 | 1.01559601 | 0.00081967 | 0.01340563 |
| *ENSGALG00000019147* | 4.70305277 | 1.45241293 | 0.00094885 | 0.01468639 |
| *ENSGALG00000044263* | 4.68753519 | 1.25011075 | 0.00053314 | 0.0103604 |
| *MLIP* | 4.68585654 | 1.31498697 | 0.0029991 | 0.02983701 |
| *ENSGALG00000004116* | 4.68183593 | 0.79922467 | 0.00483883 | 0.040315 |
| *ENSGALG00000039973* | 4.66544958 | 1.18290466 | 2.23E-06 | 0.00023689 |
| *ENSGALG00000043500* | 4.66533498 | 1.26696084 | 0.00637997 | 0.04793276 |
| *ENSGALG00000045956* | 4.64570468 | 2.93277354 | 4.37E-09 | 1.82E-06 |
| *UMODL1* | 4.64358652 | 0.2058066 | 0.0039319 | 0.03552014 |
| *ENSGALG00000015147* | 4.62106394 | 1.72558659 | 0.00061421 | 0.01124296 |
| *STK31* | 4.61923286 | 0.74122008 | 0.002815 | 0.0287529 |
| *FGF23* | 4.6174137 | 0.89552239 | 0.00012326 | 0.00395607 |
| *ENSGALG00000013083* | 4.60368884 | 1.27946494 | 0.00065685 | 0.0117477 |
| *ENSGALG00000028304* | 4.5923591 | 1.17866754 | 0.00053744 | 0.01040097 |
| *DOCK2* | 4.57990653 | 2.77159468 | 0.00027182 | 0.0065509 |

| | | | | |
|---|---|---|---|---|
| *ZZEF1* | 4.57555074 | 2.19653368 | 0.0003191 | 0.00731645 |
| *ENSGALG00000042897* | 4.56394631 | 0.26260207 | 0.00273944 | 0.028247 |
| *ERICH3* | 4.56198786 | 1.3710751 | 0.00022192 | 0.00579213 |
| *ENSGALG00000002428* | 4.54751159 | 0.82807013 | 0.00020021 | 0.00548921 |
| *ENSGALG00000042664* | 4.53075476 | 3.33836181 | 5.07E-06 | 0.00043199 |
| *BMX* | 4.53068855 | 0.48366319 | 0.00338821 | 0.03234344 |
| *ENSGALG00000008656* | 4.51389769 | 0.19391742 | 0.00078782 | 0.0130355 |
| *PLPPR4* | 4.50597951 | 0.22554529 | 0.00400819 | 0.03581144 |
| *ENSGALG00000009511* | 4.48955075 | 1.69851577 | 0.00635781 | 0.04786797 |
| *LOXHD1* | 4.45828921 | 3.38339096 | 6.31E-07 | 8.92E-05 |
| *ENSGALG00000012010* | 4.44473519 | -0.4764448 | 0.00074173 | 0.01255172 |
| *PAX6* | 4.43625508 | 0.54017721 | 0.00156141 | 0.01939353 |
| *CERKL* | 4.42997255 | 1.46049936 | 0.00017786 | 0.00510389 |
| *GSC* | 4.42476402 | 4.47316723 | 2.74E-09 | 1.35E-06 |
| *DHRS7C* | 4.42091453 | 0.97769543 | 0.00010057 | 0.00347123 |
| *KCNH7* | 4.3869436 | 0.51558305 | 0.00640159 | 0.04804416 |
| *OTC* | 4.38414042 | -0.3455415 | 0.00487445 | 0.04042735 |
| *ENSGALG00000030326* | 4.38058054 | -0.572795 | 0.00656964 | 0.04875903 |
| *ANKK1* | 4.33436508 | -0.4740786 | 0.0030313 | 0.02995457 |
| *ENSGALG00000032853* | 4.33280958 | 0.11921165 | 0.00043437 | 0.0090816 |
| *ENSGALG00000029504* | 4.33056009 | -0.3194006 | 0.00641389 | 0.04808546 |
| *1700029J07RIK* | 4.32958802 | 0.22492911 | 0.00488788 | 0.04042735 |
| *ENSGALG00000040306* | 4.32272335 | 9.19874917 | 4.01E-09 | 1.72E-06 |
| *WDFY4* | 4.32223659 | 2.95107411 | 2.03E-05 | 0.00118552 |
| *ENSGALG00000031836* | 4.31986335 | -0.0871403 | 0.00498066 | 0.04084125 |
| *EPSTI1* | 4.31779599 | 1.18108551 | 0.00025766 | 0.00636139 |
| *ENSGALG00000031268* | 4.31335064 | -0.4264091 | 0.0053272 | 0.04265075 |
| *ENSGALG00000034877* | 4.28542119 | -0.2436807 | 0.0025737 | 0.02716885 |
| *CSMD3* | 4.28526636 | 3.27280137 | 0.00122912 | 0.01709769 |
| *CNGB3* | 4.24903714 | -0.1248377 | 0.00612879 | 0.04661507 |
| *ENSGALG00000040029* | 4.2349094 | 1.56781811 | 6.14E-05 | 0.00250215 |
| *SGIP1* | 4.21861396 | -0.3231111 | 0.00446679 | 0.03838441 |
| *GABRA2* | 4.21455423 | 0.55563368 | 0.00411966 | 0.0363347 |
| *ENSGALG00000044973* | 4.18707801 | 1.06151759 | 6.81E-06 | 0.00052081 |
| *ENSGALG00000046611* | 4.1815405 | 0.75973888 | 3.77E-05 | 0.00182684 |
| *ENSGALG00000042136* | 4.17399795 | 1.7013521 | 0.00130796 | 0.01768443 |
| *ENSGALG00000019325* | 4.16082087 | 0.83017173 | 0.00026496 | 0.0064517 |
| *ENSGALG00000031659* | 4.1578538 | 0.78633192 | 0.00296271 | 0.02961982 |
| *BRDT* | 4.14555161 | 0.7958966 | 0.00245647 | 0.02635559 |
| *RD3L* | 4.1434843 | 1.92263626 | 0.00033058 | 0.00751891 |

| | | | | |
|---|---|---|---|---|
| *ENSGALG00000038191* | 4.13083488 | -0.1833871 | 0.00484603 | 0.04032756 |
| *SLC26A4* | 4.10116374 | 0.9778772 | 0.00604907 | 0.04618254 |
| *ENSGALG00000036373* | 4.09783329 | -0.1891799 | 0.00028776 | 0.00673194 |
| *OMD* | 4.08984284 | 2.5597334 | 0.00186649 | 0.02188325 |
| *NR1H5* | 4.08918424 | -0.4185517 | 0.00476849 | 0.03991977 |
| *ENSGALG00000037178* | 4.07802413 | 0.51580584 | 0.00376544 | 0.0344468 |
| *ENSGALG00000008434* | 4.07647274 | 0.98568127 | 0.00299208 | 0.02978816 |
| *CALB1* | 4.07623525 | 0.93504123 | 0.00443164 | 0.03815848 |
| *ENSGALG00000014793* | 4.07314311 | 0.54252446 | 0.00540228 | 0.04295436 |
| *ENSGALG00000036544* | 4.06367248 | 1.5819103 | 0.0001383 | 0.00425329 |
| *MSTN* | 4.060749 | 0.29532169 | 0.00294204 | 0.0295394 |
| *ENSGALG00000010943* | 4.05240595 | 2.65540543 | 0.00131081 | 0.01768443 |
| *ENSGALG00000006229* | 4.04048077 | 0.37737378 | 0.00222113 | 0.02460281 |
| *IQCM* | 4.03508858 | 0.90286328 | 0.00647484 | 0.0484141 |
| *TBX1* | 4.03297782 | -0.3188367 | 0.00155072 | 0.01934576 |
| *ENSGALG00000044864* | 4.02634071 | 1.23015684 | 0.0056841 | 0.04420725 |
| *ENSGALG00000029950* | 4.02321837 | 1.45992486 | 6.56E-05 | 0.0026161 |
| *ENSGALG00000042638* | 4.02207279 | 2.29825902 | 0.00210338 | 0.02374564 |
| *GABBR2* | 4.01746999 | 1.80189608 | 0.00173512 | 0.02087308 |
| *SNAP25* | 4.00327076 | 1.86316906 | 0.00050765 | 0.01004083 |
| *ENSGALG00000017168* | 3.99026322 | 0.70409038 | 0.00178716 | 0.02126519 |
| *ENSGALG00000005795* | 3.98614313 | 0.77713896 | 0.00296035 | 0.02961982 |
| *ENSGALG00000004946* | 3.96545379 | 1.83699495 | 0.00344753 | 0.03268914 |
| *ENSGALG00000034843* | 3.96532299 | 2.71239744 | 0.00026461 | 0.0064517 |
| *ENSGALG00000041563* | 3.96360579 | 0.83375242 | 0.0037114 | 0.03416082 |
| *MUC6* | 3.95890823 | 1.62181956 | 0.00476252 | 0.03991428 |
| *ENSGALG00000031246* | 3.95750915 | 0.06983934 | 0.00537085 | 0.04281589 |
| *SERPINA10* | 3.95538126 | 1.00015345 | 0.00092595 | 0.01445845 |
| *ENSGALG00000014252* | 3.94583415 | 0.87853481 | 0.00135382 | 0.01785699 |
| *ENSGALG00000010760* | 3.93932131 | 1.31501103 | 0.00029124 | 0.00678768 |
| *CEP126* | 3.91289171 | 0.957997 | 0.00204821 | 0.02326259 |
| *ENSGALG00000006322* | 3.90776559 | 0.94834294 | 0.0028823 | 0.02914901 |
| *MAFA* | 3.90253444 | 1.5489969 | 4.17E-05 | 0.00191949 |
| *ENSGALG00000033906* | 3.88685261 | -0.2149412 | 0.00377081 | 0.03446101 |
| *PPP4R4* | 3.87715796 | 2.54259213 | 0.00028785 | 0.00673194 |
| *GALNT12* | 3.87502572 | 0.93647475 | 0.00102773 | 0.01530447 |
| *ADCY8* | 3.87167478 | 1.67235029 | 0.00143402 | 0.01837447 |
| *ENSGALG00000032782* | 3.85620485 | -0.3504668 | 0.00218346 | 0.02432237 |
| *ENSGALG00000000625* | 3.8546632 | 3.7569549 | 5.08E-10 | 4.49E-07 |
| *SLC6A15* | 3.84934682 | 1.93275906 | 0.00118462 | 0.01674267 |

| | | | | |
|---|---|---|---|---|
| *PPIP5K1* | 3.84488671 | 0.61916733 | 0.0039795 | 0.03581144 |
| *ENSGALG00000023772* | 3.84273383 | 0.62596752 | 0.0008404 | 0.01360854 |
| *SLC24A4* | 3.82644681 | 1.0125515 | 0.00370207 | 0.0341569 |
| *ENSGALG00000036338* | 3.78733065 | 0.70168248 | 0.00013772 | 0.00424467 |
| *AXDND1* | 3.78700635 | 1.37428487 | 0.00302429 | 0.02994053 |
| *ENSGALG00000029256* | 3.76217567 | 0.89123353 | 0.00154307 | 0.01928422 |
| *INPP5D* | 3.74665528 | 1.07508983 | 2.79E-05 | 0.00148586 |
| *MYOG* | 3.74379496 | 4.13907813 | 1.96E-05 | 0.001159 |
| *ENSGALG00000041729* | 3.73008814 | -0.6364914 | 0.00584288 | 0.04494795 |
| *ENSGALG00000034128* | 3.72886871 | 2.47569237 | 0.00246445 | 0.02635559 |
| *DLX1* | 3.71183528 | 1.29490804 | 0.00019502 | 0.00539288 |
| *ENSGALG00000015599* | 3.68704836 | 2.12846798 | 8.43E-05 | 0.00312374 |
| *ENSGALG00000029432* | 3.67025525 | 0.00422606 | 0.00485647 | 0.04036693 |
| *ENSGALG00000004448* | 3.6685414 | 1.22678882 | 0.00503266 | 0.04105154 |
| *CCDC40* | 3.66078212 | 0.61292278 | 0.00105462 | 0.01557382 |
| *ADGRL4* | 3.65079841 | 1.22793813 | 0.00136484 | 0.01786165 |
| *ENSGALG00000037937* | 3.6503293 | -0.2099885 | 0.00631357 | 0.0476363 |
| *ENSGALG00000039355* | 3.63062712 | 0.3653663 | 0.00472019 | 0.03967708 |
| *ENSGALG00000032534* | 3.61969783 | 2.90815463 | 0.00237 | 0.02571191 |
| *BEND6* | 3.61267254 | 4.28105547 | 1.76E-08 | 5.66E-06 |
| *ENSGALG00000037911* | 3.59493923 | 0.53806581 | 0.00422406 | 0.03688749 |
| *FGF13* | 3.58880123 | 3.64970357 | 9.55E-05 | 0.00338738 |
| *IL1RAPL1* | 3.58246985 | 1.97913435 | 0.00053718 | 0.01040097 |
| *DCDC2A* | 3.581145 | 2.01449331 | 0.00530267 | 0.04247839 |
| *MUSTN1* | 3.5775235 | 4.05195898 | 2.25E-06 | 0.00023689 |
| *ENSGALG00000039354* | 3.56382691 | 1.66343586 | 0.00281987 | 0.0287529 |
| *ENSGALG00000031929* | 3.56285864 | 1.76539867 | 0.00193749 | 0.02250877 |
| *NLRC5* | 3.55227749 | 1.22584906 | 0.00022368 | 0.00580632 |
| *USP53* | 3.54721496 | 2.0853321 | 0.00120449 | 0.0168712 |
| *NELL1* | 3.53658568 | 0.99808178 | 0.00347397 | 0.03282983 |
| *ENSGALG00000039931* | 3.5277239 | 1.20467401 | 0.00102085 | 0.0152663 |
| *ETNPPL* | 3.52190526 | 1.50877121 | 9.23E-06 | 0.00064622 |
| *SNTG2* | 3.51973695 | 1.48763482 | 0.00204886 | 0.02326259 |
| *ENSGALG00000008638* | 3.50930563 | 1.30927303 | 0.00421941 | 0.03686969 |
| *PRSS56* | 3.50304638 | 0.68783945 | 0.00476881 | 0.03991977 |
| *POLN* | 3.50144674 | 2.50444187 | 0.00027612 | 0.00660954 |
| *ENSGALG00000044994* | 3.49689033 | 3.86780111 | 0.00194269 | 0.02250879 |
| *GJD2* | 3.49017977 | 2.36972975 | 0.00265232 | 0.02771319 |
| *ENSGALG00000014242* | 3.47718502 | 0.59747413 | 0.00281282 | 0.0287529 |
| *ENSGALG00000026840* | 3.46970379 | 1.36908962 | 0.00131471 | 0.01768443 |

| | | | | |
|---|---|---|---|---|
| *ENSGALG00000013211* | 3.46859096 | 1.4357159 | 0.00017632 | 0.00508013 |
| *CACNA1B* | 3.44164265 | 1.63369675 | 0.00122698 | 0.01708466 |
| *NWD2* | 3.42457427 | 2.3022801 | 5.50E-05 | 0.00230132 |
| *GPC5* | 3.42139414 | 1.59706089 | 0.0011369 | 0.01632861 |
| *ENPP3* | 3.40583041 | 2.16097271 | 0.00495619 | 0.04074095 |
| *SLC13A4* | 3.40402378 | 0.8407364 | 0.00329191 | 0.0316807 |
| *CPXM2* | 3.39392843 | 0.95811231 | 0.00351924 | 0.03312485 |
| *ENDOU* | 3.38464936 | -0.2613041 | 0.00571223 | 0.04429408 |
| *TLL1* | 3.37969155 | 3.63640231 | 2.22E-05 | 0.00126896 |
| *ENSGALG00000009152* | 3.36580642 | 2.46722437 | 0.00015825 | 0.00470336 |
| *PLEK* | 3.3657094 | 0.90041982 | 0.00298776 | 0.02978707 |
| *ALX1* | 3.36185995 | 4.11860374 | 4.37E-05 | 0.00198849 |
| *ENSGALG00000037845* | 3.36093335 | -0.0078621 | 0.00118329 | 0.01674267 |
| *GAL* | 3.36070625 | 0.54632684 | 0.00626006 | 0.04735888 |
| *TNN* | 3.34915985 | 4.27393415 | 0.00070222 | 0.01212987 |
| *TRHDE* | 3.34033659 | 0.81537657 | 0.00584052 | 0.04494795 |
| *GPM6A* | 3.33776952 | 1.13477229 | 0.00065943 | 0.011779 |
| *ADCY7* | 3.30469281 | 1.92789019 | 0.00211529 | 0.02378774 |
| *FHL5* | 3.2974732 | 3.47912953 | 0.00019259 | 0.00536019 |
| *TRDN* | 3.29641772 | 3.35494861 | 0.00667321 | 0.04931416 |
| *ANO1* | 3.29351071 | 1.39672539 | 0.00556686 | 0.04365538 |
| *ENSGALG00000033925* | 3.29127661 | 1.27165272 | 0.00414334 | 0.03642997 |
| *SPON2* | 3.27674579 | 4.15440369 | 0.00056069 | 0.01067574 |
| *GRID1* | 3.27247916 | 2.48952782 | 1.37E-06 | 0.00016138 |
| *TM6SF1* | 3.26682845 | 3.51473175 | 2.19E-06 | 0.0002342 |
| *SLC6A7* | 3.2547907 | 1.64698427 | 0.00017738 | 0.00510033 |
| *CALB2* | 3.24453775 | 1.92205941 | 0.00062004 | 0.01131824 |
| *ENSGALG00000037077* | 3.24290441 | 1.24746197 | 0.00335668 | 0.03215096 |
| *ENSGALG00000016622* | 3.2408103 | 2.06603323 | 0.00136016 | 0.01786165 |
| *ENSGALG00000028451* | 3.23037767 | 4.05632533 | 2.18E-06 | 0.0002342 |
| *ARL10* | 3.20407442 | 0.5341557 | 0.00331819 | 0.03184693 |
| *ENSGALG00000043541* | 3.1955499 | 3.20359557 | 0.00034569 | 0.00777493 |
| *ENSGALG00000012556* | 3.16158471 | 8.71769721 | 1.73E-12 | 2.72E-09 |
| *ENSGALG00000023601* | 3.1612662 | 0.15817891 | 0.00672112 | 0.04957439 |
| *PTPRB* | 3.1598024 | 2.59812024 | 0.00472342 | 0.03968064 |
| *SYCP1* | 3.15756629 | 1.76908113 | 0.00278572 | 0.02864071 |
| *ANKRD27* | 3.15586395 | 2.20958181 | 0.00139052 | 0.01805831 |
| *IKZF1* | 3.13402455 | 1.73263152 | 0.00076775 | 0.0128698 |
| *BANK1* | 3.13107007 | 1.52087999 | 0.00102675 | 0.01530447 |
| *ENSGALG00000029601* | 3.12960969 | 6.59321839 | 1.98E-07 | 3.68E-05 |

| | | | | |
|---|---|---|---|---|
| *ENSGALG00000045869* | 3.11669529 | 2.14412509 | 0.0007585 | 0.01277441 |
| *CNNM2* | 3.11246719 | 0.54203442 | 0.00393966 | 0.03554314 |
| *SYNC* | 3.10922984 | 2.16118123 | 0.00238509 | 0.02583602 |
| *MYF6* | 3.08523214 | 2.18443303 | 0.00627038 | 0.04736096 |
| *MYO1F* | 3.08417243 | 1.62526572 | 0.00059452 | 0.01108132 |
| *ENSGALG00000030237* | 3.0785701 | 3.1415275 | 0.00373218 | 0.03426292 |
| *ENSGALG00000016791* | 3.07275285 | 3.1932442 | 0.00101207 | 0.01520032 |
| *ENSGALG00000037220* | 3.07061357 | 0.99825183 | 0.00327738 | 0.03162692 |
| *ENSGALG00000000302* | 3.06350472 | 7.41146989 | 0.0025903 | 0.02726559 |
| *SPHKAP* | 3.04507996 | 1.46286347 | 0.00131718 | 0.01768535 |
| *ENSGALG00000008113* | 3.02664633 | 0.6953835 | 0.00079542 | 0.01309988 |
| *LVRN* | 3.02522065 | 2.81748296 | 0.00575257 | 0.04449834 |
| *ENSGALG00000006693* | 3.02292137 | 0.99166008 | 0.00640817 | 0.0480681 |
| *PTPRO* | 3.02084774 | 2.20850643 | 0.00042133 | 0.00894641 |
| *TNNC1* | 3.01687056 | 7.98228882 | 0.0023527 | 0.02558314 |
| *MYOD1* | 3.00969725 | 5.79601303 | 0.00061432 | 0.01124296 |
| *ENSGALG00000030896* | 3.00271636 | 4.49459805 | 2.09E-08 | 6.29E-06 |
| *ENSGALG00000044191* | 2.98545188 | 0.66872158 | 0.00136236 | 0.01786165 |
| *ENSGALG00000042236* | 2.98240364 | 1.64634559 | 5.78E-05 | 0.00239894 |
| *ENSGALG00000032449* | 2.97934512 | 7.59434447 | 3.57E-05 | 0.00177615 |
| *ENSGALG00000044508* | 2.97773038 | -0.2392734 | 0.00310296 | 0.03045259 |
| *ENSGALG00000034671* | 2.97052115 | 1.98466258 | 1.37E-05 | 0.00088534 |
| *BARX2* | 2.96913203 | 4.35319152 | 2.77E-05 | 0.00148567 |
| *ENSGALG00000042543* | 2.96772845 | 2.72235763 | 0.00029885 | 0.00691463 |
| *ADGRD1* | 2.95795314 | 2.59501899 | 0.00541066 | 0.04295436 |
| *ENSGALG00000001191* | 2.95540645 | 2.95682651 | 0.00057432 | 0.01084528 |
| *ENSGALG00000039430* | 2.95411365 | 0.61750328 | 0.00051114 | 0.01005715 |
| *ENSGALG00000006835* | 2.95356731 | 7.8313238 | 0.00290245 | 0.02926657 |
| *MYOZ2* | 2.95047899 | 5.22279688 | 0.0047359 | 0.03971471 |
| *RBP7* | 2.94987513 | 2.62475038 | 0.00030784 | 0.00709284 |
| *ENSGALG00000044239* | 2.94900175 | 17.1663154 | 5.67E-08 | 1.48E-05 |
| *ENSGALG00000043073* | 2.93414959 | 0.28995363 | 0.00477945 | 0.03993789 |
| *ENSGALG00000002113* | 2.92435031 | 1.87797598 | 0.00549118 | 0.04326056 |
| *ENSGALG00000039727* | 2.92071646 | 0.00447368 | 0.00416416 | 0.03653435 |
| *SLCO4A1* | 2.92010315 | 1.34404661 | 0.00034372 | 0.00774304 |
| *DGKI* | 2.91929913 | 2.77831103 | 0.00489323 | 0.04042735 |
| *ADGRG2* | 2.91032814 | 2.29602521 | 0.00267111 | 0.02782002 |
| *ENSGALG00000004239* | 2.90991951 | 8.55082855 | 1.07E-08 | 3.77E-06 |
| *ENSGALG00000030769* | 2.90276711 | 2.21588356 | 3.62E-05 | 0.00178533 |
| *SRGAP3* | 2.90142518 | 2.41126664 | 0.00012332 | 0.00395607 |

| | | | | |
|---|---|---|---|---|
| ENSGALG00000029240 | 2.89209758 | 1.74582862 | 0.004007 | 0.03581144 |
| ICE2 | 2.86923108 | 3.97459588 | 2.20E-07 | 4.04E-05 |
| LMOD3 | 2.86821768 | 2.42096884 | 0.00317279 | 0.03082788 |
| PHEX | 2.86045906 | 6.14307253 | 0.00025569 | 0.00634013 |
| ENSGALG00000002570 | 2.8592504 | 1.53319793 | 0.00049737 | 0.00995234 |
| PDZRN3 | 2.85770739 | 7.28752749 | 2.21E-09 | 1.25E-06 |
| PRRC2C | 2.85619205 | 9.22513741 | 6.27E-12 | 6.83E-09 |
| NECAB2 | 2.82643537 | 0.99218218 | 0.00374618 | 0.03434686 |
| ENSGALG00000027483 | 2.80144287 | 2.64320185 | 0.00055939 | 0.01067383 |
| BMP5 | 2.78795934 | 3.21155599 | 0.00378179 | 0.03453096 |
| ENSGALG00000012644 | 2.78570168 | 3.75033926 | 7.96E-05 | 0.00298628 |
| ENSGALG00000032304 | 2.78529915 | 4.87717549 | 0.00077741 | 0.01293785 |
| ENSGALG00000045042 | 2.78116565 | 0.15065594 | 0.00473164 | 0.03971471 |
| ENSGALG00000032493 | 2.76869452 | 1.09489666 | 0.00114608 | 0.01641051 |
| ESAM | 2.76737858 | 3.02206355 | 0.00127518 | 0.01746372 |
| TRIM71 | 2.7611578 | 5.50512413 | 1.48E-07 | 3.00E-05 |
| BLNK | 2.74993656 | 3.46066032 | 0.00011749 | 0.0038387 |
| ENSGALG00000031990 | 2.7451546 | 0.4153861 | 0.00678373 | 0.04990611 |
| ENSGALG00000030914 | 2.74110256 | 0.80011545 | 0.00657266 | 0.04875903 |
| SIX4 | 2.73856404 | 4.55140199 | 6.40E-05 | 0.00256938 |
| ENSGALG00000002431 | 2.73825821 | 3.76158335 | 0.00489405 | 0.04042735 |
| PLEKHG5 | 2.73685238 | 4.85370028 | 3.35E-05 | 0.00168681 |
| ENSGALG00000034119 | 2.73449205 | 7.71720478 | 0.00536689 | 0.04281589 |
| AEN | 2.73406213 | 3.01237646 | 0.00043666 | 0.00911118 |
| ENSGALG00000038923 | 2.73082735 | 2.92566921 | 6.45E-06 | 0.0005101 |
| ASB12 | 2.72957557 | 2.46732522 | 0.00398861 | 0.03581144 |
| SIRT7 | 2.70910474 | 0.80689338 | 0.00069343 | 0.01208572 |
| RSPO3 | 2.70627746 | 2.89932459 | 6.35E-06 | 0.00050802 |
| SLC4A4 | 2.69281523 | 1.63262515 | 0.00124058 | 0.01717271 |
| ENSGALG00000039417 | 2.67698712 | 3.18981712 | 0.0001355 | 0.00420375 |
| ENSGALG00000028273 | 2.66707638 | 6.6113368 | 0.00063411 | 0.01147157 |
| GEM | 2.65214695 | 3.98479611 | 4.20E-05 | 0.001928 |
| ENSGALG00000030329 | 2.63165234 | 1.25863365 | 0.00515812 | 0.04172206 |
| ENSGALG00000004758 | 2.62502748 | 2.22043596 | 0.00345889 | 0.03275297 |
| ENSGALG00000006681 | 2.62426285 | 2.29352892 | 0.00042237 | 0.00894641 |
| MYOM2 | 2.62298652 | 5.1968873 | 0.00438362 | 0.03783714 |
| ENSGALG00000031828 | 2.62217116 | 1.64683657 | 0.0002741 | 0.00659468 |
| NSUN7 | 2.60918478 | 1.69740921 | 0.00674081 | 0.04961612 |
| BUB1B | 2.60360889 | 6.64376607 | 1.38E-08 | 4.53E-06 |
| ENSGALG00000031943 | 2.6016563 | 0.88377423 | 0.00425005 | 0.03702303 |

| | | | | |
|---|---|---|---|---|
| *ENPP1* | 2.60158892 | 2.75117758 | 0.00246333 | 0.02635559 |
| *ENSGALG00000035219* | 2.59425159 | 1.77075789 | 0.00069147 | 0.01207679 |
| *ENSGALG00000036073* | 2.59288822 | 12.7118614 | 7.73E-05 | 0.00294157 |
| *DPF3* | 2.58393398 | 1.75959586 | 0.0056473 | 0.04404723 |
| *ENSGALG00000031597* | 2.57269941 | 8.5794544 | 0.00089978 | 0.01419091 |
| *TPM4* | 2.55098571 | 2.40359172 | 0.00035345 | 0.00784959 |
| *POPDC2* | 2.54376952 | 2.13277189 | 0.00526829 | 0.04229436 |
| *ENSGALG00000026301* | 2.53676709 | 2.62937969 | 0.00028789 | 0.00673194 |
| *ENSGALG00000001491* | 2.53507009 | 1.59678464 | 0.00395447 | 0.0356161 |
| *COL27A1* | 2.53484504 | 6.91688725 | 0.00025271 | 0.00632754 |
| *TENM1* | 2.52765865 | 2.74496349 | 0.00255177 | 0.02706141 |
| *MT-ATP6* | 2.51898994 | 13.1504572 | 2.09E-05 | 0.00121433 |
| *ARHGAP25* | 2.51749546 | 2.82574752 | 0.00125769 | 0.01729111 |
| *ENSGALG00000016356* | 2.51077239 | 1.37057622 | 0.00343264 | 0.03263844 |
| *MT-ND3* | 2.50733132 | 11.9103617 | 0.00167887 | 0.02045736 |
| *DEPTOR* | 2.50653039 | 3.38301352 | 0.00021789 | 0.00574009 |
| *NANOS1* | 2.49517857 | 1.91961592 | 0.00091145 | 0.01429527 |
| *RBBP6* | 2.49326693 | 9.27084477 | 8.13E-12 | 8.21E-09 |
| *ENSGALG00000033106* | 2.48472552 | 2.51161061 | 0.00244144 | 0.02622554 |
| *CEP55* | 2.48404082 | 4.29856117 | 8.11E-10 | 6.38E-07 |
| *DOK5* | 2.48301387 | 2.19208679 | 0.006611 | 0.04896641 |
| *CAVIN1* | 2.48196676 | 4.42933755 | 1.17E-06 | 0.00014191 |
| *MT-ND2* | 2.46904572 | 10.469919 | 3.10E-06 | 0.00029674 |
| *NPM3* | 2.46825936 | 4.56091049 | 7.82E-06 | 0.00056733 |
| *ENSGALG00000026613* | 2.46802337 | 3.440883 | 0.00164392 | 0.02015194 |
| *RBM41* | 2.45132573 | 3.14322676 | 0.00010987 | 0.0036746 |
| *MT-ND5* | 2.44752925 | 12.2828979 | 0.00015797 | 0.00470336 |
| *MT-CYTB* | 2.44481926 | 12.0762585 | 5.38E-05 | 0.00227118 |
| *ENSGALG00000037785* | 2.43558926 | 0.38539041 | 0.00382601 | 0.03485293 |
| *ENSGALG00000014412* | 2.42897699 | 4.93667143 | 0.00413068 | 0.03636387 |
| *NALCN* | 2.40492123 | 2.51944319 | 0.00246596 | 0.02635559 |
| *CARD11* | 2.40133381 | 2.39022645 | 0.00178129 | 0.02124782 |
| *CAB39L* | 2.39847666 | 7.55810627 | 6.75E-06 | 0.00051916 |
| *ENSGALG00000042020* | 2.39080112 | 7.38670039 | 9.74E-05 | 0.00341036 |
| *NOP56* | 2.3859024 | 7.9997136 | 1.10E-07 | 2.39E-05 |
| *AHDC1* | 2.37717978 | 2.86983949 | 8.86E-05 | 0.00321562 |
| *ALDOB* | 2.36842454 | 2.07721188 | 0.00170232 | 0.02065785 |
| *ENSGALG00000043925* | 2.3633488 | 2.75891292 | 0.00052075 | 0.01017539 |
| *GRTP1* | 2.35727551 | 3.17989708 | 0.00171883 | 0.02076534 |
| *KANK3* | 2.35030113 | 3.16572301 | 0.00106569 | 0.0156719 |

| | | | | |
|---|---|---|---|---|
| *ZFP106* | 2.33659511 | 4.92046316 | 4.57E-05 | 0.00202199 |
| *ENSGALG00000040521* | 2.3232156 | 3.02211419 | 0.00033553 | 0.00760699 |
| *ENSGALG00000017347* | 2.31869779 | 5.54533724 | 0.0047836 | 0.03994899 |
| *CACNA1C* | 2.31851132 | 4.36884951 | 0.00055334 | 0.01062162 |
| *TRMT12* | 2.31617642 | 3.75572812 | 3.28E-05 | 0.00167425 |
| *ENSGALG00000046614* | 2.29460703 | 2.61477172 | 0.00100329 | 0.01516739 |
| *SYNPO2* | 2.28988519 | 4.48494914 | 0.00026205 | 0.00641383 |
| *3110082I17RIK* | 2.28609288 | 4.39067011 | 1.54E-06 | 0.00017675 |
| *SIX1* | 2.27646573 | 5.77141551 | 3.67E-06 | 0.00034388 |
| *COL8A1* | 2.274595 | 4.04107745 | 0.00564928 | 0.04404723 |
| *ALOX5AP* | 2.27279421 | 3.72788811 | 0.00427303 | 0.0371318 |
| *ENSGALG00000043233* | 2.26813949 | 2.24891444 | 0.00097873 | 0.01495259 |
| *ENSGALG00000039432* | 2.26537983 | 2.01977059 | 0.00223929 | 0.02471081 |
| *ENSGALG00000040010* | 2.2594283 | 2.53183519 | 0.00655805 | 0.0487017 |
| *CREB3L2* | 2.25874862 | 6.57291881 | 9.20E-07 | 0.00012165 |
| *MT-CO2* | 2.252918 | 14.0667604 | 0.00019345 | 0.00536605 |
| *MYO6* | 2.24991031 | 8.246369 | 0.00011118 | 0.00369407 |
| *ENSGALG00000034312* | 2.24922645 | 4.04311482 | 0.00202169 | 0.02308464 |
| *PALMD* | 2.24840754 | 3.11921501 | 0.00434725 | 0.037615 |
| *NHSL2* | 2.24818858 | 2.56564653 | 0.00283418 | 0.02881267 |
| *ENSGALG00000040348* | 2.24296839 | 7.85489966 | 2.42E-06 | 0.00024805 |
| *PHACTR1* | 2.24231868 | 2.65522373 | 0.00228394 | 0.02514468 |
| *ENSGALG00000035309* | 2.24063484 | 2.29330683 | 0.00421185 | 0.03684915 |
| *MYO10* | 2.23639592 | 5.60420612 | 1.15E-09 | 7.77E-07 |
| *KIF14* | 2.23455661 | 3.04204782 | 0.00130455 | 0.01768443 |
| *CABP2* | 2.23092501 | 4.6827033 | 3.02E-05 | 0.00158067 |
| *LSP1* | 2.21680508 | 6.53134721 | 0.00200636 | 0.02300161 |
| *TGFBI* | 2.20525439 | 8.91186939 | 1.07E-05 | 0.00072694 |
| *SMOC2* | 2.19279687 | 4.0667332 | 0.00222425 | 0.02460281 |
| *ENSGALG00000038075* | 2.19028952 | 1.76593737 | 0.00563703 | 0.04404723 |
| *TTC25* | 2.18713089 | 1.74932646 | 0.0018975 | 0.02214654 |
| *POLQ* | 2.18104992 | 2.81343952 | 0.0029647 | 0.02961982 |
| *ENSGALG00000012068* | 2.17563557 | 4.62448122 | 3.12E-06 | 0.00029674 |
| *MT-ND1* | 2.16469751 | 12.5279112 | 0.00028038 | 0.00664633 |
| *THBS1* | 2.15649613 | 9.19191706 | 0.00011124 | 0.00369407 |
| *TRPV4* | 2.15052182 | 8.11259288 | 0.00523848 | 0.04217305 |
| *ENSGALG00000030587* | 2.14884897 | 4.18783618 | 0.00118871 | 0.01674968 |
| *SALL1* | 2.14514285 | 3.36402139 | 0.00514031 | 0.04160185 |
| *RAB3GAP1* | 2.14036663 | 6.3383724 | 1.47E-07 | 3.00E-05 |
| *ENSGALG00000006083* | 2.12978658 | 3.6406424 | 0.00011785 | 0.00384154 |

| | | | | |
|---|---|---|---|---|
| *TOGARAM1* | 2.12832436 | 4.07539341 | 7.74E-06 | 0.00056713 |
| *ENSGALG00000044664* | 2.1230069 | 4.09196415 | 0.00029913 | 0.00691463 |
| *ENSGALG00000031648* | 2.12131826 | 5.57644954 | 1.02E-06 | 0.00012895 |
| *ENSGALG00000038316* | 2.11483178 | 2.67139493 | 0.00096975 | 0.01491194 |
| *ENSGALG00000036033* | 2.11327075 | 1.59800432 | 0.00231339 | 0.02533094 |
| *ENSGALG00000023819* | 2.10912517 | 2.68502896 | 0.0023191 | 0.02534696 |
| *PBK* | 2.09581183 | 5.68726844 | 4.50E-05 | 0.0020093 |
| *ENSGALG00000004691* | 2.09294432 | 3.9698396 | 8.71E-05 | 0.00318244 |
| *ZFPM1* | 2.09010195 | 3.34680862 | 0.00028575 | 0.00673194 |
| *KNL1* | 2.07565632 | 3.68686964 | 0.00129263 | 0.01758652 |
| *ENSGALG00000036229* | 2.07558408 | 10.9182721 | 0.00021623 | 0.00571788 |
| *ENSGALG00000044419* | 2.07300793 | 1.9031642 | 0.00387684 | 0.03513505 |
| *MTR* | 2.06199602 | 3.81206854 | 0.00091538 | 0.01434089 |
| *MT-CO3* | 2.05690087 | 14.423504 | 0.00027109 | 0.0065509 |
| *ENSGALG00000043546* | 2.05533352 | 1.75874341 | 0.00451608 | 0.03861888 |
| *SLIT1* | 2.05387727 | 3.61413411 | 0.00459569 | 0.03904819 |
| *GM1673* | 2.05376358 | 3.52195042 | 0.00667538 | 0.04931416 |
| *WWP2* | 2.05148873 | 10.2676978 | 0.00104532 | 0.01547415 |
| *SGMS2* | 2.05024923 | 3.37062782 | 0.003505 | 0.03303484 |
| *EBF3* | 2.04928069 | 5.46278152 | 0.00015243 | 0.00460763 |
| *ENSGALG00000029899* | 2.04571155 | 4.90674453 | 0.00108484 | 0.01582185 |
| *ENSGALG00000043332* | 2.04106841 | 4.80753227 | 0.00035274 | 0.00784617 |
| *NEFL* | 2.03599309 | 3.16360107 | 0.00571628 | 0.04429408 |
| *SRGAP2* | 2.03439638 | 5.28206753 | 2.89E-06 | 0.0002844 |
| *TRAIP* | 2.03012457 | 4.89746878 | 0.00015189 | 0.00460118 |
| *ENSGALG00000010030* | 2.02714751 | 5.01920323 | 0.00026001 | 0.00639726 |
| *CEP128* | 2.02523082 | 3.71447667 | 0.00282101 | 0.0287529 |
| *ENSGALG00000039089* | 2.02138187 | 2.67952836 | 0.00082308 | 0.01343028 |
| *ANKRD1* | 2.01862732 | 1.92805207 | 0.00465255 | 0.03927186 |
| *FHL3* | 2.01814291 | 7.02511885 | 3.78E-05 | 0.00182699 |
| *ENSGALG00000029339* | 2.01278331 | 6.86196372 | 0.00046077 | 0.00944701 |
| *PRRX1* | 2.01197506 | 7.62335424 | 0.00025498 | 0.00634013 |
| *ENSGALG00000006751* | 2.00394205 | 3.40901083 | 0.00272404 | 0.02812918 |
